# Using Genomic and Transcriptome Analyses to Identify the Role of the Oxidative Stress Pathway in Renal Clear Cell Carcinoma and Its Potential Therapeutic Significance

**DOI:** 10.1155/2021/5561124

**Published:** 2021-10-20

**Authors:** Xiangyu Che, Xiaochen Qi, Yingkun Xu, Qifei Wang, Guangzhen Wu

**Affiliations:** ^1^Department of Urology, The First Affiliated Hospital of Dalian Medical University, Dalian 116011, China; ^2^Department of Endocrine and Breast Surgery, The First Affiliated Hospital of Chongqing Medical University, Chongqing 400042, China

## Abstract

Oxidative stress (OS) refers to endogenous and/or exogenous stimulation when the balance between oxidation and antioxidants in the body is disrupted, resulting in excessive production of free radicals. Excessive free radicals exert a series of negative effects on the body, which can result in the oxidation of and infliction of damage on biological molecules and further cause cell death and tissue damage, which are related to many pathological processes. Pathways related to OS have always been the focus of medical research. Several studies are being conducted to develop strategies to treat cancer by exploring the OS pathways. Therefore, this study is aimed at determining the correlation between the OS pathway and kidney renal clear cell carcinoma (KIRC) through bioinformatics analysis, at proving the effect of common anticancer drugs on the OS pathway, and at constructing a prognosis model of patients with KIRC based on several genes with the strongest correlation between the OS pathway and KIRC. We first collected and analyzed gene expression and clinical information of related patients through TCGA database. Then, we divided the samples into three clusters according to their gene expression levels obtained through cluster analysis. Using these three clusters, we performed GDSC drug analysis and GSEA analysis and examined the correlation among the OS pathway, histone modification, and immune cell infiltration. We also analyzed the response of anti-PD-1 and anti-CTLA-4 to the OS pathway. Thereafter, we used LASSO regression to select the most suitable nine genes, combined with the clinicopathological characteristics to establish the prognosis model of patients with KIRC, and verified the scientific precision of the model. Finally, tumor mutational burden was calculated to verify whether patients would benefit from immunotherapy. The results of this study may provide a reference for the establishment of treatment strategies for patients with KIRC.

## 1. Introduction

Oxidative stress (OS) is usually caused by an imbalance between the cellular antioxidant mechanism and oxidative free radical substances produced by metabolism. This imbalance leads to the accumulation of excessive reactive oxygen species (ROS) (including O^2-^, H_2_O_2_, OH^−^, O_3_, and other oxygen-containing free radicals) in the body cells, causing irreversible or reversible damage to the structure and substances of cells [1]. Since there are various antioxidant mechanisms and enzymes in the body, such as superoxide dismutase, peroxidase, and glutathione peroxidase, for the removal of excess ROS, the normal physiological level of ROS does not cause harm to the body [2]. However, when these corresponding mechanisms are abnormally inactivated, excessive ROS levels are generated that attack the components and structure of cells, causing detrimental effects, such as instability of cell membranes and structural damage to genetic materials, DNA, and proteins [3].

The Nrf2 pathway plays a core regulatory role in the OS response [4]. Nrf2 (encoded by the NFE2L2 gene) regulates the expression of approximately 250 genes involved in cell homeostasis, including antioxidant proteins, detoxifying enzymes, drug transporters, and several cell-protective proteins. Nrf2 targets genes involved in cellular defense and contains antioxidant response elements (ARE), which encode antioxidant enzymes (glutamate-cysteine ligase (GCL)), drug metabolic enzymes (cytochrome P450, glutathione S-transferase (GST)), chaperone DNA repair enzymes, and proteasome subunits. Nrf2-mediated gene transcription depends on Nrf2 heterodimerization with small Maf proteins (MafG, MafK, and MafF), which is necessary for effective binding of ARE and electrophilic response element. Transcription of these protective genes enables cells to maintain redox equilibrium and to eliminate proteins that are damaged under oxidative and allogeneic stress conditions [5]. Studies have shown that Nrf2 also regulates the synthesis of SOD, CAT, and other enzymes that remove excess ROS to regulate antioxidant activities in the body [6, 7].

On the one hand, Nrf2 regulates cellular oxygen reduction balance to combat heterologous substances and oxidative damage. On the other hand, it reduces the damage caused by OS by reducing the sensitivity of cells to stressors. Thus, Nrf2 can be considered a protective gene in normal cells [8]. However, Nrf2 confers protection to cancer cells from ROS attacks, resulting in the failure of treatments, such as chemotherapy and radiotherapy that are used to eliminate the cancer cells by facilitating the production of substantial amounts of ROS [9]. Thus, Nrf2 is considered a risk factor in cancer cells. Owing to the dual nature of NRF2, the role of the mechanism of OS in the occurrence and development of cancer is vague. This poses challenges for studies conducted to target the OS pathways for cancer treatment.

According to Globocan 2018 estimates of cancer incidence and mortality, compiled by the International Agency for Research on Cancer, kidney cancer incidence and mortality were 2.2% and 1.8%, respectively [10]. The most commonly reported type of kidney cancer is kidney renal clear cell carcinoma (KIRC), accounting for approximately 85% of the cases. In KIRC, hemorrhages, necrosis, cystic degeneration, andcalcification are often observed in the renal parenchyma. Following growth, it infiltrates, compresses, and destroys the renal pelvis and calyces; furthermore, it develops outside the renal capsule and results in the formation of hemangioma emboli or metastasizes to lymph nodes and other organs. KIRC presents with a poor prognosis and high mortality rates. Therefore, more attention has been focused on clinical treatment.

In this study, more than 30 genes closely related to OS were selected using the REACTOME database, including multiple pathway genes and gene families such as NFE2L2, SOD, CAT, and NOX [11], to represent the expression of the OS pathway. The correlation between its expression level in KIRC and the occurrence and progression of KIRC and the clinicopathological characteristics were explored using bioinformatics analysis techniques to accurately decipher the role of OS mechanisms in KIRC. Concurrently, we used LASSO regression to screen out genes with a good fit for the establishment of the prognostic model of KIRC. These results can provide a valuable reference for the development of drugs targeting the OS pathway and for the treatment of OS.

## 2. Materials and Methods

### 2.1. Data Acquisition and Analysis

Based on the REACTOME dataset (https://reactome.org/) [12] and the GSEA website (http://www.gsea-msigdb.org/gsea/index.jsp) [13], 32 genes strongly associated with the OS pathways were identified and the genes were selected for further analysis. Mutations in these genes were studied to investigate their effect on the development of cancer. The genetic and clinical data for cancer were obtained from TCGA database projects (https://tcga-data.nci.nih.gov/tcga/) [14]. There were a total of 32 cases, each representing a type of cancer. TCGA database is aimed at establishing a comprehensive map of tumor genes through large-scale gene sequencing and comprehensive, multidimensional analysis to identify the genetic changes caused by the occurrence and development of tumors. To obtain data on the genes, we included the CNV and SNV of genes, gene expression levels, clinical survival landscape, and clinicopathological features. Gene expression data were analyzed using Perl and visualized using the TBtools software for observation. Survival curves were plotted for all significant genes associated with KIRC, visually representing the specific role played in KIRC development. Statistical significance was set at *p* < 0.05.

### 2.2. Analysis of SOD2 Expression Using the GEPIA Website

GEPIA is currently a well-known online tool used for visualization analysis using TCGA data [15]. It is simple and effective with good operability. For the gene SOD2, which is known to directly regulate the process of OS, the GEPIA website (http://gepia.cancer-pku.cn/) combined with TCGA and GTEX databases was used to compare the differences in gene expression between normal tissues and cancer tissues in a variety of cancers. Statistical significance was set at *p* < 0.05.

### 2.3. Generation of the Plot of Protein-Protein Interaction (PPI) Using STRING

STRING is a database platform that is used to explore interactions between proteins [16]. It includes both direct physical interactions between proteins and indirect functional correlations between proteins. The STRING platform (http://string-db.org/) was used to map the protein-protein relationships among these 32 genes. Cytoscape was used to identify the protein-protein interaction (PPI) network obtained from the platform. The relationships between these genes are shown and explained at the protein level.

### 2.4. Derivation of Three Clusters Based on Cluster Analysis

The OS score (OS score) was obtained from the data on mRNA expression levels, which was used to quantify the expression of genes in the OS pathway. Based on the OS score, we used cluster analysis, termed as “unsupervised hierarchical clustering,” to obtain the following three clusters of samples: Cluster1 (high expression of the OS pathway gene), Cluster2 (normal expression of the OS pathway gene), and Cluster3 (low expression of the OS pathway gene). Three clusters represent different expression levels of OS-related genes, and subsequent studies are based on these three clusters. To verify the accuracy of the three clusters, we used a violin diagram to verify and illustrate their gene enrichment and subsequently compared the survival curves of triclusters, summarized the gene expression level and the clinicopathological characteristics of the three clusters, and used a heatmap to depict the obtained results. Statistical significance was set at *p* < 0.05. We used “gplots” in R for cluster analysis and “survival” in R to plot the survival curve of the three clusters. A heatmap was generated using “pheatmap” in R to describe the relationship among the three groups of clusters and the clinicopathological characteristics of patients with KIRC. The cluster analysis is based on the “ward.D” algorithm. We used the drawing software package in R to construct a violin plot.

### 2.5. GSEA Analysis of the Three Clusters

To determine the enriched gene sets in each cluster, GSEA analysis was performed using the GO database. GSEA analysis was used to interpret the genome-level expression data and to analyze their common biological functions. We selected 30 upregulated and downregulated pathways from Cluster1, Cluster2, and Cluster3, respectively, and used them to generate a heatmap of the pathways to display different gene sets enriched in different clusters. “ClusterProfiler” and “GSVA” in R were used to perform the analysis, while “gplots” and “pheatmap” in R were used to plot the data obtained.

### 2.6. Histone Modification-Related Genes and Common Oncogenes

Several classical or newly discovered oncogenes, such as VHL and EGFR, have been considered to refine our perspective. We analyzed the expression levels of classical oncogenes in the tricluster and generated a heatmap to intuitively reflect their different expression patterns in different clusters. The same method has also been used to explore genes related to histone modifications, such as the sirtuin family genes and HDAC family genes. Histone modification is directly related to gene mutations. Histone modification usually refers to methylation and acetylation. Sirtuin (SIRT) [17] is a highly conserved class of deacetylases from bacteria to humans, and histone deacetylase (HDAC) [18] is a protease that plays an important role in chromosome structural modification and gene expression regulation. Statistical significance was set at *p* < 0.05.

### 2.7. Application of the pRRophetic Algorithm Based on the GDSC Database

GDSC is a public database that presents an integration of drug, gene, and tumor information [19]. It provides free and publicly available genomic data for the formulation of cancer therapy and is committed to discovering potential cancer therapeutic targets to improve cancer therapy. It is the largest public database of its kind in the world. The GDSC database was used to predict chemotherapy responses. Several classic and novel targeted drugs were selected to treat KIRC tumor cells, including pazopanib, sorafenib, sunitinib, nilotinib, vorinostat, axitinib, gefitinib, temsirolimus, lapatinib, metformin, bosutinib, and tipifarnib. We used the “pRRophetic” package to implement the pRRophetic algorithm in R to estimate the IC50 of samples in three clusters through ridge regression [20]. All parameters were set by using default values with the removal of the batch effect of “combat” and tissue type of “allSoldTumours,” and duplicate gene expression was summarized as the mean value. Based on the results obtained, we illustrated a box diagram to visually indicate the different IC50 values of each drug in the three clusters and the correlation between them. Statistical significance was set at *p* < 0.05.

### 2.8. Immune Cell Infiltration and Immunotherapy

Many common immune cells were quantified using ssGSEA analysis in combination with TCGA database. The results of their correlations are expressed in the form of a heatmap. ssGSEA analysis can be performed by applying data on genetic signals expressed by a population of immune cells to a single sample. The 29 immune cells and regulators used in this study included the molecules involved in innate and adaptive immunity. The correlation between 29 immune cells/regulators and OS-related genes was depicted directly using a histogram. Data on APC-costimulation and macrophages were separately selected to highlight the correlation with OS score through a scatter diagram in regression analysis. Five R software packages, namely, “data.table,” “dplyr,” “tidyr,” “ggplot2,” and “ggstatsplot,” were used to analyze and to generate the figure. Two types of immune regulatory factors related to T cell-killing tumor cells, PD-1 [21], and CTLA-4 [22] have been reported. The correlation among CTLA-4, PD-1, and OS score was demonstrated through visual correlation matrix analysis. The results of the regression analysis can be visualized in the matrix. TIDE was used to predict the inhibitory response of a single sample immune checkpoint, and a submap was used to predict the subtype immune response. The two analyses were used to assess the possibility of PD-1 and CTLA-4 exhibiting responses to the agonists and inhibitors of OS-related genes. The Bonferroni correction was used to correct the *p* values at the test level. “pheatmap” in R was used to plot a heatmap.

### 2.9. Differentially Expressed Genes and LASSO Regression Analysis

Differential expression in normal and KIRC tissues is shown via a heatmap using “pheatmap” in R. “corrplot” in R was used to describe the coexpression relation between any two of the OS pathway genes. The regression relationship between SOD2 and other genes is shown separately in the scatter plots. Hazard ratio analysis was performed to analyze the relationship between the pathway and the progression of KIRC and to better understand the prognostic role of OS-related genes in ccRCC. We performed a univariate Cox regression analysis of the expression of OS-related genes using TCGA database. The correlation between high gene expression and patient survival rate was determined by analyzing the HR values in the results. The results are shown using a forest map. LASSO regression curve generated using the “glmnet” package was used to establish a risk model. We determined the cut-off value of each risk score in the tumor group using the “survminer” package. We divided the samples into the high-risk and low-risk groups based on the best cut off value. Two survival curves representing the high- and low-risk groups were obtained using the “survival” package in R studio. The “survival-ROC” software package is used to plot the curves of ROC and to obtain the value of AUC. The expression levels of nine genes selected through LASSO regression analysis are shown using a heatmap illustrated with the clinicopathological features.

### 2.10. Establishment and Verification of the Prediction Model

Sankey diagram plotted using the “ggalluvial” software package shows the attributes of expression and prognosis among selected genes. HPA (https://www.proteinatlas.org/) presents a complete map of protein levels in all major tissues and organs of the human body. Data on the protein levels of SOD2 were obtained from the HPA website and were used to verify the prediction of SOD2. GSEA with the REACTOME database revealed the enrichment of genes related to OS. Univariate and multivariate Cox regression analyses were performed to indicate the correlation of age, stage, grade, T (tumor), M (metastasis), and risk score in the model. N (node) was not included in the analysis because the sample quantity was not substantial enough to support the study. A nomogram was constructed using the “rms” software package in R. All statistical analyses were performed using the R studio. Statistical significance was set at *p* < 0.05. GSEA with the GO and KEGG databases helped elucidate the pathway by which the OS-related genes of the different risk groups were enriched.

### 2.11. Tumor Mutational Burden

Tumor mutational burden (TMB) [23] is an emerging biomarker considered for predicting ICI treatment response. TMB is often reported as the number of mutations in 1 Mb. First, we calculated the TMB value of each sample and analyzed the correlation between TMB and OS score. The samples were divided into two groups, namely, H-TMB and L-TMB, according to different TMB values. Survival was then predicted based on the OS score, and the survival curve was plotted. Finally, heatmaps were used to illustrate the results.

## 3. Results

### 3.1. Genetic Mutations of Oxidative Stress Pathway Genes Are Widespread in Cancers

**Figure 1:**
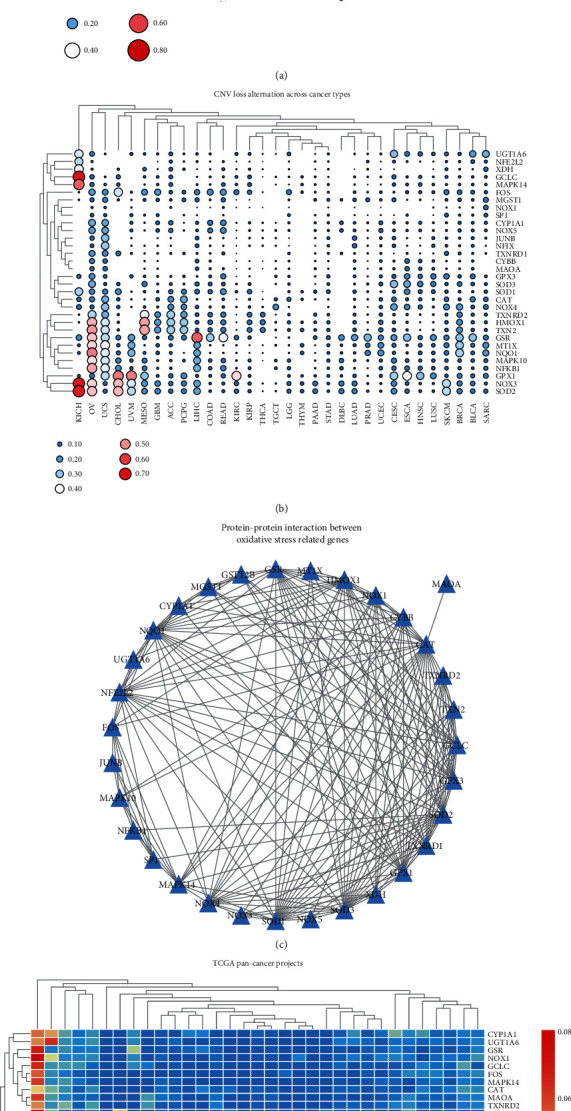
The results of gene expression derived from TCGA database. (a) Heatmap shows the level of the CNV gain mutations of the 32 genes in 32 types of cancer. The area and color of a circle show the level of gain mutation; the larger and closer the circle to a warm color, the higher its level. (b) Heatmap showing the level of the CNV loss mutations of the genes. The area and color of a circle show the level of loss mutation, the same as those in (a). (c) The PPI network diagram showing the interaction among the 32 oxidative stress- (OS-) related genes. (d) Heatmap showing the level of the SNV frequency of the genes; the color bar on the right side shows cool colors that indicate a lower level and warm colors that indicate a higher level of frequency.

**Figure 2:**
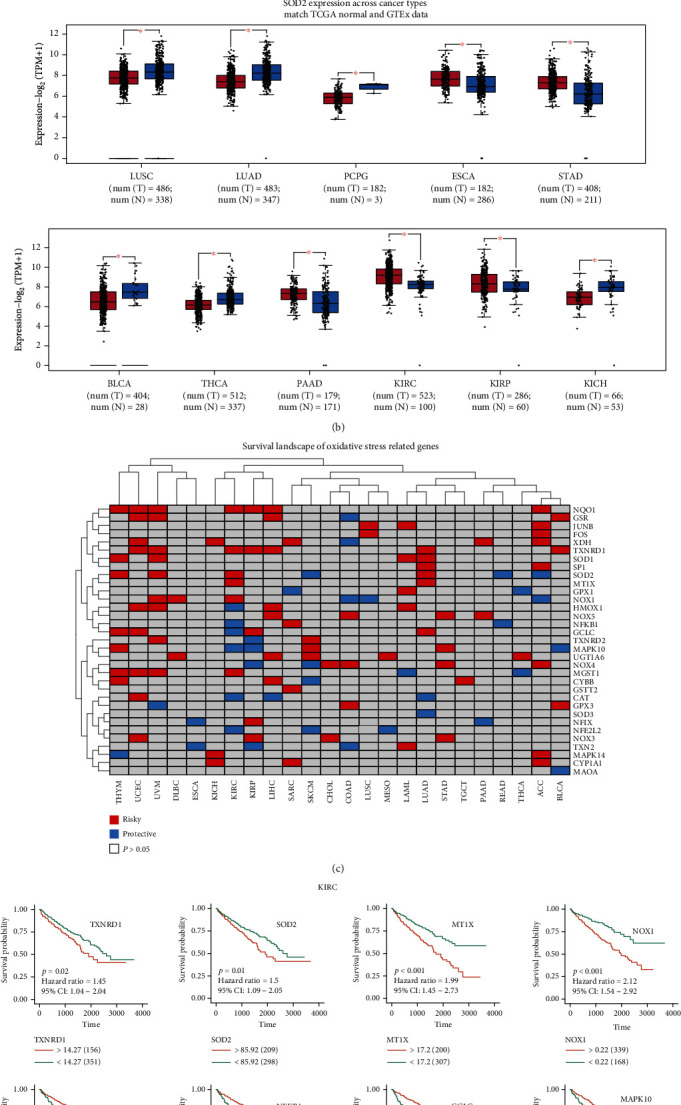
(a) Heatmap showing the expression of oxidative stress-related genes. According to the color bars provided, red indicates the high expression of genes and blue indicates the low expression of genes on the opposite; grey indicates no significance. (b) Boxplot of SOD2 expression level in both tumor and normal tissues. The data was derived from TCGA and GTEx databases; the red asterisk indicates significance. (c) Heatmap showing the nature of OS-related genes in cancer. According to the color blocks provided, red represents risky genes, blue represents protective genes, and grey represents no significance (*p* > 0.05). (d) The survival curve of the high-risk and low-risk groups in 10 genes of significance in KIRC.

Copy number variation (CNV) and single-nucleotide variation (SNV) data were downloaded from TCGA database and analyzed using Perl and R language. According to the results, MGST1, GPX3, SP1, TXNRD1, MAPK14, and SOD1 presented with CNV gains among various types of cancers. Meanwhile, GCLC, MAPK14, HMOX1, TXN2, GSR, GPX1, NOX3, and SOD2 showed CNV loss (Figures 1(a), 1(b), and 1(d), Tables S1 and S2). To better explain the relationship between genes, we used PPINETWORK to describe the relationship between genes via the application of STRING (Figure 1(c), Table S5). Gene expression investigations revealed that most genes were either upregulated or downregulated (Figure 2(a), Table S3). The survival landscape shows that NQO1, TXNRD1, SOD2, MT1X, NOX1, and MGST1 are risk genes for KIRC and HMOX1, NFKB1, GCLC, MAPK10, CAT, and NFE2L2 are protective genes in KIRC (Figure 2(c)). Survival curves also provided concrete evidence (Figure 2(d), Table S4). The expression of SOD2 in different cancers listed on the GEPIA website showed high values in KIRC (Figure 2(b)).

### 3.2. Obtainment of Three Clusters of Samples Using OS Score

**Figure 3:**
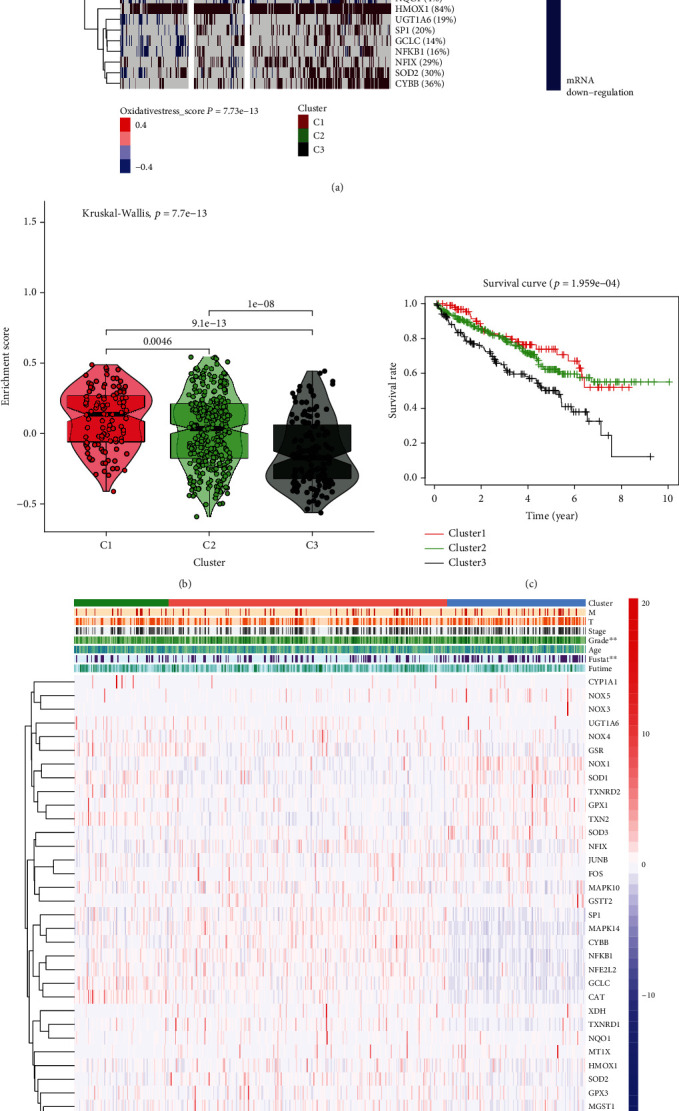
(a) Three clusters of the KIRC sample data derived from TCGA database according to the OS score; the clusters are represented in red (C1), green (C2), and black (C3), respectively. The level of OS score depends on the expression level of mRNA, which is shown using the heatmap. (b) Violin plot illustrating the enrichment score of the genes in three clusters. (c) The survival curves of three clusters, showing the difference in survival. (d) The heatmap showing the correlation between OS score and the clinicopathological characteristics (T, M, stage, grade, age, fustat, and futime) of patients with KIRC (^∗^*p* < 0.05, ^∗∗^*p* < 0.01, and ^∗∗∗^*p* < 0.001).

Through cluster analysis, all samples were divided into three clusters according to the mRNA expression of the samples, and different mRNA expression levels were expressed in the form of OS score (Figure 3(a), Table S6–S7). The clustering groups obtained were C1, C2, and C3, which represented OS score active, normal, and inactive, respectively. The violin diagram and survival curve highlight the characteristics of these three groups of samples; the survival value of the OS pathway upregulation was high, and the survival value of the OS pathway downregulation was low (Figures 3(b) and 2(c)). Using a heatmap, a close correlation was revealed among the OS pathway genes in the three clusters and grade and fustat in clinicopathology (Figure 3(d)).

### 3.3. GSEA of the Oxidative Stress-Related Pathway among the Three Clusters

**Figure 4:**
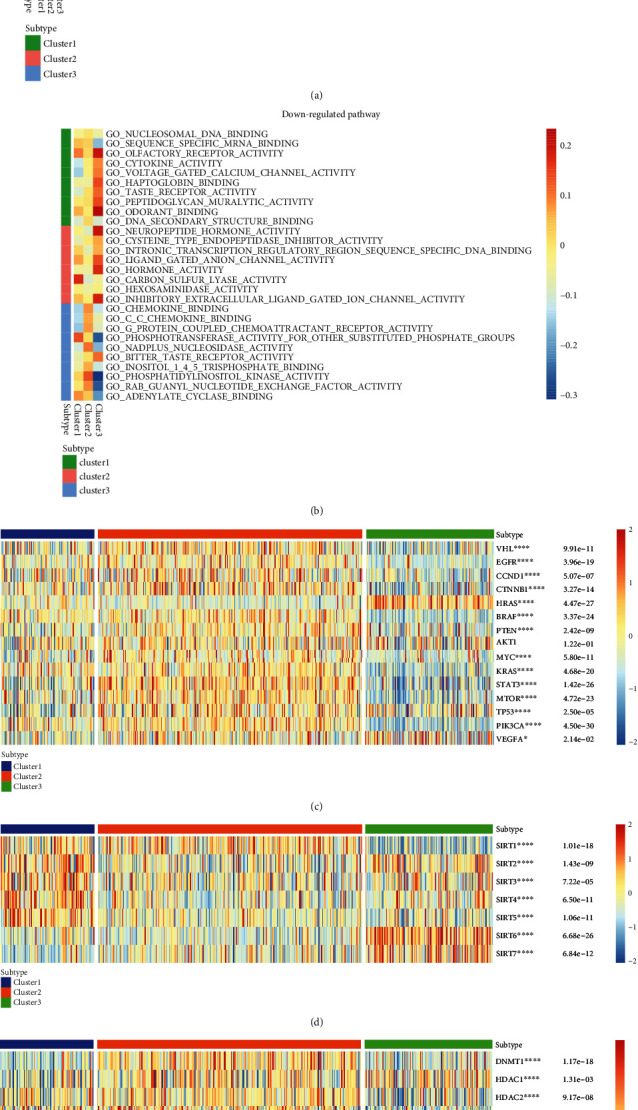
(a, b) Heatmap showing GSEA analysis of the enriched gene sets among the three clusters. The rows and columns are defined by using different gene sets based on GO analysis and three clusters successively. Cluster1, Cluster2, and Cluster3 are represented by green, red, and blue successively. Two figures have been used to show the results based on the regulation of the pathway, indicating upregulation and downregulation. Classical cancer pathway genes (c); sirtuin family genes (d); HDAC family genes (e); the expression of both in KIRC is shown using the heatmap (^∗^*p* < 0.05, ^∗∗^*p* < 0.01, ^∗∗∗^*p* < 0.001, and ^∗∗∗∗^*p* < 0.0001). Blue represents Cluster1, red represents Cluster2, and green represents Cluster3. The color bar on the right indicates that warm colors represent high gene expression, while cool colors represent low gene expression (red to blue).

Through GSEA analysis of the three clusters, we selected the 60 most representative gene sets from the three clusters. A pathway heatmap was used to reveal the different genes enriched in the different clusters. We divided the 60 gene sets into upregulated and 30 downregulated genes according to the expression of different genes. According to the results, the acyl-CoA dehydrogenase and NADH dehydrogenase pathways were enriched in Cluster1, and the odorant-binding protein and benzodiazepine receptor pathways were enriched in Cluster3 in terms of upregulation (Figure 4(a)). Additionally, the voltage-gated calcium channel pathway was enriched in Cluster1 and the phosphatidylinositol kinase and phosphotransferase pathways were enriched in Cluster3 (Figure 4(b)).

### 3.4. Expression of Other Common Genes in Three Clusters

We continued to use the three clusters obtained through cluster analysis to study the correlation among common renal cell carcinoma-related genes, sirtuin family genes, HDAC family genes, and KIRC (Figures 4(c)–4(e)). Sirtuin and HDAC are closely related to histone modification [24]. According to the heatmap generated and the *p* value obtained, most common genes of KIRC were found to be strongly correlated with the occurrence and development of tumors, except AKT1, which regulates cell proliferation and growth and is involved in cellular processes, including apoptosis and glucose metabolism (^∗^*p* < 0.05, ^∗∗∗∗^*p* < 0.001). All sirtuin family genes and most genes of the HDAC family (except HDAC5 and HDAC6) also have a strong correlation with the occurrence and progression of tumors.

### 3.5. Anticancer Drug Analysis Using GDSC

**Figure 5:**
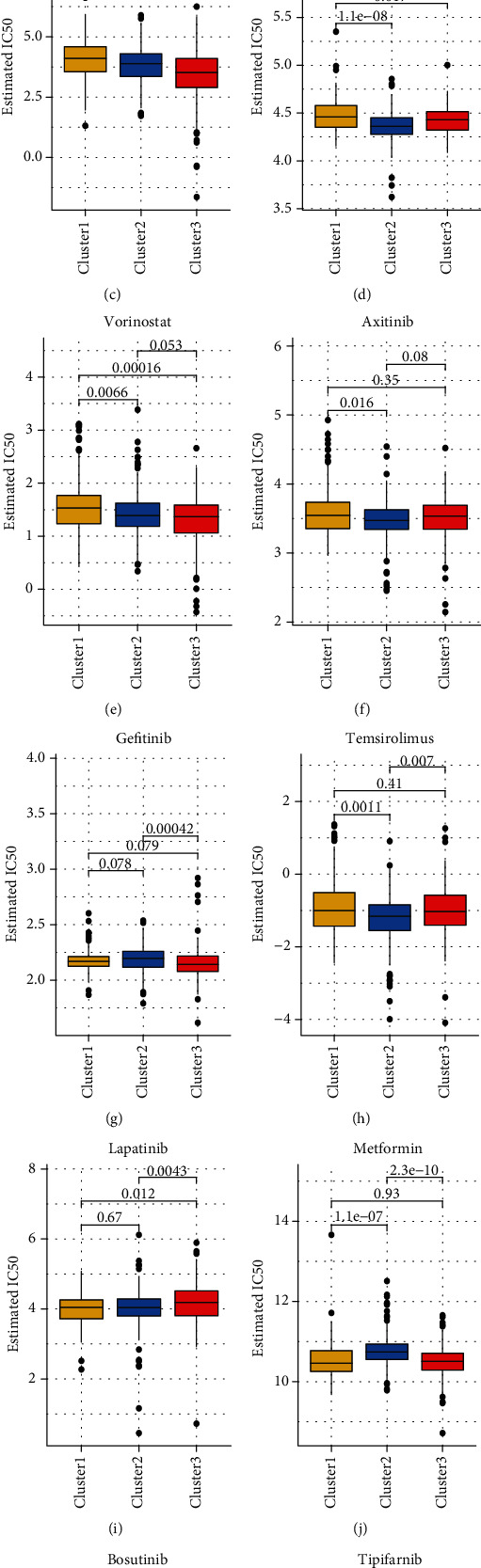
(a–l) Boxplots showing the estimated IC50 of cancer cells of the common anticancer drugs. The yellow, blue, and red boxes represent Cluster1, Cluster2, and Cluster3 successively. The value on the horizontal line represents the *p* value; *p* < 0.05 is considered significantly different. The 12 types of chemotherapeutic agents considered for analysis are pazopanib, sorafenib, sunitinib, nilotinib, vorinostat, axitinib, gefitinib, temsirolimus, lapatinib, metformin, bosutinib, and tipifarnib.

The GDSC database was used to analyze the IC50 of tumor cells subjected to treatment with different targeted anticancer drugs. Different reactions of the three clusters revealed the efficacy of different drugs influenced by the OS pathway. The results are shown as boxplots. The results are summarized into the following categories: C1 and C3 > C2 (pazopanib, nilotinib, axitinib, temsirolimus, and bosutinib), C1 and C3 < C2 (sorafenib, gefitinib, metformin, and tipifarnib), C1 > C2 > C3 (sunitinib, vorinostat), and C1 < C2 < C3 (lapatinib) (Figures 5(a)–5(l)).

### 3.6. Immune Cell Infiltration and Immunotherapy

**Figure 6:**
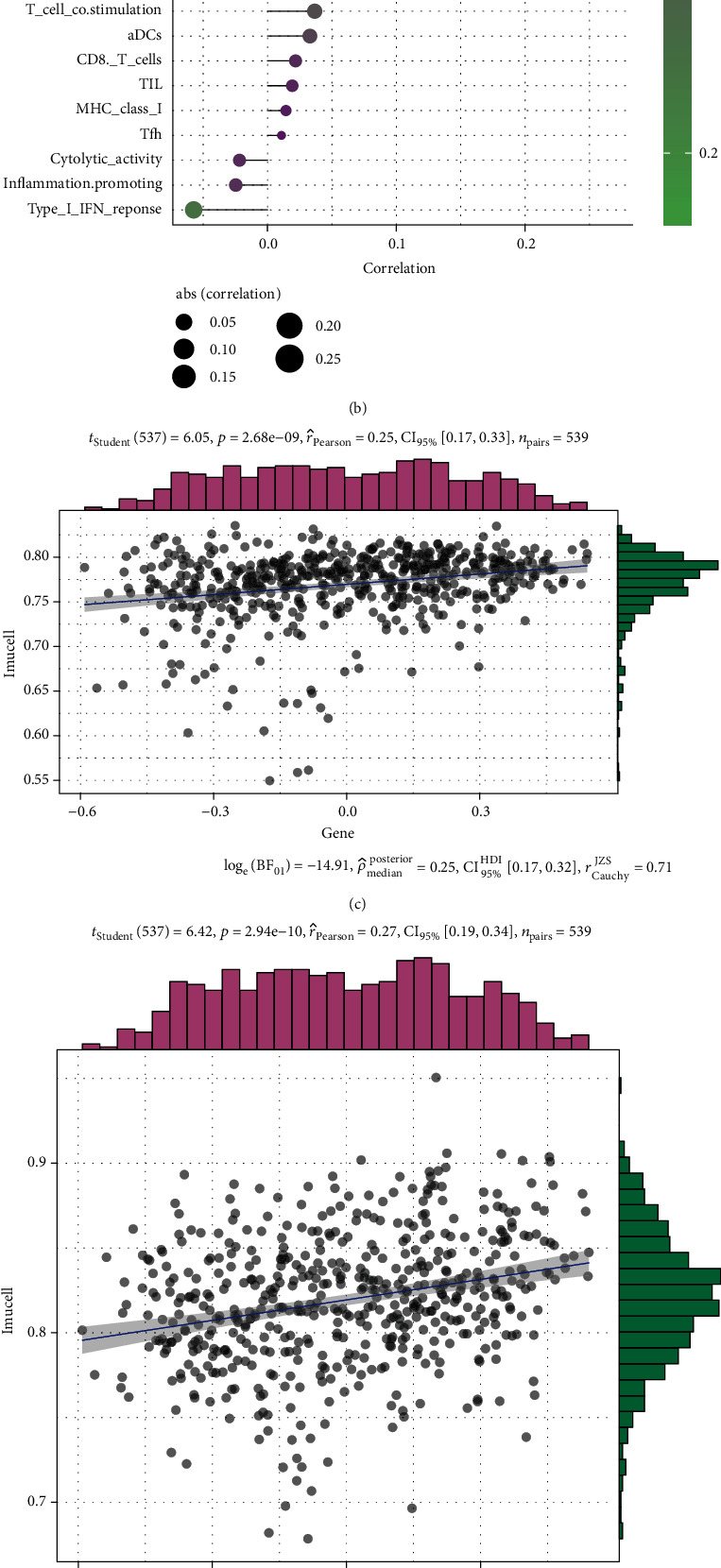
(a) Heatmap showing the correlation between the immune infiltration and the OS-related genes. The level of correlation is represented with colors; red represents positive correlation, and blue represents negative correlation (^∗^*p* < 0.05, ^∗∗^*p* < 0.01). (b) The plot shows the degree of correlation, the area of the circle represents the abs (correlation), and the color bar on the right side shows the *p* value. (c, d) APC-costimulation and macrophages were selected, and a scatter diagram has been used to show the correlation with OS score (OS score). (e) The plot shows the correlation analysis of PD-1, CTLA-4, and OS score. The scatter diagram and color bar represent the correlation and coefficient, respectively. (f) Submap analysis shows that the OS-inactive cluster could be more sensitive to cytotoxic T lymphocyte-associated protein 4 (CTLA-4) inhibitor (nominal *p* value = 0.01). However, data obtained after the Bonferroni correction indicated that it was not significantly different (*p* value is represented by the color block; purple indicates high values and yellow indicates low values).

Immunotherapy has always been the preferred method of cancer treatment. By examining the relationship between immunity and OS, we can understand the feasibility of treating KIRC with immunotherapy related to OS. The heatmap shows that immune cell infiltration strongly correlated with the OS pathway genes (Figure 6(a)). In order to explore the correlation between these genes and immune cell infiltration, it is well known that the universal marker for macrophages is CD68. Therefore, we used ten fresh kidney cancer tissues to perform real-time fluorescent quantitative PCR experiments to detect the correlation between SOD2, CAT, and the macrophage marker CD68. The results show that there is a clear positive correlation between SOD2 and CD68 and a clear negative correlation between CAT and CD68 (Supplementary Materials Figure S3). These results confirm our findings. The histogram shows the specific correlation coefficients. Infiltration of immune cells represented by neutrophils and macrophages exhibited a strong positive correlation with the OS pathway. The response of IFN-1 was negatively correlated with the OS pathway (Figure 6(b)). The scatter plot revealed that macrophage and antigen-presenting cell cosimulation demonstrated a strong correlation with the OS pathway (Figures 6(c) and (d)). Immune checkpoint-blocking antibodies, including anti-CTLA-4 and anti-PD-1, can induce tumor responses in a variety of tumor types [25, 26]. Therefore, the treatment of KIRC with immune checkpoint blocking antibodies is widely conducted [27]. In the correlation analysis, we found that OS score was negatively correlated with the expression of CTLA-4 and PD-1 (Figure 6(e)). Therefore, it can be inferred that patients with OS pathway inactivation may have higher expression of CTLA-4 and PD1 than patients with OS pathway activation. The submap algorithm of TIDE and GenePattern was used to predict the possibility of two different subtypes (Cluster1 + Cluster2 and Cluster3) exhibiting responses to immunotherapy. The results show that the OS inactive cluster is more promising regarding the generation of responses to anti-CTLA-4 therapy (Figure 6(f)). However, after data rectification using the Bonferroni correction, the difference between the two groups was not significant. This might be attributable to the small sample size.

### 3.7. LASSO Regression for the Establishment of the Prediction Model

**Figure 7:**
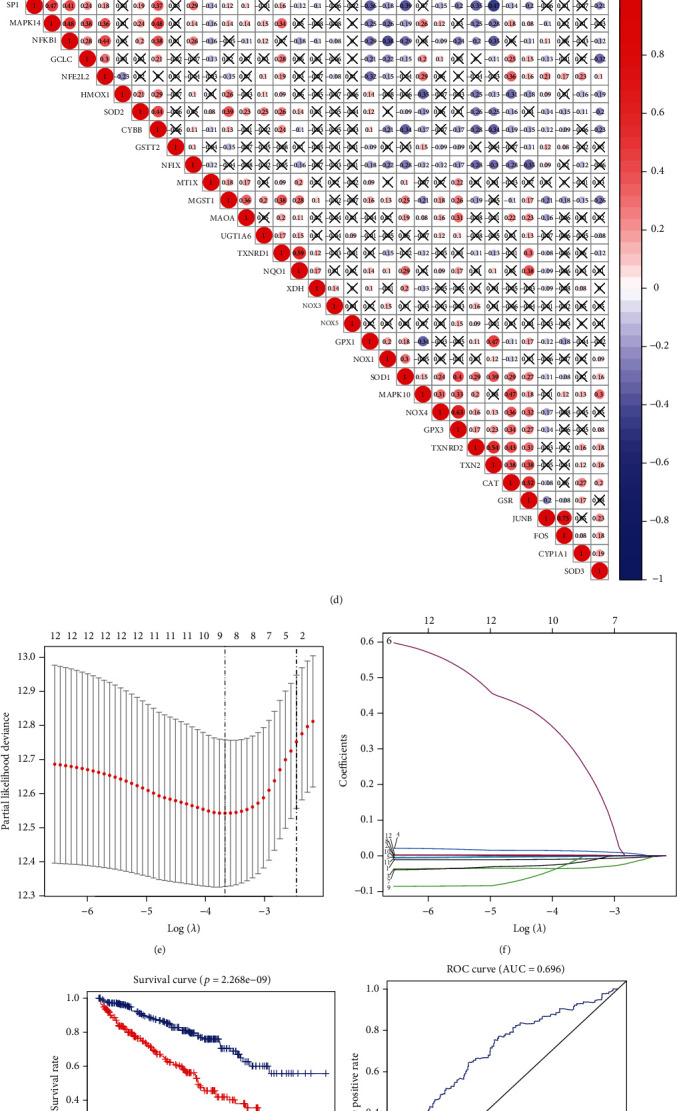
A predication model of KIRC using OS-related genes. (a) The expression of OS-related genes in patients with KIRC. In the color bar on the right side, red represents upregulation and blue represents downregulation. N (green) represents the normal sample; T (red) represents the tumor sample (^∗^*p* < 0.05, ^∗∗^*p* < 0.01, and ^∗∗∗^*p* < 0.001). (b) Hazard ratio analysis with 95% confidence intervals and *p* values for the OS-related genes. (c) The scatter diagram shows the correlation of SOD2 and other genes, including CYBB, MGST1, TXNRD1, UGT1A6, MAPK14, MAOA, FOS, CAT, MAPK10, SOD3, TXN2, and TXNRD2. (d) Coexpression analysis of OS-related genes in KIRC. (e, f) The LASSO coefficient profiles of OS-related genes in KIRC. Nine genes were selected using the LASSO Cox regression analysis. (g) The survival curve obtained using this model. Red and blue correspond, respectively, to the high-risk group and the low-risk group. (h, k) ROC curve represents data on 3, 5, 7, and 10 years. AUC of the curve is marked at the head (3 years: 0.696; 5 years: 0.734; 7 years: 0.702; and 10 years: 0.729). (l) Heatmap shows the correlation of the nine selected genes and the clinicopathological characteristics in two groups. The color bar represents the expression of genes; red represents upregulation and blue represents downregulation.

Based on the consideration of two groups, namely, the normal group (*n* = 72) and KIRC group (*n* = 539) from TCGA database, we found that the expression of 32 of the 33 OS pathway genes was significantly different between the two groups, as evidenced via analysis of gene expression except GCLC (^∗^*p* < 0.05, ^∗∗^*p* < 0.005, and ^∗∗∗^*p* < 0.001) (Figure 7(a)). The forest plot shows the results of hazard ratio analysis, the relationship between these gene pathways, and KIRC progression. NOX1, MT1X, SOD2, MGST1, TXNRD1, and NQO1 exert a risk effect, and MAPK10, HMOX1, GCLC, NFKB1, CAT, and NFE2L2 confer a protective effect (*p* < 0.05 was considered significant) (Figure 7(b), Table S8). The results of coexpression analysis revealed a correlation between two different genes, and we intercepted the correlation between SOD2 and other genes. CYBB, MGST1, TXNRD1, UGT1A6, MAPK14, and MAOA were positively correlated with SOD2 expression. FOS, CAT, MAPK10, SOD3, TXN2, and TXNRD2 were negatively correlated with SOD2 (Figures 7(c) and 7(d)). LASSO regression analysis was performed to select the appropriate genes to construct the prediction model. The samples were divided into two groups, namely, high-risk and low-risk groups, based on the best cutoff value of the risk score. Finally, the following nine genes were selected: CAT, GCLC, NFKB1, MAPK10, NOX1, MT1X, HMOX1, TXNRD1, and SOD2 (Figures 7(e)–7(g), Table S9). Receiver operating characteristic (ROC) curve analysis was then performed to analyze the predictive prognostic performance of the new survival model in patients with KIRC. We obtained the following four survival curves: 3 years with area under the curve (AUC) = 0.696, 5 years with AUC = 0.734, 7 years with AUC = 0.702, and 10 years with AUC = 0.729 (Figures 7(h)–7(k)). AUC > 0.7 was considered predictive [28]. The heatmap shows the correlation between the nine genes and the clinicopathological features (^∗^*p* < 0.05, ^∗∗^*p* < 0.005, and ^∗∗∗^*p* < 0.001) (Figure 7(l)).

### 3.8. Verification of the Prediction Models

**Figure 8:**
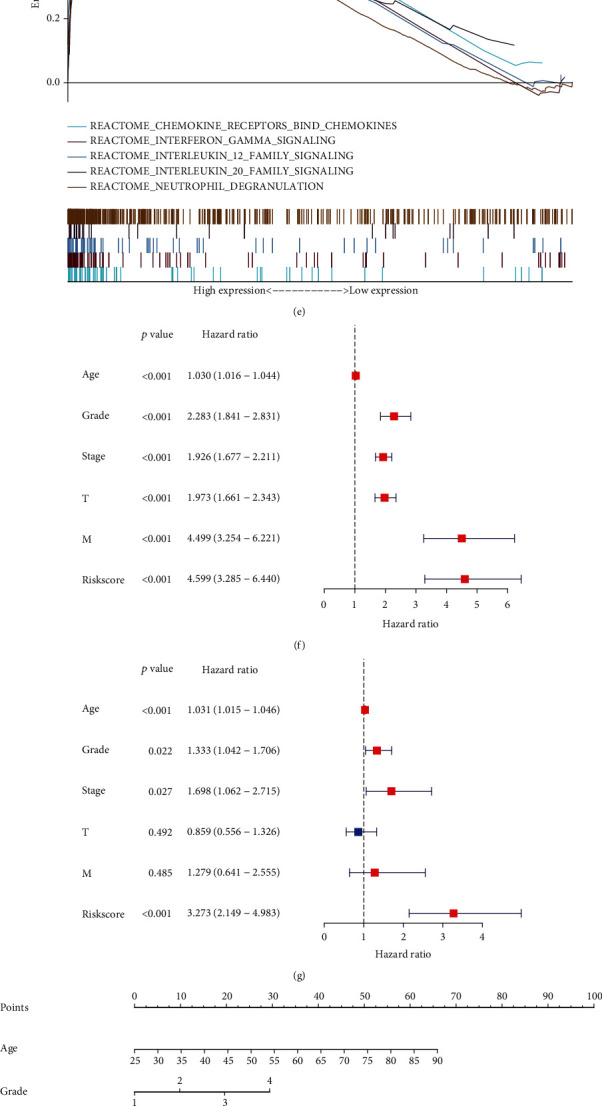
(a) Sankey diagrams used to summarize the data on the nine selected genes, including their expression and prognosis. (b–d) The association between OS-related genes and KIRC was confirmed at the protein level. The boxplot shows the SOD2 protein levels in normal tissues and primary tumor tissues. Immunohistochemical images obtained from the HPA website. (e) The curve shows the result of the gene enrichment score derived from the REACTOME database. (f) Forest plot of univariate Cox analysis. (g) Forest plot of multivariate Cox analysis. (h) Nomogram of the prediction model. The total score is calculated using ABC, from which 5-, 7-, and 10-year survival rates can be obtained. (i) The curve shows the enrichment of the different gene datasets in different physiological functions, including dystroglycan binding, immunoglobulin complex, immunoglobulin complex (circulating), immunoglobulin receptor binding, and phagocytosis, derived from the GO database. (j) The curve shows the result of gene enrichment in endocrine resistance, endocytosis, FoxO signaling pathway, lysosome, and oxidative phosphorylation derived from the KEGG database.

The Sankey diagram revealed the expression and prognosis of the nine selected genes (Figure 8(a)). The box plot of SOD2 protein expression showed that the protein expression of the tumor tissue was higher than that of the normal tissue (Figure 8(b)). The samples were obtained from the CPTAC [29]. We also obtained the immunohistochemical information on the proteins of selected genes from the Human Protein Atlas (HPA) website (Figures 8(c) and 8(d)). GSEA analysis was performed using the REACTOME, GO, and KEGG databases. The results showed that the highly expressed genes were mainly enriched in chemokine receptors, IFN-*γ*, IL-12, and IL-20, and neutrophil degranulation pathways (Figure 8(e), Table S10-11). The high-risk group and low-risk group genes were enriched in dystroglycan binding, immunoglobulin complex, immunoglobulin complex circulating, immunoglobulin receptor binding, and phagocytosis recognition in the GO database and endocrine resistance, endocytosis, foxO signaling pathway, lysosome, and oxidative phosphorylation in the KEGG database (Figures 8(i) and 8(j)). Univariate Cox regression analysis showed that age, grade, stage, risk score, and tumor node metastasis (TNM) without nodes existed as risk factors. Multivariate Cox regression analysis revealed that T and M were not significant (Figures 8(f) and 8(g), Table S12-13). Finally, the nomogram revealed the prediction model and the total points of age, grade, stage, and risk score derived from the survival ratio data of patients with KIRC in 5, 7, and 10 years (Figure 8(h)).

### 3.9. Tumor Mutational Burden

**Figure 9:**
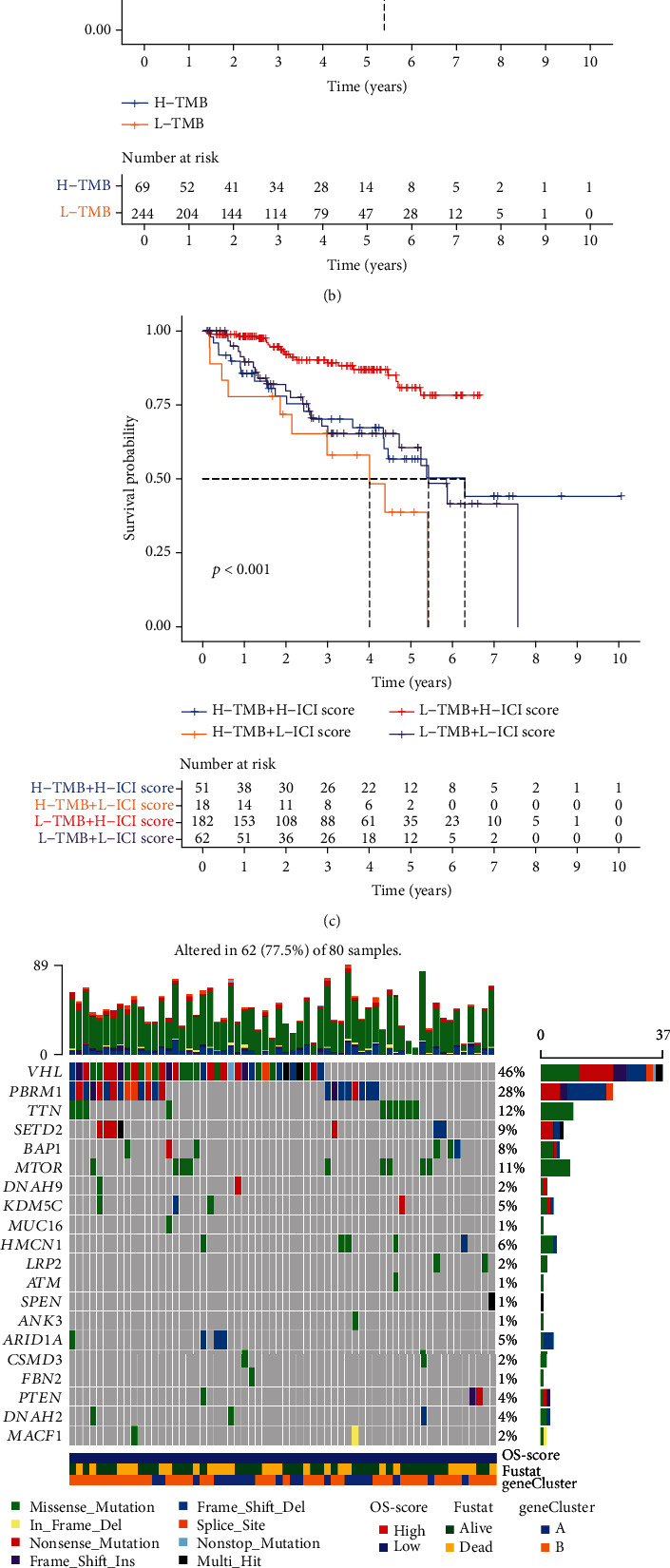
(a) Scatter plots illustrating the correlation between OS score and tumor burden mutation. The different colors represent the three clusters: Cluster1 (blue), Cluster2 (orange), and Cluster3 (red). (b) The survival curve of the high- and low-tumor mutation burden groups. (c) The survival curve of high- and low-tumor mutation burden with high- and low-ICI scores. (d, e) The analysis of gene mutation in ccRCC.

TMB levels are generally divided into three grades, namely, low TMB (1–5 muts/mb), intermediate TMB (6–19 muts/mb), and high TMB (≥20 muts/mb) [30]. The scatter plot shows the correlation between the OS score and TMB. It can be inferred from the scatter plot that there is a correlation between OS score and TMB, with a correlation coefficient of 0.28 (*p* < 0.05). In this plot, different clusters are represented with different colors to show the specific distribution of the different clusters (Figure 9(a), Table S14). The survival curve shows the comparison of survival rates among different TMB groups. It can be deduced from the figure that the survival rates of samples in the low-TMB group are better than those in the high-TMB group. When both OS score and TMB were considered, we found that the survival rate of the group with low TMB and high OS score was significantly better than that of the group with high TMB and low OS score, which corroborated the conclusions of the previous cluster analysis (Figures 9(b) and 9(c)). Based on the mutation rate of specific genes in the two groups of samples divided by the risk score, we illustrated a heatmap to show the correlation between them (Figures 9(d) and 9(e)). To improve the reliability of the research results, we conducted a random internal sampling validation in the KIRC dataset of TCGA database. Based on this risk model, we divided 50 randomly selected KIRC patients into the high-risk and low-risk groups. In the generated survival curve, we found that the prognosis of patients in the high-risk group was significantly lower than that in the low-risk group (*p* = 0.009) (Supplementary Materials Figure S1A). The expression of key genes such as CAT, NFKB1, MAPK10, NOX1, MT1X, HMOX1, TXNRD1, and SOD2 is also related to the poor prognosis of ccRCC patients (Supplementary Materials Figure S1B-I). In addition, the results of immunohistochemistry experiments on the SOD2 and CAT on our KIRC clinical specimens are also consistent with the above results, and the corresponding results are shown in Supplementary Materials Figure S2A-D. These results support our previous findings and make our conclusions more credible.

## 4. Discussion

Oxidation occurs at all times in our bodies. When air is inhaled, cells in our bodies utilize oxygen to trigger cellular processes to produce energy for survival. This is a typical chemical reaction occurring in the body. The process also produces free radicals and molecules that can damage our cells. Free radicals include ROS and other molecules with unpaired electrons, which render them unstable and highly chemically active. To achieve higher stability, free radicals damage molecules that constitute the DNA, proteins, and lipids (fats), leading to tissue damage [31]. In a few cases, a transient increase in ROS, a type of free radical, can act as a signaling mechanism that leads to a physiological cellular response, including OS. The concept of OS was first proposed in 1985 [3] and refers to a state of imbalance established between oxidation and antioxidant activity in the body, with a bias toward oxidation, leading to inflammatory infiltration of neutrophils, increased protease secretion, and the production of a substantial number of oxidative intermediates. OS is a negative effect produced by free radicals in the body and believed to be an important factor in aging and disease development. OS is feasible for the treatment of Alzheimer's disease [32], COPD [33], cardiovascular disease [34], and other diseases. ROS, which are recognized as free radicals, are the key products of OS. There is considerable evidence that the continuous production of ROS in the body can promote and inhibit the survival of cancer cells [1]. Increased production of ROS has been detected in a variety of cancers and has been shown to exert multiple effects. For example, they can activate protumorigenic signals, enhance cell survival and proliferation, and drive DNA damage and genetic instability. Conversely, ROS can also promote antitumor signaling and initiate OS-induced tumor cell death, and the redox balance between tumor cells and normal cells is altered, suggesting that ROS is a potential target for cancer therapy.

In this study, we focused on the genes and pathways related to OS and determined whether OS was a potential target for KIRC treatment by studying the correlation among gene expression and gene mutations and by utilizing clinical information reported for normal samples and KIRC samples in TCGA database. We studied the therapeutic effect of common cancer treatment drugs targeting the OS pathway in KIRC and selected the genes related to OS which were most closely associated with KIRC to establish a cancer prognosis model. TMB was calculated to verify whether the patients would benefit from immunotherapy [35]. At the beginning of the experiment, we determined the expression of genes in the OS pathway in patients with KIRC. In the heatmaps of cancer survival landscapes related to the OS pathway, we found that the ratio of risky and protective genes was 1 : 1. Therefore, we preliminarily concluded that the OS pathway was not completely carcinogenic or tumor-suppressive, as reported in previous studies. In the experiment, we focused on the SOD2 gene. SOD2 transcribes superoxide dismutase, an antioxidant that protects cells and mitochondria from ROS during inflammation [36]. Therefore, SOD2 can be used as a representative gene in the OS pathway. We studied the differential expression of SOD2 in cancer tissues and normal tissues, explored the hazard ratio of SOD2 with the survival curve, and analyzed the coexpression relationship between SOD2 and other genes in the later stage of the study, which collectively confirmed that SOD2 exerted a nonnegligible effect on KIRC. The GSEA analysis showed that many genes in the OS pathway were enriched in immune-related pathways; therefore, we conducted an in-depth study on the correlation between immune cell infiltration and the OS pathway. Previous studies have shown that the loss of SOD2 regulation can lead to abnormal T cell development and function [37–39]. In the heatmap of common KIRC-related gene expression and histone modification-related gene expression, we found that in addition to the common order of high-medium-low or low-medium-high, several genes exhibited the phenomenon of high-low-high or low-high-low in Cluster1 to Cluster3. This suggests that the expression of OS-related genes exerts a nonlinear effect on certain physiological phenomena. It is possible that any difference in OS gene expression, regardless of high or low expression, affects the expression of these oncogenes or histone modifications. The results of the analysis showed that the OS pathway was positively correlated with the antigen-presenting cell- (APC-) related pathway and macrophage pathway, which also suggested a relationship between OS and immunotherapy. As two checkpoints of cancer immunotherapy, PD-1 and CTLA-4 have attracted our attention [26]. In our study conducted on the responsiveness of three OS score clusters to PD-1 and CTAL-4 inhibitor targets, we combined Cluster1 and Cluster2 as OS-active clusters and defined Cluster3 as an OS-inactive cluster. The OS-inactive cluster is more promising in terms of exhibition of responses to anti-CTLA-4 therapy. Unfortunately, after correction using the Bonferroni test, the results lost their statistical significance, and we hypothesized that this might be due to the small sample size.

We then used LASSO regression analysis to select the genes that could be used to construct the prediction model. The nine selected genes were CAT, GCLC, NFKB1, MAPK10, NOX1, MT1X, HMOX1, TXRND1, and SOD2. CAT, a regulator of catalase, plays an important role in cancer tissues. The mutation of CAT leads to different resistance extents of cancer cells to ROS. Therefore, targeting the redox state of cancer cells by regulating the expression of catalase is a novel approach to enhance chemotherapy [40]. SOD2 and thioredoxin reductase 1 (TXNRD1), which regulate antioxidant enzymes, function based on the same principle. MAPK is a downstream pathway of Nrf2. It has been shown that Nrf2 can reduce the damage caused by OS to cells by regulating the MAPK pathway. The correlation between MAPK10 and OS has also been confirmed [41]. NF-*κ*B is an important transcription factor that regulates a variety of inflammatory factors and pathways. In Parkinson's disease, it has been reported that polymorphisms in the NFKB1 gene may affect the development of redox balance to prooxidation framework and may help regulate disease progression [42]. Among the nine genes selected using LASSO regression analysis, SOD2, NOX1 [11], and HMOX1 genes were also closely related to the regulation of redox in the body. NOX1 is involved in the synthesis of [43] nicotinamide adenine dinucleotide phosphate oxidases, while HMOX1 has been shown to inhibit the occurrence of OS. For MT1X, metallothionein has been documented as an antioxidant that protects cells from free radicals and OS generated by mutagens, antitumor drugs, and radiation [44]. As the most abundant nonprotein mercaptan, glutathione plays a key role in conferring protection against OS injury. Glutathione is also a key determinant of redox signaling. A key determinant of glutathione synthesis is the activity of the rate-limiting enzyme glutamate-cysteine ligase (GCL), which consists of a catalytic subunit (GCLC) and a modified subunit (GCLM) [45]. Therefore, GCLC is markedly associated with the regulation of OS. It was found that the pathways or molecules regulated by these nine selected genes played a key role in the balance regulation of OS, which laid the biochemical foundation for our model. After verifying the rationality of the model, we used a histogram to plot the survival prediction model for patients and finally used the TMB to confirm whether patients could benefit from immunization and targeted therapy against OS.

## 5. Conclusions

Throughout our experiments, we focused on the OS pathway, which led to the formulation of the first conclusion that OS played a bidirectional role in cancer, especially in KIRC. Three clusters representing gene expression of different OS pathway gene expression were used as research tools, and various analyses were conducted to confirm the previous findings. Finally, based on the OS pathway genes, a survival prediction model was constructed. Regulators used to build the risk signature may also become targets for the diagnosis and treatment of KIRC.

## Data Availability

The data supporting the findings of this study can be obtained from the corresponding authors.

## Abbreviations

KIRC: Kidney renal clear cell carcinoma
TCGA: The Cancer Genome Atlas
GEPIA: Gene Expression Profiling Interactive Analysis
GTEX: The Genotype-Tissue Expression
PPI: Protein-protein interaction
GDSC: Genomics of Drug Sensitivity in Cancer
HPA: Human Protein Atlas
LASSO: Least absolute shrinkage and selection operator
GSEA: Gene Set Enrichment Analysis
CNV: The copy number variation
SNV: Single-nucleotide variation
CTLA-4: Cytotoxic T lymphocyte-associated protein 4
PD-1: Programmed cell death protein 1
HDAC: Zinc-dependent histone deacetylases
SIRT: Sirtuins
KEGG: Kyoto Encyclopedia of Genes and Genomes
GO: Gene Ontology
TMB: Tumor mutation burden
SOD2: Superoxide dismutase-2
NOX1: NADPH Oxidase 1
HMOX1: Heme Oxygenase 1
CYBB: Cytochrome B-245 Beta Chain
MGST1: Microsomal Glutathione S-Transferase 1
TXNRD1: Thioredoxin reductase 1
UGT1A6: UDP Glucuronosyltransferase Family 1 Member A6
MAPK10: Mitogen-Activated Protein Kinase 10
MAOA: Monoamine Oxidase A
FOS: Fos proto-oncogene (p55)
CAT: Catalase
TXN2: Thioredoxin 2
TXNRD1: Thioredoxin Reductase 1
GCLC: Glutamate-Cysteine Ligase Catalytic Subunit
NFKB1: Nuclear factor kappa B subunit 1
MT1X: Metallothionein 1X.

## Supplementary Materials

Supplementary Materials Figure S1: the results of internal random sampling verification. Figure S2: the results of immunohistochemistry experiments on SOD2 and CAT. Figure S3: the heatmap shows the correlation between SOD2, CAT, and the macrophage marker CD68. Table S1: CNV amplification frequency of oxidative stress pathway genes across 32 cancer types. Table S2: CNV deletion frequency of oxidative stress pathway genes across 32 cancer types. Table S3: LogFCs of oxidative stress pathway genes across cancer types. Table S4: hazard ratio of 33 oxidative stress genes. Table S5: interactions of oxidative stress genes. Table S6: three levels of oxidative stress gene mRNA regulation. Table S7: clusters and OS-score information. Table S8: univariate Cox regression for hazard ratio of 32 oxidative stress genes. Table S9: LASSO regression coefficients of 9 selected genes. Table S10: information of GSEA in GO database. Table S11: information of GSEA in KEGG database. Table S12: information of univariate Cox regression analysis for the influencing factors of the prediction model. Table S13: information of multivariate Cox regression analysis for the influencing factors of the prediction model. Table S14: tumor mutation burden (TMB) value of each samples.
