# Supplementary material for: Using Genomic and Transcriptome Analyses to Identify the Role of the Oxidative Stress Pathway in Renal Clear Cell Carcinoma and Its Potential Therapeutic Significance
Source: Oxid Med Cell Longev. 2021 Oct 20;2021:5561124. doi: 10.1155/2021/5561124 (PMC8550864; doi:10.1155/2021/5561124)
Supplement: Supplementary Materials — Figure S1: the results of internal random sampling verification. Figure S2: the results of immunohistochemistry experiments on SOD2 and CAT. Figure S3: the heatmap shows the correlation between SOD2, CAT, and the macrophage marker CD68. Table S1: CNV amplification frequency of oxidative stress pathway genes across 32 cancer types. Table S2: CNV deletion frequency of oxidative stress pathway genes across 32 cancer types. Table S3: LogFCs of oxidative stress pathway genes across cancer types. Table S4: hazard ratio of 33 oxidative stress genes. Table S5: interactions of oxidative stress genes. Table S6: three levels of oxidative stress gene mRNA regulation. Table S7: clusters and OS-score information. Table S8: univariate Cox regression for hazard ratio of 32 oxidative stress genes. Table S9: LASSO regression coefficients of 9 selected genes. Table S10: information of GSEA in GO database. Table S11: information of GSEA in KEGG database. Table S12: information of univariate Cox regression analysis for the influencing factors of the prediction model. Table S13: information of multivariate Cox regression analysis for the influencing factors of the prediction model. Table S14: tumor mutation burden (TMB) value of each samples. [file 5561124.f1.pdf]

Supplementary Materials Figures and Legends

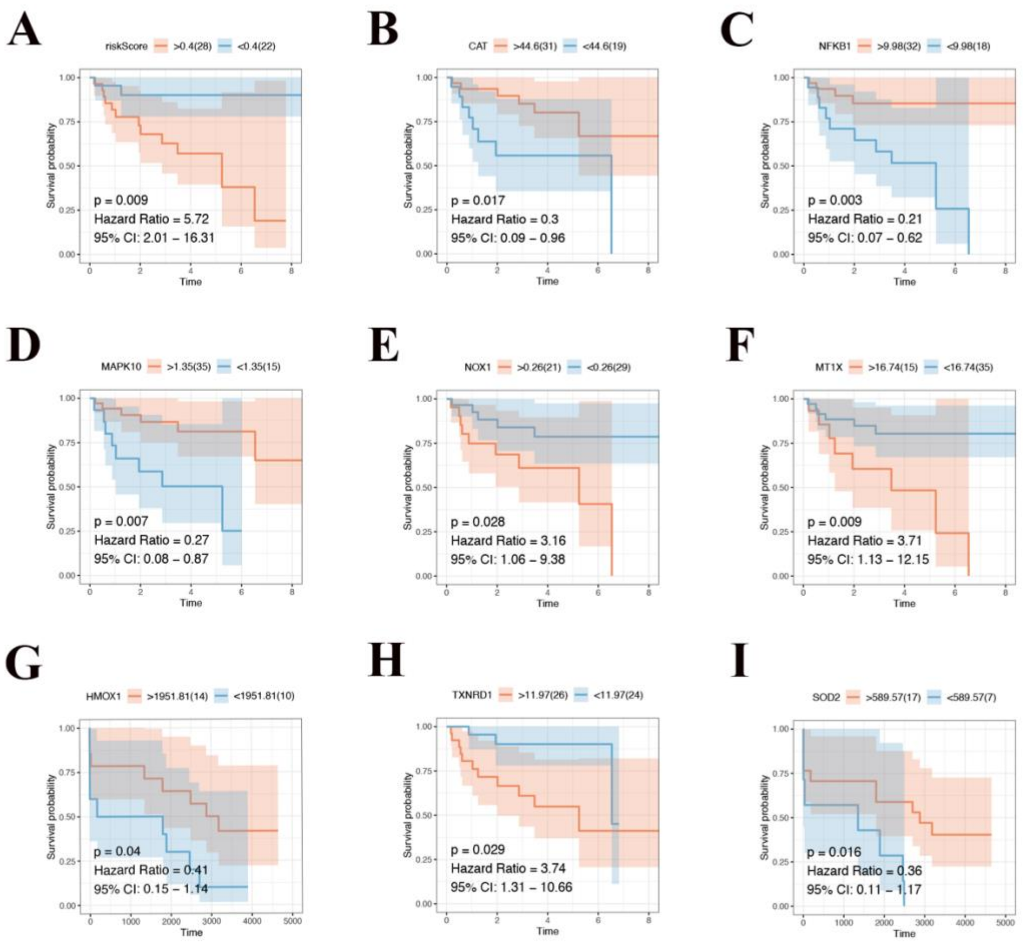

Figure S1: The results of internal random sampling verification. (A-I) RiskScore, CAT, NFKB1, MAPK10, NOX1, MT1X, HMOX1, TXNRD1 and SOD2.

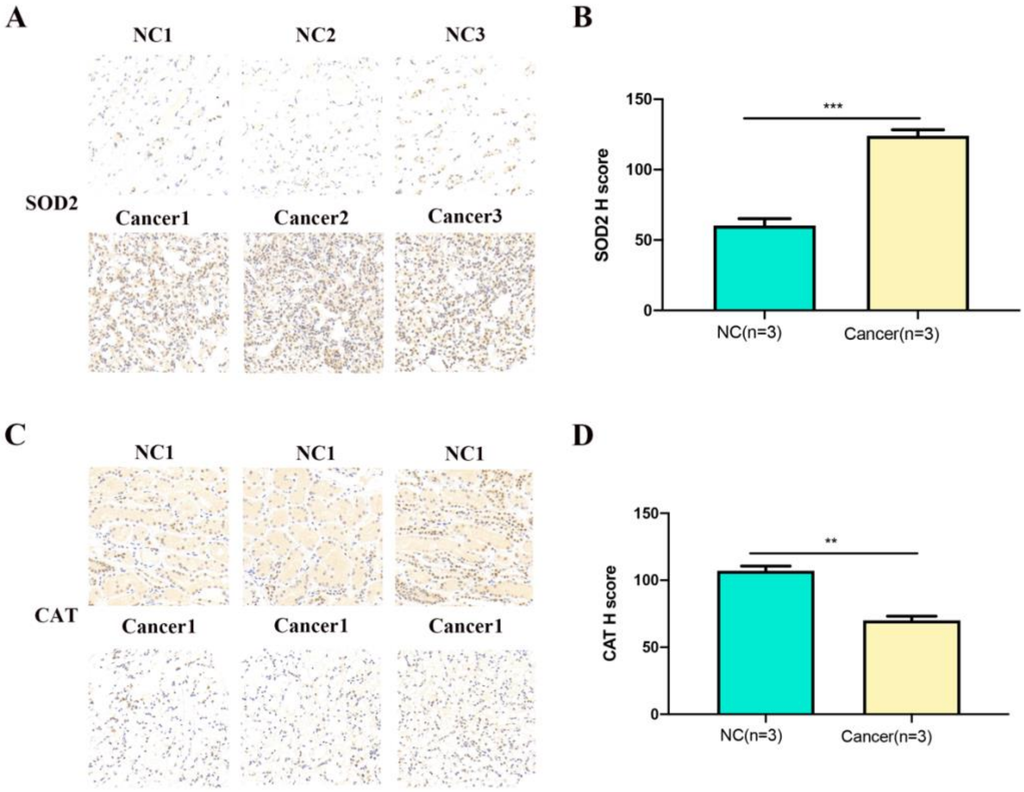

Figure S2: The results of immunohistochemistry experiments on SOD2 and CAT. (A-D) Typical immunohistochemical images of SOD2 and CAT in kidney cancer tissue and normal kidney tissue, and the corresponding semi-quantitative histogram. ***p <0.001.

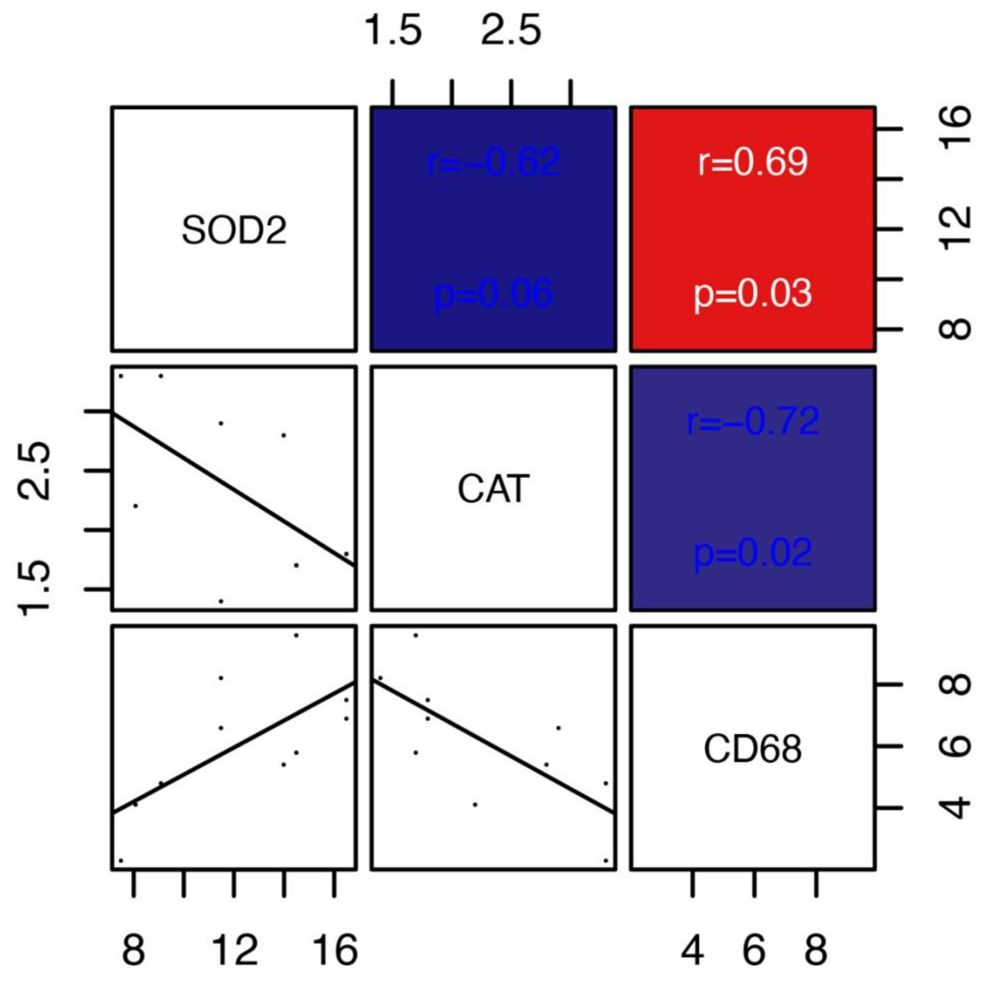

Figure S3: The heat map shows the correlation between SOD2, CAT and the macrophage marker CD68.

Table S1: CNV amplification frequency of oxidative stress pathway genes across 32 cancer types

	ACC	BLCA	BRCA	CESC	CHOL
CAT	0.011111111	0.043478261	0.102155576	0.032653061	0
CYBB	0.022222222	0.055555556	0.026241799	0.057142857	0
CYP1A1	0.066666667	0.028985507	0.038425492	0.106122449	0.03030303
FOS	0.155555556	0.038647343	0.026241799	0.102040816	0
GCLC	0.122222222	0.084541063	0.119962512	0.134693878	0.121212121
GPX1	0.022222222	0.060386473	0.010309278	0.008163265	0
GPX3	0.544444444	0.004830918	0.047797563	0.044897959	0.121212121
GSR	0.311111111	0.033816425	0.070290534	0.032653061	0.03030303
HMOX1	0.022222222	0.060386473	0.030927835	0.040816327	0
JUNB	0.477777778	0.02173913	0.049671978	0.048979592	0
MAOA	0.033333333	0.050724638	0.031865042	0.065306122	0
MAPK10	0.288888889	0.041062802	0.032802249	0.016326531	0
MAPK14	0.122222222	0.053140097	0.099343955	0.134693878	0.151515152
MGST1	0.611111111	0.089371981	0.085285848	0.057142857	0.090909091
MT1X	0.411111111	0.031400966	0.031865042	0.057142857	0
NFE2L2	0.033333333	0.041062802	0.030927835	0.048979592	0.090909091
NFIX	0.488888889	0.036231884	0.066541706	0.06122449	0.060606061
NFKB1	0.255555556	0.012077295	0.035613871	0.032653061	0
NOX1	0	0.026570048	0.0131209	0.020408163	0
NOX3	0.133333333	0.007246377	0.043111528	0.024489796	0.03030303
NOX4	0.022222222	0.074879227	0.053420806	0.06122449	0
NOX5	0.066666667	0.019323671	0.035613871	0.093877551	0.03030303
NQO1	0.4	0.082125604	0.029990628	0.073469388	0.03030303
SOD1	0.177777778	0.106280193	0.06185567	0.040816327	0
SOD2	0.122222222	0.009661836	0.044985942	0.024489796	0.03030303
SOD3	0.288888889	0.024154589	0.016869728	0.012244898	0.060606061
SP1	0.6	0.038647343	0.044048735	0.040816327	0.090909091
TXN2	0.011111111	0.04589372	0.029990628	0.032653061	0
TXNRD1	0.6	0.057971014	0.050609185	0.028571429	0.060606061
TXNRD2	0.022222222	0.043478261	0.026241799	0.040816327	0
UGT1A6	0.055555556	0.002415459	0.007497657	0	0.060606061
XDH	0.044444444	0.120772947	0.057169634	0.130612245	0.03030303

COAD	DLBC	ESCA	GBM	HNSC	KICH
0.028056112	0.068965517	0.177083333	0.012738854	0.060344828	0.287878788
0.014028056	0.034482759	0.020833333	0.00477707	0	0
0.008016032	0.034482759	0.052083333	0.01433121	0.025862069	0.409090909
0.008016032	0.068965517	0.135416667	0.011146497	0.077586207	0.46969697
0.056112224	0.103448276	0.083333333	0.01433121	0.112068966	0.03030303
0.008016032	0.137931034	0	0.038216561	0	0.303030303
0.01002004	0.068965517	0	0.01433121	0	0.333333333
0.100200401	0.034482759	0.104166667	0.02388535	0.051724138	0.196969697
0.012024048	0	0.0625	0.00477707	0.068965517	0.242424242
0.03006012	0	0.052083333	0.281847134	0.051724138	0.318181818
0.012024048	0.034482759	0.03125	0.003184713	0.017241379	0
0.008016032	0.034482759	0.041666667	0.022292994	0.00862069	0.424242424
0.062124248	0.103448276	0.03125	0.00955414	0.043103448	0.03030303
0.126252505	0.034482759	0.239583333	0.070063694	0.189655172	0.318181818
0.086172345	0.034482759	0.09375	0.017515924	0.034482759	0.318181818
0.022044088	0	0.229166667	0.012738854	0.120689655	0.060606061
0.034068136	0	0.083333333	0.28343949	0.043103448	0.333333333
0.008016032	0	0.03125	0.017515924	0	0.439393939
0.014028056	0.034482759	0.114583333	0.006369427	0.025862069	0
0.04008016	0.034482759	0.041666667	0.01433121	0.025862069	0.03030303
0.018036072	0.103448276	0.125	0.020700637	0.043103448	0.242424242
0.008016032	0.034482759	0.041666667	0.011146497	0.025862069	0.454545455
0.082164329	0.034482759	0.09375	0.012738854	0.051724138	0.272727273
0.02004008	0.068965517	0.041666667	0.050955414	0.00862069	0.090909091
0.036072144	0.068965517	0.041666667	0.011146497	0.025862069	0.03030303
0.006012024	0	0.03125	0.02388535	0.00862069	0.46969697
0.094188377	0.034482759	0.020833333	0.047770701	0.017241379	0.46969697
0.016032064	0	0.0625	0.006369427	0.077586207	0.378787879
0.070140281	0.103448276	0.052083333	0.036624204	0.025862069	0.5
0.006012024	0	0.0625	0.012738854	0.077586207	0.257575758
0.028056112	0	0.010416667	0.012738854	0.017241379	0.015151515
0.022044088	0.034482759	0.135416667	0.025477707	0.060344828	0.045454545

KIRC	KIRP	LGG	LIHC	LUAD	LUSC
0.01188455	0.026315789	0.024390244	0.023746702	0.063706564	0.053435115
0.016977929	0.003289474	0.015009381	0.005277045	0.021235521	0.030534351
0.005093379	0.006578947	0.015009381	0.042216359	0.028957529	0.041984733
0.008488964	0.003289474	0.003752345	0.021108179	0.061776062	0.061068702
0.005093379	0.023026316	0.009380863	0.221635884	0.108108108	0.124045802
0.008488964	0.131578947	0.005628518	0.039577836	0.001930502	0
0.341256367	0.082236842	0.009380863	0.163588391	0.040540541	0.003816794
0.001697793	0.055921053	0.039399625	0.021108179	0.028957529	0.030534351
0.01188455	0.009868421	0.020637899	0.052770449	0.027027027	0.158396947
0.016977929	0.013157895	0.052532833	0.029023747	0.003861004	0.066793893
0.015280136	0.003289474	0.011257036	0.021108179	0.040540541	0.036259542
0.006791171	0.009868421	0.00750469	0.013192612	0.00965251	0.028625954
0.005093379	0.009868421	0.015009381	0.245382586	0.127413127	0.045801527
0.045840407	0.325657895	0.045028143	0.023746702	0.086872587	0.188931298
0.037351443	0.378289474	0.015009381	0.013192612	0.055984556	0.036259542
0.052631579	0.065789474	0.003752345	0.07651715	0.065637066	0.124045802
0.016977929	0.023026316	0.067542214	0.047493404	0.015444015	0.064885496
0.005093379	0.013157895	0.005628518	0.01055409	0.005791506	0.011450382
0.008488964	0.006578947	0.011257036	0.055408971	0.034749035	0.040076336
0.001697793	0.006578947	0	0.034300792	0.007722008	0.051526718
0.01188455	0.029605263	0.05065666	0.029023747	0.088803089	0.06870229
0.008488964	0.006578947	0.015009381	0.029023747	0.023166023	0.051526718
0.040747029	0.358552632	0.013133208	0.01055409	0.067567568	0.028625954
0.030560272	0.042763158	0.020637899	0.023746702	0.046332046	0.028625954
0.001697793	0.009868421	0	0.036939314	0.007722008	0.049618321
0.006791171	0.006578947	0.013133208	0.044854881	0.05019305	0.011450382
0.0475382	0.282894737	0.003752345	0.034300792	0.090733591	0.049618321
0.01188455	0.009868421	0.015009381	0.052770449	0.025096525	0.15648855
0.045840407	0.309210526	0.011257036	0.034300792	0.048262548	0.026717557
0.013582343	0.009868421	0.013133208	0.026385224	0.032818533	0.154580153
0.03565365	0.046052632	0.003752345	0.029023747	0.032818533	0.009541985
0.018675722	0.046052632	0.00750469	0.044854881	0.065637066	0.185114504

MESO	OV	PAAD	PCPG	PRAD	READ
0.011627907	0.12718601	0.020134228	0.02	0.00811359	0.012048193
0	0.06200318	0.013422819	0.006666667	0.012170385	0.084337349
0.11627907	0.085850556	0.026845638	0.046666667	0.002028398	0.012048193
0.011627907	0.082670906	0.020134228	0.02	0.006085193	0.012048193
0.034883721	0.209856916	0.006711409	0.066666667	0.004056795	0.144578313
0.034883721	0.100158983	0	0.02	0.006085193	0.024096386
0.069767442	0.112877583	0.013422819	0.026666667	0.010141988	0.060240964
0.058139535	0.058823529	0.020134228	0.033333333	0.024340771	0.036144578
0.011627907	0.060413355	0.006711409	0	0	0.024096386
0.081395349	0.310015898	0	0.06	0.004056795	0.048192771
0	0.101748808	0.013422819	0.006666667	0.006085193	0.048192771
0	0.036565978	0	0.026666667	0.010141988	0.012048193
0.058139535	0.302066773	0.006711409	0.066666667	0.006085193	0.072289157
0.034883721	0.383147854	0.046979866	0.026666667	0.010141988	0.13253012
0.034883721	0.050874404	0.013422819	0.02	0.006085193	0.084337349
0.023255814	0.262321145	0.020134228	0.006666667	0.002028398	0.048192771
0.093023256	0.362480127	0.013422819	0.073333333	0.016227181	0.048192771
0	0.020667727	0	0.026666667	0.006085193	0.024096386
0	0.101748808	0.006711409	0	0	0
0	0.057233704	0	0.013333333	0.006085193	0.048192771
0.046511628	0.282988871	0.020134228	0.006666667	0.014198783	0.024096386
0.127906977	0.108108108	0.026845638	0.053333333	0.004056795	0.012048193
0.023255814	0.028616852	0	0.02	0.004056795	0.108433735
0.034883721	0.122416534	0.006711409	0	0.012170385	0.048192771
0	0.069952305	0	0.013333333	0	0.048192771
0.011627907	0.092209857	0	0.013333333	0.002028398	0.060240964
0.023255814	0.189189189	0	0.04	0.010141988	0.072289157
0.011627907	0.039745628	0.006711409	0	0.004056795	0.024096386
0.069767442	0.124006359	0	0.046666667	0.022312373	0.060240964
0.023255814	0.063593005	0	0	0.002028398	0.024096386
0.011627907	0.155802862	0.033557047	0.006666667	0.002028398	0.036144578
0.023255814	0.262321145	0.006711409	0.026666667	0.006085193	0.024096386

SARC	SKCM	STAD	TGCT	THCA	THYM
0.066666667	0.031779661	0.063348416	0.012820513	0.003913894	0
0.308333333	0.023305085	0.020361991	0.012820513	0	0
0.15	0.091101695	0.036199095	0.038461538	0	0.033333333
0.175	0.027542373	0.015837104	0.08974359	0.001956947	0
0.2	0.252118644	0.085972851	0.044871795	0.001956947	0
0.066666667	0.016949153	0.009049774	0.025641026	0	0
0.125	0.033898305	0.013574661	0	0.019569472	0
0.133333333	0.091101695	0.113122172	0.326923077	0	0
0.175	0.144067797	0.015837104	0.044871795	0	0
0.225	0.023305085	0.011312217	0.019230769	0	0
0.308333333	0.021186441	0.018099548	0.012820513	0	0
0.1	0.036016949	0.00678733	0	0.001956947	0
0.075	0.31779661	0.061085973	0.032051282	0.001956947	0
0.15	0.086864407	0.070135747	0.897435897	0.007827789	0.033333333
0.025	0.021186441	0.009049774	0.032051282	0.005870841	0
0.066666667	0.027542373	0.031674208	0.044871795	0.001956947	0
0.216666667	0.040254237	0.031674208	0.057692308	0.007827789	0
0.108333333	0.029661017	0.00678733	0	0.001956947	0
0.033333333	0.008474576	0.027149321	0.012820513	0.001956947	0
0.15	0.014830508	0.054298643	0.012820513	0	0
0.091666667	0.040254237	0.042986425	0.006410256	0	0
0.2	0.086864407	0.038461538	0.038461538	0	0.033333333
0.025	0.031779661	0.022624434	0.019230769	0.005870841	0
0.158333333	0.050847458	0.00678733	0.532051282	0	0.033333333
0.116666667	0.016949153	0.049773756	0.012820513	0	0
0.208333333	0.069915254	0.020361991	0.006410256	0.001956947	0
0.025	0.014830508	0.033936652	0.16025641	0.009784736	0
0.183333333	0.131355932	0.013574661	0.044871795	0	0
0.066666667	0.014830508	0.020361991	0.102564103	0.009784736	0
0.183333333	0.129237288	0.022624434	0.102564103	0.003913894	0.033333333
0.041666667	0.012711864	0.011312217	0.006410256	0.001956947	0
0.05	0.033898305	0.027149321	0.134615385	0	0.033333333

UCEC	UCS	UVM
0.027322404	0.017857143	0.0625
0.029143898	0.178571429	0.0125
0.018214936	0.107142857	0.0125
0.041894353	0.071428571	0.0625
0.058287796	0.357142857	0.3375
0.043715847	0.017857143	0
0.023679417	0.053571429	0
0.114754098	0.196428571	0.2
0.036429872	0.178571429	0.0125
0.120218579	0.142857143	0.0125
0.032786885	0.196428571	0.025
0.014571949	0	0.05
0.071038251	0.357142857	0.55
0.054644809	0.214285714	0
0.021857923	0.035714286	0
0.080145719	0.196428571	0.05
0.132969035	0.089285714	0.0375
0.007285974	0	0.05
0.014571949	0.053571429	0
0.04007286	0.196428571	0.05
0.04007286	0.071428571	0.075
0.009107468	0.089285714	0.0125
0.016393443	0	0
0.030965392	0.160714286	0.1875
0.038251366	0.142857143	0.0625
0.023679417	0.178571429	0.1375
0.06557377	0.196428571	0
0.038251366	0.178571429	0.0125
0.032786885	0.071428571	0
0.025500911	0.142857143	0.0125
0.049180328	0.160714286	0.025
0.109289617	0.392857143	0.125

Table S2: CNV deletion frequency of oxidative stress pathway genes across 32 cancer types

	ACC	BLCA	BRCA	CESC	CHOL
CAT	0.177777778	0.142512077	0.049671978	0.118367347	0.060606061
CYBB	0	0.028985507	0.018744142	0.024489796	0
CYP1A1	0.1	0.041062802	0.05529522	0.036734694	0
FOS	0.066666667	0.077294686	0.112464855	0.020408163	0.363636364
GCLC	0.077777778	0.031400966	0.035613871	0.089795918	0.090909091
GPX1	0.1	0.089371981	0.128397376	0.310204082	0.515151515
GPX3	0.022222222	0.125603865	0.063730084	0.089795918	0
GSR	0.122222222	0.292270531	0.287722587	0.191836735	0.121212121
HMOX1	0.311111111	0.060386473	0.187441425	0.053061224	0.03030303
JUNB	0.011111111	0.048309179	0.029053421	0.065306122	0
MAOA	0	0.038647343	0.017806935	0.032653061	0
MAPK10	0.033333333	0.06763285	0.070290534	0.142857143	0.151515152
MAPK14	0.077777778	0.036231884	0.033739456	0.06122449	0.060606061
MGST1	0.033333333	0.038647343	0.070290534	0.085714286	0.03030303
MT1X	0.055555556	0.089371981	0.305529522	0.089795918	0.060606061
NFE2L2	0.055555556	0.074879227	0.029990628	0.044897959	0
NFIX	0.011111111	0.04589372	0.026241799	0.065306122	0
NFKB1	0.033333333	0.041062802	0.063730084	0.102040816	0.181818182
NOX1	0.022222222	0.014492754	0.010309278	0	0
NOX3	0.1	0.111111111	0.138706654	0.167346939	0.454545455
NOX4	0.177777778	0.074879227	0.209934396	0.224489796	0.060606061
NOX5	0.1	0.04589372	0.057169634	0.040816327	0
NQO1	0.044444444	0.072463768	0.319587629	0.057142857	0.090909091
SOD1	0.1	0.028985507	0.040299906	0.053061224	0.181818182
SOD2	0.1	0.115942029	0.149015933	0.175510204	0.454545455
SOD3	0.044444444	0.06763285	0.11715089	0.281632653	0.060606061
SP1	0	0.026570048	0.029053421	0.004081633	0
TXN2	0.311111111	0.065217391	0.194001874	0.069387755	0.03030303
TXNRD1	0.022222222	0.026570048	0.046860356	0.004081633	0.121212121
TXNRD2	0.311111111	0.055555556	0.159325211	0.036734694	0.03030303
UGT1A6	0.077777778	0.214975845	0.059981256	0.281632653	0
XDH	0.077777778	0.012077295	0.018744142	0.004081633	0.03030303

COAD	DLBC	ESCA	GBM	HNSC	KICH
0.014028056	0	0.052083333	0.068471338	0.060344828	0.106060606
0.026052104	0	0.114583333	0.00955414	0.068965517	0
0.140280561	0.068965517	0.03125	0.076433121	0.051724138	0
0.190380762	0	0.052083333	0.203821656	0.051724138	0.015151515
0.022044088	0	0.03125	0.039808917	0.051724138	0.681818182
0.044088176	0.034482759	0.395833333	0.042993631	0.284482759	0.045454545
0.04008016	0	0.166666667	0.022292994	0.103448276	0.090909091
0.308617234	0.172413793	0.28125	0.042993631	0.103448276	0.166666667
0.114228457	0	0.03125	0.210191083	0.00862069	0.015151515
0.016032064	0	0.010416667	0.007961783	0.00862069	0
0.026052104	0	0.104166667	0.012738854	0.060344828	0
0.066132265	0.103448276	0.072916667	0.025477707	0.051724138	0
0.016032064	0	0.041666667	0.031847134	0.068965517	0.545454545
0.018036072	0.034482759	0.010416667	0.060509554	0.017241379	0.015151515
0.016032064	0.034482759	0.03125	0.065286624	0.077586207	0.045454545
0.01002004	0	0.020833333	0.003184713	0.00862069	0.333333333
0.016032064	0	0.010416667	0.01433121	0.00862069	0
0.062124248	0.068965517	0.114583333	0.027070064	0.094827586	0
0.004008016	0	0	0	0	0
0.034068136	0.137931034	0.072916667	0.164012739	0.034482759	0.666666667
0.026052104	0	0.166666667	0.025477707	0.094827586	0.121212121
0.134268537	0.068965517	0.03125	0.070063694	0.043103448	0
0.014028056	0.034482759	0.03125	0.068471338	0.077586207	0.045454545
0.098196393	0	0.166666667	0.031847134	0.086206897	0.318181818
0.036072144	0.137931034	0.104166667	0.167197452	0.043103448	0.666666667
0.078156313	0	0.239583333	0.02388535	0.163793103	0
0	0	0.03125	0.046178344	0	0
0.110220441	0	0.03125	0.207006369	0.00862069	0
0.018036072	0.034482759	0.020833333	0.01910828	0.00862069	0
0.112224449	0	0.03125	0.175159236	0.017241379	0.015151515
0.026052104	0	0.125	0.02866242	0.103448276	0.363636364
0.012024048	0	0	0.007961783	0	0.409090909

KIRC	KIRP	LGG	LIHC	LUAD	LUSC
0.005093379	0.026315789	0.088180113	0.015831135	0.027027027	0.026717557
0.003395586	0.006578947	0.018761726	0.073878628	0.036679537	0.036259542
0.008488964	0.029605263	0.026266417	0.055408971	0.036679537	0.020992366
0.132427844	0.101973684	0.135084428	0.155672823	0.038610039	0.047709924
0.042444822	0.072368421	0.015009381	0.01055409	0.023166023	0.019083969
0.42614601	0.046052632	0.041275797	0.052770449	0.063706564	0.183206107
0.001697793	0.009868421	0.030018762	0.015831135	0.042471042	0.146946565
0.108658744	0.0625	0.015009381	0.548812665	0.21042471	0.190839695
0.005093379	0.121710526	0.054409006	0.07651715	0.063706564	0.013358779
0.003395586	0.016447368	0.003752345	0.07651715	0.106177606	0.047709924
0.001697793	0.009868421	0.015009381	0.065963061	0.025096525	0.041984733
0.016977929	0.049342105	0.060037523	0.250659631	0.030888031	0.064885496
0.033955857	0.082236842	0.005628518	0.018469657	0.011583012	0.036259542
0.001697793	0.003289474	0.020637899	0.092348285	0.065637066	0.003816794
0.013582343	0.006578947	0.013133208	0.224274406	0.052123552	0.070610687
0.005093379	0.006578947	0.009380863	0.023746702	0	0.007633588
0.003395586	0.016447368	0.003752345	0.07651715	0.106177606	0.055343511
0.015280136	0.046052632	0.061913696	0.250659631	0.019305019	0.06870229
0.003395586	0.003289474	0.003752345	0.002638522	0.003861004	0.009541985
0.101867572	0.059210526	0.088180113	0.229551451	0.135135135	0.028625954
0.01188455	0.032894737	0.009380863	0.065963061	0.021235521	0.02480916
0.005093379	0.023026316	0.024390244	0.036939314	0.042471042	0.022900763
0.01188455	0.006578947	0.011257036	0.237467018	0.030888031	0.066793893
0.033955857	0.105263158	0.028142589	0.118733509	0.061776062	0.078244275
0.101867572	0.059210526	0.088180113	0.232189974	0.142857143	0.038167939
0.018675722	0.046052632	0.031894934	0.094986807	0.019305019	0.145038168
0	0	0.04315197	0.007915567	0.00965251	0.003816794
0.005093379	0.121710526	0.046904315	0.071240106	0.071428571	0.015267176
0.001697793	0	0.033771107	0.023746702	0.019305019	0.009541985
0.003395586	0.118421053	0.033771107	0.073878628	0.046332046	0.019083969
0.049235993	0.046052632	0.071294559	0.034300792	0.015444015	0.066793893
0.010186757	0.029605263	0.011257036	0.005277045	0.001930502	0.003816794

MESO	OV	PAAD	PCPG	PRAD	READ
0.011627907	0.13990461	0.013422819	0.24	0.014198783	0.036144578
0.011627907	0.141494436	0.013422819	0	0.004056795	0
0.011627907	0.246422893	0.026845638	0.006666667	0.022312373	0.168674699
0.186046512	0.211446741	0.020134228	0.073333333	0.016227181	0.192771084
0.034883721	0.101748808	0.020134228	0	0.016227181	0.024096386
0.290697674	0.128775835	0.033557047	0.18	0.014198783	0.096385542
0.034883721	0.186009539	0.013422819	0.033333333	0.010141988	0.144578313
0.093023256	0.446740859	0.073825503	0.126666667	0.269776876	0.385542169
0.476744186	0.46263911	0.040268456	0.3	0.012170385	0.144578313
0	0.100158983	0.013422819	0	0.010141988	0.012048193
0.011627907	0.101748808	0.026845638	0	0.006085193	0.024096386
0.127906977	0.422893482	0.013422819	0.04	0.022312373	0.108433735
0.011627907	0.093799682	0.020134228	0	0.004056795	0.072289157
0.046511628	0.092209857	0.040268456	0.08	0.0831643	0.096385542
0.011627907	0.432432432	0.006711409	0	0.107505071	0.012048193
0	0.028616852	0.013422819	0.013333333	0.036511156	0.024096386
0.011627907	0.074721781	0.006711409	0	0.012170385	0.012048193
0.11627907	0.473767886	0.013422819	0	0.00811359	0.156626506
0	0.027027027	0	0	0	0.024096386
0.197674419	0.434022258	0.120805369	0.14	0.024340771	0.048192771
0.034883721	0.089030207	0	0.173333333	0.006085193	0.036144578
0.011627907	0.217806041	0.026845638	0.013333333	0.014198783	0.180722892
0.046511628	0.513513514	0.006711409	0.006666667	0.154158215	0.012048193
0.034883721	0.158982512	0.053691275	0.133333333	0.020283976	0.180722892
0.174418605	0.448330684	0.120805369	0.133333333	0.032454361	0.048192771
0.139534884	0.246422893	0.006711409	0.02	0.026369168	0.072289157
0.023255814	0.06518283	0.013422819	0.013333333	0.004056795	0.012048193
0.476744186	0.483306836	0.040268456	0.3	0.010141988	0.144578313
0.011627907	0.193958665	0.013422819	0.033333333	0.014198783	0.048192771
0.38372093	0.33227345	0.053691275	0.213333333	0.014198783	0.144578313
0.034883721	0.120826709	0.006711409	0.02	0.016227181	0
0.069767442	0.027027027	0.006711409	0.033333333	0.018255578	0.048192771

SARC	SKCM	STAD	TGCT	THCA	THYM
0.108333333	0.112288136	0.013574661	0.057692308	0.005870841	0.033333333
0	0.014830508	0.011312217	0.012820513	0.001956947	0
0.05	0.023305085	0.029411765	0.006410256	0.011741683	0
0.058333333	0.131355932	0.022624434	0	0.005870841	0.033333333
0.041666667	0.046610169	0.009049774	0.012820513	0.001956947	0.033333333
0.125	0.065677966	0.042986425	0	0	0.033333333
0.041666667	0.152542373	0.056561086	0.032051282	0	0
0.133333333	0.137711864	0.083710407	0.006410256	0	0
0.05	0.063559322	0.056561086	0.019230769	0.140900196	0.033333333
0.025	0.033898305	0.042986425	0.012820513	0.011741683	0
0	0.014830508	0.011312217	0.012820513	0.001956947	0
0.083333333	0.065677966	0.056561086	0.038461538	0.001956947	0
0.033333333	0.025423729	0.024886878	0.006410256	0	0.033333333
0.133333333	0.040254237	0.042986425	0	0	0
0.191666667	0.133474576	0.049773756	0.019230769	0.005870841	0.066666667
0.05	0.029661017	0.013574661	0	0.001956947	0
0.008333333	0.040254237	0.033936652	0.012820513	0.01369863	0
0.091666667	0.072033898	0.033936652	0.044871795	0	0
0.116666667	0.008474576	0.00678733	0	0.001956947	0
0.05	0.338983051	0.038461538	0.012820513	0.009784736	0.066666667
0.125	0.184322034	0.011312217	0.173076923	0.01369863	0
0.041666667	0.023305085	0.027149321	0.019230769	0.011741683	0
0.2	0.116525424	0.040723982	0	0.005870841	0.066666667
0.058333333	0.063559322	0.074660633	0	0.001956947	0.033333333
0.091666667	0.334745763	0.036199095	0.012820513	0.005870841	0.066666667
0.05	0.061440678	0.040723982	0.012820513	0	0
0.075	0.078389831	0.024886878	0	0	0
0.05	0.06779661	0.056561086	0.019230769	0.140900196	0.066666667
0.016666667	0.091101695	0.024886878	0	0	0
0.05	0.046610169	0.036199095	0.006410256	0.119373777	0
0.25	0.082627119	0.024886878	0.006410256	0.007827789	0
0.1	0.046610169	0.015837104	0	0.007827789	0

UCEC	UCS	UVM
0.063752277	0.25	0.0125
0.04007286	0.107142857	0
0.080145719	0.232142857	0.05
0.038251366	0.178571429	0.025
0.016393443	0.017857143	0
0.052823315	0.339285714	0.5125
0.051001821	0.071428571	0.0625
0.14571949	0.339285714	0.225
0.102003643	0.357142857	0
0.04007286	0.285714286	0.0125
0.025500911	0.107142857	0
0.056466302	0.357142857	0.0625
0.007285974	0.035714286	0
0.032786885	0.142857143	0.0125
0.176684882	0.410714286	0.25
0.005464481	0	0
0.029143898	0.303571429	0.0125
0.069216758	0.375	0.0625
0.001821494	0.035714286	0.0125
0.025500911	0.089285714	0.3625
0.061930783	0.285714286	0.0125
0.092896175	0.232142857	0.05
0.193078324	0.375	0.2625
0.02003643	0.142857143	0.0125
0.030965392	0.089285714	0.3375
0.078324226	0.053571429	0
0.007285974	0.053571429	0.05
0.103825137	0.357142857	0
0.023679417	0.178571429	0.025
0.061930783	0.25	0
0.021857923	0.017857143	0.05
0.003642987	0	0

Table S3: LogFCs of oxidative stress pathway genes across cancer types

	BLCA	CHOL	COAD	ESCA	KICH
NFKB1	-0.439076225	1.329462396	-0.130029002	NA	-0.306714868
TXN2	NA	0.410249505	-0.148117699	NA	NA
SOD2	-0.716605932	NA	NA	0.668002925	-0.705701405
NOX4	0.853513976	4.605482675	3.263303752	2.817397978	NA
SOD3	-1.66235745	2.459778774	NA	NA	-3.613988059
UGT1A6	NA	0.131464889	1.710398948	NA	-1.498557151
XDH	0.992387356	-2.101025185	-2.187278835	1.448922633	-0.660752536
MAOA	-0.320697309	-0.317039197	-1.865149825	-0.974378367	-1.150010817
SP1	NA	2.206209099	NA	0.443510943	-0.478850403
GSTT2	NA	NA	NA	NA	-2.700696949
NFIX	-1.903075542	1.810023147	NA	-0.65407519	NA
GPX1	0.775961434	NA	0.804836749	0.603481312	0.515302155
JUNB	-1.568824269	NA	NA	NA	-1.97243578
MT1X	NA	-3.89169703	-2.356448686	-0.842418616	-3.099756385
MAPK10	-0.542129104	2.790690524	-1.801331162	NA	-2.311669045
NOX1	0.793480721	2.410529043	0.989596271	3.867621588	-0.361940536
CAT	-0.547521273	-2.67514698	-0.75164504	-0.574691117	-0.274220454
NOX5	0.995531155	5.411323583	-0.321129384	NA	-0.276874703
NQO1	0.658532332	6.237128844	1.052144103	NA	-1.450788816
CYBB	NA	1.513610402	-0.579851189	0.850803195	NA
FOS	-2.937240001	-0.971530023	-0.719953527	NA	-3.093925318
MAPK14	NA	0.784720038	NA	0.301408918	-1.085250937
TXNRD1	-0.866484362	1.804494111	NA	NA	-0.203232
GCLC	NA	-1.511763315	0.356831363	NA	0.66287402
TXNRD2	NA	-0.434155129	0.326994165	NA	0.531490921
SOD1	0.314028363	-1.566449041	NA	0.552337549	-0.925362982
MGST1	0.583473919	-2.076407085	-0.470610763	NA	-3.197380429
NFE2L2	-0.710630109	0.542191221	-0.192206867	-0.24189644	-0.414667511
CYP1A1	2.893471597	-4.669465444	1.620690566	-1.543078178	NA
GPX3	-0.81076426	-1.06529925	-2.3223113	-1.296400651	-2.284920971
GSR	NA	1.997840241	-0.261042732	NA	NA
HMOX1	1.224009642	NA	-1.6121345	NA	NA
NOX3	NA	NA	0.727071377	NA	-3.646518219

KIRC	KIRP	LIHC	LUAD	LUSC
0.24164608	NA	NA	NA	-0.453567795
-0.406160513	-0.289301317	0.256332069	NA	0.576993718
1.188816991	1.089821731	NA	-0.214679891	-0.67744235
-1.248573196	-1.49807705	3.48709578	1.466367641	1.440239826
-1.554854137	-0.446983446	NA	-0.95238701	-2.231188392
NA	NA	1.292981041	NA	5.765389784
1.57384433	3.183194956	-0.715127622	4.258858745	3.689520568
-0.774355542	-0.520528385	NA	-0.677017714	-1.132004821
0.25255757	0.639147616	0.72266349	NA	0.220263438
NA	-1.295413759	1.415878012	NA	NA
0.791372509	-0.220984928	0.834115225	-1.305225757	-1.241695982
0.786001703	1.685195385	0.840856729	NA	-0.320157599
NA	-1.055455372	-0.885905773	-1.01761567	-0.98808724
-0.939859158	-1.92840954	-2.043501427	-0.705279145	0.472979029
-1.046656228	-0.721211511	0.682551663	-0.859758374	0.010759772
-0.040881999	0.646698896	1.505124846	1.538775552	0.854856431
-1.274063043	-1.475989999	-0.807725504	-1.769115259	-1.972019478
0.756442146	-0.385581267	2.065800921	3.353592348	1.877260641
-0.424650319	NA	5.311194426	3.109239193	2.864582221
1.847374247	1.139740871	-0.196676902	-1.142075491	-1.926140364
-0.669584286	-1.541522051	-2.419249865	-1.495271499	-2.306173777
-0.146114969	NA	0.55436025	0.232890743	NA
0.02024632	1.551702575	2.055144716	1.716596196	1.542206242
NA	0.653778999	NA	2.388910968	3.518187265
-0.552492445	-0.413656735	NA	NA	-0.201533103
-0.451491491	-0.196548791	-0.06641154	0.611578087	0.748441236
0.578077104	0.939754416	-0.279093959	1.11877363	NA
-0.484936515	-0.12983954	-0.242815962	-0.373418731	0.998364232
-3.077547011	-3.931159158	0.944241425	-1.605427172	0.440083849
-1.560492481	-3.310815649	NA	-2.178480585	-3.312563773
-0.595887164	0.013503365	0.766254577	1.100731372	1.500504426
2.368126439	1.665403533	-0.725030232	-1.540536423	-1.135785012
0.985761138	-1.339663883	NA	-0.661500652	-0.112726279

PAAD	PCPG	PRAD	READ	SARC	SKCM
NA	-0.953182582	-0.15175299	NA	NA	NA
NA	-1.465808136	0.156747759	NA	NA	NA
-1.604856297	-1.095318861	NA	NA	NA	NA
NA	3.437971067	1.986625421	NA	-4.478358278	NA
NA	NA	-0.116434162	NA	NA	NA
NA	NA	-1.501360096	NA	-12.53357508	NA
NA	NA	NA	NA	NA	NA
NA	-1.546357692	0.625737683	-1.773576706	-3.823678923	NA
NA	-0.51880438	-0.147515129	NA	NA	NA
NA	NA	NA	NA	NA	NA
NA	NA	0.268839386	NA	3.272214622	NA
NA	-1.202369904	0.25486236	NA	NA	NA
NA	NA	NA	NA	NA	NA
NA	-1.750808849	-0.384497846	-2.715786621	-2.941219384	NA
NA	NA	-0.214905248	-2.554996071	NA	NA
NA	NA	-1.349872267	NA	NA	NA
NA	-3.118101831	-0.538722872	-0.787089763	-2.248726044	NA
NA	NA	-0.169878904	NA	NA	NA
NA	-1.532247417	NA	NA	-2.320497119	NA
NA	NA	NA	NA	NA	NA
NA	NA	-0.55125022	NA	NA	NA
NA	NA	-0.35667376	NA	NA	NA
NA	-1.189004741	-0.219151072	NA	NA	NA
NA	NA	-0.301769818	NA	NA	NA
NA	-1.832598302	0.712070896	NA	NA	NA
NA	-0.922354154	0.236613386	NA	NA	NA
NA	-4.639694965	NA	NA	-2.023831365	NA
NA	NA	-0.589800114	NA	NA	NA
NA	NA	NA	NA	NA	NA
NA	NA	-1.08113055	-2.538000845	-6.186181838	NA
NA	NA	-0.231245734	NA	-1.606165349	NA
-1.484289735	NA	NA	NA	NA	NA
NA	3.541495755	NA	-1.991942632	NA	NA

STAD	THCA	THYM	UCEC
0.457611872	-0.180483607	NA	-0.77495831
-0.360722294	-0.247654534	0.604215647	0.305924106
0.95742894	-0.056513265	NA	NA
2.11586986	2.441944531	NA	NA
-0.867017165	-1.508258699	NA	-0.958881609
0.36053314	-0.444041865	NA	6.386840579
NA	NA	NA	3.989951705
-1.277094855	NA	NA	-1.431011042
0.559678602	NA	NA	-0.420211986
NA	-0.238788552	NA	NA
-0.595283266	-0.168840506	NA	-1.116725799
NA	0.980552896	NA	1.381529882
-1.119948165	-0.613828374	NA	-1.848197165
-1.551657923	-0.873335199	NA	NA
-1.490683086	-0.390604398	NA	-2.481330046
4.713549551	0.863214636	NA	NA
-0.456557157	NA	NA	-0.348583703
0.0573108	2.459236634	NA	NA
NA	NA	NA	3.137949585
0.992911752	0.08335611	NA	NA
-1.249716	-1.567124868	-2.297600606	-2.921633431
NA	0.125366154	NA	-0.861695208
0.617191495	-0.504962638	NA	NA
0.302612028	-0.377205522	NA	NA
NA	-0.188477061	NA	1.022815874
NA	NA	0.626551197	0.472332098
NA	0.640462856	-5.072992338	1.18193823
-0.609514345	-0.428274194	NA	-0.614053405
-2.865086792	-1.194329738	-3.622801725	NA
-2.085671559	-0.759390471	NA	-0.803496544
NA	0.231239215	NA	1.475649276
NA	0.942327527	NA	0.558038135
NA	NA	NA	NA

Table S4: Hazard ratio of 33 oxidative stress genes

	ACC	BLCA	CHOL	COAD	DLBC
SOD3	NA	NA	NA	NA	NA
NFIX	NA	NA	NA	NA	NA
NFE2L2	NA	NA	NA	NA	NA
MAPK10	NA	0.865725223	NA	NA	NA
UGT1A6	NA	NA	NA	NA	8.25383E+11
NOX3	NA	NA	1.38E+68	NA	NA
NOX4	2.512576486	NA	3.727016972	1.658531431	NA
MGST1	NA	NA	NA	NA	NA
NQO1	1.01347611	NA	NA	NA	NA
SOD2	0.954997386	NA	NA	NA	NA
GSR	NA	1.005455563	NA	0.973694939	NA
JUNB	1.005923625	NA	NA	NA	NA
FOS	1.008078163	NA	NA	NA	NA
MT1X	NA	NA	NA	NA	NA
HMOX1	NA	NA	NA	NA	NA
NOX5	NA	NA	NA	3101456.087	NA
CAT	NA	NA	NA	NA	NA
GPX3	NA	1.001229733	NA	1.015396956	NA
CYBB	NA	NA	NA	NA	NA
GSTT2	NA	NA	NA	NA	NA
NFKB1	NA	NA	NA	NA	NA
GCLC	NA	NA	NA	NA	NA
XDH	1.543331478	NA	NA	0.912182864	NA
TXNRD1	NA	1.00681654	NA	NA	NA
SOD1	NA	NA	NA	NA	NA
SP1	1.092044897	NA	NA	NA	NA
TXN2	NA	NA	NA	0.977351429	NA
MAPK14	1.089823429	NA	NA	NA	NA
GPX1	NA	NA	NA	NA	NA
CYP1A1	59.4406026	NA	NA	NA	NA
NOX1	0.116706078	NA	NA	0.995184244	3.624643563
MAOA	NA	0.990560553	NA	NA	NA
TXNRD2	NA	NA	NA	NA	NA

ESCA	KICH	KIRC	KIRP	LAML	LIHC
NA	NA	NA	NA	NA	NA
0.973836743	NA	NA	1.080003205	NA	NA
NA	NA	0.971996264	NA	NA	NA
NA	NA	0.717608306	0.602993462	NA	NA
NA	NA	NA	NA	NA	1.033937102
NA	NA	NA	64116973200	NA	NA
NA	NA	NA	0.886990172	NA	NA
NA	NA	1.0115201	NA	0.975245488	NA
NA	NA	1.01606743	1.002258972	NA	1.002031516
NA	NA	1.002887455	NA	NA	NA
NA	NA	NA	NA	NA	1.020421406
NA	NA	NA	NA	1.002215162	NA
NA	NA	NA	NA	NA	NA
NA	NA	1.003163628	NA	NA	NA
NA	NA	0.997403844	NA	1.008152656	1.002645374
NA	NA	NA	NA	NA	561.6574966
NA	NA	0.981559106	NA	NA	0.996000413
NA	NA	NA	NA	NA	NA
NA	NA	NA	NA	NA	1.022473751
NA	NA	NA	NA	NA	NA
NA	NA	0.918321975	NA	NA	NA
NA	NA	0.863130022	1.058420599	NA	NA
NA	1228.264444	NA	NA	NA	NA
NA	NA	1.022932228	1.003704601	NA	1.014935366
NA	NA	NA	NA	1.024650838	NA
NA	NA	NA	NA	NA	NA
0.961848345	NA	NA	0.971892992	1.060790633	NA
NA	1.470030524	NA	NA	NA	NA
NA	NA	NA	NA	1.003634879	NA
NA	1.048433505	NA	NA	NA	NA
NA	NA	3.83524286	NA	NA	NA
NA	NA	NA	NA	NA	NA
NA	NA	NA	0.561434307	NA	NA

LUAD	LUSC	MESO	OV	PAAD	PCPG	PRAD
0.986696149	NA	NA	NA	NA	NA	NA
NA	NA	NA	NA	0.954080163	NA	NA
NA	NA	0.959975496	NA	NA	NA	NA
NA	NA	NA	NA	NA	NA	NA
NA	NA	46.51736934	NA	NA	NA	NA
NA	NA	NA	NA	NA	NA	NA
NA	NA	NA	NA	NA	NA	NA
NA	NA	NA	NA	NA	NA	NA
NA	NA	NA	NA	NA	NA	NA
1.007863675	NA	NA	NA	NA	NA	NA
NA	NA	NA	NA	NA	NA	NA
NA	1.001774085	NA	NA	NA	NA	NA
NA	1.001585816	NA	NA	NA	NA	NA
1.00871575	NA	NA	NA	NA	NA	NA
NA	NA	NA	NA	NA	NA	NA
NA	NA	NA	NA	50.65908738	NA	NA
0.985591302	NA	NA	NA	NA	NA	NA
NA	NA	NA	NA	NA	NA	NA
NA	NA	NA	NA	NA	NA	NA
NA	NA	NA	NA	NA	NA	NA
NA	NA	NA	NA	NA	NA	NA
1.005245027	NA	NA	NA	NA	NA	NA
NA	NA	NA	NA	1.027686513	NA	NA
1.001878065	NA	NA	NA	NA	NA	NA
1.00333498	NA	NA	NA	NA	NA	NA
1.039122943	NA	NA	NA	NA	NA	NA
NA	NA	NA	NA	NA	NA	NA
NA	NA	NA	NA	NA	NA	NA
NA	NA	NA	NA	NA	NA	NA
NA	NA	NA	NA	NA	NA	NA
NA	0.553454391	NA	NA	NA	NA	NA
NA	NA	NA	NA	NA	NA	NA
NA	NA	NA	NA	NA	NA	NA

READ	SARC	SKCM	STAD	TGCT	THCA
NA	NA	NA	NA	NA	NA
NA	NA	NA	NA	NA	NA
NA	NA	0.983456093	NA	NA	NA
NA	NA	1.369520523	1.356161302	NA	NA
NA	NA	625.7462605	NA	NA	5871.13486
NA	NA	NA	1.502812871	NA	NA
NA	NA	0.915061461	1.730025697	NA	NA
NA	NA	NA	NA	NA	0.955192278
NA	NA	NA	NA	NA	NA
0.816720387	NA	0.969525262	NA	NA	NA
NA	NA	NA	NA	NA	NA
NA	NA	NA	NA	NA	NA
NA	NA	NA	NA	NA	NA
NA	NA	NA	NA	NA	NA
NA	NA	NA	NA	NA	NA
NA	NA	NA	7.292740985	NA	NA
NA	NA	NA	NA	NA	NA
NA	NA	NA	NA	NA	NA
NA	NA	0.981249405	NA	1.028345641	NA
NA	25.76201011	NA	NA	NA	NA
0.761634235	1.077655684	NA	NA	NA	NA
NA	NA	NA	NA	NA	NA
NA	3.96591063	NA	NA	NA	NA
NA	NA	NA	NA	NA	NA
NA	NA	NA	NA	NA	NA
NA	NA	NA	NA	NA	NA
NA	NA	NA	NA	NA	NA
NA	NA	NA	NA	NA	NA
NA	0.99400374	NA	NA	NA	0.991143393
NA	1.993088606	NA	NA	NA	NA
NA	NA	NA	NA	NA	NA
NA	NA	NA	NA	NA	NA
NA	NA	1.075552906	NA	NA	NA

THYM	UCEC	UVM
NA	NA	NA
NA	NA	NA
NA	NA	NA
2.843137789	NA	NA
NA	NA	NA
NA	1.364524472	NA
NA	NA	NA
1.214890669	1.038535317	1.255909152
1.103832945	1.002086818	1.014386597
1.1076399	NA	1.086611277
NA	1.008933678	1.140517527
NA	NA	NA
NA	NA	NA
NA	NA	NA
NA	1.00246328	1.034538568
NA	NA	NA
NA	1.0216427	NA
NA	NA	0.993332584
1.065476233	NA	NA
NA	NA	NA
NA	NA	NA
1.41991098	1.031449696	NA
NA	1.033964159	NA
NA	1.011682064	1.122743846
1.016226093	NA	1.0182634
NA	NA	NA
NA	NA	NA
0.698263879	NA	NA
NA	NA	NA
NA	NA	NA
NA	NA	40.1337081
NA	NA	NA
NA	NA	1.231062953

Table S5: Interactions of oxidative stress genes

#node1	node2	node1_string_	node2_string_	neighborhoo(	gene_fusion	phylogenetic_
CAT	NOX3	9606.ENSP0(	9606.ENSP0(	0	0	0
CAT	HMOX1	9606.ENSP0(	9606.ENSP0(	0	0	0
CAT	TXN2	9606.ENSP0(	9606.ENSP0(	0	0	0
CAT	GSR	9606.ENSP0(	9606.ENSP0(	0	0	0
CAT	GCLC	9606.ENSP0(	9606.ENSP0(	0	0	0
CAT	MAPK14	9606.ENSP0(	9606.ENSP0(	0	0	0
CAT	MAPK10	9606.ENSP0(	9606.ENSP0(	0	0	0
CAT	MAOA	9606.ENSP0(	9606.ENSP0(	0	0	0
CAT	CYP1A1	9606.ENSP0(	9606.ENSP0(	0.045	0	0
CAT	FOS	9606.ENSP0(	9606.ENSP0(	0	0	0
CAT	NOX5	9606.ENSP0(	9606.ENSP0(	0	0	0
CAT	MT1X	9606.ENSP0(	9606.ENSP0(	0	0	0
CAT	XDH	9606.ENSP0(	9606.ENSP0(	0	0	0
CAT	NFE2L2	9606.ENSP0(	9606.ENSP0(	0	0	0
CAT	NOX1	9606.ENSP0(	9606.ENSP0(	0	0	0
CAT	NQO1	9606.ENSP0(	9606.ENSP0(	0	0	0
CAT	TXNRD2	9606.ENSP0(	9606.ENSP0(	0	0	0
CAT	NOX4	9606.ENSP0(	9606.ENSP0(	0	0	0
CAT	CYBB	9606.ENSP0(	9606.ENSP0(	0	0	0
CAT	GPX3	9606.ENSP0(	9606.ENSP0(	0	0	0
CAT	TXNRD1	9606.ENSP0(	9606.ENSP0(	0.142	0	0
CAT	GPX1	9606.ENSP0(	9606.ENSP0(	0	0	0
CAT	SOD3	9606.ENSP0(	9606.ENSP0(	0.11	0	0
CAT	SOD1	9606.ENSP0(	9606.ENSP0(	0.11	0	0
CAT	SOD2	9606.ENSP0(	9606.ENSP0(	0.069	0	0
CYBB	HMOX1	9606.ENSP0(	9606.ENSP0(	0	0	0
CYBB	GSR	9606.ENSP0(	9606.ENSP0(	0	0	0
CYBB	MAPK14	9606.ENSP0(	9606.ENSP0(	0	0	0
CYBB	NOX4	9606.ENSP0(	9606.ENSP0(	0	0	0
CYBB	SOD1	9606.ENSP0(	9606.ENSP0(	0	0	0
CYBB	NQO1	9606.ENSP0(	9606.ENSP0(	0	0	0
CYBB	MAPK10	9606.ENSP0(	9606.ENSP0(	0	0	0
CYBB	NOX1	9606.ENSP0(	9606.ENSP0(	0	0	0
CYBB	GPX3	9606.ENSP0(	9606.ENSP0(	0	0	0
CYBB	NFE2L2	9606.ENSP0(	9606.ENSP0(	0	0	0
CYBB	GPX1	9606.ENSP0(	9606.ENSP0(	0	0	0
CYBB	XDH	9606.ENSP0(	9606.ENSP0(	0	0	0
CYBB	SOD3	9606.ENSP0(	9606.ENSP0(	0	0	0
CYBB	SOD2	9606.ENSP0(	9606.ENSP0(	0	0	0
CYP1A1	HMOX1	9606.ENSP0(	9606.ENSP0(	0	0	0
CYP1A1	GSTT2B	9606.ENSP0(	9606.ENSP0(	0	0	0
CYP1A1	UGT1A6	9606.ENSP0(	9606.ENSP0(	0	0	0
CYP1A1	NQO1	9606.ENSP0(	9606.ENSP0(	0	0	0
CYP1A1	GPX1	9606.ENSP0(	9606.ENSP0(	0	0	0
CYP1A1	NFE2L2	9606.ENSP0(	9606.ENSP0(	0	0	0
CYP1A1	MGST1	9606.ENSP0(	9606.ENSP0(	0	0	0
FOS	HMOX1	9606.ENSP0(	9606.ENSP0(	0	0	0

FOS	NFKB1	9606.ENSP0(	9606.ENSP0(	0	0	0
FOS	MAPK14	9606.ENSP0(	9606.ENSP0(	0	0	0
FOS	UGT1A6	9606.ENSP0(	9606.ENSP0(	0	0	0
FOS	JUNB	9606.ENSP0(	9606.ENSP0(	0	0	0
FOS	NQO1	9606.ENSP0(	9606.ENSP0(	0	0	0
FOS	NFE2L2	9606.ENSP0(	9606.ENSP0(	0	0	0
FOS	SP1	9606.ENSP0(	9606.ENSP0(	0	0	0
FOS	MAPK10	9606.ENSP0(	9606.ENSP0(	0	0	0
GCLC	HMOX1	9606.ENSP0(	9606.ENSP0(	0	0	0
GCLC	TXN2	9606.ENSP0(	9606.ENSP0(	0	0	0
GCLC	GSR	9606.ENSP0(	9606.ENSP0(	0	0	0
GCLC	MGST1	9606.ENSP0(	9606.ENSP0(	0	0	0
GCLC	GSTT2B	9606.ENSP0(	9606.ENSP0(	0	0	0
GCLC	TXNRD2	9606.ENSP0(	9606.ENSP0(	0	0	0
GCLC	GPX3	9606.ENSP0(	9606.ENSP0(	0	0	0
GCLC	SOD3	9606.ENSP0(	9606.ENSP0(	0	0	0
GCLC	GPX1	9606.ENSP0(	9606.ENSP0(	0	0	0
GCLC	SOD1	9606.ENSP0(	9606.ENSP0(	0	0	0
GCLC	SOD2	9606.ENSP0(	9606.ENSP0(	0	0	0
GCLC	TXNRD1	9606.ENSP0(	9606.ENSP0(	0	0	0
GCLC	NQO1	9606.ENSP0(	9606.ENSP0(	0	0	0
GCLC	NFE2L2	9606.ENSP0(	9606.ENSP0(	0	0	0
GPX1	HMOX1	9606.ENSP0(	9606.ENSP0(	0	0	0
GPX1	TXN2	9606.ENSP0(	9606.ENSP0(	0	0	0
GPX1	GSR	9606.ENSP0(	9606.ENSP0(	0.049	0	0
GPX1	NOX4	9606.ENSP0(	9606.ENSP0(	0	0	0
GPX1	SOD1	9606.ENSP0(	9606.ENSP0(	0.044	0	0
GPX1	GSTT2B	9606.ENSP0(	9606.ENSP0(	0	0	0
GPX1	NQO1	9606.ENSP0(	9606.ENSP0(	0	0	0
GPX1	NOX1	9606.ENSP0(	9606.ENSP0(	0	0	0
GPX1	XDH	9606.ENSP0(	9606.ENSP0(	0	0	0
GPX1	SOD3	9606.ENSP0(	9606.ENSP0(	0.044	0	0
GPX1	MGST1	9606.ENSP0(	9606.ENSP0(	0	0	0
GPX1	NFE2L2	9606.ENSP0(	9606.ENSP0(	0	0	0
GPX1	TXNRD2	9606.ENSP0(	9606.ENSP0(	0.049	0	0
GPX1	TXNRD1	9606.ENSP0(	9606.ENSP0(	0.23	0	0
GPX1	SOD2	9606.ENSP0(	9606.ENSP0(	0	0	0
GPX3	HMOX1	9606.ENSP0(	9606.ENSP0(	0	0	0
GPX3	TXN2	9606.ENSP0(	9606.ENSP0(	0	0	0
GPX3	GSR	9606.ENSP0(	9606.ENSP0(	0.049	0	0
GPX3	NOX4	9606.ENSP0(	9606.ENSP0(	0	0	0
GPX3	SOD1	9606.ENSP0(	9606.ENSP0(	0.044	0	0
GPX3	GSTT2B	9606.ENSP0(	9606.ENSP0(	0	0	0
GPX3	NQO1	9606.ENSP0(	9606.ENSP0(	0	0	0
GPX3	NOX1	9606.ENSP0(	9606.ENSP0(	0	0	0
GPX3	SOD3	9606.ENSP0(	9606.ENSP0(	0.044	0	0
GPX3	NFE2L2	9606.ENSP0(	9606.ENSP0(	0	0	0
GPX3	TXNRD2	9606.ENSP0(	9606.ENSP0(	0.049	0	0
GPX3	MGST1	9606.ENSP0(	9606.ENSP0(	0	0	0

GPX3	TXNRD1	9606.ENSP0(	9606.ENSP0(	0.23	0	0
GPX3	SOD2	9606.ENSP0(	9606.ENSP0(	0	0	0
GSR	HMOX1	9606.ENSP0(	9606.ENSP0(	0	0	0
GSR	TXN2	9606.ENSP0(	9606.ENSP0(	0.05	0	0
GSR	TXNRD1	9606.ENSP0(	9606.ENSP0(	0.069	0	0.428
GSR	NOX1	9606.ENSP0(	9606.ENSP0(	0	0	0
GSR	NOX4	9606.ENSP0(	9606.ENSP0(	0	0	0
GSR	MT1X	9606.ENSP0(	9606.ENSP0(	0	0	0
GSR	XDH	9606.ENSP0(	9606.ENSP0(	0	0	0
GSR	NFE2L2	9606.ENSP0(	9606.ENSP0(	0	0	0
GSR	NQO1	9606.ENSP0(	9606.ENSP0(	0	0	0
GSR	SOD3	9606.ENSP0(	9606.ENSP0(	0	0	0
GSR	GSTT2B	9606.ENSP0(	9606.ENSP0(	0	0	0
GSR	MGST1	9606.ENSP0(	9606.ENSP0(	0	0	0
GSR	SOD1	9606.ENSP0(	9606.ENSP0(	0	0	0
GSR	SOD2	9606.ENSP0(	9606.ENSP0(	0.071	0	0
GSTT2B	NQO1	9606.ENSP0(	9606.ENSP0(	0	0	0
GSTT2B	TXNRD1	9606.ENSP0(	9606.ENSP0(	0	0	0
GSTT2B	MGST1	9606.ENSP0(	9606.ENSP0(	0	0	0
HMOX1	MT1X	9606.ENSP0(	9606.ENSP0(	0	0	0
HMOX1	UGT1A6	9606.ENSP0(	9606.ENSP0(	0	0	0
HMOX1	NOX1	9606.ENSP0(	9606.ENSP0(	0	0	0
HMOX1	SOD3	9606.ENSP0(	9606.ENSP0(	0	0	0
HMOX1	SOD1	9606.ENSP0(	9606.ENSP0(	0	0	0
HMOX1	TXNRD1	9606.ENSP0(	9606.ENSP0(	0	0	0
HMOX1	NOX4	9606.ENSP0(	9606.ENSP0(	0	0	0
HMOX1	SOD2	9606.ENSP0(	9606.ENSP0(	0.071	0	0
HMOX1	NQO1	9606.ENSP0(	9606.ENSP0(	0	0	0
HMOX1	MAPK14	9606.ENSP0(	9606.ENSP0(	0	0	0
HMOX1	NFE2L2	9606.ENSP0(	9606.ENSP0(	0	0	0
JUNB	MAPK14	9606.ENSP0(	9606.ENSP0(	0	0	0
JUNB	MAPK10	9606.ENSP0(	9606.ENSP0(	0	0	0
JUNB	SP1	9606.ENSP0(	9606.ENSP0(	0	0	0
MAPK10	NFKB1	9606.ENSP0(	9606.ENSP0(	0	0	0
MAPK10	MAPK14	9606.ENSP0(	9606.ENSP0(	0	0	0.347
MAPK10	SP1	9606.ENSP0(	9606.ENSP0(	0	0	0
MAPK14	NFKB1	9606.ENSP0(	9606.ENSP0(	0	0	0
MAPK14	NOX1	9606.ENSP0(	9606.ENSP0(	0	0	0
MAPK14	SOD3	9606.ENSP0(	9606.ENSP0(	0	0	0
MAPK14	SOD1	9606.ENSP0(	9606.ENSP0(	0	0	0
MAPK14	NOX4	9606.ENSP0(	9606.ENSP0(	0	0	0
MAPK14	SOD2	9606.ENSP0(	9606.ENSP0(	0	0	0
MAPK14	SP1	9606.ENSP0(	9606.ENSP0(	0	0	0
MGST1	NQO1	9606.ENSP0(	9606.ENSP0(	0	0	0
MT1X	SOD1	9606.ENSP0(	9606.ENSP0(	0	0	0
MT1X	NQO1	9606.ENSP0(	9606.ENSP0(	0	0	0
MT1X	SOD3	9606.ENSP0(	9606.ENSP0(	0	0	0
MT1X	SOD2	9606.ENSP0(	9606.ENSP0(	0	0	0
NFE2L2	NOX4	9606.ENSP0(	9606.ENSP0(	0	0	0

NFE2L2	SOD1	9606.ENSP0(	9606.ENSP0(	0	0	0
NFE2L2	UGT1A6	9606.ENSP0(	9606.ENSP0(	0	0	0
NFE2L2	NQO1	9606.ENSP0(	9606.ENSP0(	0	0	0
NFE2L2	SP1	9606.ENSP0(	9606.ENSP0(	0	0	0
NFE2L2	NOX1	9606.ENSP0(	9606.ENSP0(	0	0	0
NFE2L2	SOD3	9606.ENSP0(	9606.ENSP0(	0	0	0
NFE2L2	TXNRD1	9606.ENSP0(	9606.ENSP0(	0	0	0
NFE2L2	SOD2	9606.ENSP0(	9606.ENSP0(	0	0	0
NFKB1	SOD2	9606.ENSP0(	9606.ENSP0(	0	0	0
NFKB1	SP1	9606.ENSP0(	9606.ENSP0(	0	0	0
NOX1	NOX4	9606.ENSP0(	9606.ENSP0(	0	0	0
NOX1	SOD1	9606.ENSP0(	9606.ENSP0(	0	0	0
NOX1	NQO1	9606.ENSP0(	9606.ENSP0(	0	0	0
NOX1	XDH	9606.ENSP0(	9606.ENSP0(	0	0	0
NOX1	SOD2	9606.ENSP0(	9606.ENSP0(	0	0	0
NOX1	SOD3	9606.ENSP0(	9606.ENSP0(	0	0	0
NOX3	SOD2	9606.ENSP0(	9606.ENSP0(	0	0	0
NOX3	XDH	9606.ENSP0(	9606.ENSP0(	0	0	0
NOX3	SOD3	9606.ENSP0(	9606.ENSP0(	0	0	0
NOX3	NOX4	9606.ENSP0(	9606.ENSP0(	0	0	0
NOX3	SOD1	9606.ENSP0(	9606.ENSP0(	0	0	0
NOX4	TXN2	9606.ENSP0(	9606.ENSP0(	0	0	0
NOX4	NQO1	9606.ENSP0(	9606.ENSP0(	0	0	0
NOX4	TXNRD1	9606.ENSP0(	9606.ENSP0(	0	0	0
NOX4	XDH	9606.ENSP0(	9606.ENSP0(	0	0	0
NOX4	SOD1	9606.ENSP0(	9606.ENSP0(	0	0	0
NOX4	SOD3	9606.ENSP0(	9606.ENSP0(	0	0	0
NOX4	SOD2	9606.ENSP0(	9606.ENSP0(	0	0	0
NOX5	SOD1	9606.ENSP0(	9606.ENSP0(	0	0	0
NOX5	SOD3	9606.ENSP0(	9606.ENSP0(	0	0	0
NOX5	SOD2	9606.ENSP0(	9606.ENSP0(	0	0	0
NQO1	SOD1	9606.ENSP0(	9606.ENSP0(	0	0	0
NQO1	UGT1A6	9606.ENSP0(	9606.ENSP0(	0	0	0
NQO1	SOD3	9606.ENSP0(	9606.ENSP0(	0	0	0
NQO1	SOD2	9606.ENSP0(	9606.ENSP0(	0	0	0
NQO1	TXNRD1	9606.ENSP0(	9606.ENSP0(	0	0	0
SOD1	TXN2	9606.ENSP0(	9606.ENSP0(	0	0	0
SOD1	XDH	9606.ENSP0(	9606.ENSP0(	0	0	0
SOD1	TXNRD2	9606.ENSP0(	9606.ENSP0(	0	0	0
SOD1	TXNRD1	9606.ENSP0(	9606.ENSP0(	0	0	0
SOD1	SOD3	9606.ENSP0(	9606.ENSP0(	0	0	0.281
SOD1	SOD2	9606.ENSP0(	9606.ENSP0(	0	0	0
SOD2	TXN2	9606.ENSP0(	9606.ENSP0(	0	0	0
SOD2	SP1	9606.ENSP0(	9606.ENSP0(	0	0	0
SOD2	XDH	9606.ENSP0(	9606.ENSP0(	0	0	0
SOD2	SOD3	9606.ENSP0(	9606.ENSP0(	0	0	0
SOD2	TXNRD2	9606.ENSP0(	9606.ENSP0(	0.071	0	0
SOD2	TXNRD1	9606.ENSP0(	9606.ENSP0(	0.247	0	0
SOD3	TXN2	9606.ENSP0(	9606.ENSP0(	0	0	0

SOD3	XDH	9606.ENSP0(	9606.ENSP0(	0	0	0
SOD3	TXNRD2	9606.ENSP0(	9606.ENSP0(	0	0	0
SOD3	TXNRD1	9606.ENSP0(	9606.ENSP0(	0	0	0
TXN2	TXNRD1	9606.ENSP0(	9606.ENSP0(	0.179	0	0
TXN2	TXNRD2	9606.ENSP0(	9606.ENSP0(	0.05	0	0
TXNRD1	TXNRD2	9606.ENSP0(	9606.ENSP0(	0.069	0	0.444

homology	coexpression	experimentall	database_ann	automated_te	combined_score
0	0	0	0	0.574	0.574
0	0.062	0.194	0	0.883	0.904
0	0.126	0.185	0	0.816	0.858
0	0.225	0.457	0	0.972	0.987
0	0.141	0.457	0	0.838	0.917
0	0	0.177	0	0.604	0.66
0	0	0	0	0.451	0.451
0	0	0	0	0.49	0.49
0	0.061	0	0	0.501	0.514
0	0.061	0	0	0.519	0.529
0	0	0	0	0.645	0.645
0	0.077	0	0	0.676	0.688
0	0.062	0	0	0.71	0.717
0	0	0	0	0.743	0.743
0	0	0	0	0.748	0.748
0	0.062	0	0	0.751	0.756
0	0.116	0.323	0	0.636	0.763
0	0.062	0	0	0.775	0.78
0	0.067	0	0	0.778	0.784
0	0.14	0.134	0	0.861	0.887
0	0.188	0.323	0	0.806	0.896
0	0.14	0.134	0	0.905	0.923
0	0.129	0.13	0.9	0.866	0.989
0	0.129	0.44	0.9	0.937	0.996
0	0.08	0.419	0.9	0.968	0.998
0	0.069	0	0	0.76	0.767
0	0	0	0	0.495	0.495
0	0	0.124	0.65	0.416	0.805
0.874	0.061	0	0.9	0.96	0.913
0	0	0.265	0	0.679	0.754
0	0	0	0	0.438	0.438
0	0	0	0	0.555	0.555
0.959	0.061	0	0.8	0.939	0.811
0	0.061	0	0	0.44	0.452
0	0	0	0	0.477	0.477
0	0.062	0	0	0.565	0.575
0	0.063	0	0	0.608	0.617
0	0	0.164	0	0.626	0.674
0	0.092	0	0	0.693	0.709
0	0.064	0	0	0.535	0.547
0	0.061	0	0.65	0.425	0.794
0	0.179	0	0.65	0.741	0.919
0	0.085	0	0	0.741	0.753
0	0	0	0	0.412	0.412
0	0	0	0	0.466	0.466
0	0.062	0	0.65	0.264	0.737
0	0	0	0.9	0.482	0.946

0	0.051	0.402	0	0.512	0.699
0	0	0.264	0.9	0.733	0.978
0	0	0	0.9	0.115	0.907
0	0.824	0.884	0.9	0.852	0.999
0	0	0.379	0	0.412	0.619
0	0	0.051	0.9	0.426	0.94
0	0	0.073	0.9	0.476	0.947
0	0	0.26	0.9	0.56	0.964
0	0	0.261	0	0.855	0.888
0	0.062	0.094	0	0.379	0.426
0	0.188	0	0	0.881	0.899
0	0.061	0	0	0.389	0.401
0	0.077	0	0	0.44	0.461
0	0.115	0	0	0.455	0.497
0	0.061	0	0	0.505	0.516
0	0.052	0	0	0.59	0.594
0	0.061	0	0	0.755	0.76
0	0.061	0	0	0.757	0.762
0	0.065	0	0	0.852	0.855
0	0.189	0	0	0.831	0.857
0	0.062	0	0	0.876	0.878
0	0	0	0	0.885	0.886
0	0.081	0.17	0	0.718	0.766
0	0.061	0.106	0	0.691	0.718
0	0.064	0	0.9	0.872	0.987
0	0.061	0	0	0.576	0.585
0	0.093	0	0.9	0.866	0.986
0	0.064	0	0.65	0.345	0.766
0	0	0	0	0.675	0.675
0	0.061	0	0	0.517	0.527
0	0.062	0	0	0.39	0.403
0	0.093	0	0.9	0.731	0.973
0	0.064	0	0.65	0.404	0.787
0	0	0	0	0.591	0.591
0	0.063	0	0	0.724	0.733
0	0.148	0.065	0	0.841	0.889
0	0.061	0	0.9	0.885	0.988
0	0	0.17	0	0.638	0.686
0	0.061	0.106	0	0.654	0.684
0	0.064	0	0.9	0.853	0.985
0	0.061	0	0	0.495	0.505
0	0.093	0	0.9	0.73	0.973
0	0.064	0	0.65	0.324	0.759
0	0	0	0	0.498	0.498
0	0.061	0	0	0.41	0.422
0	0.16	0	0.9	0.665	0.969
0	0	0	0	0.443	0.443
0	0.063	0	0	0.659	0.669
0	0.061	0	0.65	0.307	0.752

0	0.148	0.065	0	0.778	0.846
0	0.061	0	0.9	0.747	0.974
0	0.082	0.125	0	0.692	0.731
0	0.079	0.266	0	0.759	0.824
0.809	0.208	0	0	0.87	0.407
0	0	0	0	0.467	0.467
0	0	0	0	0.493	0.493
0	0	0	0	0.538	0.538
0	0	0	0	0.563	0.563
0	0	0	0	0.674	0.674
0	0.063	0	0	0.711	0.717
0	0.061	0.13	0	0.763	0.789
0	0.109	0	0.65	0.386	0.792
0	0.061	0	0.65	0.433	0.797
0	0.072	0.13	0	0.868	0.884
0	0.113	0	0	0.906	0.916
0	0	0	0	0.4	0.4
0	0.112	0.083	0	0.387	0.457
0	0.062	0	0.65	0.465	0.809
0	0	0	0	0.428	0.428
0	0	0	0	0.498	0.498
0	0	0	0	0.57	0.57
0	0	0	0	0.591	0.591
0	0	0	0	0.728	0.728
0	0.065	0.157	0	0.699	0.742
0	0	0	0	0.754	0.754
0	0.063	0	0	0.763	0.776
0	0.056	0	0	0.951	0.952
0	0	0	0.9	0.735	0.972
0	0	0	0.9	0.968	0.996
0	0	0.424	0.6	0.195	0.798
0	0	0.351	0.6	0.325	0.809
0	0.046	0.095	0.9	0.295	0.931
0	0	0.261	0.8	0.285	0.885
0.921	0.064	0.052	0.9	0.594	0.91
0	0	0.071	0.9	0.208	0.92
0	0.065	0.185	0.9	0.409	0.948
0	0	0.124	0	0.351	0.407
0	0	0.157	0	0.378	0.453
0	0	0.177	0	0.413	0.496
0	0	0.087	0	0.578	0.598
0	0.059	0.185	0	0.53	0.608
0	0	0.443	0.9	0.376	0.962
0	0.088	0	0	0.39	0.419
0	0.049	0.17	0	0.535	0.601
0	0	0	0	0.509	0.509
0	0	0.17	0	0.333	0.423
0	0.062	0	0	0.487	0.498
0	0	0	0	0.515	0.515

0	0	0	0	0.683	0.683
0	0	0	0.9	0.402	0.937
0	0	0	0	0.94	0.941
0	0	0.294	0	0.25	0.447
0	0	0	0	0.445	0.445
0	0	0	0	0.51	0.51
0	0	0	0	0.638	0.638
0	0	0	0	0.82	0.821
0	0	0	0	0.696	0.697
0	0.072	0.432	0	0.535	0.734
0.859	0.061	0	0.8	0.945	0.83
0	0	0.19	0	0.585	0.65
0	0	0	0	0.428	0.428
0	0.055	0	0	0.569	0.575
0	0.064	0	0	0.606	0.615
0	0.061	0.164	0	0.6	0.658
0	0.064	0	0	0.396	0.41
0	0.058	0	0	0.411	0.421
0	0	0.164	0	0.358	0.44
0.859	0.061	0	0.36	0.885	0.451
0	0	0.26	0	0.374	0.517
0	0.061	0.05	0	0.427	0.445
0	0	0	0	0.402	0.402
0	0	0	0	0.444	0.444
0	0.061	0	0	0.574	0.583
0	0	0.164	0	0.629	0.676
0	0.061	0.164	0	0.625	0.68
0	0.064	0	0	0.692	0.699
0	0	0.164	0	0.595	0.647
0	0	0.164	0	0.447	0.518
0	0.064	0	0	0.497	0.509
0	0.061	0	0	0.665	0.672
0	0.062	0	0	0.698	0.704
0	0.046	0	0	0.453	0.456
0	0	0	0	0.703	0.703
0	0.152	0	0	0.708	0.742
0	0.104	0.504	0	0.511	0.764
0	0	0	0	0.571	0.571
0	0.061	0.185	0	0.625	0.688
0	0.068	0.185	0	0.733	0.78
0.865	0	0	0.9	0.84	0.914
0	0.176	0.624	0.9	0.959	0.998
0	0.083	0	0	0.697	0.711
0	0	0.081	0	0.578	0.595
0	0	0	0	0.556	0.556
0	0.114	0.345	0.9	0.929	0.995
0	0.109	0	0	0.685	0.716
0	0.147	0	0	0.834	0.884
0	0.064	0.186	0	0.336	0.45

0	0.063	0	0	0.539	0.549
0	0.061	0.168	0	0.5	0.575
0	0.063	0.168	0	0.671	0.721
0	0.135	0.339	0	0.728	0.855
0	0.079	0.266	0.9	0.766	0.982
0.95	0.082	0	0.8	0.883	0.826

Table S6: Three levels of oxidative stress gene mRNA regulation

	TCGA-A3-3306-01	TCGA-A3-3329-01	TCGA-B8-5550-01	TCGA-A3-3378-01
MGST1	NOCHANGE	NOCHANGE	NOCHANGE	NOCHANGE
HMOX1	UP	UP	UP	UP
NOX4	NOCHANGE	NOCHANGE	NOCHANGE	NOCHANGE
MAPK10	DOWN	DOWN	DOWN	DOWN
SOD2	NOCHANGE	UP	UP	UP
UGT1A6	NOCHANGE	NOCHANGE	UP	UP
CYP1A1	NOCHANGE	DOWN	DOWN	DOWN
MT1X	NOCHANGE	NOCHANGE	NOCHANGE	NOCHANGE
XDH	NOCHANGE	DOWN	UP	UP
TXNRD2	NOCHANGE	NOCHANGE	NOCHANGE	NOCHANGE
NOX1	NOCHANGE	NOCHANGE	DOWN	NOCHANGE
NOX5	DOWN	DOWN	DOWN	DOWN
GPX3	NOCHANGE	NOCHANGE	NOCHANGE	NOCHANGE
MAPK14	NOCHANGE	NOCHANGE	NOCHANGE	NOCHANGE
SOD3	DOWN	DOWN	NOCHANGE	DOWN
GCLC	NOCHANGE	NOCHANGE	NOCHANGE	NOCHANGE
NFKB1	NOCHANGE	UP	NOCHANGE	NOCHANGE
SOD1	NOCHANGE	NOCHANGE	DOWN	DOWN
SP1	NOCHANGE	NOCHANGE	NOCHANGE	UP
MAOA	UP	NOCHANGE	NOCHANGE	NOCHANGE
CYBB	NOCHANGE	UP	UP	UP
NFIX	NOCHANGE	UP	UP	NOCHANGE
TXNRD1	DOWN	NOCHANGE	NOCHANGE	NOCHANGE
TXN2	UP	NOCHANGE	NOCHANGE	DOWN
CAT	NOCHANGE	NOCHANGE	DOWN	NOCHANGE
NQO1	DOWN	DOWN	NOCHANGE	DOWN
NOX3	NOCHANGE	NOCHANGE	NOCHANGE	NOCHANGE
JUNB	NOCHANGE	NOCHANGE	NOCHANGE	NOCHANGE
NFE2L2	NOCHANGE	NOCHANGE	NOCHANGE	NOCHANGE
FOS	NOCHANGE	NOCHANGE	DOWN	NOCHANGE
GSR	NOCHANGE	DOWN	NOCHANGE	DOWN

TCGA-A3-3343-01	TCGA-B0-5100-01.	TCGA-CJ-4869-01/	TCGA-CJ-4895-01/	TCGA-B0-5691-01.
NOCHANGE	NOCHANGE	NOCHANGE	NOCHANGE	NOCHANGE
UP	NOCHANGE	UP	UP	UP
NOCHANGE	NOCHANGE	NOCHANGE	NOCHANGE	NOCHANGE
DOWN	DOWN	DOWN	DOWN	NOCHANGE
UP	DOWN	UP	UP	NOCHANGE
NOCHANGE	DOWN	NOCHANGE	UP	NOCHANGE
DOWN	DOWN	NOCHANGE	DOWN	NOCHANGE
NOCHANGE	NOCHANGE	NOCHANGE	NOCHANGE	NOCHANGE
NOCHANGE	NOCHANGE	UP	NOCHANGE	NOCHANGE
NOCHANGE	NOCHANGE	NOCHANGE	NOCHANGE	NOCHANGE
NOCHANGE	UP	NOCHANGE	NOCHANGE	DOWN
NOCHANGE	DOWN	NOCHANGE	DOWN	NOCHANGE
NOCHANGE	NOCHANGE	NOCHANGE	NOCHANGE	NOCHANGE
NOCHANGE	DOWN	NOCHANGE	NOCHANGE	NOCHANGE
DOWN	UP	NOCHANGE	NOCHANGE	NOCHANGE
NOCHANGE	DOWN	NOCHANGE	NOCHANGE	NOCHANGE
NOCHANGE	DOWN	NOCHANGE	UP	NOCHANGE
NOCHANGE	NOCHANGE	NOCHANGE	DOWN	DOWN
NOCHANGE	NOCHANGE	NOCHANGE	NOCHANGE	NOCHANGE
DOWN	DOWN	DOWN	DOWN	NOCHANGE
UP	NOCHANGE	NOCHANGE	UP	NOCHANGE
UP	UP	NOCHANGE	NOCHANGE	UP
DOWN	DOWN	NOCHANGE	NOCHANGE	NOCHANGE
NOCHANGE	NOCHANGE	NOCHANGE	DOWN	DOWN
NOCHANGE	DOWN	DOWN	NOCHANGE	NOCHANGE
NOCHANGE	DOWN	NOCHANGE	NOCHANGE	NOCHANGE
NOCHANGE	NOCHANGE	NOCHANGE	NOCHANGE	NOCHANGE
NOCHANGE	NOCHANGE	NOCHANGE	NOCHANGE	NOCHANGE
DOWN	DOWN	NOCHANGE	NOCHANGE	NOCHANGE
NOCHANGE	NOCHANGE	NOCHANGE	NOCHANGE	NOCHANGE
NOCHANGE	DOWN	NOCHANGE	NOCHANGE	NOCHANGE

TCGA-B0-5121-01.	TCGA-B0-5098-01.	TCGA-A3-3308-01	TCGA-BP-4807-01.	TCGA-B0-5707-01.
NOCHANGE	NOCHANGE	NOCHANGE	NOCHANGE	NOCHANGE
UP	NOCHANGE	UP	UP	NOCHANGE
NOCHANGE	NOCHANGE	NOCHANGE	NOCHANGE	NOCHANGE
DOWN	DOWN	NOCHANGE	DOWN	DOWN
NOCHANGE	NOCHANGE	UP	NOCHANGE	NOCHANGE
NOCHANGE	NOCHANGE	NOCHANGE	NOCHANGE	NOCHANGE
DOWN	NOCHANGE	NOCHANGE	NOCHANGE	NOCHANGE
NOCHANGE	NOCHANGE	NOCHANGE	DOWN	NOCHANGE
DOWN	NOCHANGE	NOCHANGE	DOWN	UP
NOCHANGE	NOCHANGE	NOCHANGE	NOCHANGE	NOCHANGE
NOCHANGE	NOCHANGE	DOWN	NOCHANGE	NOCHANGE
NOCHANGE	NOCHANGE	UP	NOCHANGE	DOWN
NOCHANGE	NOCHANGE	NOCHANGE	NOCHANGE	NOCHANGE
DOWN	NOCHANGE	NOCHANGE	NOCHANGE	NOCHANGE
DOWN	DOWN	DOWN	NOCHANGE	NOCHANGE
NOCHANGE	UP	NOCHANGE	NOCHANGE	NOCHANGE
NOCHANGE	NOCHANGE	NOCHANGE	NOCHANGE	NOCHANGE
NOCHANGE	NOCHANGE	DOWN	DOWN	NOCHANGE
NOCHANGE	NOCHANGE	NOCHANGE	NOCHANGE	NOCHANGE
DOWN	DOWN	DOWN	DOWN	NOCHANGE
NOCHANGE	NOCHANGE	UP	UP	NOCHANGE
NOCHANGE	NOCHANGE	UP	NOCHANGE	NOCHANGE
DOWN	UP	NOCHANGE	NOCHANGE	NOCHANGE
DOWN	DOWN	DOWN	NOCHANGE	NOCHANGE
DOWN	DOWN	DOWN	NOCHANGE	DOWN
NOCHANGE	UP	DOWN	NOCHANGE	NOCHANGE
NOCHANGE	NOCHANGE	NOCHANGE	NOCHANGE	NOCHANGE
NOCHANGE	NOCHANGE	NOCHANGE	NOCHANGE	NOCHANGE
DOWN	NOCHANGE	NOCHANGE	NOCHANGE	NOCHANGE
DOWN	NOCHANGE	NOCHANGE	NOCHANGE	NOCHANGE
DOWN	UP	NOCHANGE	DOWN	NOCHANGE

TCGA-BP-4971-01.	TCGA-B8-4620-01.	TCGA-BP-4967-01.	TCGA-BP-5198-01.	TCGA-BP-4326-01.
NOCHANGE	NOCHANGE	NOCHANGE	NOCHANGE	NOCHANGE
UP	NOCHANGE	UP	UP	NOCHANGE
NOCHANGE	NOCHANGE	NOCHANGE	NOCHANGE	NOCHANGE
DOWN	DOWN	DOWN	DOWN	DOWN
NOCHANGE	NOCHANGE	NOCHANGE	UP	UP
NOCHANGE	NOCHANGE	NOCHANGE	UP	DOWN
NOCHANGE	DOWN	DOWN	DOWN	DOWN
NOCHANGE	NOCHANGE	NOCHANGE	UP	NOCHANGE
NOCHANGE	DOWN	NOCHANGE	NOCHANGE	NOCHANGE
NOCHANGE	DOWN	NOCHANGE	NOCHANGE	DOWN
NOCHANGE	NOCHANGE	DOWN	DOWN	DOWN
UP	NOCHANGE	NOCHANGE	DOWN	NOCHANGE
NOCHANGE	NOCHANGE	NOCHANGE	NOCHANGE	NOCHANGE
DOWN	DOWN	NOCHANGE	NOCHANGE	DOWN
NOCHANGE	DOWN	DOWN	NOCHANGE	DOWN
NOCHANGE	NOCHANGE	NOCHANGE	NOCHANGE	NOCHANGE
NOCHANGE	NOCHANGE	NOCHANGE	NOCHANGE	NOCHANGE
DOWN	DOWN	DOWN	DOWN	DOWN
NOCHANGE	NOCHANGE	NOCHANGE	NOCHANGE	NOCHANGE
DOWN	DOWN	NOCHANGE	DOWN	DOWN
UP	UP	NOCHANGE	NOCHANGE	NOCHANGE
NOCHANGE	NOCHANGE	UP	NOCHANGE	UP
DOWN	NOCHANGE	NOCHANGE	NOCHANGE	DOWN
DOWN	DOWN	DOWN	NOCHANGE	DOWN
DOWN	DOWN	DOWN	DOWN	DOWN
DOWN	NOCHANGE	DOWN	DOWN	DOWN
NOCHANGE	NOCHANGE	NOCHANGE	NOCHANGE	NOCHANGE
NOCHANGE	NOCHANGE	NOCHANGE	NOCHANGE	UP
DOWN	NOCHANGE	DOWN	DOWN	NOCHANGE
NOCHANGE	NOCHANGE	NOCHANGE	NOCHANGE	NOCHANGE
DOWN	DOWN	DOWN	NOCHANGE	DOWN

TCGA-CJ-4904-01/	TCGA-CZ-4853-01	TCGA-A3-3387-01	TCGA-A3-3326-01	TCGA-BP-4784-01.
NOCHANGE	NOCHANGE	NOCHANGE	NOCHANGE	NOCHANGE
NOCHANGE	UP	NOCHANGE	UP	NOCHANGE
NOCHANGE	NOCHANGE	NOCHANGE	NOCHANGE	NOCHANGE
DOWN	DOWN	DOWN	DOWN	DOWN
NOCHANGE	NOCHANGE	UP	NOCHANGE	NOCHANGE
NOCHANGE	UP	NOCHANGE	UP	NOCHANGE
NOCHANGE	DOWN	NOCHANGE	NOCHANGE	DOWN
NOCHANGE	DOWN	NOCHANGE	NOCHANGE	NOCHANGE
NOCHANGE	NOCHANGE	NOCHANGE	UP	UP
NOCHANGE	NOCHANGE	NOCHANGE	NOCHANGE	DOWN
NOCHANGE	DOWN	DOWN	DOWN	NOCHANGE
NOCHANGE	DOWN	UP	NOCHANGE	UP
NOCHANGE	NOCHANGE	NOCHANGE	NOCHANGE	DOWN
NOCHANGE	DOWN	NOCHANGE	NOCHANGE	NOCHANGE
DOWN	DOWN	NOCHANGE	DOWN	NOCHANGE
UP	NOCHANGE	NOCHANGE	UP	NOCHANGE
UP	NOCHANGE	NOCHANGE	UP	NOCHANGE
DOWN	NOCHANGE	DOWN	DOWN	DOWN
NOCHANGE	UP	NOCHANGE	UP	NOCHANGE
NOCHANGE	NOCHANGE	NOCHANGE	DOWN	DOWN
NOCHANGE	NOCHANGE	UP	UP	NOCHANGE
NOCHANGE	UP	NOCHANGE	UP	NOCHANGE
NOCHANGE	NOCHANGE	UP	NOCHANGE	DOWN
DOWN	NOCHANGE	DOWN	DOWN	DOWN
NOCHANGE	NOCHANGE	DOWN	DOWN	DOWN
NOCHANGE	NOCHANGE	NOCHANGE	NOCHANGE	NOCHANGE
NOCHANGE	NOCHANGE	NOCHANGE	NOCHANGE	NOCHANGE
NOCHANGE	NOCHANGE	NOCHANGE	NOCHANGE	NOCHANGE
NOCHANGE	DOWN	NOCHANGE	DOWN	DOWN
NOCHANGE	NOCHANGE	DOWN	NOCHANGE	NOCHANGE
DOWN	DOWN	DOWN	NOCHANGE	NOCHANGE

TCGA-BP-4766-01	TCGA-CZ-5456-01	TCGA-CZ-4857-01	TCGA-B0-5400-01	TCGA-BP-5010-01
NOCHANGE	UP	NOCHANGE	NOCHANGE	NOCHANGE
UP	UP	UP	UP	UP
NOCHANGE	NOCHANGE	NOCHANGE	NOCHANGE	NOCHANGE
DOWN	DOWN	DOWN	DOWN	NOCHANGE
NOCHANGE	UP	UP	UP	NOCHANGE
UP	UP	NOCHANGE	UP	NOCHANGE
DOWN	DOWN	DOWN	DOWN	NOCHANGE
DOWN	NOCHANGE	NOCHANGE	NOCHANGE	NOCHANGE
DOWN	DOWN	NOCHANGE	UP	NOCHANGE
NOCHANGE	NOCHANGE	NOCHANGE	NOCHANGE	NOCHANGE
DOWN	DOWN	DOWN	NOCHANGE	NOCHANGE
NOCHANGE	DOWN	NOCHANGE	DOWN	NOCHANGE
NOCHANGE	DOWN	DOWN	NOCHANGE	NOCHANGE
NOCHANGE	NOCHANGE	NOCHANGE	NOCHANGE	DOWN
DOWN	DOWN	DOWN	NOCHANGE	NOCHANGE
UP	NOCHANGE	NOCHANGE	NOCHANGE	NOCHANGE
UP	NOCHANGE	NOCHANGE	DOWN	NOCHANGE
DOWN	DOWN	DOWN	NOCHANGE	DOWN
NOCHANGE	UP	UP	DOWN	NOCHANGE
DOWN	UP	DOWN	DOWN	DOWN
UP	UP	UP	NOCHANGE	NOCHANGE
NOCHANGE	NOCHANGE	NOCHANGE	NOCHANGE	NOCHANGE
NOCHANGE	UP	NOCHANGE	NOCHANGE	DOWN
NOCHANGE	DOWN	DOWN	NOCHANGE	NOCHANGE
NOCHANGE	DOWN	DOWN	DOWN	DOWN
NOCHANGE	UP	DOWN	NOCHANGE	DOWN
NOCHANGE	NOCHANGE	NOCHANGE	NOCHANGE	NOCHANGE
NOCHANGE	NOCHANGE	NOCHANGE	NOCHANGE	NOCHANGE
NOCHANGE	NOCHANGE	DOWN	DOWN	DOWN
NOCHANGE	NOCHANGE	NOCHANGE	NOCHANGE	NOCHANGE
NOCHANGE	UP	DOWN	NOCHANGE	DOWN

TCGA-CJ-4644-01/	TCGA-CJ-6033-01/	TCGA-EU-5907-01	TCGA-B4-5836-01.	TCGA-A3-3323-01
NOCHANGE	NOCHANGE	NOCHANGE	NOCHANGE	NOCHANGE
UP	UP	UP	UP	UP
NOCHANGE	NOCHANGE	NOCHANGE	NOCHANGE	NOCHANGE
DOWN	DOWN	DOWN	DOWN	DOWN
NOCHANGE	NOCHANGE	NOCHANGE	UP	UP
UP	NOCHANGE	NOCHANGE	NOCHANGE	NOCHANGE
DOWN	DOWN	DOWN	DOWN	NOCHANGE
DOWN	NOCHANGE	NOCHANGE	NOCHANGE	NOCHANGE
DOWN	NOCHANGE	NOCHANGE	NOCHANGE	NOCHANGE
NOCHANGE	NOCHANGE	NOCHANGE	NOCHANGE	NOCHANGE
NOCHANGE	NOCHANGE	NOCHANGE	NOCHANGE	DOWN
DOWN	NOCHANGE	DOWN	NOCHANGE	NOCHANGE
NOCHANGE	NOCHANGE	NOCHANGE	NOCHANGE	NOCHANGE
NOCHANGE	DOWN	NOCHANGE	NOCHANGE	NOCHANGE
DOWN	DOWN	DOWN	DOWN	DOWN
NOCHANGE	NOCHANGE	NOCHANGE	NOCHANGE	NOCHANGE
NOCHANGE	NOCHANGE	NOCHANGE	UP	NOCHANGE
NOCHANGE	NOCHANGE	NOCHANGE	NOCHANGE	DOWN
NOCHANGE	NOCHANGE	NOCHANGE	NOCHANGE	UP
NOCHANGE	NOCHANGE	NOCHANGE	NOCHANGE	DOWN
UP	NOCHANGE	NOCHANGE	NOCHANGE	UP
NOCHANGE	NOCHANGE	UP	NOCHANGE	UP
NOCHANGE	NOCHANGE	NOCHANGE	NOCHANGE	NOCHANGE
NOCHANGE	NOCHANGE	NOCHANGE	DOWN	DOWN
NOCHANGE	DOWN	DOWN	NOCHANGE	DOWN
NOCHANGE	NOCHANGE	NOCHANGE	NOCHANGE	DOWN
NOCHANGE	NOCHANGE	NOCHANGE	NOCHANGE	NOCHANGE
NOCHANGE	NOCHANGE	NOCHANGE	NOCHANGE	DOWN
NOCHANGE	NOCHANGE	NOCHANGE	NOCHANGE	DOWN
DOWN	NOCHANGE	DOWN	NOCHANGE	DOWN
NOCHANGE	DOWN	NOCHANGE	NOCHANGE	NOCHANGE

TCGA-BP-4353-01.	TCGA-B0-5077-01.	TCGA-CZ-5984-01	TCGA-B8-A54G-01	TCGA-B0-4713-01.
NOCHANGE	NOCHANGE	UP	NOCHANGE	UP
UP	UP	UP	UP	UP
NOCHANGE	NOCHANGE	NOCHANGE	NOCHANGE	NOCHANGE
DOWN	DOWN	DOWN	DOWN	DOWN
NOCHANGE	NOCHANGE	UP	NOCHANGE	UP
NOCHANGE	UP	NOCHANGE	NOCHANGE	UP
DOWN	DOWN	NOCHANGE	DOWN	DOWN
NOCHANGE	NOCHANGE	NOCHANGE	NOCHANGE	NOCHANGE
NOCHANGE	NOCHANGE	UP	NOCHANGE	UP
DOWN	NOCHANGE	NOCHANGE	NOCHANGE	NOCHANGE
NOCHANGE	DOWN	NOCHANGE	NOCHANGE	UP
NOCHANGE	NOCHANGE	NOCHANGE	DOWN	NOCHANGE
NOCHANGE	NOCHANGE	NOCHANGE	NOCHANGE	NOCHANGE
NOCHANGE	NOCHANGE	NOCHANGE	NOCHANGE	NOCHANGE
DOWN	DOWN	DOWN	NOCHANGE	DOWN
UP	NOCHANGE	NOCHANGE	NOCHANGE	NOCHANGE
NOCHANGE	NOCHANGE	NOCHANGE	UP	DOWN
DOWN	DOWN	NOCHANGE	NOCHANGE	NOCHANGE
NOCHANGE	NOCHANGE	UP	UP	NOCHANGE
NOCHANGE	DOWN	UP	NOCHANGE	NOCHANGE
UP	NOCHANGE	UP	UP	NOCHANGE
NOCHANGE	UP	UP	NOCHANGE	NOCHANGE
NOCHANGE	DOWN	NOCHANGE	DOWN	NOCHANGE
DOWN	DOWN	NOCHANGE	NOCHANGE	NOCHANGE
DOWN	DOWN	DOWN	DOWN	NOCHANGE
NOCHANGE	NOCHANGE	NOCHANGE	DOWN	NOCHANGE
NOCHANGE	NOCHANGE	NOCHANGE	NOCHANGE	NOCHANGE
NOCHANGE	NOCHANGE	NOCHANGE	NOCHANGE	NOCHANGE
NOCHANGE	DOWN	NOCHANGE	NOCHANGE	DOWN
NOCHANGE	NOCHANGE	DOWN	DOWN	DOWN
DOWN	DOWN	DOWN	NOCHANGE	NOCHANGE

TCGA-CZ-5463-01	TCGA-6D-AA2E-0	TCGA-CJ-6032-01/	TCGA-BP-4801-01.	TCGA-EU-5904-01
NOCHANGE	NOCHANGE	NOCHANGE	NOCHANGE	NOCHANGE
UP	NOCHANGE	UP	UP	UP
NOCHANGE	NOCHANGE	NOCHANGE	NOCHANGE	NOCHANGE
NOCHANGE	DOWN	DOWN	DOWN	NOCHANGE
NOCHANGE	NOCHANGE	NOCHANGE	NOCHANGE	NOCHANGE
NOCHANGE	UP	UP	UP	NOCHANGE
DOWN	DOWN	DOWN	NOCHANGE	NOCHANGE
NOCHANGE	NOCHANGE	DOWN	NOCHANGE	NOCHANGE
DOWN	NOCHANGE	NOCHANGE	NOCHANGE	DOWN
NOCHANGE	NOCHANGE	NOCHANGE	NOCHANGE	NOCHANGE
DOWN	NOCHANGE	DOWN	DOWN	NOCHANGE
DOWN	NOCHANGE	NOCHANGE	DOWN	DOWN
NOCHANGE	NOCHANGE	NOCHANGE	NOCHANGE	NOCHANGE
NOCHANGE	NOCHANGE	NOCHANGE	NOCHANGE	NOCHANGE
DOWN	NOCHANGE	DOWN	NOCHANGE	DOWN
UP	NOCHANGE	NOCHANGE	UP	NOCHANGE
NOCHANGE	NOCHANGE	UP	NOCHANGE	NOCHANGE
NOCHANGE	NOCHANGE	DOWN	NOCHANGE	DOWN
NOCHANGE	DOWN	NOCHANGE	NOCHANGE	NOCHANGE
NOCHANGE	NOCHANGE	NOCHANGE	DOWN	DOWN
UP	NOCHANGE	NOCHANGE	UP	NOCHANGE
NOCHANGE	NOCHANGE	NOCHANGE	NOCHANGE	UP
NOCHANGE	NOCHANGE	NOCHANGE	UP	DOWN
NOCHANGE	NOCHANGE	DOWN	NOCHANGE	DOWN
NOCHANGE	NOCHANGE	NOCHANGE	DOWN	NOCHANGE
NOCHANGE	DOWN	NOCHANGE	UP	NOCHANGE
NOCHANGE	NOCHANGE	NOCHANGE	NOCHANGE	NOCHANGE
NOCHANGE	NOCHANGE	NOCHANGE	NOCHANGE	UP
NOCHANGE	NOCHANGE	UP	NOCHANGE	NOCHANGE
DOWN	NOCHANGE	DOWN	NOCHANGE	UP
DOWN	NOCHANGE	NOCHANGE	NOCHANGE	DOWN

TCGA-B8-5158-01.	TCGA-BP-4969-01.	TCGA-B8-5546-01.	TCGA-B4-5844-01.	TCGA-CZ-5452-01
NOCHANGE	NOCHANGE	NOCHANGE	NOCHANGE	NOCHANGE
UP	UP	UP	UP	UP
NOCHANGE	NOCHANGE	NOCHANGE	NOCHANGE	NOCHANGE
DOWN	NOCHANGE	NOCHANGE	DOWN	NOCHANGE
UP	DOWN	NOCHANGE	NOCHANGE	NOCHANGE
UP	NOCHANGE	NOCHANGE	UP	NOCHANGE
DOWN	NOCHANGE	DOWN	DOWN	DOWN
NOCHANGE	NOCHANGE	NOCHANGE	NOCHANGE	DOWN
UP	NOCHANGE	DOWN	DOWN	NOCHANGE
NOCHANGE	NOCHANGE	NOCHANGE	NOCHANGE	NOCHANGE
NOCHANGE	NOCHANGE	NOCHANGE	NOCHANGE	NOCHANGE
NOCHANGE	UP	DOWN	NOCHANGE	NOCHANGE
NOCHANGE	NOCHANGE	NOCHANGE	NOCHANGE	NOCHANGE
NOCHANGE	DOWN	NOCHANGE	NOCHANGE	NOCHANGE
DOWN	DOWN	DOWN	DOWN	DOWN
NOCHANGE	NOCHANGE	UP	UP	NOCHANGE
NOCHANGE	DOWN	NOCHANGE	UP	UP
DOWN	NOCHANGE	NOCHANGE	DOWN	NOCHANGE
NOCHANGE	NOCHANGE	NOCHANGE	NOCHANGE	NOCHANGE
UP	DOWN	NOCHANGE	DOWN	DOWN
UP	NOCHANGE	NOCHANGE	NOCHANGE	UP
NOCHANGE	NOCHANGE	NOCHANGE	NOCHANGE	NOCHANGE
NOCHANGE	DOWN	NOCHANGE	NOCHANGE	DOWN
DOWN	DOWN	NOCHANGE	DOWN	NOCHANGE
DOWN	DOWN	NOCHANGE	NOCHANGE	NOCHANGE
NOCHANGE	NOCHANGE	DOWN	NOCHANGE	DOWN
NOCHANGE	NOCHANGE	NOCHANGE	NOCHANGE	NOCHANGE
NOCHANGE	NOCHANGE	DOWN	NOCHANGE	NOCHANGE
NOCHANGE	DOWN	NOCHANGE	NOCHANGE	DOWN
NOCHANGE	NOCHANGE	DOWN	DOWN	NOCHANGE
NOCHANGE	DOWN	UP	DOWN	NOCHANGE

TCGA-BP-4991-01.	TCGA-A3-3316-01	TCGA-B8-A54E-01	TCGA-BP-5170-01.	TCGA-A3-A6NI-01
NOCHANGE	UP	NOCHANGE	NOCHANGE	NOCHANGE
UP	UP	UP	UP	UP
NOCHANGE	NOCHANGE	NOCHANGE	NOCHANGE	NOCHANGE
NOCHANGE	DOWN	UP	DOWN	DOWN
NOCHANGE	NOCHANGE	NOCHANGE	NOCHANGE	NOCHANGE
NOCHANGE	NOCHANGE	UP	NOCHANGE	NOCHANGE
DOWN	NOCHANGE	NOCHANGE	DOWN	NOCHANGE
DOWN	NOCHANGE	NOCHANGE	NOCHANGE	NOCHANGE
NOCHANGE	NOCHANGE	DOWN	NOCHANGE	DOWN
NOCHANGE	NOCHANGE	NOCHANGE	DOWN	NOCHANGE
NOCHANGE	NOCHANGE	NOCHANGE	NOCHANGE	NOCHANGE
NOCHANGE	UP	UP	NOCHANGE	DOWN
NOCHANGE	NOCHANGE	NOCHANGE	NOCHANGE	NOCHANGE
NOCHANGE	NOCHANGE	NOCHANGE	DOWN	NOCHANGE
DOWN	DOWN	DOWN	DOWN	DOWN
NOCHANGE	NOCHANGE	UP	NOCHANGE	NOCHANGE
UP	NOCHANGE	NOCHANGE	NOCHANGE	NOCHANGE
DOWN	DOWN	UP	DOWN	DOWN
UP	NOCHANGE	DOWN	NOCHANGE	NOCHANGE
DOWN	DOWN	NOCHANGE	NOCHANGE	NOCHANGE
NOCHANGE	UP	NOCHANGE	NOCHANGE	NOCHANGE
UP	UP	DOWN	NOCHANGE	UP
NOCHANGE	NOCHANGE	NOCHANGE	NOCHANGE	NOCHANGE
DOWN	DOWN	DOWN	NOCHANGE	NOCHANGE
DOWN	DOWN	NOCHANGE	DOWN	DOWN
NOCHANGE	DOWN	UP	NOCHANGE	NOCHANGE
NOCHANGE	NOCHANGE	NOCHANGE	NOCHANGE	NOCHANGE
NOCHANGE	NOCHANGE	NOCHANGE	NOCHANGE	NOCHANGE
NOCHANGE	NOCHANGE	NOCHANGE	NOCHANGE	DOWN
NOCHANGE	NOCHANGE	NOCHANGE	NOCHANGE	NOCHANGE
DOWN	UP	UP	DOWN	DOWN

TCGA-B0-5711-01.	TCGA-CZ-5451-01	TCGA-BP-4340-01.	TCGA-BP-5180-01.	TCGA-CJ-4639-01/
NOCHANGE	NOCHANGE	NOCHANGE	NOCHANGE	NOCHANGE
UP	UP	UP	UP	UP
NOCHANGE	NOCHANGE	NOCHANGE	NOCHANGE	NOCHANGE
DOWN	DOWN	NOCHANGE	DOWN	DOWN
NOCHANGE	NOCHANGE	NOCHANGE	NOCHANGE	NOCHANGE
NOCHANGE	NOCHANGE	NOCHANGE	NOCHANGE	NOCHANGE
DOWN	DOWN	DOWN	DOWN	NOCHANGE
DOWN	NOCHANGE	DOWN	NOCHANGE	DOWN
DOWN	UP	NOCHANGE	NOCHANGE	NOCHANGE
NOCHANGE	NOCHANGE	NOCHANGE	NOCHANGE	NOCHANGE
NOCHANGE	NOCHANGE	NOCHANGE	NOCHANGE	NOCHANGE
DOWN	DOWN	NOCHANGE	DOWN	NOCHANGE
NOCHANGE	NOCHANGE	NOCHANGE	NOCHANGE	NOCHANGE
NOCHANGE	NOCHANGE	NOCHANGE	DOWN	NOCHANGE
DOWN	DOWN	DOWN	DOWN	DOWN
NOCHANGE	NOCHANGE	UP	NOCHANGE	UP
NOCHANGE	NOCHANGE	NOCHANGE	NOCHANGE	NOCHANGE
NOCHANGE	NOCHANGE	DOWN	NOCHANGE	DOWN
UP	NOCHANGE	UP	NOCHANGE	UP
NOCHANGE	NOCHANGE	DOWN	NOCHANGE	DOWN
NOCHANGE	NOCHANGE	NOCHANGE	NOCHANGE	NOCHANGE
NOCHANGE	UP	UP	NOCHANGE	NOCHANGE
NOCHANGE	NOCHANGE	DOWN	NOCHANGE	NOCHANGE
DOWN	NOCHANGE	NOCHANGE	NOCHANGE	DOWN
DOWN	NOCHANGE	NOCHANGE	DOWN	NOCHANGE
NOCHANGE	NOCHANGE	DOWN	NOCHANGE	DOWN
NOCHANGE	NOCHANGE	NOCHANGE	NOCHANGE	NOCHANGE
NOCHANGE	NOCHANGE	NOCHANGE	NOCHANGE	NOCHANGE
NOCHANGE	NOCHANGE	NOCHANGE	NOCHANGE	NOCHANGE
NOCHANGE	NOCHANGE	NOCHANGE	NOCHANGE	NOCHANGE
NOCHANGE	DOWN	DOWN	NOCHANGE	DOWN

TCGA-B0-4821-01.	TCGA-CZ-4860-01	TCGA-B0-4813-01.	TCGA-B8-4619-01.	TCGA-CJ-4892-01/
NOCHANGE	UP	NOCHANGE	NOCHANGE	NOCHANGE
UP	UP	NOCHANGE	NOCHANGE	UP
NOCHANGE	NOCHANGE	NOCHANGE	DOWN	NOCHANGE
DOWN	DOWN	DOWN	NOCHANGE	DOWN
NOCHANGE	UP	NOCHANGE	NOCHANGE	NOCHANGE
NOCHANGE	UP	DOWN	NOCHANGE	NOCHANGE
DOWN	DOWN	NOCHANGE	NOCHANGE	NOCHANGE
UP	NOCHANGE	NOCHANGE	NOCHANGE	NOCHANGE
UP	NOCHANGE	NOCHANGE	NOCHANGE	NOCHANGE
NOCHANGE	NOCHANGE	NOCHANGE	NOCHANGE	NOCHANGE
NOCHANGE	DOWN	UP	DOWN	NOCHANGE
NOCHANGE	DOWN	NOCHANGE	NOCHANGE	DOWN
NOCHANGE	NOCHANGE	NOCHANGE	NOCHANGE	NOCHANGE
NOCHANGE	UP	DOWN	NOCHANGE	NOCHANGE
DOWN	DOWN	DOWN	DOWN	NOCHANGE
NOCHANGE	NOCHANGE	DOWN	UP	NOCHANGE
DOWN	NOCHANGE	DOWN	NOCHANGE	NOCHANGE
DOWN	NOCHANGE	NOCHANGE	DOWN	NOCHANGE
NOCHANGE	NOCHANGE	NOCHANGE	NOCHANGE	NOCHANGE
DOWN	DOWN	DOWN	DOWN	DOWN
NOCHANGE	UP	NOCHANGE	NOCHANGE	NOCHANGE
NOCHANGE	NOCHANGE	UP	NOCHANGE	UP
DOWN	UP	DOWN	NOCHANGE	DOWN
NOCHANGE	NOCHANGE	NOCHANGE	NOCHANGE	NOCHANGE
DOWN	DOWN	DOWN	NOCHANGE	NOCHANGE
NOCHANGE	UP	DOWN	DOWN	DOWN
NOCHANGE	NOCHANGE	NOCHANGE	NOCHANGE	NOCHANGE
NOCHANGE	NOCHANGE	NOCHANGE	NOCHANGE	NOCHANGE
DOWN	NOCHANGE	DOWN	NOCHANGE	DOWN
NOCHANGE	DOWN	NOCHANGE	NOCHANGE	NOCHANGE
NOCHANGE	NOCHANGE	DOWN	NOCHANGE	DOWN

TCGA-CJ-4916-01/	TCGA-BP-4964-01.	TCGA-B0-5712-01.	TCGA-BP-4965-01.	TCGA-BP-5199-01.
NOCHANGE	NOCHANGE	UP	NOCHANGE	NOCHANGE
UP	UP	UP	UP	UP
NOCHANGE	NOCHANGE	NOCHANGE	NOCHANGE	NOCHANGE
DOWN	DOWN	DOWN	DOWN	DOWN
NOCHANGE	NOCHANGE	NOCHANGE	NOCHANGE	NOCHANGE
NOCHANGE	NOCHANGE	UP	NOCHANGE	DOWN
NOCHANGE	NOCHANGE	DOWN	DOWN	DOWN
DOWN	NOCHANGE	NOCHANGE	DOWN	NOCHANGE
DOWN	UP	UP	NOCHANGE	NOCHANGE
NOCHANGE	NOCHANGE	NOCHANGE	NOCHANGE	NOCHANGE
NOCHANGE	NOCHANGE	NOCHANGE	NOCHANGE	NOCHANGE
NOCHANGE	DOWN	DOWN	NOCHANGE	NOCHANGE
NOCHANGE	NOCHANGE	NOCHANGE	NOCHANGE	DOWN
DOWN	NOCHANGE	NOCHANGE	NOCHANGE	NOCHANGE
DOWN	DOWN	NOCHANGE	DOWN	NOCHANGE
NOCHANGE	NOCHANGE	NOCHANGE	NOCHANGE	NOCHANGE
NOCHANGE	NOCHANGE	NOCHANGE	NOCHANGE	NOCHANGE
NOCHANGE	DOWN	NOCHANGE	DOWN	NOCHANGE
NOCHANGE	UP	NOCHANGE	UP	NOCHANGE
DOWN	DOWN	DOWN	NOCHANGE	DOWN
NOCHANGE	NOCHANGE	NOCHANGE	UP	NOCHANGE
NOCHANGE	NOCHANGE	NOCHANGE	NOCHANGE	UP
DOWN	NOCHANGE	NOCHANGE	NOCHANGE	DOWN
DOWN	NOCHANGE	NOCHANGE	DOWN	DOWN
DOWN	DOWN	NOCHANGE	NOCHANGE	DOWN
DOWN	NOCHANGE	NOCHANGE	NOCHANGE	NOCHANGE
NOCHANGE	NOCHANGE	NOCHANGE	NOCHANGE	NOCHANGE
NOCHANGE	NOCHANGE	NOCHANGE	NOCHANGE	NOCHANGE
NOCHANGE	NOCHANGE	NOCHANGE	NOCHANGE	DOWN
NOCHANGE	NOCHANGE	NOCHANGE	NOCHANGE	NOCHANGE
DOWN	NOCHANGE	DOWN	DOWN	DOWN

TCGA-BP-4973-01	TCGA-B2-5635-01	TCGA-B0-4844-01	TCGA-G6-A8L7-01	TCGA-B0-5088-01
NOCHANGE	NOCHANGE	NOCHANGE	NOCHANGE	NOCHANGE
UP	UP	UP	UP	UP
NOCHANGE	NOCHANGE	NOCHANGE	NOCHANGE	NOCHANGE
DOWN	DOWN	DOWN	DOWN	DOWN
UP	UP	UP	NOCHANGE	NOCHANGE
NOCHANGE	NOCHANGE	NOCHANGE	NOCHANGE	NOCHANGE
DOWN	DOWN	DOWN	DOWN	NOCHANGE
NOCHANGE	NOCHANGE	UP	UP	NOCHANGE
NOCHANGE	NOCHANGE	NOCHANGE	NOCHANGE	NOCHANGE
NOCHANGE	NOCHANGE	NOCHANGE	NOCHANGE	NOCHANGE
DOWN	NOCHANGE	UP	UP	NOCHANGE
DOWN	DOWN	NOCHANGE	DOWN	NOCHANGE
NOCHANGE	NOCHANGE	NOCHANGE	DOWN	NOCHANGE
NOCHANGE	NOCHANGE	NOCHANGE	DOWN	NOCHANGE
DOWN	DOWN	DOWN	DOWN	DOWN
NOCHANGE	NOCHANGE	DOWN	DOWN	NOCHANGE
NOCHANGE	UP	DOWN	DOWN	NOCHANGE
DOWN	NOCHANGE	NOCHANGE	NOCHANGE	DOWN
NOCHANGE	UP	NOCHANGE	DOWN	NOCHANGE
NOCHANGE	NOCHANGE	NOCHANGE	NOCHANGE	DOWN
NOCHANGE	UP	NOCHANGE	NOCHANGE	NOCHANGE
UP	NOCHANGE	UP	DOWN	NOCHANGE
DOWN	NOCHANGE	DOWN	DOWN	DOWN
DOWN	DOWN	DOWN	NOCHANGE	NOCHANGE
DOWN	NOCHANGE	DOWN	DOWN	DOWN
DOWN	NOCHANGE	NOCHANGE	UP	NOCHANGE
NOCHANGE	NOCHANGE	NOCHANGE	NOCHANGE	NOCHANGE
NOCHANGE	NOCHANGE	NOCHANGE	NOCHANGE	NOCHANGE
NOCHANGE	NOCHANGE	DOWN	DOWN	DOWN
NOCHANGE	DOWN	NOCHANGE	NOCHANGE	NOCHANGE
DOWN	NOCHANGE	NOCHANGE	UP	NOCHANGE

TCGA-A3-3324-01	TCGA-BP-4968-01.	TCGA-BP-4352-01.	TCGA-CJ-4872-01/	TCGA-DV-A4VZ-0
NOCHANGE	NOCHANGE	NOCHANGE	NOCHANGE	NOCHANGE
UP	UP	NOCHANGE	UP	NOCHANGE
NOCHANGE	NOCHANGE	DOWN	NOCHANGE	NOCHANGE
DOWN	DOWN	DOWN	DOWN	DOWN
UP	NOCHANGE	NOCHANGE	NOCHANGE	NOCHANGE
NOCHANGE	NOCHANGE	NOCHANGE	NOCHANGE	DOWN
DOWN	DOWN	DOWN	NOCHANGE	DOWN
NOCHANGE	NOCHANGE	UP	NOCHANGE	NOCHANGE
NOCHANGE	NOCHANGE	UP	DOWN	UP
NOCHANGE	NOCHANGE	NOCHANGE	DOWN	NOCHANGE
DOWN	NOCHANGE	NOCHANGE	NOCHANGE	DOWN
DOWN	DOWN	UP	NOCHANGE	UP
NOCHANGE	NOCHANGE	DOWN	NOCHANGE	DOWN
NOCHANGE	DOWN	DOWN	NOCHANGE	DOWN
DOWN	DOWN	DOWN	NOCHANGE	NOCHANGE
UP	NOCHANGE	NOCHANGE	NOCHANGE	DOWN
NOCHANGE	NOCHANGE	DOWN	NOCHANGE	NOCHANGE
DOWN	NOCHANGE	NOCHANGE	DOWN	NOCHANGE
NOCHANGE	NOCHANGE	NOCHANGE	NOCHANGE	NOCHANGE
DOWN	DOWN	DOWN	DOWN	DOWN
UP	NOCHANGE	NOCHANGE	NOCHANGE	DOWN
NOCHANGE	NOCHANGE	NOCHANGE	NOCHANGE	NOCHANGE
NOCHANGE	DOWN	UP	NOCHANGE	DOWN
DOWN	NOCHANGE	NOCHANGE	DOWN	DOWN
DOWN	DOWN	DOWN	DOWN	DOWN
NOCHANGE	NOCHANGE	UP	NOCHANGE	DOWN
NOCHANGE	NOCHANGE	NOCHANGE	NOCHANGE	NOCHANGE
NOCHANGE	NOCHANGE	NOCHANGE	NOCHANGE	NOCHANGE
DOWN	DOWN	DOWN	NOCHANGE	DOWN
NOCHANGE	NOCHANGE	DOWN	NOCHANGE	DOWN
DOWN	DOWN	DOWN	NOCHANGE	DOWN

TCGA-BP-5006-01.	TCGA-BP-5009-01.	TCGA-B0-4699-01.	TCGA-B0-4834-01.	TCGA-BP-4998-01.
NOCHANGE	NOCHANGE	DOWN	DOWN	NOCHANGE
UP	UP	UP	NOCHANGE	UP
NOCHANGE	NOCHANGE	NOCHANGE	NOCHANGE	NOCHANGE
DOWN	DOWN	DOWN	DOWN	DOWN
NOCHANGE	UP	NOCHANGE	NOCHANGE	UP
NOCHANGE	NOCHANGE	DOWN	NOCHANGE	NOCHANGE
DOWN	DOWN	NOCHANGE	NOCHANGE	DOWN
NOCHANGE	NOCHANGE	NOCHANGE	NOCHANGE	DOWN
DOWN	NOCHANGE	NOCHANGE	NOCHANGE	UP
NOCHANGE	NOCHANGE	NOCHANGE	NOCHANGE	NOCHANGE
NOCHANGE	DOWN	NOCHANGE	DOWN	DOWN
NOCHANGE	DOWN	UP	UP	NOCHANGE
NOCHANGE	NOCHANGE	DOWN	NOCHANGE	NOCHANGE
NOCHANGE	DOWN	NOCHANGE	DOWN	NOCHANGE
DOWN	DOWN	DOWN	DOWN	NOCHANGE
NOCHANGE	NOCHANGE	NOCHANGE	UP	NOCHANGE
UP	NOCHANGE	NOCHANGE	DOWN	NOCHANGE
DOWN	DOWN	DOWN	DOWN	DOWN
NOCHANGE	NOCHANGE	NOCHANGE	NOCHANGE	UP
DOWN	DOWN	DOWN	DOWN	NOCHANGE
NOCHANGE	NOCHANGE	UP	NOCHANGE	UP
NOCHANGE	NOCHANGE	NOCHANGE	NOCHANGE	UP
NOCHANGE	NOCHANGE	NOCHANGE	NOCHANGE	NOCHANGE
NOCHANGE	DOWN	NOCHANGE	NOCHANGE	DOWN
NOCHANGE	DOWN	NOCHANGE	NOCHANGE	DOWN
NOCHANGE	DOWN	NOCHANGE	DOWN	NOCHANGE
NOCHANGE	NOCHANGE	NOCHANGE	NOCHANGE	NOCHANGE
NOCHANGE	NOCHANGE	NOCHANGE	NOCHANGE	NOCHANGE
NOCHANGE	DOWN	NOCHANGE	NOCHANGE	NOCHANGE
NOCHANGE	NOCHANGE	NOCHANGE	NOCHANGE	NOCHANGE
NOCHANGE	DOWN	DOWN	NOCHANGE	DOWN

TCGA-B0-5104-01.	TCGA-B0-4945-01.	TCGA-B0-4842-01.	TCGA-AK-3447-01	TCGA-B8-5551-01.
NOCHANGE	NOCHANGE	NOCHANGE	DOWN	NOCHANGE
UP	UP	UP	DOWN	UP
NOCHANGE	NOCHANGE	NOCHANGE	NOCHANGE	DOWN
DOWN	DOWN	DOWN	DOWN	DOWN
UP	NOCHANGE	UP	DOWN	NOCHANGE
UP	NOCHANGE	UP	DOWN	UP
NOCHANGE	NOCHANGE	DOWN	NOCHANGE	DOWN
NOCHANGE	NOCHANGE	NOCHANGE	NOCHANGE	NOCHANGE
NOCHANGE	NOCHANGE	NOCHANGE	DOWN	NOCHANGE
NOCHANGE	NOCHANGE	NOCHANGE	NOCHANGE	NOCHANGE
DOWN	NOCHANGE	UP	NOCHANGE	DOWN
NOCHANGE	NOCHANGE	DOWN	DOWN	NOCHANGE
NOCHANGE	NOCHANGE	NOCHANGE	NOCHANGE	NOCHANGE
NOCHANGE	NOCHANGE	NOCHANGE	DOWN	NOCHANGE
DOWN	DOWN	NOCHANGE	DOWN	DOWN
NOCHANGE	NOCHANGE	DOWN	NOCHANGE	NOCHANGE
UP	NOCHANGE	DOWN	DOWN	UP
DOWN	NOCHANGE	NOCHANGE	UP	DOWN
NOCHANGE	NOCHANGE	DOWN	DOWN	NOCHANGE
DOWN	NOCHANGE	DOWN	DOWN	DOWN
NOCHANGE	NOCHANGE	NOCHANGE	DOWN	UP
NOCHANGE	NOCHANGE	NOCHANGE	DOWN	NOCHANGE
NOCHANGE	NOCHANGE	DOWN	DOWN	UP
DOWN	DOWN	NOCHANGE	UP	DOWN
NOCHANGE	NOCHANGE	DOWN	UP	DOWN
NOCHANGE	NOCHANGE	DOWN	NOCHANGE	NOCHANGE
NOCHANGE	UP	NOCHANGE	NOCHANGE	NOCHANGE
NOCHANGE	NOCHANGE	NOCHANGE	DOWN	NOCHANGE
NOCHANGE	NOCHANGE	DOWN	DOWN	NOCHANGE
NOCHANGE	NOCHANGE	DOWN	DOWN	NOCHANGE
DOWN	DOWN	NOCHANGE	UP	DOWN

TCGA-A3-3349-01	TCGA-B0-4693-01.	TCGA-A3-3346-01	TCGA-B0-5120-01.	TCGA-BP-5177-01.
NOCHANGE	NOCHANGE	UP	NOCHANGE	NOCHANGE
UP	UP	UP	UP	UP
NOCHANGE	NOCHANGE	NOCHANGE	NOCHANGE	NOCHANGE
NOCHANGE	DOWN	DOWN	DOWN	NOCHANGE
NOCHANGE	NOCHANGE	NOCHANGE	NOCHANGE	DOWN
NOCHANGE	UP	UP	NOCHANGE	NOCHANGE
DOWN	DOWN	DOWN	DOWN	NOCHANGE
DOWN	NOCHANGE	NOCHANGE	NOCHANGE	NOCHANGE
DOWN	UP	UP	DOWN	DOWN
NOCHANGE	NOCHANGE	NOCHANGE	NOCHANGE	NOCHANGE
NOCHANGE	DOWN	NOCHANGE	NOCHANGE	NOCHANGE
NOCHANGE	DOWN	NOCHANGE	NOCHANGE	NOCHANGE
NOCHANGE	NOCHANGE	NOCHANGE	NOCHANGE	NOCHANGE
NOCHANGE	NOCHANGE	NOCHANGE	NOCHANGE	NOCHANGE
NOCHANGE	DOWN	DOWN	DOWN	NOCHANGE
NOCHANGE	NOCHANGE	NOCHANGE	UP	NOCHANGE
NOCHANGE	UP	NOCHANGE	NOCHANGE	NOCHANGE
DOWN	DOWN	NOCHANGE	NOCHANGE	NOCHANGE
NOCHANGE	NOCHANGE	DOWN	NOCHANGE	NOCHANGE
DOWN	NOCHANGE	DOWN	DOWN	DOWN
UP	UP	NOCHANGE	UP	NOCHANGE
UP	NOCHANGE	UP	UP	NOCHANGE
DOWN	NOCHANGE	UP	NOCHANGE	DOWN
DOWN	DOWN	NOCHANGE	NOCHANGE	DOWN
DOWN	NOCHANGE	DOWN	DOWN	DOWN
NOCHANGE	NOCHANGE	UP	NOCHANGE	NOCHANGE
NOCHANGE	NOCHANGE	NOCHANGE	NOCHANGE	UP
NOCHANGE	NOCHANGE	NOCHANGE	NOCHANGE	NOCHANGE
NOCHANGE	NOCHANGE	DOWN	NOCHANGE	NOCHANGE
NOCHANGE	NOCHANGE	NOCHANGE	NOCHANGE	NOCHANGE
DOWN	DOWN	NOCHANGE	DOWN	DOWN

TCGA-B8-4154-01	TCGA-B8-4153-01	TCGA-CJ-6030-01	TCGA-CW-5584-01	TCGA-B0-5696-01
NOCHANGE	NOCHANGE	NOCHANGE	NOCHANGE	NOCHANGE
UP	UP	UP	UP	UP
NOCHANGE	NOCHANGE	NOCHANGE	NOCHANGE	NOCHANGE
NOCHANGE	DOWN	DOWN	DOWN	DOWN
NOCHANGE	NOCHANGE	UP	NOCHANGE	UP
NOCHANGE	NOCHANGE	NOCHANGE	NOCHANGE	UP
DOWN	DOWN	NOCHANGE	NOCHANGE	NOCHANGE
NOCHANGE	DOWN	NOCHANGE	NOCHANGE	NOCHANGE
NOCHANGE	DOWN	NOCHANGE	DOWN	NOCHANGE
NOCHANGE	NOCHANGE	NOCHANGE	NOCHANGE	NOCHANGE
NOCHANGE	NOCHANGE	NOCHANGE	NOCHANGE	NOCHANGE
NOCHANGE	NOCHANGE	NOCHANGE	DOWN	DOWN
NOCHANGE	NOCHANGE	NOCHANGE	NOCHANGE	NOCHANGE
NOCHANGE	DOWN	NOCHANGE	NOCHANGE	NOCHANGE
DOWN	DOWN	DOWN	NOCHANGE	DOWN
NOCHANGE	NOCHANGE	NOCHANGE	NOCHANGE	UP
NOCHANGE	NOCHANGE	UP	UP	NOCHANGE
NOCHANGE	NOCHANGE	NOCHANGE	DOWN	UP
NOCHANGE	NOCHANGE	UP	NOCHANGE	NOCHANGE
DOWN	DOWN	DOWN	NOCHANGE	DOWN
NOCHANGE	NOCHANGE	UP	UP	NOCHANGE
NOCHANGE	UP	NOCHANGE	NOCHANGE	NOCHANGE
DOWN	DOWN	NOCHANGE	NOCHANGE	UP
NOCHANGE	NOCHANGE	NOCHANGE	NOCHANGE	NOCHANGE
NOCHANGE	DOWN	NOCHANGE	NOCHANGE	NOCHANGE
NOCHANGE	NOCHANGE	NOCHANGE	NOCHANGE	UP
NOCHANGE	NOCHANGE	NOCHANGE	NOCHANGE	NOCHANGE
NOCHANGE	NOCHANGE	NOCHANGE	NOCHANGE	NOCHANGE
NOCHANGE	NOCHANGE	NOCHANGE	NOCHANGE	NOCHANGE
NOCHANGE	NOCHANGE	NOCHANGE	NOCHANGE	NOCHANGE
NOCHANGE	DOWN	DOWN	DOWN	DOWN

TCGA-BP-4765-01.	TCGA-BP-4782-01.	TCGA-B8-5165-01.	TCGA-B2-3923-01.	TCGA-AK-3425-01
NOCHANGE	NOCHANGE	NOCHANGE	DOWN	UP
UP	UP	UP	NOCHANGE	UP
NOCHANGE	NOCHANGE	NOCHANGE	NOCHANGE	NOCHANGE
NOCHANGE	DOWN	DOWN	DOWN	DOWN
UP	NOCHANGE	NOCHANGE	NOCHANGE	UP
NOCHANGE	NOCHANGE	NOCHANGE	NOCHANGE	NOCHANGE
DOWN	DOWN	NOCHANGE	NOCHANGE	DOWN
NOCHANGE	NOCHANGE	NOCHANGE	DOWN	NOCHANGE
DOWN	UP	NOCHANGE	NOCHANGE	NOCHANGE
NOCHANGE	NOCHANGE	NOCHANGE	UP	NOCHANGE
NOCHANGE	NOCHANGE	DOWN	DOWN	DOWN
NOCHANGE	UP	NOCHANGE	UP	NOCHANGE
NOCHANGE	NOCHANGE	NOCHANGE	NOCHANGE	NOCHANGE
NOCHANGE	NOCHANGE	NOCHANGE	DOWN	NOCHANGE
DOWN	DOWN	DOWN	DOWN	NOCHANGE
NOCHANGE	UP	NOCHANGE	UP	NOCHANGE
NOCHANGE	NOCHANGE	NOCHANGE	NOCHANGE	NOCHANGE
DOWN	DOWN	DOWN	NOCHANGE	NOCHANGE
NOCHANGE	NOCHANGE	NOCHANGE	NOCHANGE	NOCHANGE
DOWN	NOCHANGE	NOCHANGE	DOWN	NOCHANGE
NOCHANGE	NOCHANGE	NOCHANGE	NOCHANGE	UP
NOCHANGE	NOCHANGE	NOCHANGE	DOWN	NOCHANGE
NOCHANGE	NOCHANGE	NOCHANGE	NOCHANGE	UP
NOCHANGE	NOCHANGE	DOWN	DOWN	NOCHANGE
NOCHANGE	NOCHANGE	NOCHANGE	UP	DOWN
NOCHANGE	NOCHANGE	NOCHANGE	DOWN	NOCHANGE
NOCHANGE	NOCHANGE	NOCHANGE	UP	NOCHANGE
NOCHANGE	NOCHANGE	NOCHANGE	NOCHANGE	NOCHANGE
NOCHANGE	DOWN	NOCHANGE	NOCHANGE	NOCHANGE
NOCHANGE	NOCHANGE	NOCHANGE	NOCHANGE	NOCHANGE
DOWN	DOWN	DOWN	UP	DOWN

TCGA-AS-3778-01	TCGA-CW-6087-01	TCGA-B0-5108-01.	TCGA-B0-5699-01.	TCGA-B0-4814-01.
NOCHANGE	NOCHANGE	NOCHANGE	NOCHANGE	NOCHANGE
UP	UP	UP	UP	UP
NOCHANGE	NOCHANGE	NOCHANGE	NOCHANGE	NOCHANGE
DOWN	DOWN	DOWN	DOWN	DOWN
NOCHANGE	UP	UP	NOCHANGE	NOCHANGE
NOCHANGE	NOCHANGE	NOCHANGE	NOCHANGE	UP
NOCHANGE	DOWN	DOWN	DOWN	DOWN
NOCHANGE	NOCHANGE	NOCHANGE	DOWN	NOCHANGE
NOCHANGE	UP	NOCHANGE	DOWN	NOCHANGE
NOCHANGE	DOWN	NOCHANGE	NOCHANGE	NOCHANGE
NOCHANGE	NOCHANGE	NOCHANGE	NOCHANGE	NOCHANGE
NOCHANGE	NOCHANGE	DOWN	DOWN	DOWN
NOCHANGE	DOWN	NOCHANGE	NOCHANGE	NOCHANGE
NOCHANGE	UP	NOCHANGE	NOCHANGE	NOCHANGE
NOCHANGE	DOWN	DOWN	NOCHANGE	DOWN
NOCHANGE	NOCHANGE	NOCHANGE	NOCHANGE	NOCHANGE
NOCHANGE	UP	NOCHANGE	UP	NOCHANGE
DOWN	DOWN	DOWN	NOCHANGE	NOCHANGE
NOCHANGE	UP	NOCHANGE	UP	UP
DOWN	DOWN	DOWN	DOWN	NOCHANGE
NOCHANGE	UP	UP	NOCHANGE	NOCHANGE
NOCHANGE	NOCHANGE	UP	NOCHANGE	NOCHANGE
DOWN	UP	NOCHANGE	NOCHANGE	NOCHANGE
NOCHANGE	DOWN	DOWN	NOCHANGE	NOCHANGE
DOWN	DOWN	DOWN	NOCHANGE	NOCHANGE
NOCHANGE	NOCHANGE	DOWN	NOCHANGE	NOCHANGE
NOCHANGE	NOCHANGE	NOCHANGE	NOCHANGE	NOCHANGE
NOCHANGE	NOCHANGE	NOCHANGE	NOCHANGE	NOCHANGE
DOWN	NOCHANGE	NOCHANGE	NOCHANGE	NOCHANGE
NOCHANGE	DOWN	NOCHANGE	DOWN	NOCHANGE
DOWN	DOWN	DOWN	NOCHANGE	DOWN

TCGA-AK-3465-01	TCGA-CJ-4918-01/	TCGA-BP-4344-01.	TCGA-B4-5835-01.	TCGA-A3-3372-01
NOCHANGE	NOCHANGE	NOCHANGE	UP	NOCHANGE
NOCHANGE	UP	UP	UP	NOCHANGE
NOCHANGE	NOCHANGE	NOCHANGE	NOCHANGE	NOCHANGE
DOWN	DOWN	NOCHANGE	DOWN	DOWN
DOWN	NOCHANGE	NOCHANGE	NOCHANGE	NOCHANGE
NOCHANGE	NOCHANGE	NOCHANGE	UP	NOCHANGE
NOCHANGE	NOCHANGE	DOWN	NOCHANGE	DOWN
NOCHANGE	NOCHANGE	NOCHANGE	NOCHANGE	NOCHANGE
NOCHANGE	NOCHANGE	NOCHANGE	NOCHANGE	NOCHANGE
UP	NOCHANGE	NOCHANGE	NOCHANGE	DOWN
DOWN	DOWN	NOCHANGE	UP	DOWN
UP	NOCHANGE	NOCHANGE	NOCHANGE	UP
NOCHANGE	NOCHANGE	NOCHANGE	NOCHANGE	NOCHANGE
DOWN	NOCHANGE	NOCHANGE	UP	NOCHANGE
DOWN	DOWN	DOWN	DOWN	DOWN
UP	NOCHANGE	NOCHANGE	UP	UP
NOCHANGE	NOCHANGE	NOCHANGE	NOCHANGE	NOCHANGE
DOWN	DOWN	DOWN	NOCHANGE	DOWN
DOWN	NOCHANGE	UP	NOCHANGE	UP
DOWN	DOWN	DOWN	NOCHANGE	NOCHANGE
NOCHANGE	UP	UP	NOCHANGE	UP
NOCHANGE	NOCHANGE	NOCHANGE	NOCHANGE	UP
DOWN	NOCHANGE	NOCHANGE	UP	NOCHANGE
UP	DOWN	DOWN	NOCHANGE	DOWN
NOCHANGE	DOWN	DOWN	DOWN	DOWN
DOWN	NOCHANGE	NOCHANGE	NOCHANGE	DOWN
NOCHANGE	NOCHANGE	NOCHANGE	NOCHANGE	NOCHANGE
DOWN	NOCHANGE	NOCHANGE	NOCHANGE	NOCHANGE
DOWN	NOCHANGE	NOCHANGE	NOCHANGE	NOCHANGE
DOWN	NOCHANGE	UP	DOWN	DOWN
NOCHANGE	DOWN	DOWN	UP	DOWN

TCGA-BP-4981-01.	TCGA-BP-4170-01.	TCGA-CJ-4643-01/	TCGA-BP-4977-01.	TCGA-B2-5633-01.
NOCHANGE	NOCHANGE	NOCHANGE	UP	NOCHANGE
UP	UP	UP	UP	UP
NOCHANGE	NOCHANGE	NOCHANGE	NOCHANGE	NOCHANGE
DOWN	DOWN	NOCHANGE	NOCHANGE	DOWN
UP	NOCHANGE	NOCHANGE	NOCHANGE	UP
NOCHANGE	UP	UP	NOCHANGE	NOCHANGE
NOCHANGE	DOWN	NOCHANGE	DOWN	DOWN
NOCHANGE	NOCHANGE	NOCHANGE	NOCHANGE	NOCHANGE
NOCHANGE	NOCHANGE	NOCHANGE	NOCHANGE	NOCHANGE
DOWN	NOCHANGE	NOCHANGE	NOCHANGE	NOCHANGE
NOCHANGE	DOWN	NOCHANGE	NOCHANGE	NOCHANGE
DOWN	DOWN	NOCHANGE	NOCHANGE	NOCHANGE
NOCHANGE	NOCHANGE	NOCHANGE	NOCHANGE	NOCHANGE
DOWN	NOCHANGE	NOCHANGE	NOCHANGE	NOCHANGE
DOWN	NOCHANGE	NOCHANGE	NOCHANGE	DOWN
DOWN	NOCHANGE	NOCHANGE	NOCHANGE	NOCHANGE
NOCHANGE	NOCHANGE	UP	NOCHANGE	NOCHANGE
NOCHANGE	NOCHANGE	DOWN	DOWN	DOWN
NOCHANGE	NOCHANGE	UP	UP	NOCHANGE
DOWN	DOWN	DOWN	NOCHANGE	DOWN
NOCHANGE	UP	UP	NOCHANGE	UP
NOCHANGE	NOCHANGE	NOCHANGE	NOCHANGE	UP
DOWN	NOCHANGE	NOCHANGE	NOCHANGE	NOCHANGE
NOCHANGE	NOCHANGE	DOWN	NOCHANGE	DOWN
DOWN	NOCHANGE	NOCHANGE	DOWN	DOWN
NOCHANGE	NOCHANGE	DOWN	NOCHANGE	DOWN
NOCHANGE	NOCHANGE	NOCHANGE	NOCHANGE	NOCHANGE
NOCHANGE	NOCHANGE	NOCHANGE	NOCHANGE	NOCHANGE
DOWN	NOCHANGE	NOCHANGE	DOWN	DOWN
NOCHANGE	NOCHANGE	NOCHANGE	DOWN	DOWN
DOWN	DOWN	DOWN	DOWN	DOWN

TCGA-A3-3311-01	TCGA-BP-5173-01.	TCGA-BP-5007-01.	TCGA-A3-3362-01	TCGA-BP-4781-01.
NOCHANGE	NOCHANGE	NOCHANGE	NOCHANGE	NOCHANGE
UP	UP	UP	UP	UP
NOCHANGE	NOCHANGE	NOCHANGE	NOCHANGE	NOCHANGE
DOWN	DOWN	NOCHANGE	NOCHANGE	DOWN
UP	NOCHANGE	NOCHANGE	NOCHANGE	NOCHANGE
NOCHANGE	NOCHANGE	NOCHANGE	UP	NOCHANGE
DOWN	DOWN	NOCHANGE	DOWN	NOCHANGE
DOWN	DOWN	NOCHANGE	NOCHANGE	NOCHANGE
NOCHANGE	NOCHANGE	NOCHANGE	NOCHANGE	NOCHANGE
NOCHANGE	NOCHANGE	NOCHANGE	NOCHANGE	DOWN
NOCHANGE	NOCHANGE	NOCHANGE	NOCHANGE	DOWN
DOWN	NOCHANGE	NOCHANGE	UP	UP
NOCHANGE	NOCHANGE	NOCHANGE	NOCHANGE	NOCHANGE
NOCHANGE	NOCHANGE	NOCHANGE	NOCHANGE	NOCHANGE
DOWN	DOWN	DOWN	DOWN	DOWN
NOCHANGE	NOCHANGE	NOCHANGE	NOCHANGE	NOCHANGE
UP	NOCHANGE	NOCHANGE	NOCHANGE	NOCHANGE
DOWN	NOCHANGE	DOWN	NOCHANGE	DOWN
UP	NOCHANGE	NOCHANGE	NOCHANGE	NOCHANGE
DOWN	DOWN	NOCHANGE	NOCHANGE	NOCHANGE
UP	NOCHANGE	NOCHANGE	NOCHANGE	UP
UP	NOCHANGE	NOCHANGE	NOCHANGE	UP
NOCHANGE	NOCHANGE	DOWN	NOCHANGE	NOCHANGE
NOCHANGE	NOCHANGE	DOWN	NOCHANGE	DOWN
NOCHANGE	DOWN	DOWN	NOCHANGE	DOWN
NOCHANGE	NOCHANGE	NOCHANGE	NOCHANGE	NOCHANGE
NOCHANGE	NOCHANGE	UP	UP	NOCHANGE
NOCHANGE	NOCHANGE	NOCHANGE	NOCHANGE	NOCHANGE
NOCHANGE	NOCHANGE	DOWN	NOCHANGE	NOCHANGE
NOCHANGE	NOCHANGE	DOWN	NOCHANGE	NOCHANGE
NOCHANGE	DOWN	DOWN	DOWN	DOWN

TCGA-A3-3365-01	TCGA-DV-A4W0-0	TCGA-B0-5092-01.	TCGA-CJ-5683-01/	TCGA-BP-4331-01.
NOCHANGE	NOCHANGE	NOCHANGE	NOCHANGE	NOCHANGE
UP	UP	UP	UP	UP
NOCHANGE	NOCHANGE	NOCHANGE	NOCHANGE	NOCHANGE
NOCHANGE	DOWN	DOWN	DOWN	DOWN
DOWN	NOCHANGE	NOCHANGE	NOCHANGE	NOCHANGE
NOCHANGE	UP	NOCHANGE	NOCHANGE	NOCHANGE
NOCHANGE	NOCHANGE	NOCHANGE	NOCHANGE	DOWN
NOCHANGE	NOCHANGE	NOCHANGE	NOCHANGE	DOWN
UP	UP	NOCHANGE	NOCHANGE	DOWN
DOWN	NOCHANGE	NOCHANGE	NOCHANGE	NOCHANGE
NOCHANGE	NOCHANGE	NOCHANGE	NOCHANGE	DOWN
NOCHANGE	NOCHANGE	NOCHANGE	NOCHANGE	NOCHANGE
NOCHANGE	NOCHANGE	NOCHANGE	NOCHANGE	NOCHANGE
NOCHANGE	NOCHANGE	NOCHANGE	NOCHANGE	NOCHANGE
DOWN	NOCHANGE	DOWN	DOWN	DOWN
UP	NOCHANGE	NOCHANGE	NOCHANGE	NOCHANGE
NOCHANGE	UP	NOCHANGE	NOCHANGE	NOCHANGE
DOWN	UP	NOCHANGE	NOCHANGE	DOWN
NOCHANGE	NOCHANGE	NOCHANGE	NOCHANGE	NOCHANGE
DOWN	DOWN	DOWN	NOCHANGE	DOWN
NOCHANGE	NOCHANGE	NOCHANGE	NOCHANGE	UP
NOCHANGE	NOCHANGE	UP	NOCHANGE	NOCHANGE
NOCHANGE	UP	DOWN	NOCHANGE	NOCHANGE
DOWN	DOWN	DOWN	NOCHANGE	NOCHANGE
DOWN	DOWN	DOWN	NOCHANGE	NOCHANGE
NOCHANGE	NOCHANGE	NOCHANGE	NOCHANGE	NOCHANGE
NOCHANGE	NOCHANGE	NOCHANGE	NOCHANGE	NOCHANGE
NOCHANGE	NOCHANGE	NOCHANGE	NOCHANGE	NOCHANGE
NOCHANGE	UP	DOWN	NOCHANGE	NOCHANGE
NOCHANGE	NOCHANGE	NOCHANGE	NOCHANGE	NOCHANGE
DOWN	NOCHANGE	DOWN	DOWN	DOWN

TCGA-B2-A4SR-01	TCGA-BP-4976-01.	TCGA-B2-4099-01.	TCGA-B2-3923-01.	TCGA-G6-A5PC-01
NOCHANGE	NOCHANGE	NOCHANGE	DOWN	NOCHANGE
UP	UP	UP	NOCHANGE	UP
NOCHANGE	NOCHANGE	NOCHANGE	NOCHANGE	NOCHANGE
DOWN	DOWN	DOWN	NOCHANGE	DOWN
NOCHANGE	NOCHANGE	NOCHANGE	NOCHANGE	UP
NOCHANGE	NOCHANGE	NOCHANGE	DOWN	NOCHANGE
NOCHANGE	DOWN	NOCHANGE	NOCHANGE	DOWN
NOCHANGE	DOWN	NOCHANGE	DOWN	NOCHANGE
NOCHANGE	DOWN	DOWN	NOCHANGE	NOCHANGE
NOCHANGE	NOCHANGE	NOCHANGE	NOCHANGE	UP
NOCHANGE	NOCHANGE	NOCHANGE	DOWN	UP
UP	NOCHANGE	DOWN	UP	NOCHANGE
NOCHANGE	NOCHANGE	NOCHANGE	NOCHANGE	NOCHANGE
NOCHANGE	NOCHANGE	NOCHANGE	NOCHANGE	DOWN
DOWN	DOWN	DOWN	DOWN	NOCHANGE
NOCHANGE	NOCHANGE	NOCHANGE	UP	DOWN
UP	NOCHANGE	NOCHANGE	NOCHANGE	DOWN
DOWN	NOCHANGE	NOCHANGE	DOWN	UP
NOCHANGE	NOCHANGE	NOCHANGE	UP	NOCHANGE
NOCHANGE	NOCHANGE	DOWN	DOWN	DOWN
NOCHANGE	NOCHANGE	UP	NOCHANGE	NOCHANGE
UP	NOCHANGE	NOCHANGE	NOCHANGE	NOCHANGE
NOCHANGE	NOCHANGE	NOCHANGE	NOCHANGE	DOWN
DOWN	NOCHANGE	NOCHANGE	DOWN	UP
DOWN	NOCHANGE	NOCHANGE	NOCHANGE	DOWN
NOCHANGE	DOWN	NOCHANGE	DOWN	DOWN
NOCHANGE	NOCHANGE	NOCHANGE	NOCHANGE	NOCHANGE
NOCHANGE	NOCHANGE	NOCHANGE	NOCHANGE	NOCHANGE
NOCHANGE	DOWN	NOCHANGE	NOCHANGE	DOWN
NOCHANGE	DOWN	NOCHANGE	NOCHANGE	DOWN
DOWN	DOWN	NOCHANGE	NOCHANGE	NOCHANGE

TCGA-B0-5812-01.	TCGA-BP-4341-01.	TCGA-BP-4795-01.	TCGA-AK-3451-01	TCGA-CZ-5987-01
NOCHANGE	NOCHANGE	DOWN	UP	NOCHANGE
UP	UP	NOCHANGE	UP	UP
NOCHANGE	NOCHANGE	NOCHANGE	NOCHANGE	NOCHANGE
DOWN	DOWN	DOWN	DOWN	DOWN
NOCHANGE	NOCHANGE	DOWN	UP	UP
NOCHANGE	NOCHANGE	DOWN	NOCHANGE	NOCHANGE
NOCHANGE	NOCHANGE	NOCHANGE	NOCHANGE	DOWN
NOCHANGE	NOCHANGE	NOCHANGE	NOCHANGE	NOCHANGE
UP	NOCHANGE	UP	NOCHANGE	NOCHANGE
NOCHANGE	NOCHANGE	NOCHANGE	NOCHANGE	NOCHANGE
NOCHANGE	NOCHANGE	DOWN	UP	NOCHANGE
DOWN	UP	NOCHANGE	DOWN	NOCHANGE
NOCHANGE	NOCHANGE	NOCHANGE	NOCHANGE	NOCHANGE
NOCHANGE	NOCHANGE	NOCHANGE	NOCHANGE	DOWN
DOWN	DOWN	NOCHANGE	DOWN	DOWN
NOCHANGE	NOCHANGE	NOCHANGE	NOCHANGE	DOWN
NOCHANGE	NOCHANGE	NOCHANGE	DOWN	NOCHANGE
DOWN	NOCHANGE	DOWN	UP	NOCHANGE
NOCHANGE	NOCHANGE	NOCHANGE	DOWN	UP
DOWN	DOWN	DOWN	DOWN	NOCHANGE
NOCHANGE	NOCHANGE	NOCHANGE	NOCHANGE	NOCHANGE
UP	NOCHANGE	UP	DOWN	NOCHANGE
NOCHANGE	DOWN	NOCHANGE	DOWN	NOCHANGE
DOWN	NOCHANGE	DOWN	UP	NOCHANGE
NOCHANGE	DOWN	DOWN	NOCHANGE	DOWN
NOCHANGE	NOCHANGE	NOCHANGE	UP	NOCHANGE
NOCHANGE	NOCHANGE	NOCHANGE	NOCHANGE	NOCHANGE
NOCHANGE	NOCHANGE	NOCHANGE	DOWN	NOCHANGE
NOCHANGE	NOCHANGE	NOCHANGE	DOWN	NOCHANGE
NOCHANGE	NOCHANGE	NOCHANGE	DOWN	DOWN
DOWN	DOWN	DOWN	UP	DOWN

TCGA-MM-A564-0	TCGA-BP-4787-01.	TCGA-DV-5566-01	TCGA-BP-5190-01.	TCGA-BP-4162-01.
NOCHANGE	UP	NOCHANGE	NOCHANGE	NOCHANGE
UP	UP	UP	UP	UP
NOCHANGE	NOCHANGE	NOCHANGE	NOCHANGE	NOCHANGE
DOWN	DOWN	DOWN	DOWN	DOWN
DOWN	UP	NOCHANGE	UP	NOCHANGE
NOCHANGE	NOCHANGE	NOCHANGE	UP	UP
DOWN	DOWN	DOWN	DOWN	DOWN
NOCHANGE	UP	NOCHANGE	NOCHANGE	NOCHANGE
NOCHANGE	NOCHANGE	DOWN	NOCHANGE	NOCHANGE
NOCHANGE	NOCHANGE	NOCHANGE	NOCHANGE	NOCHANGE
UP	NOCHANGE	NOCHANGE	UP	DOWN
NOCHANGE	UP	NOCHANGE	DOWN	DOWN
NOCHANGE	NOCHANGE	NOCHANGE	NOCHANGE	NOCHANGE
DOWN	NOCHANGE	NOCHANGE	DOWN	NOCHANGE
DOWN	NOCHANGE	NOCHANGE	DOWN	DOWN
NOCHANGE	NOCHANGE	NOCHANGE	NOCHANGE	NOCHANGE
NOCHANGE	NOCHANGE	UP	NOCHANGE	NOCHANGE
NOCHANGE	DOWN	NOCHANGE	UP	DOWN
NOCHANGE	UP	NOCHANGE	NOCHANGE	NOCHANGE
DOWN	NOCHANGE	DOWN	NOCHANGE	NOCHANGE
NOCHANGE	UP	NOCHANGE	NOCHANGE	UP
NOCHANGE	NOCHANGE	UP	NOCHANGE	UP
DOWN	NOCHANGE	DOWN	UP	NOCHANGE
NOCHANGE	DOWN	NOCHANGE	NOCHANGE	DOWN
DOWN	DOWN	NOCHANGE	NOCHANGE	NOCHANGE
DOWN	NOCHANGE	NOCHANGE	UP	NOCHANGE
NOCHANGE	NOCHANGE	NOCHANGE	NOCHANGE	NOCHANGE
NOCHANGE	NOCHANGE	UP	DOWN	NOCHANGE
DOWN	DOWN	NOCHANGE	DOWN	NOCHANGE
NOCHANGE	NOCHANGE	UP	DOWN	NOCHANGE
DOWN	DOWN	NOCHANGE	NOCHANGE	DOWN

TCGA-BP-4343-01.	TCGA-AK-3458-01	TCGA-CZ-5457-01	TCGA-A3-A8OX-0	TCGA-B0-4817-01.
NOCHANGE	NOCHANGE	NOCHANGE	NOCHANGE	UP
UP	UP	UP	UP	UP
NOCHANGE	NOCHANGE	NOCHANGE	NOCHANGE	NOCHANGE
DOWN	DOWN	DOWN	NOCHANGE	NOCHANGE
NOCHANGE	NOCHANGE	UP	UP	UP
NOCHANGE	NOCHANGE	NOCHANGE	UP	UP
DOWN	NOCHANGE	DOWN	NOCHANGE	NOCHANGE
NOCHANGE	NOCHANGE	NOCHANGE	NOCHANGE	NOCHANGE
NOCHANGE	NOCHANGE	NOCHANGE	DOWN	UP
DOWN	NOCHANGE	DOWN	NOCHANGE	NOCHANGE
NOCHANGE	UP	NOCHANGE	NOCHANGE	UP
DOWN	NOCHANGE	NOCHANGE	NOCHANGE	NOCHANGE
NOCHANGE	NOCHANGE	NOCHANGE	NOCHANGE	NOCHANGE
NOCHANGE	NOCHANGE	NOCHANGE	NOCHANGE	NOCHANGE
DOWN	DOWN	DOWN	NOCHANGE	NOCHANGE
NOCHANGE	NOCHANGE	NOCHANGE	NOCHANGE	NOCHANGE
NOCHANGE	DOWN	NOCHANGE	NOCHANGE	DOWN
NOCHANGE	NOCHANGE	DOWN	NOCHANGE	UP
NOCHANGE	NOCHANGE	NOCHANGE	NOCHANGE	NOCHANGE
DOWN	DOWN	NOCHANGE	NOCHANGE	NOCHANGE
UP	NOCHANGE	NOCHANGE	NOCHANGE	NOCHANGE
NOCHANGE	NOCHANGE	NOCHANGE	UP	NOCHANGE
NOCHANGE	DOWN	NOCHANGE	NOCHANGE	UP
NOCHANGE	NOCHANGE	DOWN	DOWN	DOWN
NOCHANGE	NOCHANGE	NOCHANGE	NOCHANGE	NOCHANGE
DOWN	NOCHANGE	NOCHANGE	NOCHANGE	NOCHANGE
NOCHANGE	UP	NOCHANGE	NOCHANGE	NOCHANGE
NOCHANGE	NOCHANGE	NOCHANGE	NOCHANGE	NOCHANGE
NOCHANGE	DOWN	NOCHANGE	NOCHANGE	DOWN
NOCHANGE	DOWN	NOCHANGE	NOCHANGE	NOCHANGE
DOWN	NOCHANGE	DOWN	NOCHANGE	NOCHANGE

TCGA-CJ-4888-01[illegible]	TCGA-BP-5191-01[illegible]	TCGA-B2-5635-01[illegible]	TCGA-T7-A92I-01[illegible]	TCGA-CJ-4901-01[illegible]
NOCHANGE	NOCHANGE	NOCHANGE	DOWN	NOCHANGE
UP	UP	UP	NOCHANGE	UP
NOCHANGE	NOCHANGE	NOCHANGE	NOCHANGE	NOCHANGE
DOWN	DOWN	DOWN	NOCHANGE	DOWN
UP	NOCHANGE	UP	NOCHANGE	NOCHANGE
NOCHANGE	NOCHANGE	NOCHANGE	UP	NOCHANGE
DOWN	NOCHANGE	DOWN	NOCHANGE	NOCHANGE
NOCHANGE	NOCHANGE	NOCHANGE	DOWN	NOCHANGE
NOCHANGE	DOWN	DOWN	DOWN	NOCHANGE
NOCHANGE	NOCHANGE	NOCHANGE	NOCHANGE	NOCHANGE
NOCHANGE	UP	NOCHANGE	NOCHANGE	DOWN
DOWN	NOCHANGE	NOCHANGE	UP	NOCHANGE
NOCHANGE	NOCHANGE	NOCHANGE	NOCHANGE	NOCHANGE
NOCHANGE	DOWN	NOCHANGE	NOCHANGE	DOWN
DOWN	DOWN	DOWN	NOCHANGE	DOWN
UP	DOWN	NOCHANGE	NOCHANGE	NOCHANGE
NOCHANGE	DOWN	NOCHANGE	NOCHANGE	NOCHANGE
NOCHANGE	NOCHANGE	DOWN	NOCHANGE	DOWN
NOCHANGE	NOCHANGE	NOCHANGE	NOCHANGE	NOCHANGE
NOCHANGE	DOWN	NOCHANGE	NOCHANGE	DOWN
UP	NOCHANGE	UP	NOCHANGE	UP
NOCHANGE	NOCHANGE	UP	NOCHANGE	NOCHANGE
UP	DOWN	DOWN	NOCHANGE	NOCHANGE
DOWN	DOWN	DOWN	NOCHANGE	DOWN
DOWN	DOWN	DOWN	NOCHANGE	DOWN
NOCHANGE	NOCHANGE	DOWN	NOCHANGE	DOWN
NOCHANGE	NOCHANGE	NOCHANGE	NOCHANGE	UP
NOCHANGE	NOCHANGE	NOCHANGE	NOCHANGE	NOCHANGE
NOCHANGE	DOWN	NOCHANGE	NOCHANGE	NOCHANGE
NOCHANGE	NOCHANGE	NOCHANGE	NOCHANGE	NOCHANGE
NOCHANGE	NOCHANGE	DOWN	NOCHANGE	DOWN

TCGA-G6-A8L8-01	TCGA-BP-4173-01.	TCGA-AK-3434-01	TCGA-BP-4329-01.	TCGA-CZ-5462-01
NOCHANGE	NOCHANGE	NOCHANGE	NOCHANGE	UP
UP	UP	UP	UP	UP
NOCHANGE	NOCHANGE	NOCHANGE	NOCHANGE	NOCHANGE
NOCHANGE	DOWN	DOWN	NOCHANGE	NOCHANGE
DOWN	UP	NOCHANGE	NOCHANGE	UP
NOCHANGE	NOCHANGE	NOCHANGE	NOCHANGE	NOCHANGE
DOWN	DOWN	NOCHANGE	DOWN	NOCHANGE
DOWN	NOCHANGE	NOCHANGE	NOCHANGE	DOWN
DOWN	NOCHANGE	NOCHANGE	DOWN	NOCHANGE
NOCHANGE	NOCHANGE	NOCHANGE	DOWN	NOCHANGE
NOCHANGE	DOWN	NOCHANGE	NOCHANGE	NOCHANGE
NOCHANGE	DOWN	NOCHANGE	DOWN	DOWN
NOCHANGE	NOCHANGE	NOCHANGE	NOCHANGE	NOCHANGE
DOWN	NOCHANGE	DOWN	NOCHANGE	UP
DOWN	NOCHANGE	DOWN	DOWN	DOWN
NOCHANGE	NOCHANGE	NOCHANGE	NOCHANGE	NOCHANGE
NOCHANGE	UP	NOCHANGE	NOCHANGE	NOCHANGE
UP	DOWN	NOCHANGE	NOCHANGE	UP
NOCHANGE	NOCHANGE	NOCHANGE	NOCHANGE	NOCHANGE
NOCHANGE	DOWN	NOCHANGE	NOCHANGE	UP
NOCHANGE	UP	NOCHANGE	NOCHANGE	UP
NOCHANGE	NOCHANGE	NOCHANGE	NOCHANGE	NOCHANGE
DOWN	NOCHANGE	DOWN	NOCHANGE	UP
NOCHANGE	DOWN	NOCHANGE	NOCHANGE	DOWN
DOWN	DOWN	DOWN	NOCHANGE	NOCHANGE
DOWN	DOWN	NOCHANGE	NOCHANGE	NOCHANGE
NOCHANGE	NOCHANGE	NOCHANGE	NOCHANGE	NOCHANGE
NOCHANGE	NOCHANGE	NOCHANGE	NOCHANGE	NOCHANGE
NOCHANGE	NOCHANGE	NOCHANGE	NOCHANGE	NOCHANGE
NOCHANGE	NOCHANGE	NOCHANGE	NOCHANGE	NOCHANGE
DOWN	DOWN	DOWN	DOWN	DOWN

TCGA-B0-4706-01.	TCGA-CJ-4920-01/	TCGA-B8-A54I-01.	TCGA-CJ-4893-01/	TCGA-BP-4960-01.
NOCHANGE	NOCHANGE	NOCHANGE	NOCHANGE	NOCHANGE
UP	UP	UP	UP	UP
NOCHANGE	NOCHANGE	NOCHANGE	NOCHANGE	NOCHANGE
DOWN	DOWN	DOWN	DOWN	DOWN
NOCHANGE	NOCHANGE	UP	NOCHANGE	UP
NOCHANGE	UP	NOCHANGE	NOCHANGE	NOCHANGE
NOCHANGE	NOCHANGE	DOWN	DOWN	DOWN
NOCHANGE	NOCHANGE	NOCHANGE	DOWN	NOCHANGE
DOWN	NOCHANGE	UP	NOCHANGE	NOCHANGE
NOCHANGE	NOCHANGE	NOCHANGE	NOCHANGE	NOCHANGE
NOCHANGE	DOWN	DOWN	DOWN	NOCHANGE
NOCHANGE	UP	UP	NOCHANGE	NOCHANGE
NOCHANGE	NOCHANGE	NOCHANGE	NOCHANGE	NOCHANGE
NOCHANGE	NOCHANGE	DOWN	NOCHANGE	DOWN
NOCHANGE	DOWN	DOWN	DOWN	DOWN
NOCHANGE	NOCHANGE	DOWN	UP	DOWN
NOCHANGE	NOCHANGE	NOCHANGE	NOCHANGE	NOCHANGE
NOCHANGE	DOWN	NOCHANGE	NOCHANGE	DOWN
NOCHANGE	UP	NOCHANGE	NOCHANGE	NOCHANGE
DOWN	DOWN	NOCHANGE	NOCHANGE	NOCHANGE
NOCHANGE	NOCHANGE	NOCHANGE	UP	NOCHANGE
UP	UP	NOCHANGE	NOCHANGE	NOCHANGE
UP	NOCHANGE	NOCHANGE	NOCHANGE	DOWN
NOCHANGE	NOCHANGE	DOWN	NOCHANGE	DOWN
DOWN	DOWN	DOWN	NOCHANGE	DOWN
NOCHANGE	NOCHANGE	NOCHANGE	NOCHANGE	DOWN
NOCHANGE	NOCHANGE	NOCHANGE	NOCHANGE	NOCHANGE
NOCHANGE	NOCHANGE	NOCHANGE	NOCHANGE	NOCHANGE
NOCHANGE	NOCHANGE	DOWN	NOCHANGE	DOWN
NOCHANGE	NOCHANGE	NOCHANGE	NOCHANGE	NOCHANGE
DOWN	DOWN	DOWN	NOCHANGE	DOWN

TCGA-B2-3924-01.	TCGA-B2-3923-01.	TCGA-BP-5185-01.	TCGA-A3-3357-01	TCGA-A3-A8CQ-0
NOCHANGE	DOWN	NOCHANGE	UP	NOCHANGE
UP	NOCHANGE	UP	UP	UP
NOCHANGE	NOCHANGE	NOCHANGE	NOCHANGE	NOCHANGE
DOWN	DOWN	DOWN	DOWN	DOWN
NOCHANGE	NOCHANGE	DOWN	UP	DOWN
NOCHANGE	NOCHANGE	DOWN	NOCHANGE	UP
DOWN	NOCHANGE	DOWN	DOWN	DOWN
NOCHANGE	DOWN	NOCHANGE	NOCHANGE	NOCHANGE
DOWN	NOCHANGE	DOWN	UP	DOWN
NOCHANGE	UP	NOCHANGE	NOCHANGE	NOCHANGE
NOCHANGE	DOWN	UP	NOCHANGE	NOCHANGE
NOCHANGE	UP	DOWN	UP	UP
NOCHANGE	NOCHANGE	NOCHANGE	NOCHANGE	NOCHANGE
NOCHANGE	DOWN	DOWN	NOCHANGE	NOCHANGE
DOWN	DOWN	DOWN	DOWN	NOCHANGE
NOCHANGE	NOCHANGE	DOWN	NOCHANGE	NOCHANGE
NOCHANGE	NOCHANGE	DOWN	NOCHANGE	UP
NOCHANGE	DOWN	NOCHANGE	DOWN	DOWN
NOCHANGE	NOCHANGE	DOWN	NOCHANGE	NOCHANGE
NOCHANGE	DOWN	DOWN	DOWN	DOWN
UP	NOCHANGE	NOCHANGE	UP	NOCHANGE
NOCHANGE	NOCHANGE	NOCHANGE	NOCHANGE	NOCHANGE
NOCHANGE	NOCHANGE	DOWN	NOCHANGE	NOCHANGE
NOCHANGE	NOCHANGE	NOCHANGE	DOWN	DOWN
NOCHANGE	NOCHANGE	DOWN	NOCHANGE	NOCHANGE
NOCHANGE	DOWN	DOWN	DOWN	DOWN
NOCHANGE	NOCHANGE	NOCHANGE	NOCHANGE	NOCHANGE
NOCHANGE	NOCHANGE	NOCHANGE	NOCHANGE	NOCHANGE
NOCHANGE	NOCHANGE	DOWN	NOCHANGE	NOCHANGE
NOCHANGE	NOCHANGE	NOCHANGE	NOCHANGE	NOCHANGE
DOWN	UP	DOWN	NOCHANGE	DOWN

TCGA-BP-5176-01.	TCGA-BP-4160-01.	TCGA-B2-3924-01.	TCGA-B0-4822-01.	TCGA-A3-3328-01
NOCHANGE	NOCHANGE	NOCHANGE	NOCHANGE	DOWN
UP	UP	UP	UP	NOCHANGE
NOCHANGE	NOCHANGE	NOCHANGE	NOCHANGE	NOCHANGE
DOWN	DOWN	DOWN	DOWN	DOWN
NOCHANGE	NOCHANGE	UP	NOCHANGE	NOCHANGE
UP	NOCHANGE	NOCHANGE	NOCHANGE	NOCHANGE
DOWN	DOWN	NOCHANGE	NOCHANGE	DOWN
NOCHANGE	NOCHANGE	NOCHANGE	NOCHANGE	DOWN
DOWN	NOCHANGE	NOCHANGE	NOCHANGE	DOWN
NOCHANGE	NOCHANGE	NOCHANGE	NOCHANGE	UP
NOCHANGE	DOWN	DOWN	UP	NOCHANGE
NOCHANGE	NOCHANGE	NOCHANGE	NOCHANGE	UP
NOCHANGE	NOCHANGE	NOCHANGE	NOCHANGE	NOCHANGE
NOCHANGE	NOCHANGE	NOCHANGE	DOWN	NOCHANGE
DOWN	DOWN	DOWN	NOCHANGE	DOWN
NOCHANGE	NOCHANGE	UP	DOWN	UP
NOCHANGE	NOCHANGE	UP	DOWN	NOCHANGE
NOCHANGE	DOWN	NOCHANGE	DOWN	DOWN
NOCHANGE	NOCHANGE	NOCHANGE	NOCHANGE	DOWN
DOWN	DOWN	NOCHANGE	DOWN	DOWN
NOCHANGE	UP	UP	NOCHANGE	NOCHANGE
NOCHANGE	NOCHANGE	NOCHANGE	NOCHANGE	NOCHANGE
DOWN	NOCHANGE	NOCHANGE	DOWN	DOWN
NOCHANGE	NOCHANGE	DOWN	NOCHANGE	UP
NOCHANGE	NOCHANGE	NOCHANGE	DOWN	NOCHANGE
NOCHANGE	NOCHANGE	NOCHANGE	NOCHANGE	DOWN
NOCHANGE	NOCHANGE	NOCHANGE	NOCHANGE	NOCHANGE
NOCHANGE	NOCHANGE	NOCHANGE	NOCHANGE	NOCHANGE
NOCHANGE	NOCHANGE	NOCHANGE	DOWN	NOCHANGE
NOCHANGE	NOCHANGE	NOCHANGE	NOCHANGE	NOCHANGE
DOWN	DOWN	NOCHANGE	DOWN	NOCHANGE

TCGA-CW-5583-01	TCGA-B8-4148-01.	TCGA-BP-4959-01.	TCGA-B0-5097-01.	TCGA-CW-6090-01
NOCHANGE	NOCHANGE	NOCHANGE	NOCHANGE	NOCHANGE
UP	UP	UP	UP	UP
NOCHANGE	NOCHANGE	NOCHANGE	NOCHANGE	NOCHANGE
DOWN	DOWN	DOWN	DOWN	DOWN
NOCHANGE	NOCHANGE	UP	UP	NOCHANGE
NOCHANGE	NOCHANGE	NOCHANGE	UP	DOWN
NOCHANGE	DOWN	NOCHANGE	DOWN	DOWN
NOCHANGE	NOCHANGE	DOWN	NOCHANGE	NOCHANGE
NOCHANGE	NOCHANGE	UP	UP	NOCHANGE
NOCHANGE	NOCHANGE	NOCHANGE	DOWN	DOWN
NOCHANGE	NOCHANGE	DOWN	DOWN	NOCHANGE
NOCHANGE	NOCHANGE	NOCHANGE	NOCHANGE	DOWN
NOCHANGE	NOCHANGE	NOCHANGE	NOCHANGE	NOCHANGE
NOCHANGE	NOCHANGE	NOCHANGE	UP	NOCHANGE
NOCHANGE	DOWN	DOWN	DOWN	DOWN
NOCHANGE	NOCHANGE	NOCHANGE	NOCHANGE	NOCHANGE
NOCHANGE	UP	NOCHANGE	NOCHANGE	NOCHANGE
DOWN	DOWN	DOWN	DOWN	NOCHANGE
UP	NOCHANGE	NOCHANGE	NOCHANGE	NOCHANGE
NOCHANGE	DOWN	NOCHANGE	DOWN	DOWN
NOCHANGE	UP	UP	UP	UP
UP	UP	NOCHANGE	NOCHANGE	NOCHANGE
NOCHANGE	DOWN	NOCHANGE	UP	NOCHANGE
DOWN	DOWN	NOCHANGE	DOWN	NOCHANGE
NOCHANGE	NOCHANGE	NOCHANGE	DOWN	DOWN
NOCHANGE	DOWN	NOCHANGE	NOCHANGE	NOCHANGE
NOCHANGE	NOCHANGE	NOCHANGE	NOCHANGE	NOCHANGE
NOCHANGE	NOCHANGE	NOCHANGE	NOCHANGE	NOCHANGE
NOCHANGE	NOCHANGE	DOWN	NOCHANGE	NOCHANGE
NOCHANGE	NOCHANGE	DOWN	NOCHANGE	NOCHANGE
DOWN	DOWN	DOWN	DOWN	DOWN

TCGA-B2-4102-01.	TCGA-BP-4799-01.	TCGA-BP-4177-01.	TCGA-CZ-4858-01	TCGA-AK-3450-01
NOCHANGE	NOCHANGE	NOCHANGE	NOCHANGE	NOCHANGE
UP	UP	NOCHANGE	NOCHANGE	UP
NOCHANGE	NOCHANGE	NOCHANGE	DOWN	NOCHANGE
NOCHANGE	DOWN	DOWN	DOWN	DOWN
UP	UP	DOWN	NOCHANGE	UP
NOCHANGE	UP	DOWN	DOWN	UP
NOCHANGE	DOWN	DOWN	NOCHANGE	DOWN
DOWN	NOCHANGE	NOCHANGE	NOCHANGE	NOCHANGE
NOCHANGE	NOCHANGE	NOCHANGE	DOWN	NOCHANGE
NOCHANGE	DOWN	NOCHANGE	DOWN	NOCHANGE
DOWN	NOCHANGE	UP	NOCHANGE	NOCHANGE
NOCHANGE	DOWN	UP	UP	NOCHANGE
NOCHANGE	NOCHANGE	NOCHANGE	DOWN	NOCHANGE
NOCHANGE	NOCHANGE	NOCHANGE	DOWN	NOCHANGE
DOWN	NOCHANGE	NOCHANGE	DOWN	DOWN
NOCHANGE	NOCHANGE	NOCHANGE	NOCHANGE	NOCHANGE
UP	DOWN	NOCHANGE	NOCHANGE	NOCHANGE
DOWN	DOWN	NOCHANGE	DOWN	NOCHANGE
UP	DOWN	NOCHANGE	DOWN	NOCHANGE
NOCHANGE	DOWN	DOWN	DOWN	NOCHANGE
UP	NOCHANGE	NOCHANGE	NOCHANGE	NOCHANGE
NOCHANGE	NOCHANGE	NOCHANGE	NOCHANGE	UP
NOCHANGE	NOCHANGE	DOWN	DOWN	DOWN
DOWN	DOWN	NOCHANGE	DOWN	NOCHANGE
NOCHANGE	DOWN	DOWN	DOWN	NOCHANGE
NOCHANGE	NOCHANGE	NOCHANGE	NOCHANGE	NOCHANGE
NOCHANGE	NOCHANGE	NOCHANGE	NOCHANGE	NOCHANGE
NOCHANGE	NOCHANGE	NOCHANGE	NOCHANGE	NOCHANGE
NOCHANGE	NOCHANGE	NOCHANGE	DOWN	NOCHANGE
NOCHANGE	NOCHANGE	NOCHANGE	DOWN	NOCHANGE
DOWN	DOWN	DOWN	DOWN	NOCHANGE

TCGA-B0-5102-01.	TCGA-BP-4167-01.	TCGA-BP-4989-01.	TCGA-BP-5200-01.	TCGA-B0-4818-01.
NOCHANGE	NOCHANGE	NOCHANGE	NOCHANGE	NOCHANGE
UP	UP	UP	UP	UP
NOCHANGE	NOCHANGE	DOWN	NOCHANGE	NOCHANGE
DOWN	DOWN	NOCHANGE	DOWN	NOCHANGE
NOCHANGE	NOCHANGE	NOCHANGE	NOCHANGE	NOCHANGE
DOWN	DOWN	NOCHANGE	NOCHANGE	NOCHANGE
DOWN	DOWN	DOWN	DOWN	DOWN
NOCHANGE	NOCHANGE	NOCHANGE	NOCHANGE	NOCHANGE
NOCHANGE	NOCHANGE	NOCHANGE	NOCHANGE	NOCHANGE
NOCHANGE	NOCHANGE	NOCHANGE	NOCHANGE	UP
NOCHANGE	NOCHANGE	NOCHANGE	DOWN	NOCHANGE
NOCHANGE	NOCHANGE	DOWN	DOWN	NOCHANGE
NOCHANGE	NOCHANGE	NOCHANGE	DOWN	NOCHANGE
NOCHANGE	NOCHANGE	NOCHANGE	NOCHANGE	NOCHANGE
DOWN	NOCHANGE	DOWN	DOWN	DOWN
NOCHANGE	NOCHANGE	NOCHANGE	NOCHANGE	NOCHANGE
NOCHANGE	DOWN	NOCHANGE	NOCHANGE	NOCHANGE
NOCHANGE	NOCHANGE	DOWN	DOWN	NOCHANGE
NOCHANGE	NOCHANGE	NOCHANGE	NOCHANGE	NOCHANGE
NOCHANGE	DOWN	DOWN	NOCHANGE	DOWN
NOCHANGE	UP	UP	UP	NOCHANGE
UP	NOCHANGE	NOCHANGE	NOCHANGE	UP
DOWN	NOCHANGE	DOWN	NOCHANGE	DOWN
NOCHANGE	NOCHANGE	DOWN	DOWN	NOCHANGE
DOWN	DOWN	DOWN	DOWN	NOCHANGE
DOWN	NOCHANGE	NOCHANGE	NOCHANGE	DOWN
NOCHANGE	NOCHANGE	NOCHANGE	NOCHANGE	NOCHANGE
NOCHANGE	NOCHANGE	NOCHANGE	NOCHANGE	NOCHANGE
DOWN	DOWN	DOWN	NOCHANGE	NOCHANGE
NOCHANGE	NOCHANGE	NOCHANGE	NOCHANGE	NOCHANGE
DOWN	DOWN	DOWN	DOWN	DOWN

TCGA-B0-4714-01.	TCGA-3Z-A93Z-01	TCGA-DV-5574-01	TCGA-BP-5194-01.	TCGA-BP-4961-01.
NOCHANGE	NOCHANGE	NOCHANGE	NOCHANGE	NOCHANGE
UP	UP	UP	UP	UP
NOCHANGE	NOCHANGE	NOCHANGE	NOCHANGE	NOCHANGE
NOCHANGE	NOCHANGE	DOWN	DOWN	DOWN
NOCHANGE	NOCHANGE	NOCHANGE	NOCHANGE	NOCHANGE
NOCHANGE	NOCHANGE	NOCHANGE	NOCHANGE	NOCHANGE
DOWN	NOCHANGE	DOWN	DOWN	NOCHANGE
DOWN	NOCHANGE	NOCHANGE	NOCHANGE	NOCHANGE
NOCHANGE	DOWN	NOCHANGE	NOCHANGE	NOCHANGE
NOCHANGE	NOCHANGE	NOCHANGE	NOCHANGE	NOCHANGE
NOCHANGE	NOCHANGE	NOCHANGE	NOCHANGE	NOCHANGE
NOCHANGE	DOWN	NOCHANGE	DOWN	NOCHANGE
NOCHANGE	NOCHANGE	NOCHANGE	NOCHANGE	NOCHANGE
NOCHANGE	NOCHANGE	NOCHANGE	NOCHANGE	NOCHANGE
DOWN	NOCHANGE	NOCHANGE	DOWN	DOWN
NOCHANGE	NOCHANGE	NOCHANGE	NOCHANGE	NOCHANGE
NOCHANGE	NOCHANGE	NOCHANGE	NOCHANGE	NOCHANGE
NOCHANGE	NOCHANGE	DOWN	NOCHANGE	DOWN
NOCHANGE	NOCHANGE	NOCHANGE	UP	NOCHANGE
DOWN	DOWN	DOWN	DOWN	NOCHANGE
NOCHANGE	NOCHANGE	UP	NOCHANGE	NOCHANGE
NOCHANGE	NOCHANGE	NOCHANGE	NOCHANGE	NOCHANGE
DOWN	DOWN	NOCHANGE	NOCHANGE	NOCHANGE
DOWN	NOCHANGE	DOWN	NOCHANGE	DOWN
DOWN	DOWN	DOWN	DOWN	DOWN
DOWN	NOCHANGE	NOCHANGE	DOWN	NOCHANGE
NOCHANGE	NOCHANGE	NOCHANGE	NOCHANGE	NOCHANGE
NOCHANGE	NOCHANGE	NOCHANGE	NOCHANGE	NOCHANGE
DOWN	NOCHANGE	NOCHANGE	NOCHANGE	DOWN
NOCHANGE	NOCHANGE	UP	NOCHANGE	NOCHANGE
DOWN	DOWN	DOWN	DOWN	DOWN

TCGA-BP-4963-01.	TCGA-AK-3460-01	TCGA-B8-5545-01.	TCGA-BP-4165-01.	TCGA-DV-A4VX-0
NOCHANGE	NOCHANGE	NOCHANGE	NOCHANGE	UP
UP	NOCHANGE	UP	UP	UP
NOCHANGE	NOCHANGE	NOCHANGE	NOCHANGE	NOCHANGE
DOWN	DOWN	DOWN	DOWN	DOWN
NOCHANGE	NOCHANGE	NOCHANGE	NOCHANGE	UP
NOCHANGE	NOCHANGE	NOCHANGE	NOCHANGE	UP
NOCHANGE	DOWN	NOCHANGE	DOWN	DOWN
NOCHANGE	NOCHANGE	NOCHANGE	NOCHANGE	UP
NOCHANGE	NOCHANGE	DOWN	DOWN	UP
NOCHANGE	NOCHANGE	NOCHANGE	NOCHANGE	NOCHANGE
DOWN	NOCHANGE	NOCHANGE	UP	NOCHANGE
NOCHANGE	NOCHANGE	NOCHANGE	DOWN	DOWN
NOCHANGE	NOCHANGE	NOCHANGE	NOCHANGE	NOCHANGE
NOCHANGE	NOCHANGE	NOCHANGE	NOCHANGE	NOCHANGE
DOWN	DOWN	NOCHANGE	NOCHANGE	DOWN
UP	NOCHANGE	NOCHANGE	NOCHANGE	NOCHANGE
NOCHANGE	NOCHANGE	NOCHANGE	NOCHANGE	DOWN
DOWN	DOWN	DOWN	NOCHANGE	NOCHANGE
UP	NOCHANGE	NOCHANGE	NOCHANGE	UP
DOWN	NOCHANGE	DOWN	DOWN	UP
UP	NOCHANGE	UP	NOCHANGE	NOCHANGE
NOCHANGE	NOCHANGE	UP	NOCHANGE	NOCHANGE
NOCHANGE	DOWN	NOCHANGE	DOWN	UP
DOWN	DOWN	NOCHANGE	NOCHANGE	NOCHANGE
NOCHANGE	DOWN	DOWN	NOCHANGE	DOWN
DOWN	NOCHANGE	NOCHANGE	NOCHANGE	UP
NOCHANGE	UP	NOCHANGE	NOCHANGE	NOCHANGE
NOCHANGE	NOCHANGE	NOCHANGE	NOCHANGE	NOCHANGE
NOCHANGE	NOCHANGE	NOCHANGE	DOWN	NOCHANGE
NOCHANGE	NOCHANGE	NOCHANGE	NOCHANGE	NOCHANGE
NOCHANGE	NOCHANGE	DOWN	DOWN	UP

TCGA-B0-4846-01	TCGA-CZ-5453-01	TCGA-B0-5095-01	TCGA-BP-4972-01	TCGA-B0-4712-01
NOCHANGE	NOCHANGE	NOCHANGE	NOCHANGE	NOCHANGE
UP	UP	UP	UP	UP
NOCHANGE	NOCHANGE	NOCHANGE	NOCHANGE	NOCHANGE
NOCHANGE	DOWN	DOWN	DOWN	DOWN
UP	NOCHANGE	NOCHANGE	NOCHANGE	UP
NOCHANGE	NOCHANGE	NOCHANGE	NOCHANGE	NOCHANGE
DOWN	DOWN	DOWN	NOCHANGE	NOCHANGE
NOCHANGE	NOCHANGE	NOCHANGE	NOCHANGE	UP
NOCHANGE	NOCHANGE	NOCHANGE	NOCHANGE	NOCHANGE
NOCHANGE	NOCHANGE	NOCHANGE	NOCHANGE	DOWN
NOCHANGE	NOCHANGE	DOWN	DOWN	NOCHANGE
NOCHANGE	DOWN	NOCHANGE	DOWN	NOCHANGE
NOCHANGE	NOCHANGE	NOCHANGE	NOCHANGE	NOCHANGE
NOCHANGE	NOCHANGE	NOCHANGE	NOCHANGE	NOCHANGE
DOWN	DOWN	NOCHANGE	DOWN	NOCHANGE
UP	NOCHANGE	NOCHANGE	NOCHANGE	NOCHANGE
UP	NOCHANGE	NOCHANGE	NOCHANGE	NOCHANGE
DOWN	NOCHANGE	DOWN	DOWN	DOWN
UP	NOCHANGE	NOCHANGE	NOCHANGE	NOCHANGE
DOWN	NOCHANGE	DOWN	DOWN	NOCHANGE
UP	NOCHANGE	NOCHANGE	UP	NOCHANGE
NOCHANGE	NOCHANGE	UP	NOCHANGE	NOCHANGE
NOCHANGE	NOCHANGE	NOCHANGE	UP	UP
DOWN	NOCHANGE	DOWN	DOWN	DOWN
NOCHANGE	NOCHANGE	DOWN	DOWN	DOWN
DOWN	DOWN	DOWN	NOCHANGE	NOCHANGE
NOCHANGE	NOCHANGE	NOCHANGE	NOCHANGE	NOCHANGE
NOCHANGE	NOCHANGE	NOCHANGE	NOCHANGE	NOCHANGE
NOCHANGE	NOCHANGE	DOWN	NOCHANGE	DOWN
NOCHANGE	NOCHANGE	NOCHANGE	NOCHANGE	NOCHANGE
DOWN	DOWN	DOWN	DOWN	DOWN

TCGA-BP-4164-01.	TCGA-CZ-5454-01	TCGA-BP-5202-01.	TCGA-CZ-5470-01	TCGA-CJ-4902-01/
NOCHANGE	NOCHANGE	NOCHANGE	UP	NOCHANGE
UP	UP	UP	UP	UP
NOCHANGE	NOCHANGE	NOCHANGE	NOCHANGE	NOCHANGE
DOWN	DOWN	DOWN	DOWN	DOWN
NOCHANGE	UP	NOCHANGE	NOCHANGE	NOCHANGE
UP	NOCHANGE	NOCHANGE	NOCHANGE	NOCHANGE
DOWN	DOWN	DOWN	NOCHANGE	DOWN
NOCHANGE	NOCHANGE	NOCHANGE	DOWN	NOCHANGE
NOCHANGE	NOCHANGE	NOCHANGE	NOCHANGE	NOCHANGE
NOCHANGE	NOCHANGE	NOCHANGE	NOCHANGE	NOCHANGE
DOWN	NOCHANGE	DOWN	NOCHANGE	NOCHANGE
DOWN	DOWN	NOCHANGE	DOWN	NOCHANGE
NOCHANGE	NOCHANGE	NOCHANGE	NOCHANGE	NOCHANGE
NOCHANGE	NOCHANGE	NOCHANGE	NOCHANGE	NOCHANGE
DOWN	DOWN	DOWN	DOWN	DOWN
NOCHANGE	NOCHANGE	NOCHANGE	NOCHANGE	NOCHANGE
NOCHANGE	NOCHANGE	NOCHANGE	NOCHANGE	NOCHANGE
DOWN	NOCHANGE	NOCHANGE	UP	NOCHANGE
NOCHANGE	NOCHANGE	NOCHANGE	NOCHANGE	NOCHANGE
DOWN	NOCHANGE	DOWN	DOWN	DOWN
NOCHANGE	UP	NOCHANGE	NOCHANGE	NOCHANGE
NOCHANGE	NOCHANGE	NOCHANGE	NOCHANGE	NOCHANGE
NOCHANGE	NOCHANGE	NOCHANGE	NOCHANGE	NOCHANGE
NOCHANGE	NOCHANGE	NOCHANGE	NOCHANGE	DOWN
NOCHANGE	NOCHANGE	NOCHANGE	NOCHANGE	DOWN
DOWN	DOWN	NOCHANGE	NOCHANGE	NOCHANGE
NOCHANGE	NOCHANGE	NOCHANGE	NOCHANGE	NOCHANGE
NOCHANGE	NOCHANGE	NOCHANGE	NOCHANGE	NOCHANGE
NOCHANGE	NOCHANGE	NOCHANGE	NOCHANGE	DOWN
NOCHANGE	NOCHANGE	NOCHANGE	NOCHANGE	NOCHANGE
DOWN	DOWN	NOCHANGE	NOCHANGE	DOWN

TCGA-CJ-4905-01	TCGA-B0-5075-01	TCGA-BP-4798-01	TCGA-CJ-4882-01	TCGA-B0-5706-01
NOCHANGE	UP	NOCHANGE	NOCHANGE	NOCHANGE
NOCHANGE	UP	UP	UP	NOCHANGE
NOCHANGE	NOCHANGE	NOCHANGE	NOCHANGE	NOCHANGE
DOWN	DOWN	DOWN	DOWN	DOWN
NOCHANGE	UP	UP	NOCHANGE	UP
UP	NOCHANGE	NOCHANGE	NOCHANGE	NOCHANGE
DOWN	DOWN	DOWN	NOCHANGE	DOWN
NOCHANGE	NOCHANGE	NOCHANGE	NOCHANGE	NOCHANGE
DOWN	UP	NOCHANGE	UP	NOCHANGE
NOCHANGE	NOCHANGE	DOWN	NOCHANGE	DOWN
DOWN	NOCHANGE	NOCHANGE	NOCHANGE	NOCHANGE
DOWN	NOCHANGE	DOWN	UP	NOCHANGE
NOCHANGE	NOCHANGE	NOCHANGE	NOCHANGE	NOCHANGE
NOCHANGE	NOCHANGE	NOCHANGE	NOCHANGE	NOCHANGE
DOWN	DOWN	DOWN	NOCHANGE	NOCHANGE
NOCHANGE	NOCHANGE	NOCHANGE	NOCHANGE	NOCHANGE
UP	NOCHANGE	NOCHANGE	NOCHANGE	NOCHANGE
DOWN	DOWN	DOWN	DOWN	DOWN
UP	UP	NOCHANGE	NOCHANGE	NOCHANGE
DOWN	NOCHANGE	NOCHANGE	DOWN	DOWN
NOCHANGE	UP	UP	NOCHANGE	NOCHANGE
UP	NOCHANGE	UP	NOCHANGE	UP
NOCHANGE	UP	DOWN	DOWN	NOCHANGE
DOWN	NOCHANGE	DOWN	NOCHANGE	DOWN
NOCHANGE	NOCHANGE	DOWN	DOWN	DOWN
NOCHANGE	NOCHANGE	DOWN	DOWN	DOWN
NOCHANGE	NOCHANGE	NOCHANGE	UP	NOCHANGE
NOCHANGE	NOCHANGE	NOCHANGE	NOCHANGE	NOCHANGE
NOCHANGE	NOCHANGE	DOWN	NOCHANGE	NOCHANGE
DOWN	NOCHANGE	DOWN	NOCHANGE	NOCHANGE
DOWN	NOCHANGE	DOWN	DOWN	DOWN

TCGA-CJ-4891-01/	TCGA-CZ-5988-01	TCGA-AK-3440-01	TCGA-AK-3453-01	TCGA-B0-5697-01.
UP	NOCHANGE	DOWN	NOCHANGE	NOCHANGE
UP	UP	NOCHANGE	UP	NOCHANGE
NOCHANGE	NOCHANGE	NOCHANGE	NOCHANGE	NOCHANGE
DOWN	DOWN	DOWN	DOWN	DOWN
UP	NOCHANGE	NOCHANGE	NOCHANGE	UP
NOCHANGE	NOCHANGE	NOCHANGE	NOCHANGE	NOCHANGE
DOWN	DOWN	NOCHANGE	NOCHANGE	NOCHANGE
UP	NOCHANGE	NOCHANGE	NOCHANGE	NOCHANGE
NOCHANGE	UP	NOCHANGE	NOCHANGE	UP
DOWN	DOWN	UP	NOCHANGE	NOCHANGE
NOCHANGE	NOCHANGE	NOCHANGE	UP	NOCHANGE
NOCHANGE	UP	DOWN	UP	NOCHANGE
NOCHANGE	NOCHANGE	NOCHANGE	NOCHANGE	NOCHANGE
NOCHANGE	NOCHANGE	DOWN	DOWN	NOCHANGE
NOCHANGE	DOWN	DOWN	NOCHANGE	NOCHANGE
NOCHANGE	NOCHANGE	NOCHANGE	NOCHANGE	NOCHANGE
NOCHANGE	UP	NOCHANGE	NOCHANGE	NOCHANGE
NOCHANGE	DOWN	DOWN	NOCHANGE	DOWN
DOWN	UP	NOCHANGE	NOCHANGE	NOCHANGE
NOCHANGE	NOCHANGE	DOWN	DOWN	NOCHANGE
NOCHANGE	NOCHANGE	NOCHANGE	NOCHANGE	UP
NOCHANGE	UP	NOCHANGE	NOCHANGE	NOCHANGE
UP	NOCHANGE	UP	DOWN	NOCHANGE
DOWN	DOWN	UP	NOCHANGE	NOCHANGE
DOWN	DOWN	NOCHANGE	DOWN	DOWN
UP	NOCHANGE	NOCHANGE	DOWN	NOCHANGE
NOCHANGE	NOCHANGE	NOCHANGE	UP	NOCHANGE
NOCHANGE	NOCHANGE	NOCHANGE	NOCHANGE	NOCHANGE
DOWN	NOCHANGE	NOCHANGE	DOWN	NOCHANGE
NOCHANGE	NOCHANGE	NOCHANGE	NOCHANGE	NOCHANGE
UP	DOWN	NOCHANGE	DOWN	NOCHANGE

TCGA-B0-4841-01.	TCGA-CJ-5679-01/	TCGA-B0-4845-01.	TCGA-CZ-5465-01	TCGA-CJ-5686-01/
NOCHANGE	NOCHANGE	NOCHANGE	NOCHANGE	UP
NOCHANGE	UP	UP	UP	UP
NOCHANGE	NOCHANGE	NOCHANGE	NOCHANGE	NOCHANGE
DOWN	DOWN	NOCHANGE	DOWN	NOCHANGE
DOWN	NOCHANGE	NOCHANGE	NOCHANGE	UP
DOWN	UP	NOCHANGE	UP	UP
DOWN	NOCHANGE	NOCHANGE	DOWN	DOWN
NOCHANGE	NOCHANGE	NOCHANGE	NOCHANGE	NOCHANGE
NOCHANGE	NOCHANGE	NOCHANGE	NOCHANGE	NOCHANGE
NOCHANGE	NOCHANGE	NOCHANGE	NOCHANGE	DOWN
UP	NOCHANGE	NOCHANGE	DOWN	NOCHANGE
NOCHANGE	DOWN	NOCHANGE	NOCHANGE	DOWN
NOCHANGE	NOCHANGE	NOCHANGE	NOCHANGE	NOCHANGE
DOWN	DOWN	NOCHANGE	NOCHANGE	NOCHANGE
DOWN	DOWN	DOWN	DOWN	DOWN
DOWN	DOWN	NOCHANGE	NOCHANGE	UP
DOWN	DOWN	NOCHANGE	NOCHANGE	NOCHANGE
NOCHANGE	NOCHANGE	NOCHANGE	DOWN	DOWN
DOWN	NOCHANGE	NOCHANGE	UP	NOCHANGE
DOWN	NOCHANGE	DOWN	DOWN	UP
NOCHANGE	NOCHANGE	NOCHANGE	NOCHANGE	UP
NOCHANGE	NOCHANGE	NOCHANGE	NOCHANGE	NOCHANGE
DOWN	DOWN	DOWN	NOCHANGE	UP
DOWN	NOCHANGE	NOCHANGE	NOCHANGE	DOWN
DOWN	NOCHANGE	NOCHANGE	NOCHANGE	NOCHANGE
NOCHANGE	NOCHANGE	NOCHANGE	NOCHANGE	NOCHANGE
UP	NOCHANGE	UP	NOCHANGE	NOCHANGE
NOCHANGE	NOCHANGE	NOCHANGE	NOCHANGE	NOCHANGE
DOWN	DOWN	NOCHANGE	NOCHANGE	NOCHANGE
NOCHANGE	NOCHANGE	NOCHANGE	NOCHANGE	NOCHANGE
DOWN	NOCHANGE	DOWN	DOWN	NOCHANGE

TCGA-BP-4345-01.	TCGA-B0-5693-01.	TCGA-B0-4838-01.	TCGA-B8-5549-01.	TCGA-CJ-4876-01/
NOCHANGE	NOCHANGE	NOCHANGE	NOCHANGE	NOCHANGE
UP	UP	UP	UP	UP
NOCHANGE	NOCHANGE	NOCHANGE	NOCHANGE	NOCHANGE
DOWN	DOWN	NOCHANGE	DOWN	DOWN
NOCHANGE	NOCHANGE	NOCHANGE	NOCHANGE	NOCHANGE
NOCHANGE	NOCHANGE	NOCHANGE	NOCHANGE	NOCHANGE
DOWN	NOCHANGE	DOWN	DOWN	DOWN
NOCHANGE	NOCHANGE	NOCHANGE	NOCHANGE	NOCHANGE
NOCHANGE	DOWN	NOCHANGE	NOCHANGE	NOCHANGE
NOCHANGE	NOCHANGE	NOCHANGE	NOCHANGE	NOCHANGE
NOCHANGE	NOCHANGE	DOWN	DOWN	UP
NOCHANGE	NOCHANGE	DOWN	DOWN	NOCHANGE
DOWN	NOCHANGE	NOCHANGE	NOCHANGE	NOCHANGE
NOCHANGE	NOCHANGE	NOCHANGE	NOCHANGE	DOWN
NOCHANGE	DOWN	NOCHANGE	DOWN	NOCHANGE
NOCHANGE	NOCHANGE	NOCHANGE	NOCHANGE	NOCHANGE
NOCHANGE	UP	UP	NOCHANGE	NOCHANGE
DOWN	NOCHANGE	DOWN	NOCHANGE	NOCHANGE
NOCHANGE	NOCHANGE	NOCHANGE	NOCHANGE	NOCHANGE
DOWN	DOWN	NOCHANGE	NOCHANGE	NOCHANGE
UP	NOCHANGE	UP	NOCHANGE	NOCHANGE
UP	NOCHANGE	UP	UP	NOCHANGE
NOCHANGE	NOCHANGE	NOCHANGE	NOCHANGE	DOWN
DOWN	NOCHANGE	DOWN	NOCHANGE	NOCHANGE
DOWN	NOCHANGE	NOCHANGE	NOCHANGE	DOWN
NOCHANGE	NOCHANGE	NOCHANGE	NOCHANGE	NOCHANGE
NOCHANGE	NOCHANGE	NOCHANGE	NOCHANGE	NOCHANGE
NOCHANGE	NOCHANGE	NOCHANGE	NOCHANGE	NOCHANGE
DOWN	NOCHANGE	NOCHANGE	NOCHANGE	NOCHANGE
NOCHANGE	NOCHANGE	DOWN	NOCHANGE	NOCHANGE
DOWN	DOWN	DOWN	DOWN	DOWN

TCGA-BP-4337-01.	TCGA-BP-4985-01.	TCGA-A3-3374-01	TCGA-A3-3373-01	TCGA-B0-5700-01.
NOCHANGE	NOCHANGE	NOCHANGE	NOCHANGE	NOCHANGE
UP	UP	UP	UP	UP
NOCHANGE	NOCHANGE	NOCHANGE	NOCHANGE	NOCHANGE
DOWN	DOWN	DOWN	DOWN	DOWN
NOCHANGE	NOCHANGE	NOCHANGE	UP	NOCHANGE
NOCHANGE	UP	NOCHANGE	UP	NOCHANGE
NOCHANGE	NOCHANGE	UP	NOCHANGE	NOCHANGE
NOCHANGE	NOCHANGE	NOCHANGE	NOCHANGE	NOCHANGE
NOCHANGE	UP	NOCHANGE	NOCHANGE	NOCHANGE
NOCHANGE	DOWN	UP	NOCHANGE	NOCHANGE
NOCHANGE	NOCHANGE	DOWN	DOWN	NOCHANGE
NOCHANGE	UP	UP	DOWN	NOCHANGE
NOCHANGE	NOCHANGE	NOCHANGE	NOCHANGE	NOCHANGE
NOCHANGE	NOCHANGE	DOWN	NOCHANGE	DOWN
NOCHANGE	DOWN	DOWN	DOWN	NOCHANGE
DOWN	NOCHANGE	UP	UP	NOCHANGE
NOCHANGE	DOWN	NOCHANGE	NOCHANGE	NOCHANGE
NOCHANGE	NOCHANGE	DOWN	DOWN	NOCHANGE
NOCHANGE	NOCHANGE	DOWN	NOCHANGE	DOWN
NOCHANGE	NOCHANGE	DOWN	DOWN	NOCHANGE
NOCHANGE	NOCHANGE	UP	UP	NOCHANGE
NOCHANGE	NOCHANGE	NOCHANGE	NOCHANGE	NOCHANGE
DOWN	UP	UP	NOCHANGE	DOWN
NOCHANGE	DOWN	NOCHANGE	DOWN	NOCHANGE
DOWN	DOWN	NOCHANGE	NOCHANGE	NOCHANGE
DOWN	NOCHANGE	NOCHANGE	NOCHANGE	NOCHANGE
UP	NOCHANGE	NOCHANGE	NOCHANGE	NOCHANGE
NOCHANGE	NOCHANGE	NOCHANGE	NOCHANGE	NOCHANGE
DOWN	NOCHANGE	DOWN	NOCHANGE	NOCHANGE
NOCHANGE	NOCHANGE	NOCHANGE	DOWN	NOCHANGE
DOWN	DOWN	NOCHANGE	DOWN	DOWN

TCGA-B4-5378-01.	TCGA-B0-5709-01.	TCGA-BP-4351-01.	TCGA-BP-4774-01.	TCGA-BP-4986-01.
DOWN	NOCHANGE	NOCHANGE	NOCHANGE	NOCHANGE
NOCHANGE	UP	UP	UP	NOCHANGE
NOCHANGE	NOCHANGE	NOCHANGE	NOCHANGE	NOCHANGE
DOWN	DOWN	DOWN	DOWN	DOWN
DOWN	UP	NOCHANGE	NOCHANGE	NOCHANGE
DOWN	NOCHANGE	NOCHANGE	UP	NOCHANGE
NOCHANGE	NOCHANGE	DOWN	DOWN	DOWN
NOCHANGE	NOCHANGE	UP	DOWN	NOCHANGE
UP	NOCHANGE	NOCHANGE	NOCHANGE	NOCHANGE
NOCHANGE	NOCHANGE	NOCHANGE	NOCHANGE	DOWN
NOCHANGE	NOCHANGE	NOCHANGE	NOCHANGE	NOCHANGE
NOCHANGE	NOCHANGE	UP	NOCHANGE	UP
NOCHANGE	NOCHANGE	NOCHANGE	NOCHANGE	NOCHANGE
NOCHANGE	UP	DOWN	DOWN	NOCHANGE
NOCHANGE	NOCHANGE	NOCHANGE	DOWN	DOWN
NOCHANGE	NOCHANGE	NOCHANGE	NOCHANGE	NOCHANGE
NOCHANGE	UP	DOWN	NOCHANGE	NOCHANGE
DOWN	DOWN	DOWN	NOCHANGE	DOWN
NOCHANGE	NOCHANGE	NOCHANGE	NOCHANGE	NOCHANGE
DOWN	DOWN	NOCHANGE	DOWN	NOCHANGE
NOCHANGE	UP	NOCHANGE	NOCHANGE	UP
NOCHANGE	UP	NOCHANGE	NOCHANGE	UP
DOWN	NOCHANGE	DOWN	DOWN	NOCHANGE
NOCHANGE	DOWN	DOWN	DOWN	DOWN
DOWN	NOCHANGE	DOWN	NOCHANGE	DOWN
NOCHANGE	NOCHANGE	DOWN	NOCHANGE	NOCHANGE
NOCHANGE	NOCHANGE	NOCHANGE	NOCHANGE	NOCHANGE
NOCHANGE	NOCHANGE	NOCHANGE	NOCHANGE	NOCHANGE
NOCHANGE	NOCHANGE	DOWN	NOCHANGE	NOCHANGE
NOCHANGE	NOCHANGE	NOCHANGE	NOCHANGE	NOCHANGE
DOWN	NOCHANGE	NOCHANGE	DOWN	DOWN

TCGA-B0-5113-01.	TCGA-A3-A6NL-0	TCGA-CJ-5680-01/	TCGA-B0-5402-01.	TCGA-CZ-4862-01
NOCHANGE	NOCHANGE	NOCHANGE	UP	NOCHANGE
UP	UP	UP	NOCHANGE	UP
NOCHANGE	NOCHANGE	NOCHANGE	NOCHANGE	NOCHANGE
DOWN	DOWN	NOCHANGE	DOWN	NOCHANGE
NOCHANGE	NOCHANGE	NOCHANGE	NOCHANGE	UP
UP	NOCHANGE	UP	NOCHANGE	NOCHANGE
DOWN	NOCHANGE	DOWN	DOWN	DOWN
NOCHANGE	NOCHANGE	NOCHANGE	NOCHANGE	NOCHANGE
NOCHANGE	DOWN	NOCHANGE	NOCHANGE	NOCHANGE
NOCHANGE	NOCHANGE	NOCHANGE	NOCHANGE	NOCHANGE
NOCHANGE	NOCHANGE	NOCHANGE	DOWN	NOCHANGE
DOWN	NOCHANGE	DOWN	NOCHANGE	UP
NOCHANGE	NOCHANGE	NOCHANGE	NOCHANGE	NOCHANGE
NOCHANGE	NOCHANGE	NOCHANGE	NOCHANGE	UP
DOWN	DOWN	NOCHANGE	DOWN	DOWN
NOCHANGE	NOCHANGE	DOWN	UP	NOCHANGE
NOCHANGE	NOCHANGE	UP	NOCHANGE	UP
NOCHANGE	DOWN	NOCHANGE	DOWN	DOWN
UP	NOCHANGE	NOCHANGE	NOCHANGE	UP
NOCHANGE	DOWN	DOWN	UP	NOCHANGE
UP	NOCHANGE	NOCHANGE	NOCHANGE	UP
NOCHANGE	NOCHANGE	UP	NOCHANGE	NOCHANGE
NOCHANGE	DOWN	NOCHANGE	NOCHANGE	NOCHANGE
DOWN	DOWN	NOCHANGE	NOCHANGE	DOWN
NOCHANGE	DOWN	NOCHANGE	NOCHANGE	DOWN
DOWN	DOWN	NOCHANGE	NOCHANGE	DOWN
NOCHANGE	NOCHANGE	NOCHANGE	NOCHANGE	NOCHANGE
NOCHANGE	NOCHANGE	NOCHANGE	NOCHANGE	NOCHANGE
NOCHANGE	NOCHANGE	NOCHANGE	NOCHANGE	NOCHANGE
NOCHANGE	NOCHANGE	DOWN	NOCHANGE	NOCHANGE
DOWN	DOWN	NOCHANGE	NOCHANGE	DOWN

TCGA-CW-6097-01	TCGA-B4-5843-01.	TCGA-A3-3307-01	TCGA-B0-4688-01.	TCGA-CJ-5689-01/
NOCHANGE	NOCHANGE	NOCHANGE	NOCHANGE	NOCHANGE
UP	UP	UP	NOCHANGE	UP
NOCHANGE	NOCHANGE	NOCHANGE	NOCHANGE	NOCHANGE
DOWN	NOCHANGE	DOWN	DOWN	DOWN
UP	NOCHANGE	NOCHANGE	NOCHANGE	UP
NOCHANGE	NOCHANGE	NOCHANGE	DOWN	NOCHANGE
DOWN	NOCHANGE	NOCHANGE	NOCHANGE	NOCHANGE
NOCHANGE	NOCHANGE	NOCHANGE	NOCHANGE	NOCHANGE
NOCHANGE	UP	NOCHANGE	UP	UP
DOWN	NOCHANGE	NOCHANGE	NOCHANGE	DOWN
DOWN	NOCHANGE	NOCHANGE	UP	NOCHANGE
DOWN	NOCHANGE	DOWN	UP	NOCHANGE
NOCHANGE	NOCHANGE	NOCHANGE	DOWN	NOCHANGE
UP	NOCHANGE	NOCHANGE	NOCHANGE	NOCHANGE
NOCHANGE	DOWN	DOWN	DOWN	NOCHANGE
NOCHANGE	NOCHANGE	NOCHANGE	NOCHANGE	NOCHANGE
NOCHANGE	NOCHANGE	UP	NOCHANGE	NOCHANGE
DOWN	DOWN	NOCHANGE	DOWN	DOWN
UP	UP	NOCHANGE	NOCHANGE	NOCHANGE
DOWN	DOWN	NOCHANGE	DOWN	DOWN
UP	NOCHANGE	UP	UP	NOCHANGE
UP	NOCHANGE	NOCHANGE	UP	NOCHANGE
NOCHANGE	NOCHANGE	NOCHANGE	UP	NOCHANGE
DOWN	NOCHANGE	NOCHANGE	DOWN	DOWN
DOWN	NOCHANGE	NOCHANGE	DOWN	DOWN
NOCHANGE	NOCHANGE	NOCHANGE	NOCHANGE	NOCHANGE
NOCHANGE	NOCHANGE	NOCHANGE	NOCHANGE	NOCHANGE
NOCHANGE	NOCHANGE	NOCHANGE	NOCHANGE	NOCHANGE
NOCHANGE	NOCHANGE	NOCHANGE	DOWN	NOCHANGE
NOCHANGE	NOCHANGE	DOWN	NOCHANGE	NOCHANGE
NOCHANGE	DOWN	NOCHANGE	DOWN	DOWN

TCGA-BP-4335-01	TCGA-CJ-4908-01	TCGA-CZ-5458-01	TCGA-B0-4690-01	TCGA-CJ-4900-01
NOCHANGE	NOCHANGE	NOCHANGE	UP	NOCHANGE
UP	UP	UP	UP	UP
NOCHANGE	NOCHANGE	NOCHANGE	NOCHANGE	NOCHANGE
NOCHANGE	DOWN	NOCHANGE	DOWN	DOWN
NOCHANGE	NOCHANGE	NOCHANGE	UP	UP
NOCHANGE	NOCHANGE	NOCHANGE	NOCHANGE	NOCHANGE
NOCHANGE	NOCHANGE	DOWN	NOCHANGE	NOCHANGE
NOCHANGE	NOCHANGE	NOCHANGE	NOCHANGE	NOCHANGE
NOCHANGE	NOCHANGE	NOCHANGE	NOCHANGE	UP
NOCHANGE	NOCHANGE	NOCHANGE	NOCHANGE	NOCHANGE
DOWN	DOWN	NOCHANGE	DOWN	NOCHANGE
NOCHANGE	NOCHANGE	NOCHANGE	NOCHANGE	NOCHANGE
NOCHANGE	NOCHANGE	NOCHANGE	DOWN	NOCHANGE
DOWN	NOCHANGE	NOCHANGE	NOCHANGE	NOCHANGE
DOWN	DOWN	NOCHANGE	DOWN	DOWN
NOCHANGE	NOCHANGE	NOCHANGE	NOCHANGE	NOCHANGE
NOCHANGE	NOCHANGE	NOCHANGE	NOCHANGE	NOCHANGE
DOWN	DOWN	DOWN	DOWN	NOCHANGE
NOCHANGE	NOCHANGE	NOCHANGE	NOCHANGE	NOCHANGE
NOCHANGE	DOWN	UP	DOWN	DOWN
NOCHANGE	NOCHANGE	NOCHANGE	UP	NOCHANGE
NOCHANGE	NOCHANGE	NOCHANGE	NOCHANGE	NOCHANGE
DOWN	NOCHANGE	NOCHANGE	UP	NOCHANGE
DOWN	DOWN	NOCHANGE	DOWN	DOWN
DOWN	DOWN	DOWN	DOWN	DOWN
NOCHANGE	NOCHANGE	NOCHANGE	NOCHANGE	NOCHANGE
UP	NOCHANGE	NOCHANGE	NOCHANGE	UP
NOCHANGE	NOCHANGE	NOCHANGE	NOCHANGE	NOCHANGE
NOCHANGE	NOCHANGE	DOWN	NOCHANGE	NOCHANGE
UP	NOCHANGE	NOCHANGE	NOCHANGE	NOCHANGE
DOWN	DOWN	DOWN	DOWN	DOWN

TCGA-A3-3359-01	TCGA-CJ-4907-01/	TCGA-CZ-5460-01	TCGA-B8-4622-01.	TCGA-BP-4994-01.
NOCHANGE	NOCHANGE	NOCHANGE	NOCHANGE	NOCHANGE
UP	UP	UP	UP	NOCHANGE
NOCHANGE	NOCHANGE	NOCHANGE	NOCHANGE	NOCHANGE
NOCHANGE	DOWN	DOWN	NOCHANGE	DOWN
NOCHANGE	NOCHANGE	NOCHANGE	NOCHANGE	NOCHANGE
NOCHANGE	NOCHANGE	UP	NOCHANGE	NOCHANGE
NOCHANGE	DOWN	DOWN	DOWN	NOCHANGE
DOWN	DOWN	NOCHANGE	NOCHANGE	NOCHANGE
DOWN	NOCHANGE	NOCHANGE	NOCHANGE	NOCHANGE
NOCHANGE	NOCHANGE	NOCHANGE	NOCHANGE	NOCHANGE
DOWN	DOWN	NOCHANGE	DOWN	DOWN
DOWN	NOCHANGE	DOWN	UP	DOWN
NOCHANGE	NOCHANGE	NOCHANGE	NOCHANGE	NOCHANGE
NOCHANGE	NOCHANGE	NOCHANGE	NOCHANGE	NOCHANGE
DOWN	NOCHANGE	DOWN	DOWN	DOWN
NOCHANGE	NOCHANGE	NOCHANGE	NOCHANGE	NOCHANGE
NOCHANGE	NOCHANGE	NOCHANGE	NOCHANGE	NOCHANGE
DOWN	DOWN	NOCHANGE	DOWN	DOWN
NOCHANGE	NOCHANGE	NOCHANGE	UP	NOCHANGE
DOWN	NOCHANGE	NOCHANGE	NOCHANGE	DOWN
UP	NOCHANGE	NOCHANGE	UP	NOCHANGE
UP	UP	NOCHANGE	UP	NOCHANGE
DOWN	NOCHANGE	NOCHANGE	NOCHANGE	UP
DOWN	DOWN	NOCHANGE	DOWN	NOCHANGE
NOCHANGE	DOWN	DOWN	DOWN	NOCHANGE
NOCHANGE	NOCHANGE	NOCHANGE	DOWN	NOCHANGE
NOCHANGE	NOCHANGE	NOCHANGE	NOCHANGE	NOCHANGE
NOCHANGE	NOCHANGE	NOCHANGE	NOCHANGE	NOCHANGE
NOCHANGE	NOCHANGE	NOCHANGE	NOCHANGE	NOCHANGE
NOCHANGE	NOCHANGE	NOCHANGE	NOCHANGE	NOCHANGE
DOWN	DOWN	NOCHANGE	NOCHANGE	NOCHANGE

TCGA-B0-4703-01.	TCGA-BP-4346-01.	TCGA-B0-4839-01.	TCGA-CZ-5985-01	TCGA-CZ-4865-01
NOCHANGE	NOCHANGE	NOCHANGE	NOCHANGE	NOCHANGE
NOCHANGE	UP	UP	UP	UP
NOCHANGE	NOCHANGE	NOCHANGE	NOCHANGE	NOCHANGE
DOWN	DOWN	DOWN	DOWN	DOWN
NOCHANGE	NOCHANGE	NOCHANGE	NOCHANGE	UP
NOCHANGE	NOCHANGE	DOWN	NOCHANGE	NOCHANGE
NOCHANGE	DOWN	DOWN	DOWN	DOWN
NOCHANGE	DOWN	NOCHANGE	NOCHANGE	DOWN
NOCHANGE	NOCHANGE	NOCHANGE	UP	NOCHANGE
NOCHANGE	NOCHANGE	NOCHANGE	DOWN	NOCHANGE
NOCHANGE	NOCHANGE	NOCHANGE	NOCHANGE	UP
UP	DOWN	NOCHANGE	DOWN	NOCHANGE
NOCHANGE	NOCHANGE	NOCHANGE	NOCHANGE	NOCHANGE
NOCHANGE	NOCHANGE	DOWN	UP	NOCHANGE
DOWN	NOCHANGE	DOWN	DOWN	NOCHANGE
NOCHANGE	NOCHANGE	NOCHANGE	NOCHANGE	NOCHANGE
NOCHANGE	UP	DOWN	UP	NOCHANGE
DOWN	DOWN	NOCHANGE	NOCHANGE	NOCHANGE
NOCHANGE	UP	NOCHANGE	NOCHANGE	NOCHANGE
DOWN	DOWN	DOWN	UP	DOWN
NOCHANGE	UP	NOCHANGE	UP	NOCHANGE
UP	NOCHANGE	NOCHANGE	NOCHANGE	NOCHANGE
NOCHANGE	DOWN	DOWN	NOCHANGE	NOCHANGE
DOWN	DOWN	NOCHANGE	DOWN	NOCHANGE
DOWN	DOWN	DOWN	NOCHANGE	DOWN
NOCHANGE	DOWN	NOCHANGE	DOWN	NOCHANGE
NOCHANGE	UP	NOCHANGE	NOCHANGE	NOCHANGE
NOCHANGE	NOCHANGE	NOCHANGE	NOCHANGE	NOCHANGE
NOCHANGE	NOCHANGE	DOWN	NOCHANGE	NOCHANGE
NOCHANGE	NOCHANGE	NOCHANGE	NOCHANGE	NOCHANGE
DOWN	NOCHANGE	DOWN	NOCHANGE	DOWN

TCGA-CJ-5675-01/	TCGA-AK-3429-01	TCGA-B0-4843-01.	TCGA-CJ-4886-01/	TCGA-B0-5083-01.
NOCHANGE	NOCHANGE	NOCHANGE	NOCHANGE	DOWN
UP	UP	NOCHANGE	UP	UP
NOCHANGE	NOCHANGE	NOCHANGE	NOCHANGE	NOCHANGE
DOWN	DOWN	DOWN	DOWN	DOWN
NOCHANGE	NOCHANGE	NOCHANGE	NOCHANGE	NOCHANGE
NOCHANGE	NOCHANGE	NOCHANGE	NOCHANGE	DOWN
DOWN	DOWN	DOWN	DOWN	NOCHANGE
DOWN	NOCHANGE	DOWN	NOCHANGE	NOCHANGE
NOCHANGE	DOWN	UP	DOWN	NOCHANGE
NOCHANGE	DOWN	NOCHANGE	NOCHANGE	NOCHANGE
NOCHANGE	NOCHANGE	NOCHANGE	NOCHANGE	NOCHANGE
DOWN	DOWN	NOCHANGE	NOCHANGE	DOWN
NOCHANGE	NOCHANGE	NOCHANGE	NOCHANGE	NOCHANGE
NOCHANGE	NOCHANGE	DOWN	NOCHANGE	DOWN
NOCHANGE	DOWN	NOCHANGE	DOWN	NOCHANGE
NOCHANGE	NOCHANGE	NOCHANGE	NOCHANGE	NOCHANGE
NOCHANGE	NOCHANGE	NOCHANGE	NOCHANGE	NOCHANGE
NOCHANGE	UP	DOWN	DOWN	DOWN
NOCHANGE	NOCHANGE	NOCHANGE	NOCHANGE	NOCHANGE
NOCHANGE	DOWN	DOWN	DOWN	DOWN
UP	NOCHANGE	NOCHANGE	NOCHANGE	UP
UP	NOCHANGE	NOCHANGE	NOCHANGE	NOCHANGE
DOWN	DOWN	DOWN	NOCHANGE	NOCHANGE
NOCHANGE	NOCHANGE	DOWN	DOWN	NOCHANGE
NOCHANGE	DOWN	DOWN	NOCHANGE	NOCHANGE
DOWN	NOCHANGE	DOWN	NOCHANGE	DOWN
NOCHANGE	NOCHANGE	NOCHANGE	NOCHANGE	NOCHANGE
NOCHANGE	NOCHANGE	NOCHANGE	NOCHANGE	NOCHANGE
NOCHANGE	NOCHANGE	DOWN	NOCHANGE	DOWN
NOCHANGE	NOCHANGE	NOCHANGE	NOCHANGE	NOCHANGE
DOWN	DOWN	NOCHANGE	DOWN	NOCHANGE

TCGA-B0-5117-01.	TCGA-BP-4325-01.	TCGA-CW-5580-01	TCGA-BP-5000-01.	TCGA-B0-5106-01.
DOWN	NOCHANGE	NOCHANGE	NOCHANGE	NOCHANGE
NOCHANGE	UP	NOCHANGE	UP	UP
NOCHANGE	NOCHANGE	NOCHANGE	NOCHANGE	NOCHANGE
DOWN	DOWN	DOWN	DOWN	DOWN
DOWN	NOCHANGE	UP	NOCHANGE	UP
NOCHANGE	UP	NOCHANGE	NOCHANGE	DOWN
NOCHANGE	NOCHANGE	DOWN	NOCHANGE	DOWN
DOWN	DOWN	NOCHANGE	NOCHANGE	NOCHANGE
NOCHANGE	NOCHANGE	NOCHANGE	UP	NOCHANGE
UP	NOCHANGE	DOWN	NOCHANGE	NOCHANGE
DOWN	DOWN	NOCHANGE	NOCHANGE	NOCHANGE
DOWN	DOWN	NOCHANGE	NOCHANGE	NOCHANGE
NOCHANGE	NOCHANGE	NOCHANGE	NOCHANGE	NOCHANGE
DOWN	NOCHANGE	NOCHANGE	DOWN	NOCHANGE
DOWN	DOWN	DOWN	NOCHANGE	DOWN
NOCHANGE	UP	UP	NOCHANGE	NOCHANGE
DOWN	UP	UP	NOCHANGE	NOCHANGE
DOWN	DOWN	DOWN	DOWN	NOCHANGE
DOWN	NOCHANGE	UP	NOCHANGE	NOCHANGE
DOWN	DOWN	DOWN	DOWN	DOWN
NOCHANGE	UP	UP	NOCHANGE	NOCHANGE
NOCHANGE	NOCHANGE	NOCHANGE	NOCHANGE	NOCHANGE
DOWN	NOCHANGE	NOCHANGE	DOWN	DOWN
NOCHANGE	DOWN	DOWN	DOWN	DOWN
NOCHANGE	NOCHANGE	NOCHANGE	DOWN	DOWN
DOWN	DOWN	NOCHANGE	NOCHANGE	NOCHANGE
NOCHANGE	NOCHANGE	NOCHANGE	NOCHANGE	UP
NOCHANGE	NOCHANGE	NOCHANGE	NOCHANGE	NOCHANGE
DOWN	NOCHANGE	NOCHANGE	DOWN	NOCHANGE
NOCHANGE	NOCHANGE	NOCHANGE	NOCHANGE	NOCHANGE
NOCHANGE	NOCHANGE	DOWN	DOWN	DOWN

TCGA-CJ-6031-01/	TCGA-B0-4852-01.	TCGA-B0-5690-01.	TCGA-B0-4697-01.	TCGA-CZ-4859-01
NOCHANGE	NOCHANGE	NOCHANGE	NOCHANGE	NOCHANGE
UP	UP	UP	NOCHANGE	UP
NOCHANGE	NOCHANGE	NOCHANGE	NOCHANGE	NOCHANGE
DOWN	DOWN	DOWN	DOWN	DOWN
UP	NOCHANGE	NOCHANGE	UP	DOWN
UP	NOCHANGE	NOCHANGE	DOWN	NOCHANGE
DOWN	DOWN	NOCHANGE	DOWN	DOWN
NOCHANGE	NOCHANGE	DOWN	NOCHANGE	DOWN
UP	DOWN	DOWN	NOCHANGE	DOWN
DOWN	NOCHANGE	NOCHANGE	NOCHANGE	NOCHANGE
NOCHANGE	NOCHANGE	NOCHANGE	NOCHANGE	NOCHANGE
NOCHANGE	DOWN	NOCHANGE	NOCHANGE	DOWN
NOCHANGE	NOCHANGE	NOCHANGE	NOCHANGE	NOCHANGE
NOCHANGE	NOCHANGE	NOCHANGE	NOCHANGE	NOCHANGE
NOCHANGE	DOWN	DOWN	NOCHANGE	DOWN
NOCHANGE	UP	NOCHANGE	DOWN	UP
NOCHANGE	NOCHANGE	NOCHANGE	DOWN	UP
NOCHANGE	DOWN	DOWN	DOWN	NOCHANGE
NOCHANGE	NOCHANGE	NOCHANGE	NOCHANGE	NOCHANGE
DOWN	DOWN	DOWN	DOWN	DOWN
UP	NOCHANGE	NOCHANGE	NOCHANGE	NOCHANGE
UP	NOCHANGE	NOCHANGE	NOCHANGE	NOCHANGE
UP	NOCHANGE	DOWN	DOWN	DOWN
DOWN	DOWN	DOWN	NOCHANGE	NOCHANGE
DOWN	NOCHANGE	NOCHANGE	DOWN	NOCHANGE
NOCHANGE	NOCHANGE	NOCHANGE	NOCHANGE	NOCHANGE
NOCHANGE	NOCHANGE	NOCHANGE	UP	NOCHANGE
NOCHANGE	NOCHANGE	NOCHANGE	NOCHANGE	NOCHANGE
NOCHANGE	NOCHANGE	NOCHANGE	DOWN	NOCHANGE
NOCHANGE	NOCHANGE	NOCHANGE	NOCHANGE	NOCHANGE
DOWN	DOWN	DOWN	DOWN	NOCHANGE

TCGA-BP-4789-01.	TCGA-CZ-5989-01	TCGA-A3-3347-01	TCGA-B0-4827-01.	TCGA-BP-4760-01.
NOCHANGE	NOCHANGE	UP	NOCHANGE	DOWN
UP	NOCHANGE	UP	UP	NOCHANGE
NOCHANGE	NOCHANGE	NOCHANGE	NOCHANGE	NOCHANGE
DOWN	DOWN	DOWN	DOWN	DOWN
NOCHANGE	UP	NOCHANGE	UP	NOCHANGE
NOCHANGE	NOCHANGE	NOCHANGE	UP	NOCHANGE
NOCHANGE	NOCHANGE	NOCHANGE	DOWN	DOWN
NOCHANGE	DOWN	NOCHANGE	NOCHANGE	NOCHANGE
DOWN	NOCHANGE	NOCHANGE	NOCHANGE	UP
DOWN	DOWN	NOCHANGE	NOCHANGE	NOCHANGE
NOCHANGE	UP	DOWN	NOCHANGE	NOCHANGE
NOCHANGE	DOWN	NOCHANGE	DOWN	UP
NOCHANGE	NOCHANGE	DOWN	NOCHANGE	NOCHANGE
NOCHANGE	NOCHANGE	NOCHANGE	NOCHANGE	NOCHANGE
NOCHANGE	DOWN	DOWN	NOCHANGE	NOCHANGE
NOCHANGE	UP	NOCHANGE	NOCHANGE	NOCHANGE
NOCHANGE	NOCHANGE	NOCHANGE	UP	NOCHANGE
DOWN	DOWN	DOWN	DOWN	DOWN
NOCHANGE	UP	UP	NOCHANGE	NOCHANGE
NOCHANGE	NOCHANGE	NOCHANGE	NOCHANGE	DOWN
NOCHANGE	UP	UP	UP	NOCHANGE
UP	NOCHANGE	UP	UP	UP
NOCHANGE	NOCHANGE	UP	DOWN	DOWN
DOWN	DOWN	DOWN	DOWN	NOCHANGE
DOWN	DOWN	NOCHANGE	NOCHANGE	DOWN
NOCHANGE	NOCHANGE	NOCHANGE	DOWN	NOCHANGE
NOCHANGE	NOCHANGE	NOCHANGE	NOCHANGE	NOCHANGE
NOCHANGE	DOWN	NOCHANGE	NOCHANGE	NOCHANGE
NOCHANGE	NOCHANGE	NOCHANGE	DOWN	NOCHANGE
NOCHANGE	NOCHANGE	NOCHANGE	NOCHANGE	NOCHANGE
DOWN	DOWN	DOWN	DOWN	NOCHANGE

TCGA-CW-5581-01	TCGA-AK-3427-01	TCGA-AK-3445-01	TCGA-BP-4763-01.	TCGA-BP-4776-01.
NOCHANGE	DOWN	NOCHANGE	UP	NOCHANGE
UP	NOCHANGE	UP	UP	UP
NOCHANGE	DOWN	NOCHANGE	NOCHANGE	NOCHANGE
DOWN	NOCHANGE	DOWN	DOWN	DOWN
UP	DOWN	UP	UP	NOCHANGE
UP	DOWN	NOCHANGE	NOCHANGE	NOCHANGE
NOCHANGE	NOCHANGE	DOWN	DOWN	NOCHANGE
NOCHANGE	NOCHANGE	NOCHANGE	NOCHANGE	NOCHANGE
NOCHANGE	NOCHANGE	NOCHANGE	NOCHANGE	NOCHANGE
NOCHANGE	NOCHANGE	NOCHANGE	DOWN	NOCHANGE
DOWN	NOCHANGE	NOCHANGE	DOWN	UP
DOWN	DOWN	UP	NOCHANGE	NOCHANGE
NOCHANGE	NOCHANGE	NOCHANGE	NOCHANGE	NOCHANGE
NOCHANGE	DOWN	NOCHANGE	NOCHANGE	DOWN
DOWN	DOWN	DOWN	DOWN	NOCHANGE
NOCHANGE	UP	NOCHANGE	UP	NOCHANGE
NOCHANGE	DOWN	NOCHANGE	NOCHANGE	NOCHANGE
NOCHANGE	DOWN	NOCHANGE	NOCHANGE	NOCHANGE
NOCHANGE	DOWN	NOCHANGE	NOCHANGE	NOCHANGE
NOCHANGE	DOWN	UP	DOWN	NOCHANGE
NOCHANGE	NOCHANGE	UP	NOCHANGE	NOCHANGE
NOCHANGE	DOWN	NOCHANGE	NOCHANGE	NOCHANGE
NOCHANGE	NOCHANGE	NOCHANGE	NOCHANGE	DOWN
NOCHANGE	UP	DOWN	NOCHANGE	NOCHANGE
NOCHANGE	NOCHANGE	DOWN	NOCHANGE	DOWN
NOCHANGE	NOCHANGE	NOCHANGE	NOCHANGE	NOCHANGE
NOCHANGE	UP	NOCHANGE	NOCHANGE	NOCHANGE
NOCHANGE	DOWN	DOWN	NOCHANGE	NOCHANGE
NOCHANGE	NOCHANGE	NOCHANGE	NOCHANGE	DOWN
NOCHANGE	DOWN	DOWN	NOCHANGE	NOCHANGE
NOCHANGE	UP	DOWN	NOCHANGE	DOWN

TCGA-BP-5008-01.	TCGA-B0-5119-01.	TCGA-CJ-5684-01/	TCGA-BP-4338-01.	TCGA-B0-5085-01.
NOCHANGE	NOCHANGE	NOCHANGE	UP	NOCHANGE
UP	UP	UP	UP	UP
NOCHANGE	NOCHANGE	NOCHANGE	NOCHANGE	NOCHANGE
NOCHANGE	DOWN	DOWN	DOWN	DOWN
NOCHANGE	NOCHANGE	NOCHANGE	NOCHANGE	NOCHANGE
NOCHANGE	NOCHANGE	NOCHANGE	UP	NOCHANGE
NOCHANGE	DOWN	NOCHANGE	DOWN	NOCHANGE
NOCHANGE	NOCHANGE	NOCHANGE	UP	NOCHANGE
DOWN	NOCHANGE	DOWN	UP	NOCHANGE
NOCHANGE	NOCHANGE	NOCHANGE	NOCHANGE	NOCHANGE
DOWN	NOCHANGE	NOCHANGE	DOWN	NOCHANGE
NOCHANGE	DOWN	NOCHANGE	UP	NOCHANGE
NOCHANGE	NOCHANGE	NOCHANGE	NOCHANGE	NOCHANGE
NOCHANGE	NOCHANGE	NOCHANGE	UP	DOWN
NOCHANGE	NOCHANGE	DOWN	DOWN	DOWN
NOCHANGE	NOCHANGE	NOCHANGE	NOCHANGE	NOCHANGE
UP	NOCHANGE	NOCHANGE	NOCHANGE	DOWN
DOWN	NOCHANGE	NOCHANGE	NOCHANGE	NOCHANGE
NOCHANGE	UP	NOCHANGE	UP	NOCHANGE
DOWN	NOCHANGE	NOCHANGE	NOCHANGE	DOWN
NOCHANGE	NOCHANGE	NOCHANGE	UP	NOCHANGE
NOCHANGE	NOCHANGE	NOCHANGE	NOCHANGE	NOCHANGE
NOCHANGE	NOCHANGE	NOCHANGE	UP	DOWN
DOWN	NOCHANGE	NOCHANGE	DOWN	NOCHANGE
DOWN	NOCHANGE	DOWN	NOCHANGE	DOWN
NOCHANGE	NOCHANGE	NOCHANGE	UP	DOWN
NOCHANGE	NOCHANGE	NOCHANGE	NOCHANGE	NOCHANGE
UP	NOCHANGE	NOCHANGE	NOCHANGE	NOCHANGE
NOCHANGE	NOCHANGE	DOWN	NOCHANGE	DOWN
UP	NOCHANGE	NOCHANGE	NOCHANGE	NOCHANGE
DOWN	NOCHANGE	DOWN	NOCHANGE	DOWN

TCGA-A3-3380-01	TCGA-B0-4691-01.	TCGA-B0-5695-01.	TCGA-A3-3317-01	TCGA-CJ-5672-01/
NOCHANGE	UP	NOCHANGE	NOCHANGE	NOCHANGE
NOCHANGE	UP	UP	UP	UP
NOCHANGE	NOCHANGE	NOCHANGE	NOCHANGE	NOCHANGE
DOWN	DOWN	DOWN	UP	DOWN
NOCHANGE	UP	NOCHANGE	NOCHANGE	NOCHANGE
NOCHANGE	NOCHANGE	NOCHANGE	UP	NOCHANGE
NOCHANGE	NOCHANGE	NOCHANGE	NOCHANGE	DOWN
NOCHANGE	NOCHANGE	NOCHANGE	DOWN	NOCHANGE
NOCHANGE	UP	NOCHANGE	NOCHANGE	NOCHANGE
NOCHANGE	NOCHANGE	NOCHANGE	NOCHANGE	NOCHANGE
NOCHANGE	NOCHANGE	NOCHANGE	NOCHANGE	NOCHANGE
UP	NOCHANGE	NOCHANGE	UP	NOCHANGE
NOCHANGE	NOCHANGE	NOCHANGE	NOCHANGE	NOCHANGE
NOCHANGE	NOCHANGE	NOCHANGE	NOCHANGE	NOCHANGE
DOWN	DOWN	DOWN	DOWN	NOCHANGE
NOCHANGE	DOWN	NOCHANGE	NOCHANGE	NOCHANGE
NOCHANGE	NOCHANGE	NOCHANGE	NOCHANGE	NOCHANGE
DOWN	NOCHANGE	DOWN	DOWN	NOCHANGE
NOCHANGE	NOCHANGE	NOCHANGE	NOCHANGE	NOCHANGE
DOWN	NOCHANGE	NOCHANGE	DOWN	NOCHANGE
NOCHANGE	NOCHANGE	NOCHANGE	UP	UP
NOCHANGE	NOCHANGE	UP	UP	UP
DOWN	NOCHANGE	DOWN	NOCHANGE	DOWN
DOWN	NOCHANGE	DOWN	DOWN	NOCHANGE
DOWN	DOWN	NOCHANGE	NOCHANGE	NOCHANGE
NOCHANGE	NOCHANGE	NOCHANGE	NOCHANGE	NOCHANGE
NOCHANGE	UP	NOCHANGE	NOCHANGE	NOCHANGE
NOCHANGE	NOCHANGE	NOCHANGE	NOCHANGE	NOCHANGE
DOWN	DOWN	NOCHANGE	NOCHANGE	DOWN
DOWN	NOCHANGE	NOCHANGE	NOCHANGE	NOCHANGE
NOCHANGE	DOWN	DOWN	NOCHANGE	DOWN

TCGA-CJ-5677-01/	TCGA-A3-3367-01	TCGA-B8-A54K-01	TCGA-BP-4992-01.	TCGA-B0-5705-01.
UP	NOCHANGE	DOWN	NOCHANGE	NOCHANGE
UP	UP	NOCHANGE	NOCHANGE	UP
NOCHANGE	NOCHANGE	NOCHANGE	NOCHANGE	NOCHANGE
DOWN	NOCHANGE	DOWN	NOCHANGE	DOWN
UP	NOCHANGE	DOWN	NOCHANGE	NOCHANGE
NOCHANGE	UP	DOWN	NOCHANGE	NOCHANGE
DOWN	NOCHANGE	NOCHANGE	NOCHANGE	DOWN
NOCHANGE	NOCHANGE	NOCHANGE	NOCHANGE	DOWN
NOCHANGE	NOCHANGE	NOCHANGE	NOCHANGE	NOCHANGE
NOCHANGE	NOCHANGE	NOCHANGE	NOCHANGE	NOCHANGE
NOCHANGE	NOCHANGE	NOCHANGE	NOCHANGE	DOWN
NOCHANGE	NOCHANGE	DOWN	UP	NOCHANGE
NOCHANGE	NOCHANGE	DOWN	NOCHANGE	NOCHANGE
NOCHANGE	NOCHANGE	NOCHANGE	DOWN	NOCHANGE
DOWN	DOWN	NOCHANGE	DOWN	DOWN
NOCHANGE	UP	NOCHANGE	DOWN	NOCHANGE
NOCHANGE	NOCHANGE	NOCHANGE	DOWN	NOCHANGE
UP	NOCHANGE	DOWN	DOWN	NOCHANGE
NOCHANGE	UP	NOCHANGE	NOCHANGE	NOCHANGE
NOCHANGE	NOCHANGE	DOWN	DOWN	DOWN
NOCHANGE	UP	NOCHANGE	NOCHANGE	UP
NOCHANGE	NOCHANGE	UP	NOCHANGE	UP
NOCHANGE	NOCHANGE	DOWN	DOWN	NOCHANGE
NOCHANGE	NOCHANGE	NOCHANGE	DOWN	NOCHANGE
DOWN	NOCHANGE	DOWN	DOWN	NOCHANGE
NOCHANGE	NOCHANGE	NOCHANGE	DOWN	NOCHANGE
NOCHANGE	NOCHANGE	NOCHANGE	UP	NOCHANGE
NOCHANGE	NOCHANGE	NOCHANGE	NOCHANGE	NOCHANGE
DOWN	NOCHANGE	DOWN	DOWN	NOCHANGE
NOCHANGE	NOCHANGE	NOCHANGE	NOCHANGE	NOCHANGE
DOWN	NOCHANGE	DOWN	DOWN	NOCHANGE

TCGA-CW-6088-01	TCGA-BP-4758-01.	TCGA-BP-4355-01.	TCGA-CZ-4866-01	TCGA-B8-4621-01.
NOCHANGE	NOCHANGE	NOCHANGE	NOCHANGE	NOCHANGE
UP	UP	UP	UP	UP
NOCHANGE	NOCHANGE	NOCHANGE	UP	NOCHANGE
DOWN	DOWN	NOCHANGE	DOWN	DOWN
NOCHANGE	DOWN	NOCHANGE	UP	NOCHANGE
UP	DOWN	NOCHANGE	UP	NOCHANGE
DOWN	DOWN	NOCHANGE	DOWN	DOWN
DOWN	NOCHANGE	NOCHANGE	NOCHANGE	NOCHANGE
NOCHANGE	DOWN	NOCHANGE	NOCHANGE	UP
NOCHANGE	NOCHANGE	NOCHANGE	DOWN	NOCHANGE
NOCHANGE	UP	NOCHANGE	NOCHANGE	DOWN
UP	DOWN	UP	DOWN	NOCHANGE
NOCHANGE	NOCHANGE	NOCHANGE	NOCHANGE	NOCHANGE
NOCHANGE	DOWN	NOCHANGE	UP	NOCHANGE
DOWN	DOWN	NOCHANGE	DOWN	NOCHANGE
UP	NOCHANGE	NOCHANGE	NOCHANGE	NOCHANGE
UP	NOCHANGE	NOCHANGE	NOCHANGE	NOCHANGE
DOWN	NOCHANGE	DOWN	NOCHANGE	DOWN
UP	NOCHANGE	NOCHANGE	NOCHANGE	NOCHANGE
NOCHANGE	DOWN	DOWN	UP	NOCHANGE
NOCHANGE	NOCHANGE	NOCHANGE	UP	NOCHANGE
UP	NOCHANGE	NOCHANGE	NOCHANGE	NOCHANGE
UP	DOWN	DOWN	NOCHANGE	UP
DOWN	NOCHANGE	DOWN	DOWN	NOCHANGE
NOCHANGE	DOWN	DOWN	NOCHANGE	NOCHANGE
NOCHANGE	DOWN	NOCHANGE	NOCHANGE	NOCHANGE
NOCHANGE	NOCHANGE	NOCHANGE	NOCHANGE	NOCHANGE
NOCHANGE	NOCHANGE	NOCHANGE	NOCHANGE	NOCHANGE
NOCHANGE	DOWN	DOWN	NOCHANGE	DOWN
NOCHANGE	NOCHANGE	NOCHANGE	NOCHANGE	DOWN
DOWN	DOWN	DOWN	UP	NOCHANGE

TCGA-BP-4995-01.	TCGA-BP-5004-01.	TCGA-B8-5162-01.	TCGA-B0-4847-01.	TCGA-BP-4769-01.
NOCHANGE	NOCHANGE	NOCHANGE	NOCHANGE	NOCHANGE
NOCHANGE	UP	UP	UP	NOCHANGE
NOCHANGE	NOCHANGE	NOCHANGE	NOCHANGE	NOCHANGE
DOWN	DOWN	DOWN	DOWN	NOCHANGE
NOCHANGE	UP	NOCHANGE	NOCHANGE	NOCHANGE
NOCHANGE	NOCHANGE	DOWN	NOCHANGE	NOCHANGE
NOCHANGE	DOWN	DOWN	NOCHANGE	DOWN
NOCHANGE	NOCHANGE	NOCHANGE	NOCHANGE	NOCHANGE
NOCHANGE	NOCHANGE	NOCHANGE	NOCHANGE	UP
NOCHANGE	NOCHANGE	DOWN	NOCHANGE	NOCHANGE
DOWN	DOWN	NOCHANGE	UP	NOCHANGE
DOWN	NOCHANGE	NOCHANGE	NOCHANGE	UP
NOCHANGE	NOCHANGE	DOWN	NOCHANGE	NOCHANGE
NOCHANGE	NOCHANGE	NOCHANGE	DOWN	NOCHANGE
DOWN	DOWN	DOWN	NOCHANGE	NOCHANGE
NOCHANGE	UP	UP	NOCHANGE	NOCHANGE
NOCHANGE	NOCHANGE	NOCHANGE	NOCHANGE	NOCHANGE
DOWN	DOWN	DOWN	NOCHANGE	DOWN
NOCHANGE	NOCHANGE	NOCHANGE	NOCHANGE	NOCHANGE
DOWN	NOCHANGE	DOWN	DOWN	DOWN
NOCHANGE	NOCHANGE	UP	NOCHANGE	NOCHANGE
NOCHANGE	UP	NOCHANGE	NOCHANGE	UP
UP	NOCHANGE	NOCHANGE	DOWN	DOWN
NOCHANGE	DOWN	DOWN	NOCHANGE	DOWN
NOCHANGE	NOCHANGE	DOWN	DOWN	DOWN
NOCHANGE	NOCHANGE	NOCHANGE	DOWN	NOCHANGE
NOCHANGE	NOCHANGE	NOCHANGE	NOCHANGE	NOCHANGE
NOCHANGE	NOCHANGE	NOCHANGE	NOCHANGE	UP
NOCHANGE	NOCHANGE	NOCHANGE	DOWN	NOCHANGE
NOCHANGE	NOCHANGE	NOCHANGE	NOCHANGE	NOCHANGE
NOCHANGE	DOWN	DOWN	DOWN	NOCHANGE

TCGA-CZ-5982-01	TCGA-CJ-4870-01	TCGA-AK-3454-01	TCGA-BP-4975-01	TCGA-CW-5585-01
NOCHANGE	NOCHANGE	NOCHANGE	NOCHANGE	NOCHANGE
NOCHANGE	UP	UP	UP	UP
NOCHANGE	NOCHANGE	NOCHANGE	NOCHANGE	NOCHANGE
DOWN	DOWN	DOWN	DOWN	DOWN
UP	NOCHANGE	NOCHANGE	NOCHANGE	DOWN
UP	NOCHANGE	NOCHANGE	UP	NOCHANGE
DOWN	DOWN	NOCHANGE	DOWN	DOWN
NOCHANGE	NOCHANGE	NOCHANGE	NOCHANGE	DOWN
DOWN	DOWN	NOCHANGE	UP	NOCHANGE
NOCHANGE	NOCHANGE	NOCHANGE	NOCHANGE	NOCHANGE
DOWN	NOCHANGE	UP	DOWN	NOCHANGE
NOCHANGE	DOWN	UP	NOCHANGE	DOWN
NOCHANGE	NOCHANGE	NOCHANGE	NOCHANGE	NOCHANGE
NOCHANGE	NOCHANGE	DOWN	NOCHANGE	NOCHANGE
DOWN	DOWN	NOCHANGE	DOWN	DOWN
NOCHANGE	NOCHANGE	DOWN	NOCHANGE	NOCHANGE
UP	NOCHANGE	DOWN	NOCHANGE	NOCHANGE
DOWN	NOCHANGE	DOWN	NOCHANGE	NOCHANGE
NOCHANGE	NOCHANGE	NOCHANGE	UP	NOCHANGE
DOWN	NOCHANGE	DOWN	NOCHANGE	NOCHANGE
NOCHANGE	NOCHANGE	NOCHANGE	NOCHANGE	NOCHANGE
UP	NOCHANGE	NOCHANGE	NOCHANGE	UP
NOCHANGE	DOWN	DOWN	NOCHANGE	DOWN
DOWN	NOCHANGE	NOCHANGE	NOCHANGE	NOCHANGE
NOCHANGE	DOWN	DOWN	NOCHANGE	NOCHANGE
DOWN	NOCHANGE	NOCHANGE	NOCHANGE	NOCHANGE
NOCHANGE	NOCHANGE	NOCHANGE	NOCHANGE	NOCHANGE
NOCHANGE	NOCHANGE	NOCHANGE	NOCHANGE	NOCHANGE
NOCHANGE	NOCHANGE	DOWN	NOCHANGE	NOCHANGE
DOWN	NOCHANGE	NOCHANGE	NOCHANGE	DOWN
NOCHANGE	DOWN	DOWN	NOCHANGE	DOWN

TCGA-B2-4098-01.	TCGA-CJ-5678-01/	TCGA-B0-5702-01.	TCGA-CJ-4638-01/	TCGA-DV-5573-01
UP	UP	NOCHANGE	UP	NOCHANGE
UP	UP	UP	UP	UP
NOCHANGE	NOCHANGE	NOCHANGE	NOCHANGE	NOCHANGE
DOWN	DOWN	DOWN	DOWN	DOWN
UP	UP	DOWN	NOCHANGE	NOCHANGE
UP	NOCHANGE	DOWN	NOCHANGE	NOCHANGE
DOWN	DOWN	NOCHANGE	DOWN	NOCHANGE
NOCHANGE	NOCHANGE	NOCHANGE	NOCHANGE	NOCHANGE
UP	NOCHANGE	NOCHANGE	UP	NOCHANGE
NOCHANGE	NOCHANGE	UP	NOCHANGE	NOCHANGE
NOCHANGE	NOCHANGE	DOWN	UP	NOCHANGE
DOWN	DOWN	NOCHANGE	NOCHANGE	NOCHANGE
NOCHANGE	NOCHANGE	NOCHANGE	NOCHANGE	NOCHANGE
NOCHANGE	NOCHANGE	DOWN	NOCHANGE	NOCHANGE
DOWN	DOWN	NOCHANGE	DOWN	DOWN
UP	NOCHANGE	DOWN	DOWN	NOCHANGE
NOCHANGE	NOCHANGE	DOWN	DOWN	NOCHANGE
NOCHANGE	UP	UP	NOCHANGE	NOCHANGE
NOCHANGE	NOCHANGE	DOWN	NOCHANGE	NOCHANGE
NOCHANGE	NOCHANGE	DOWN	DOWN	NOCHANGE
UP	NOCHANGE	UP	NOCHANGE	NOCHANGE
NOCHANGE	NOCHANGE	NOCHANGE	NOCHANGE	NOCHANGE
UP	DOWN	DOWN	DOWN	NOCHANGE
DOWN	NOCHANGE	UP	NOCHANGE	DOWN
DOWN	DOWN	DOWN	DOWN	DOWN
UP	DOWN	NOCHANGE	NOCHANGE	NOCHANGE
NOCHANGE	NOCHANGE	NOCHANGE	UP	NOCHANGE
NOCHANGE	NOCHANGE	NOCHANGE	NOCHANGE	NOCHANGE
NOCHANGE	NOCHANGE	DOWN	DOWN	NOCHANGE
DOWN	NOCHANGE	NOCHANGE	DOWN	NOCHANGE
UP	NOCHANGE	DOWN	NOCHANGE	DOWN

TCGA-BP-5196-01	TCGA-A3-3351-01	TCGA-B8-A54D-01	TCGA-A3-3322-01	TCGA-B8-A54H-01
NOCHANGE	NOCHANGE	NOCHANGE	NOCHANGE	NOCHANGE
NOCHANGE	UP	UP	UP	UP
NOCHANGE	NOCHANGE	NOCHANGE	NOCHANGE	NOCHANGE
DOWN	DOWN	NOCHANGE	DOWN	DOWN
NOCHANGE	UP	NOCHANGE	NOCHANGE	NOCHANGE
NOCHANGE	NOCHANGE	NOCHANGE	NOCHANGE	NOCHANGE
DOWN	NOCHANGE	DOWN	NOCHANGE	DOWN
NOCHANGE	NOCHANGE	NOCHANGE	DOWN	DOWN
NOCHANGE	DOWN	NOCHANGE	NOCHANGE	NOCHANGE
DOWN	NOCHANGE	NOCHANGE	NOCHANGE	NOCHANGE
NOCHANGE	DOWN	UP	DOWN	NOCHANGE
UP	NOCHANGE	NOCHANGE	DOWN	DOWN
NOCHANGE	NOCHANGE	NOCHANGE	NOCHANGE	NOCHANGE
NOCHANGE	NOCHANGE	NOCHANGE	NOCHANGE	NOCHANGE
DOWN	DOWN	DOWN	DOWN	NOCHANGE
DOWN	NOCHANGE	NOCHANGE	UP	NOCHANGE
NOCHANGE	UP	NOCHANGE	NOCHANGE	UP
DOWN	DOWN	DOWN	NOCHANGE	NOCHANGE
UP	UP	NOCHANGE	UP	UP
DOWN	DOWN	DOWN	DOWN	DOWN
NOCHANGE	UP	NOCHANGE	UP	NOCHANGE
UP	UP	NOCHANGE	UP	UP
NOCHANGE	DOWN	DOWN	NOCHANGE	DOWN
NOCHANGE	DOWN	DOWN	DOWN	DOWN
DOWN	DOWN	DOWN	DOWN	NOCHANGE
NOCHANGE	DOWN	DOWN	NOCHANGE	NOCHANGE
NOCHANGE	NOCHANGE	NOCHANGE	NOCHANGE	NOCHANGE
NOCHANGE	NOCHANGE	NOCHANGE	NOCHANGE	NOCHANGE
NOCHANGE	NOCHANGE	DOWN	NOCHANGE	NOCHANGE
NOCHANGE	NOCHANGE	NOCHANGE	NOCHANGE	NOCHANGE
DOWN	DOWN	NOCHANGE	DOWN	NOCHANGE

TCGA-A3-A8OW-0	TCGA-B0-4701-01.	TCGA-B2-4101-01.	TCGA-A3-3320-01	TCGA-BP-4999-01.
NOCHANGE	NOCHANGE	NOCHANGE	NOCHANGE	NOCHANGE
UP	NOCHANGE	UP	UP	NOCHANGE
NOCHANGE	NOCHANGE	NOCHANGE	NOCHANGE	NOCHANGE
DOWN	DOWN	DOWN	NOCHANGE	NOCHANGE
NOCHANGE	UP	UP	NOCHANGE	NOCHANGE
NOCHANGE	UP	NOCHANGE	UP	NOCHANGE
NOCHANGE	NOCHANGE	NOCHANGE	NOCHANGE	NOCHANGE
NOCHANGE	UP	NOCHANGE	NOCHANGE	NOCHANGE
DOWN	NOCHANGE	NOCHANGE	NOCHANGE	DOWN
NOCHANGE	NOCHANGE	NOCHANGE	NOCHANGE	NOCHANGE
NOCHANGE	NOCHANGE	NOCHANGE	DOWN	DOWN
NOCHANGE	NOCHANGE	NOCHANGE	NOCHANGE	NOCHANGE
NOCHANGE	NOCHANGE	NOCHANGE	NOCHANGE	NOCHANGE
NOCHANGE	NOCHANGE	NOCHANGE	NOCHANGE	NOCHANGE
DOWN	DOWN	DOWN	DOWN	DOWN
NOCHANGE	NOCHANGE	NOCHANGE	UP	NOCHANGE
NOCHANGE	UP	NOCHANGE	NOCHANGE	NOCHANGE
NOCHANGE	DOWN	DOWN	DOWN	DOWN
NOCHANGE	NOCHANGE	NOCHANGE	UP	UP
NOCHANGE	DOWN	NOCHANGE	DOWN	NOCHANGE
NOCHANGE	NOCHANGE	UP	NOCHANGE	NOCHANGE
UP	NOCHANGE	UP	UP	NOCHANGE
DOWN	UP	UP	NOCHANGE	NOCHANGE
NOCHANGE	NOCHANGE	DOWN	DOWN	DOWN
NOCHANGE	DOWN	DOWN	NOCHANGE	DOWN
NOCHANGE	UP	NOCHANGE	NOCHANGE	DOWN
UP	NOCHANGE	NOCHANGE	UP	NOCHANGE
NOCHANGE	NOCHANGE	NOCHANGE	NOCHANGE	NOCHANGE
DOWN	NOCHANGE	NOCHANGE	NOCHANGE	NOCHANGE
NOCHANGE	NOCHANGE	DOWN	NOCHANGE	NOCHANGE
DOWN	NOCHANGE	DOWN	DOWN	DOWN

TCGA-BP-4161-01.	TCGA-B8-4143-01.	TCGA-B0-5701-01.	TCGA-BP-4797-01.	TCGA-DV-A4W0-0
NOCHANGE	NOCHANGE	UP	NOCHANGE	DOWN
UP	UP	UP	UP	NOCHANGE
NOCHANGE	NOCHANGE	NOCHANGE	NOCHANGE	NOCHANGE
DOWN	DOWN	DOWN	DOWN	DOWN
UP	UP	UP	UP	NOCHANGE
NOCHANGE	NOCHANGE	NOCHANGE	UP	NOCHANGE
DOWN	DOWN	DOWN	DOWN	DOWN
NOCHANGE	NOCHANGE	NOCHANGE	NOCHANGE	NOCHANGE
NOCHANGE	NOCHANGE	NOCHANGE	NOCHANGE	NOCHANGE
NOCHANGE	NOCHANGE	DOWN	NOCHANGE	NOCHANGE
NOCHANGE	NOCHANGE	NOCHANGE	NOCHANGE	DOWN
NOCHANGE	NOCHANGE	NOCHANGE	NOCHANGE	NOCHANGE
NOCHANGE	NOCHANGE	NOCHANGE	NOCHANGE	DOWN
NOCHANGE	NOCHANGE	NOCHANGE	NOCHANGE	NOCHANGE
DOWN	DOWN	DOWN	DOWN	NOCHANGE
NOCHANGE	NOCHANGE	NOCHANGE	NOCHANGE	DOWN
NOCHANGE	NOCHANGE	NOCHANGE	NOCHANGE	NOCHANGE
DOWN	DOWN	NOCHANGE	DOWN	DOWN
UP	NOCHANGE	NOCHANGE	NOCHANGE	UP
NOCHANGE	DOWN	NOCHANGE	NOCHANGE	DOWN
UP	NOCHANGE	NOCHANGE	UP	NOCHANGE
UP	NOCHANGE	NOCHANGE	NOCHANGE	NOCHANGE
NOCHANGE	DOWN	NOCHANGE	NOCHANGE	DOWN
DOWN	DOWN	DOWN	NOCHANGE	NOCHANGE
DOWN	DOWN	NOCHANGE	NOCHANGE	DOWN
NOCHANGE	NOCHANGE	NOCHANGE	NOCHANGE	NOCHANGE
NOCHANGE	NOCHANGE	NOCHANGE	NOCHANGE	NOCHANGE
NOCHANGE	NOCHANGE	DOWN	NOCHANGE	NOCHANGE
NOCHANGE	DOWN	DOWN	NOCHANGE	NOCHANGE
NOCHANGE	NOCHANGE	DOWN	NOCHANGE	DOWN
NOCHANGE	DOWN	NOCHANGE	NOCHANGE	DOWN

TCGA-BP-4174-01.	TCGA-A3-3385-01	TCGA-CZ-5469-01	TCGA-BP-4970-01.	TCGA-BP-4330-01.
NOCHANGE	NOCHANGE	NOCHANGE	NOCHANGE	NOCHANGE
UP	UP	NOCHANGE	UP	UP
NOCHANGE	NOCHANGE	NOCHANGE	NOCHANGE	NOCHANGE
NOCHANGE	DOWN	DOWN	DOWN	DOWN
UP	NOCHANGE	NOCHANGE	UP	NOCHANGE
NOCHANGE	UP	DOWN	NOCHANGE	NOCHANGE
DOWN	NOCHANGE	DOWN	DOWN	DOWN
DOWN	DOWN	NOCHANGE	NOCHANGE	NOCHANGE
UP	NOCHANGE	UP	NOCHANGE	NOCHANGE
NOCHANGE	NOCHANGE	NOCHANGE	NOCHANGE	NOCHANGE
DOWN	DOWN	UP	NOCHANGE	DOWN
NOCHANGE	NOCHANGE	NOCHANGE	NOCHANGE	NOCHANGE
NOCHANGE	NOCHANGE	NOCHANGE	NOCHANGE	NOCHANGE
NOCHANGE	NOCHANGE	NOCHANGE	NOCHANGE	NOCHANGE
DOWN	DOWN	DOWN	NOCHANGE	DOWN
NOCHANGE	NOCHANGE	DOWN	NOCHANGE	NOCHANGE
NOCHANGE	NOCHANGE	NOCHANGE	UP	NOCHANGE
NOCHANGE	NOCHANGE	NOCHANGE	DOWN	DOWN
NOCHANGE	UP	NOCHANGE	NOCHANGE	UP
NOCHANGE	DOWN	DOWN	NOCHANGE	DOWN
UP	NOCHANGE	NOCHANGE	UP	UP
UP	NOCHANGE	NOCHANGE	UP	UP
NOCHANGE	DOWN	DOWN	NOCHANGE	DOWN
NOCHANGE	DOWN	DOWN	DOWN	DOWN
NOCHANGE	NOCHANGE	DOWN	DOWN	DOWN
NOCHANGE	DOWN	NOCHANGE	NOCHANGE	DOWN
NOCHANGE	NOCHANGE	NOCHANGE	NOCHANGE	NOCHANGE
NOCHANGE	DOWN	NOCHANGE	NOCHANGE	NOCHANGE
NOCHANGE	DOWN	DOWN	NOCHANGE	NOCHANGE
NOCHANGE	DOWN	DOWN	NOCHANGE	DOWN
DOWN	NOCHANGE	NOCHANGE	DOWN	DOWN

TCGA-BP-4349-01	TCGA-AK-3461-01	TCGA-A3-3383-01	TCGA-BP-4159-01	TCGA-B2-5635-01
NOCHANGE	NOCHANGE	NOCHANGE	NOCHANGE	NOCHANGE
NOCHANGE	NOCHANGE	UP	UP	NOCHANGE
NOCHANGE	NOCHANGE	NOCHANGE	NOCHANGE	NOCHANGE
NOCHANGE	NOCHANGE	DOWN	DOWN	NOCHANGE
NOCHANGE	UP	NOCHANGE	NOCHANGE	UP
UP	NOCHANGE	NOCHANGE	NOCHANGE	NOCHANGE
NOCHANGE	NOCHANGE	NOCHANGE	DOWN	DOWN
NOCHANGE	NOCHANGE	NOCHANGE	NOCHANGE	DOWN
NOCHANGE	NOCHANGE	DOWN	NOCHANGE	NOCHANGE
NOCHANGE	NOCHANGE	UP	NOCHANGE	NOCHANGE
NOCHANGE	NOCHANGE	UP	NOCHANGE	NOCHANGE
NOCHANGE	NOCHANGE	NOCHANGE	DOWN	NOCHANGE
NOCHANGE	NOCHANGE	NOCHANGE	NOCHANGE	NOCHANGE
NOCHANGE	NOCHANGE	NOCHANGE	DOWN	UP
NOCHANGE	NOCHANGE	DOWN	DOWN	DOWN
NOCHANGE	NOCHANGE	NOCHANGE	NOCHANGE	UP
NOCHANGE	UP	NOCHANGE	NOCHANGE	NOCHANGE
NOCHANGE	DOWN	NOCHANGE	NOCHANGE	DOWN
NOCHANGE	NOCHANGE	NOCHANGE	DOWN	UP
DOWN	NOCHANGE	DOWN	DOWN	NOCHANGE
NOCHANGE	NOCHANGE	NOCHANGE	NOCHANGE	UP
NOCHANGE	NOCHANGE	NOCHANGE	NOCHANGE	NOCHANGE
NOCHANGE	DOWN	DOWN	DOWN	NOCHANGE
DOWN	DOWN	NOCHANGE	NOCHANGE	DOWN
NOCHANGE	NOCHANGE	DOWN	DOWN	DOWN
NOCHANGE	DOWN	NOCHANGE	NOCHANGE	DOWN
NOCHANGE	NOCHANGE	NOCHANGE	NOCHANGE	NOCHANGE
NOCHANGE	NOCHANGE	NOCHANGE	NOCHANGE	DOWN
NOCHANGE	NOCHANGE	DOWN	NOCHANGE	NOCHANGE
NOCHANGE	NOCHANGE	DOWN	DOWN	NOCHANGE
DOWN	DOWN	DOWN	DOWN	DOWN

TCGA-CJ-4889-01A	TCGA-BP-4962-01.	TCGA-CJ-5682-01A	TCGA-BP-4347-01.	TCGA-BP-5186-01.
NOCHANGE	NOCHANGE	NOCHANGE	NOCHANGE	NOCHANGE
UP	UP	UP	UP	UP
NOCHANGE	NOCHANGE	NOCHANGE	NOCHANGE	NOCHANGE
DOWN	DOWN	DOWN	DOWN	DOWN
NOCHANGE	UP	NOCHANGE	NOCHANGE	NOCHANGE
NOCHANGE	NOCHANGE	NOCHANGE	UP	NOCHANGE
DOWN	NOCHANGE	DOWN	DOWN	DOWN
NOCHANGE	NOCHANGE	NOCHANGE	NOCHANGE	DOWN
NOCHANGE	UP	NOCHANGE	NOCHANGE	DOWN
NOCHANGE	NOCHANGE	NOCHANGE	NOCHANGE	NOCHANGE
NOCHANGE	DOWN	NOCHANGE	NOCHANGE	NOCHANGE
NOCHANGE	NOCHANGE	DOWN	NOCHANGE	UP
NOCHANGE	NOCHANGE	NOCHANGE	NOCHANGE	NOCHANGE
NOCHANGE	NOCHANGE	NOCHANGE	NOCHANGE	NOCHANGE
NOCHANGE	NOCHANGE	NOCHANGE	DOWN	DOWN
NOCHANGE	NOCHANGE	NOCHANGE	NOCHANGE	NOCHANGE
UP	NOCHANGE	NOCHANGE	NOCHANGE	UP
DOWN	NOCHANGE	DOWN	DOWN	DOWN
NOCHANGE	DOWN	UP	NOCHANGE	NOCHANGE
DOWN	NOCHANGE	NOCHANGE	NOCHANGE	DOWN
UP	UP	NOCHANGE	NOCHANGE	NOCHANGE
UP	NOCHANGE	UP	UP	NOCHANGE
UP	NOCHANGE	NOCHANGE	NOCHANGE	DOWN
DOWN	NOCHANGE	NOCHANGE	DOWN	NOCHANGE
DOWN	DOWN	NOCHANGE	DOWN	NOCHANGE
NOCHANGE	NOCHANGE	NOCHANGE	NOCHANGE	DOWN
NOCHANGE	NOCHANGE	NOCHANGE	NOCHANGE	NOCHANGE
NOCHANGE	NOCHANGE	NOCHANGE	NOCHANGE	NOCHANGE
NOCHANGE	DOWN	NOCHANGE	NOCHANGE	NOCHANGE
NOCHANGE	DOWN	NOCHANGE	NOCHANGE	NOCHANGE
DOWN	NOCHANGE	DOWN	DOWN	DOWN

TCGA-CZ-4856-01	TCGA-B0-4824-01.	TCGA-A3-3376-01	TCGA-CJ-4635-01/	TCGA-BP-5183-01.
NOCHANGE	NOCHANGE	NOCHANGE	NOCHANGE	NOCHANGE
UP	UP	UP	UP	UP
NOCHANGE	NOCHANGE	NOCHANGE	NOCHANGE	NOCHANGE
DOWN	DOWN	DOWN	DOWN	DOWN
NOCHANGE	NOCHANGE	NOCHANGE	NOCHANGE	NOCHANGE
NOCHANGE	NOCHANGE	NOCHANGE	NOCHANGE	UP
NOCHANGE	DOWN	NOCHANGE	NOCHANGE	DOWN
NOCHANGE	DOWN	DOWN	NOCHANGE	NOCHANGE
NOCHANGE	NOCHANGE	NOCHANGE	DOWN	NOCHANGE
NOCHANGE	NOCHANGE	NOCHANGE	NOCHANGE	NOCHANGE
NOCHANGE	NOCHANGE	DOWN	NOCHANGE	DOWN
NOCHANGE	NOCHANGE	NOCHANGE	DOWN	NOCHANGE
NOCHANGE	NOCHANGE	NOCHANGE	NOCHANGE	NOCHANGE
NOCHANGE	NOCHANGE	NOCHANGE	NOCHANGE	NOCHANGE
DOWN	DOWN	DOWN	DOWN	DOWN
NOCHANGE	UP	NOCHANGE	NOCHANGE	NOCHANGE
NOCHANGE	NOCHANGE	NOCHANGE	NOCHANGE	NOCHANGE
NOCHANGE	NOCHANGE	DOWN	DOWN	NOCHANGE
NOCHANGE	NOCHANGE	UP	NOCHANGE	NOCHANGE
DOWN	DOWN	DOWN	NOCHANGE	NOCHANGE
NOCHANGE	UP	UP	UP	NOCHANGE
NOCHANGE	NOCHANGE	NOCHANGE	UP	UP
NOCHANGE	DOWN	NOCHANGE	NOCHANGE	NOCHANGE
NOCHANGE	NOCHANGE	DOWN	DOWN	NOCHANGE
DOWN	NOCHANGE	DOWN	DOWN	NOCHANGE
NOCHANGE	NOCHANGE	NOCHANGE	DOWN	NOCHANGE
NOCHANGE	NOCHANGE	NOCHANGE	NOCHANGE	NOCHANGE
NOCHANGE	NOCHANGE	NOCHANGE	NOCHANGE	NOCHANGE
DOWN	DOWN	NOCHANGE	NOCHANGE	DOWN
NOCHANGE	NOCHANGE	NOCHANGE	NOCHANGE	DOWN
DOWN	DOWN	DOWN	NOCHANGE	NOCHANGE

TCGA-A3-A8OV-0	TCGA-B8-5159-01.	TCGA-B8-5163-01.	TCGA-CZ-5461-01	TCGA-GK-A6C7-0
NOCHANGE	NOCHANGE	NOCHANGE	NOCHANGE	NOCHANGE
UP	UP	UP	UP	NOCHANGE
NOCHANGE	NOCHANGE	NOCHANGE	NOCHANGE	NOCHANGE
DOWN	NOCHANGE	DOWN	DOWN	DOWN
NOCHANGE	NOCHANGE	NOCHANGE	UP	NOCHANGE
UP	NOCHANGE	NOCHANGE	NOCHANGE	NOCHANGE
DOWN	DOWN	DOWN	DOWN	NOCHANGE
NOCHANGE	NOCHANGE	NOCHANGE	NOCHANGE	NOCHANGE
DOWN	DOWN	NOCHANGE	DOWN	UP
NOCHANGE	NOCHANGE	DOWN	DOWN	NOCHANGE
NOCHANGE	DOWN	DOWN	DOWN	NOCHANGE
DOWN	NOCHANGE	UP	DOWN	DOWN
NOCHANGE	NOCHANGE	NOCHANGE	NOCHANGE	NOCHANGE
NOCHANGE	NOCHANGE	NOCHANGE	NOCHANGE	NOCHANGE
DOWN	DOWN	DOWN	DOWN	DOWN
NOCHANGE	NOCHANGE	NOCHANGE	NOCHANGE	UP
NOCHANGE	NOCHANGE	NOCHANGE	NOCHANGE	UP
NOCHANGE	DOWN	DOWN	NOCHANGE	NOCHANGE
NOCHANGE	NOCHANGE	NOCHANGE	UP	NOCHANGE
NOCHANGE	DOWN	DOWN	DOWN	NOCHANGE
NOCHANGE	NOCHANGE	UP	UP	NOCHANGE
NOCHANGE	UP	NOCHANGE	NOCHANGE	NOCHANGE
DOWN	NOCHANGE	NOCHANGE	NOCHANGE	DOWN
NOCHANGE	NOCHANGE	DOWN	DOWN	DOWN
NOCHANGE	NOCHANGE	DOWN	NOCHANGE	DOWN
NOCHANGE	NOCHANGE	NOCHANGE	NOCHANGE	DOWN
NOCHANGE	NOCHANGE	NOCHANGE	NOCHANGE	NOCHANGE
NOCHANGE	DOWN	NOCHANGE	NOCHANGE	NOCHANGE
DOWN	NOCHANGE	NOCHANGE	NOCHANGE	NOCHANGE
NOCHANGE	DOWN	NOCHANGE	NOCHANGE	NOCHANGE
DOWN	NOCHANGE	DOWN	DOWN	DOWN

TCGA-BP-4771-01.	TCGA-BP-4803-01.	TCGA-B2-5639-01.	TCGA-CZ-5455-01	TCGA-CJ-4897-01/
NOCHANGE	NOCHANGE	NOCHANGE	NOCHANGE	NOCHANGE
UP	UP	UP	UP	UP
NOCHANGE	NOCHANGE	NOCHANGE	NOCHANGE	NOCHANGE
DOWN	DOWN	DOWN	DOWN	DOWN
UP	UP	NOCHANGE	UP	NOCHANGE
NOCHANGE	NOCHANGE	NOCHANGE	NOCHANGE	UP
DOWN	DOWN	DOWN	DOWN	DOWN
NOCHANGE	NOCHANGE	DOWN	NOCHANGE	NOCHANGE
NOCHANGE	DOWN	DOWN	UP	DOWN
NOCHANGE	DOWN	NOCHANGE	NOCHANGE	NOCHANGE
NOCHANGE	DOWN	NOCHANGE	NOCHANGE	DOWN
NOCHANGE	UP	DOWN	NOCHANGE	NOCHANGE
NOCHANGE	NOCHANGE	NOCHANGE	NOCHANGE	NOCHANGE
NOCHANGE	NOCHANGE	NOCHANGE	NOCHANGE	NOCHANGE
NOCHANGE	DOWN	DOWN	DOWN	NOCHANGE
NOCHANGE	NOCHANGE	NOCHANGE	UP	NOCHANGE
NOCHANGE	NOCHANGE	UP	UP	NOCHANGE
DOWN	DOWN	NOCHANGE	DOWN	NOCHANGE
NOCHANGE	UP	NOCHANGE	NOCHANGE	NOCHANGE
DOWN	NOCHANGE	NOCHANGE	NOCHANGE	NOCHANGE
UP	NOCHANGE	UP	UP	NOCHANGE
NOCHANGE	UP	NOCHANGE	NOCHANGE	UP
DOWN	NOCHANGE	NOCHANGE	NOCHANGE	DOWN
DOWN	DOWN	NOCHANGE	DOWN	DOWN
DOWN	DOWN	NOCHANGE	NOCHANGE	NOCHANGE
DOWN	DOWN	NOCHANGE	NOCHANGE	NOCHANGE
NOCHANGE	NOCHANGE	NOCHANGE	NOCHANGE	NOCHANGE
NOCHANGE	NOCHANGE	NOCHANGE	NOCHANGE	NOCHANGE
DOWN	NOCHANGE	NOCHANGE	NOCHANGE	NOCHANGE
NOCHANGE	NOCHANGE	NOCHANGE	DOWN	NOCHANGE
DOWN	DOWN	NOCHANGE	NOCHANGE	DOWN

TCGA-B0-5107-01.	TCGA-BP-4759-01.	TCGA-B0-4836-01.	TCGA-B8-A7U6-01	TCGA-B2-5636-01.
UP	NOCHANGE	NOCHANGE	NOCHANGE	DOWN
NOCHANGE	UP	UP	UP	NOCHANGE
NOCHANGE	NOCHANGE	NOCHANGE	NOCHANGE	NOCHANGE
DOWN	DOWN	DOWN	DOWN	DOWN
UP	NOCHANGE	NOCHANGE	UP	DOWN
NOCHANGE	NOCHANGE	NOCHANGE	NOCHANGE	NOCHANGE
DOWN	DOWN	NOCHANGE	DOWN	DOWN
DOWN	NOCHANGE	NOCHANGE	NOCHANGE	NOCHANGE
NOCHANGE	NOCHANGE	NOCHANGE	NOCHANGE	NOCHANGE
NOCHANGE	DOWN	NOCHANGE	NOCHANGE	NOCHANGE
NOCHANGE	DOWN	NOCHANGE	NOCHANGE	NOCHANGE
DOWN	DOWN	NOCHANGE	NOCHANGE	UP
NOCHANGE	NOCHANGE	NOCHANGE	NOCHANGE	DOWN
NOCHANGE	DOWN	NOCHANGE	NOCHANGE	NOCHANGE
DOWN	NOCHANGE	NOCHANGE	DOWN	NOCHANGE
DOWN	NOCHANGE	NOCHANGE	NOCHANGE	NOCHANGE
UP	NOCHANGE	NOCHANGE	UP	NOCHANGE
NOCHANGE	NOCHANGE	NOCHANGE	NOCHANGE	DOWN
NOCHANGE	NOCHANGE	NOCHANGE	NOCHANGE	NOCHANGE
DOWN	DOWN	NOCHANGE	DOWN	DOWN
NOCHANGE	NOCHANGE	NOCHANGE	NOCHANGE	UP
NOCHANGE	NOCHANGE	UP	NOCHANGE	NOCHANGE
DOWN	DOWN	NOCHANGE	DOWN	NOCHANGE
NOCHANGE	NOCHANGE	DOWN	NOCHANGE	DOWN
DOWN	DOWN	DOWN	DOWN	DOWN
NOCHANGE	NOCHANGE	NOCHANGE	NOCHANGE	NOCHANGE
NOCHANGE	NOCHANGE	NOCHANGE	NOCHANGE	NOCHANGE
NOCHANGE	NOCHANGE	NOCHANGE	UP	NOCHANGE
DOWN	DOWN	DOWN	NOCHANGE	DOWN
DOWN	DOWN	NOCHANGE	UP	NOCHANGE
DOWN	DOWN	NOCHANGE	DOWN	NOCHANGE

TCGA-A3-3382-01	TCGA-CZ-5464-01	TCGA-CJ-4634-01	TCGA-CJ-6028-01	TCGA-BP-4804-01
NOCHANGE	NOCHANGE	NOCHANGE	NOCHANGE	NOCHANGE
UP	UP	UP	UP	UP
NOCHANGE	NOCHANGE	NOCHANGE	NOCHANGE	NOCHANGE
DOWN	DOWN	DOWN	DOWN	DOWN
UP	NOCHANGE	DOWN	UP	NOCHANGE
UP	NOCHANGE	UP	NOCHANGE	NOCHANGE
DOWN	DOWN	DOWN	DOWN	NOCHANGE
NOCHANGE	NOCHANGE	NOCHANGE	NOCHANGE	NOCHANGE
UP	NOCHANGE	UP	UP	NOCHANGE
NOCHANGE	NOCHANGE	NOCHANGE	DOWN	NOCHANGE
NOCHANGE	NOCHANGE	NOCHANGE	NOCHANGE	NOCHANGE
NOCHANGE	NOCHANGE	NOCHANGE	NOCHANGE	NOCHANGE
NOCHANGE	NOCHANGE	NOCHANGE	NOCHANGE	NOCHANGE
NOCHANGE	NOCHANGE	NOCHANGE	NOCHANGE	NOCHANGE
DOWN	DOWN	DOWN	DOWN	NOCHANGE
NOCHANGE	NOCHANGE	NOCHANGE	UP	NOCHANGE
NOCHANGE	NOCHANGE	UP	NOCHANGE	NOCHANGE
DOWN	NOCHANGE	DOWN	DOWN	DOWN
UP	UP	UP	UP	NOCHANGE
UP	NOCHANGE	DOWN	UP	NOCHANGE
UP	UP	NOCHANGE	UP	UP
UP	NOCHANGE	UP	UP	NOCHANGE
UP	NOCHANGE	NOCHANGE	UP	NOCHANGE
DOWN	NOCHANGE	DOWN	DOWN	DOWN
DOWN	DOWN	NOCHANGE	DOWN	DOWN
DOWN	DOWN	NOCHANGE	NOCHANGE	NOCHANGE
NOCHANGE	NOCHANGE	NOCHANGE	NOCHANGE	NOCHANGE
NOCHANGE	NOCHANGE	NOCHANGE	NOCHANGE	NOCHANGE
NOCHANGE	NOCHANGE	NOCHANGE	NOCHANGE	DOWN
NOCHANGE	NOCHANGE	NOCHANGE	NOCHANGE	NOCHANGE
DOWN	NOCHANGE	DOWN	DOWN	DOWN

TCGA-CJ-4912-01/	TCGA-CJ-4873-01/	TCGA-CJ-4894-01/	TCGA-CW-5588-01	TCGA-A3-3325-01
NOCHANGE	NOCHANGE	NOCHANGE	UP	NOCHANGE
UP	UP	UP	UP	UP
NOCHANGE	NOCHANGE	NOCHANGE	NOCHANGE	NOCHANGE
DOWN	DOWN	NOCHANGE	DOWN	DOWN
UP	NOCHANGE	NOCHANGE	UP	NOCHANGE
NOCHANGE	DOWN	NOCHANGE	NOCHANGE	UP
NOCHANGE	DOWN	NOCHANGE	DOWN	NOCHANGE
NOCHANGE	NOCHANGE	DOWN	NOCHANGE	DOWN
UP	NOCHANGE	DOWN	DOWN	NOCHANGE
NOCHANGE	NOCHANGE	NOCHANGE	NOCHANGE	NOCHANGE
DOWN	NOCHANGE	DOWN	DOWN	NOCHANGE
NOCHANGE	NOCHANGE	NOCHANGE	NOCHANGE	NOCHANGE
NOCHANGE	NOCHANGE	NOCHANGE	NOCHANGE	NOCHANGE
DOWN	DOWN	NOCHANGE	NOCHANGE	NOCHANGE
DOWN	DOWN	DOWN	DOWN	DOWN
DOWN	NOCHANGE	NOCHANGE	NOCHANGE	NOCHANGE
DOWN	NOCHANGE	UP	NOCHANGE	NOCHANGE
DOWN	NOCHANGE	DOWN	DOWN	DOWN
NOCHANGE	NOCHANGE	UP	UP	NOCHANGE
NOCHANGE	DOWN	DOWN	NOCHANGE	DOWN
NOCHANGE	NOCHANGE	UP	UP	UP
NOCHANGE	NOCHANGE	UP	NOCHANGE	UP
DOWN	DOWN	NOCHANGE	UP	NOCHANGE
DOWN	NOCHANGE	DOWN	NOCHANGE	DOWN
DOWN	DOWN	NOCHANGE	DOWN	NOCHANGE
DOWN	NOCHANGE	NOCHANGE	NOCHANGE	NOCHANGE
NOCHANGE	NOCHANGE	NOCHANGE	NOCHANGE	NOCHANGE
NOCHANGE	NOCHANGE	NOCHANGE	NOCHANGE	NOCHANGE
DOWN	DOWN	NOCHANGE	DOWN	NOCHANGE
NOCHANGE	NOCHANGE	NOCHANGE	NOCHANGE	NOCHANGE
DOWN	DOWN	DOWN	NOCHANGE	DOWN

TCGA-CJ-4871-01	TCGA-CJ-4640-01	TCGA-BP-4762-01	TCGA-B0-5094-01	TCGA-CZ-5467-01
NOCHANGE	NOCHANGE	NOCHANGE	NOCHANGE	NOCHANGE
UP	UP	UP	UP	UP
NOCHANGE	NOCHANGE	NOCHANGE	NOCHANGE	NOCHANGE
DOWN	DOWN	NOCHANGE	DOWN	DOWN
UP	UP	NOCHANGE	UP	UP
UP	NOCHANGE	NOCHANGE	UP	NOCHANGE
DOWN	NOCHANGE	DOWN	DOWN	NOCHANGE
NOCHANGE	DOWN	NOCHANGE	NOCHANGE	UP
NOCHANGE	UP	NOCHANGE	NOCHANGE	DOWN
DOWN	NOCHANGE	DOWN	NOCHANGE	NOCHANGE
NOCHANGE	NOCHANGE	DOWN	NOCHANGE	DOWN
DOWN	DOWN	NOCHANGE	UP	DOWN
NOCHANGE	NOCHANGE	NOCHANGE	NOCHANGE	NOCHANGE
DOWN	NOCHANGE	NOCHANGE	NOCHANGE	NOCHANGE
DOWN	NOCHANGE	NOCHANGE	DOWN	DOWN
NOCHANGE	NOCHANGE	NOCHANGE	NOCHANGE	NOCHANGE
NOCHANGE	NOCHANGE	NOCHANGE	NOCHANGE	NOCHANGE
DOWN	NOCHANGE	DOWN	DOWN	DOWN
NOCHANGE	NOCHANGE	NOCHANGE	UP	NOCHANGE
NOCHANGE	NOCHANGE	DOWN	UP	DOWN
NOCHANGE	UP	UP	NOCHANGE	NOCHANGE
UP	UP	NOCHANGE	NOCHANGE	NOCHANGE
NOCHANGE	DOWN	NOCHANGE	UP	NOCHANGE
DOWN	NOCHANGE	NOCHANGE	DOWN	NOCHANGE
DOWN	NOCHANGE	NOCHANGE	DOWN	NOCHANGE
NOCHANGE	NOCHANGE	NOCHANGE	DOWN	NOCHANGE
NOCHANGE	NOCHANGE	NOCHANGE	NOCHANGE	NOCHANGE
NOCHANGE	NOCHANGE	NOCHANGE	NOCHANGE	NOCHANGE
NOCHANGE	NOCHANGE	NOCHANGE	DOWN	NOCHANGE
NOCHANGE	NOCHANGE	NOCHANGE	DOWN	NOCHANGE
NOCHANGE	DOWN	DOWN	DOWN	DOWN

TCGA-A3-3363-01	TCGA-CZ-5468-01	TCGA-B2-3924-01	TCGA-BP-5181-01	TCGA-B8-5553-01
NOCHANGE	UP	NOCHANGE	NOCHANGE	NOCHANGE
NOCHANGE	UP	NOCHANGE	UP	UP
NOCHANGE	NOCHANGE	NOCHANGE	NOCHANGE	NOCHANGE
NOCHANGE	DOWN	UP	DOWN	DOWN
NOCHANGE	NOCHANGE	NOCHANGE	NOCHANGE	UP
NOCHANGE	NOCHANGE	NOCHANGE	UP	NOCHANGE
NOCHANGE	DOWN	NOCHANGE	DOWN	DOWN
DOWN	NOCHANGE	NOCHANGE	NOCHANGE	NOCHANGE
NOCHANGE	NOCHANGE	NOCHANGE	DOWN	NOCHANGE
NOCHANGE	DOWN	NOCHANGE	NOCHANGE	NOCHANGE
NOCHANGE	NOCHANGE	NOCHANGE	NOCHANGE	DOWN
NOCHANGE	NOCHANGE	UP	DOWN	UP
NOCHANGE	DOWN	NOCHANGE	NOCHANGE	NOCHANGE
UP	NOCHANGE	NOCHANGE	NOCHANGE	NOCHANGE
DOWN	DOWN	DOWN	NOCHANGE	DOWN
UP	NOCHANGE	UP	NOCHANGE	UP
NOCHANGE	NOCHANGE	UP	NOCHANGE	NOCHANGE
DOWN	UP	DOWN	NOCHANGE	DOWN
UP	NOCHANGE	UP	UP	NOCHANGE
NOCHANGE	UP	NOCHANGE	DOWN	UP
UP	NOCHANGE	UP	NOCHANGE	UP
DOWN	NOCHANGE	NOCHANGE	NOCHANGE	NOCHANGE
NOCHANGE	NOCHANGE	NOCHANGE	NOCHANGE	DOWN
NOCHANGE	DOWN	DOWN	NOCHANGE	DOWN
NOCHANGE	DOWN	DOWN	NOCHANGE	NOCHANGE
DOWN	NOCHANGE	DOWN	NOCHANGE	NOCHANGE
NOCHANGE	NOCHANGE	NOCHANGE	NOCHANGE	NOCHANGE
NOCHANGE	NOCHANGE	DOWN	NOCHANGE	NOCHANGE
NOCHANGE	DOWN	NOCHANGE	NOCHANGE	DOWN
DOWN	NOCHANGE	NOCHANGE	NOCHANGE	NOCHANGE
NOCHANGE	NOCHANGE	DOWN	DOWN	DOWN

TCGA-BP-4761-01.	TCGA-B2-5633-01.	TCGA-A3-3358-01	TCGA-CZ-4864-01	TCGA-AK-3436-01
NOCHANGE	NOCHANGE	NOCHANGE	NOCHANGE	UP
UP	UP	UP	UP	NOCHANGE
NOCHANGE	NOCHANGE	NOCHANGE	NOCHANGE	NOCHANGE
DOWN	NOCHANGE	DOWN	DOWN	DOWN
UP	UP	UP	NOCHANGE	NOCHANGE
DOWN	NOCHANGE	NOCHANGE	NOCHANGE	NOCHANGE
NOCHANGE	DOWN	NOCHANGE	DOWN	DOWN
NOCHANGE	NOCHANGE	NOCHANGE	DOWN	NOCHANGE
UP	DOWN	NOCHANGE	NOCHANGE	NOCHANGE
NOCHANGE	NOCHANGE	NOCHANGE	NOCHANGE	DOWN
NOCHANGE	DOWN	DOWN	NOCHANGE	DOWN
UP	NOCHANGE	DOWN	DOWN	NOCHANGE
NOCHANGE	DOWN	NOCHANGE	NOCHANGE	NOCHANGE
DOWN	NOCHANGE	NOCHANGE	NOCHANGE	DOWN
DOWN	DOWN	DOWN	DOWN	DOWN
DOWN	NOCHANGE	NOCHANGE	UP	NOCHANGE
DOWN	UP	NOCHANGE	NOCHANGE	DOWN
NOCHANGE	NOCHANGE	DOWN	NOCHANGE	NOCHANGE
NOCHANGE	NOCHANGE	NOCHANGE	NOCHANGE	NOCHANGE
NOCHANGE	NOCHANGE	DOWN	UP	UP
NOCHANGE	UP	UP	UP	NOCHANGE
NOCHANGE	DOWN	NOCHANGE	NOCHANGE	UP
DOWN	UP	NOCHANGE	DOWN	NOCHANGE
UP	DOWN	NOCHANGE	DOWN	NOCHANGE
DOWN	NOCHANGE	DOWN	NOCHANGE	DOWN
NOCHANGE	NOCHANGE	NOCHANGE	DOWN	NOCHANGE
NOCHANGE	NOCHANGE	NOCHANGE	NOCHANGE	UP
NOCHANGE	DOWN	NOCHANGE	NOCHANGE	DOWN
DOWN	DOWN	NOCHANGE	NOCHANGE	DOWN
DOWN	DOWN	NOCHANGE	NOCHANGE	NOCHANGE
DOWN	UP	NOCHANGE	DOWN	DOWN

TCGA-CZ-5466-01	TCGA-B0-5080-01.	TCGA-CZ-4861-01	TCGA-CJ-5671-01/	TCGA-AK-3431-01
UP	NOCHANGE	NOCHANGE	NOCHANGE	NOCHANGE
UP	NOCHANGE	UP	UP	NOCHANGE
NOCHANGE	NOCHANGE	NOCHANGE	NOCHANGE	NOCHANGE
DOWN	DOWN	DOWN	DOWN	DOWN
NOCHANGE	NOCHANGE	UP	NOCHANGE	NOCHANGE
UP	NOCHANGE	NOCHANGE	NOCHANGE	NOCHANGE
DOWN	DOWN	DOWN	NOCHANGE	DOWN
NOCHANGE	NOCHANGE	NOCHANGE	NOCHANGE	NOCHANGE
NOCHANGE	NOCHANGE	NOCHANGE	NOCHANGE	NOCHANGE
NOCHANGE	DOWN	NOCHANGE	NOCHANGE	DOWN
DOWN	NOCHANGE	NOCHANGE	DOWN	DOWN
DOWN	UP	DOWN	NOCHANGE	DOWN
NOCHANGE	NOCHANGE	NOCHANGE	NOCHANGE	NOCHANGE
NOCHANGE	NOCHANGE	UP	NOCHANGE	NOCHANGE
NOCHANGE	NOCHANGE	DOWN	NOCHANGE	DOWN
NOCHANGE	NOCHANGE	NOCHANGE	NOCHANGE	NOCHANGE
NOCHANGE	NOCHANGE	NOCHANGE	NOCHANGE	NOCHANGE
DOWN	DOWN	DOWN	DOWN	DOWN
NOCHANGE	NOCHANGE	NOCHANGE	NOCHANGE	NOCHANGE
NOCHANGE	DOWN	NOCHANGE	DOWN	NOCHANGE
UP	NOCHANGE	UP	UP	NOCHANGE
NOCHANGE	UP	NOCHANGE	NOCHANGE	NOCHANGE
UP	NOCHANGE	NOCHANGE	NOCHANGE	NOCHANGE
NOCHANGE	DOWN	NOCHANGE	NOCHANGE	NOCHANGE
DOWN	DOWN	DOWN	DOWN	DOWN
NOCHANGE	NOCHANGE	NOCHANGE	NOCHANGE	NOCHANGE
NOCHANGE	NOCHANGE	NOCHANGE	NOCHANGE	NOCHANGE
NOCHANGE	NOCHANGE	NOCHANGE	NOCHANGE	NOCHANGE
DOWN	NOCHANGE	NOCHANGE	NOCHANGE	NOCHANGE
NOCHANGE	NOCHANGE	NOCHANGE	NOCHANGE	NOCHANGE
DOWN	DOWN	NOCHANGE	DOWN	DOWN

TCGA-B0-4811-01.	TCGA-A3-3313-01	TCGA-B8-5552-01.	TCGA-CJ-6027-01.	TCGA-BP-4756-01.
NOCHANGE	NOCHANGE	NOCHANGE	NOCHANGE	NOCHANGE
UP	NOCHANGE	UP	UP	UP
NOCHANGE	NOCHANGE	NOCHANGE	NOCHANGE	NOCHANGE
DOWN	DOWN	DOWN	DOWN	NOCHANGE
NOCHANGE	NOCHANGE	NOCHANGE	UP	NOCHANGE
NOCHANGE	NOCHANGE	NOCHANGE	NOCHANGE	NOCHANGE
NOCHANGE	NOCHANGE	NOCHANGE	NOCHANGE	NOCHANGE
NOCHANGE	DOWN	NOCHANGE	NOCHANGE	NOCHANGE
NOCHANGE	NOCHANGE	DOWN	NOCHANGE	NOCHANGE
NOCHANGE	NOCHANGE	NOCHANGE	DOWN	NOCHANGE
NOCHANGE	DOWN	NOCHANGE	NOCHANGE	DOWN
NOCHANGE	NOCHANGE	DOWN	DOWN	NOCHANGE
NOCHANGE	NOCHANGE	NOCHANGE	NOCHANGE	NOCHANGE
NOCHANGE	NOCHANGE	NOCHANGE	NOCHANGE	NOCHANGE
DOWN	DOWN	NOCHANGE	DOWN	DOWN
NOCHANGE	UP	NOCHANGE	NOCHANGE	UP
DOWN	NOCHANGE	UP	NOCHANGE	NOCHANGE
DOWN	DOWN	DOWN	NOCHANGE	NOCHANGE
NOCHANGE	NOCHANGE	NOCHANGE	NOCHANGE	NOCHANGE
DOWN	NOCHANGE	NOCHANGE	DOWN	NOCHANGE
NOCHANGE	NOCHANGE	UP	NOCHANGE	NOCHANGE
NOCHANGE	NOCHANGE	NOCHANGE	NOCHANGE	NOCHANGE
DOWN	NOCHANGE	NOCHANGE	NOCHANGE	NOCHANGE
DOWN	DOWN	DOWN	NOCHANGE	NOCHANGE
DOWN	NOCHANGE	DOWN	DOWN	NOCHANGE
NOCHANGE	NOCHANGE	NOCHANGE	NOCHANGE	NOCHANGE
UP	NOCHANGE	NOCHANGE	NOCHANGE	NOCHANGE
NOCHANGE	NOCHANGE	NOCHANGE	NOCHANGE	DOWN
DOWN	DOWN	NOCHANGE	NOCHANGE	NOCHANGE
NOCHANGE	NOCHANGE	NOCHANGE	DOWN	DOWN
DOWN	UP	DOWN	DOWN	NOCHANGE

TCGA-A3-3319-01	TCGA-MW-A4EC-(	TCGA-B0-4816-01.	TCGA-CZ-4863-01	TCGA-B0-5110-01.
UP	NOCHANGE	NOCHANGE	NOCHANGE	NOCHANGE
UP	UP	UP	UP	UP
NOCHANGE	NOCHANGE	NOCHANGE	NOCHANGE	NOCHANGE
DOWN	DOWN	DOWN	DOWN	NOCHANGE
UP	NOCHANGE	UP	UP	NOCHANGE
NOCHANGE	UP	NOCHANGE	UP	NOCHANGE
DOWN	NOCHANGE	DOWN	DOWN	DOWN
NOCHANGE	NOCHANGE	NOCHANGE	NOCHANGE	DOWN
UP	DOWN	NOCHANGE	NOCHANGE	DOWN
NOCHANGE	NOCHANGE	NOCHANGE	NOCHANGE	NOCHANGE
NOCHANGE	NOCHANGE	NOCHANGE	DOWN	NOCHANGE
UP	UP	UP	NOCHANGE	NOCHANGE
NOCHANGE	NOCHANGE	NOCHANGE	NOCHANGE	NOCHANGE
NOCHANGE	NOCHANGE	NOCHANGE	NOCHANGE	NOCHANGE
DOWN	DOWN	NOCHANGE	DOWN	DOWN
DOWN	NOCHANGE	NOCHANGE	NOCHANGE	NOCHANGE
NOCHANGE	NOCHANGE	NOCHANGE	UP	UP
NOCHANGE	NOCHANGE	DOWN	NOCHANGE	DOWN
UP	NOCHANGE	NOCHANGE	NOCHANGE	NOCHANGE
UP	NOCHANGE	DOWN	NOCHANGE	DOWN
UP	NOCHANGE	UP	UP	UP
NOCHANGE	NOCHANGE	UP	NOCHANGE	UP
UP	DOWN	DOWN	NOCHANGE	NOCHANGE
DOWN	NOCHANGE	DOWN	DOWN	DOWN
NOCHANGE	NOCHANGE	NOCHANGE	NOCHANGE	NOCHANGE
NOCHANGE	DOWN	DOWN	DOWN	NOCHANGE
NOCHANGE	NOCHANGE	NOCHANGE	NOCHANGE	NOCHANGE
NOCHANGE	NOCHANGE	NOCHANGE	NOCHANGE	NOCHANGE
DOWN	NOCHANGE	NOCHANGE	NOCHANGE	NOCHANGE
NOCHANGE	NOCHANGE	NOCHANGE	NOCHANGE	NOCHANGE
DOWN	DOWN	DOWN	DOWN	DOWN

TCGA-CZ-5986-01	TCGA-B0-4828-01.	TCGA-B8-4151-01.	TCGA-EU-5905-01	TCGA-CJ-4903-01/
NOCHANGE	NOCHANGE	UP	NOCHANGE	NOCHANGE
UP	UP	UP	UP	UP
NOCHANGE	NOCHANGE	NOCHANGE	NOCHANGE	NOCHANGE
DOWN	DOWN	DOWN	DOWN	DOWN
NOCHANGE	NOCHANGE	NOCHANGE	NOCHANGE	NOCHANGE
UP	NOCHANGE	NOCHANGE	DOWN	NOCHANGE
DOWN	NOCHANGE	DOWN	DOWN	NOCHANGE
DOWN	NOCHANGE	NOCHANGE	NOCHANGE	NOCHANGE
UP	NOCHANGE	DOWN	DOWN	NOCHANGE
DOWN	NOCHANGE	NOCHANGE	DOWN	NOCHANGE
NOCHANGE	NOCHANGE	NOCHANGE	NOCHANGE	NOCHANGE
DOWN	NOCHANGE	DOWN	NOCHANGE	DOWN
NOCHANGE	NOCHANGE	NOCHANGE	NOCHANGE	NOCHANGE
NOCHANGE	DOWN	NOCHANGE	NOCHANGE	NOCHANGE
DOWN	DOWN	DOWN	DOWN	DOWN
UP	NOCHANGE	NOCHANGE	NOCHANGE	NOCHANGE
NOCHANGE	NOCHANGE	NOCHANGE	NOCHANGE	NOCHANGE
DOWN	NOCHANGE	NOCHANGE	NOCHANGE	DOWN
UP	UP	NOCHANGE	NOCHANGE	UP
NOCHANGE	NOCHANGE	DOWN	NOCHANGE	NOCHANGE
NOCHANGE	NOCHANGE	NOCHANGE	NOCHANGE	NOCHANGE
NOCHANGE	NOCHANGE	NOCHANGE	NOCHANGE	NOCHANGE
UP	NOCHANGE	DOWN	DOWN	NOCHANGE
DOWN	NOCHANGE	NOCHANGE	DOWN	NOCHANGE
DOWN	DOWN	NOCHANGE	DOWN	DOWN
DOWN	DOWN	DOWN	NOCHANGE	NOCHANGE
NOCHANGE	UP	NOCHANGE	NOCHANGE	NOCHANGE
NOCHANGE	NOCHANGE	NOCHANGE	NOCHANGE	NOCHANGE
NOCHANGE	NOCHANGE	NOCHANGE	DOWN	NOCHANGE
NOCHANGE	NOCHANGE	NOCHANGE	DOWN	NOCHANGE
DOWN	NOCHANGE	DOWN	NOCHANGE	DOWN

TCGA-B0-5698-01.	TCGA-B0-4815-01.	TCGA-B0-5081-01.	TCGA-A3-3352-01	TCGA-A3-A6NJ-01
NOCHANGE	UP	NOCHANGE	NOCHANGE	NOCHANGE
UP	UP	UP	UP	UP
NOCHANGE	NOCHANGE	NOCHANGE	NOCHANGE	NOCHANGE
DOWN	DOWN	DOWN	DOWN	DOWN
UP	UP	NOCHANGE	NOCHANGE	NOCHANGE
UP	NOCHANGE	NOCHANGE	UP	UP
DOWN	DOWN	DOWN	NOCHANGE	NOCHANGE
NOCHANGE	NOCHANGE	NOCHANGE	DOWN	NOCHANGE
DOWN	UP	NOCHANGE	NOCHANGE	NOCHANGE
NOCHANGE	NOCHANGE	NOCHANGE	NOCHANGE	NOCHANGE
NOCHANGE	NOCHANGE	NOCHANGE	NOCHANGE	NOCHANGE
DOWN	NOCHANGE	NOCHANGE	DOWN	DOWN
NOCHANGE	NOCHANGE	NOCHANGE	NOCHANGE	NOCHANGE
NOCHANGE	NOCHANGE	NOCHANGE	NOCHANGE	NOCHANGE
DOWN	DOWN	NOCHANGE	DOWN	DOWN
NOCHANGE	UP	NOCHANGE	NOCHANGE	NOCHANGE
UP	NOCHANGE	NOCHANGE	NOCHANGE	UP
NOCHANGE	NOCHANGE	DOWN	NOCHANGE	NOCHANGE
NOCHANGE	UP	NOCHANGE	UP	NOCHANGE
NOCHANGE	DOWN	DOWN	DOWN	DOWN
UP	UP	UP	NOCHANGE	NOCHANGE
NOCHANGE	NOCHANGE	NOCHANGE	NOCHANGE	NOCHANGE
NOCHANGE	UP	NOCHANGE	NOCHANGE	NOCHANGE
DOWN	NOCHANGE	NOCHANGE	NOCHANGE	DOWN
DOWN	DOWN	DOWN	NOCHANGE	DOWN
DOWN	UP	DOWN	NOCHANGE	DOWN
NOCHANGE	NOCHANGE	NOCHANGE	NOCHANGE	NOCHANGE
NOCHANGE	NOCHANGE	NOCHANGE	NOCHANGE	NOCHANGE
NOCHANGE	DOWN	DOWN	NOCHANGE	NOCHANGE
NOCHANGE	NOCHANGE	NOCHANGE	NOCHANGE	NOCHANGE
DOWN	DOWN	DOWN	NOCHANGE	DOWN

TCGA-BP-5184-01.	TCGA-CJ-4899-01/	TCGA-CJ-4881-01/	TCGA-CJ-4878-01/	TCGA-B0-4819-01.
NOCHANGE	NOCHANGE	UP	NOCHANGE	NOCHANGE
UP	UP	UP	UP	UP
NOCHANGE	NOCHANGE	NOCHANGE	NOCHANGE	NOCHANGE
DOWN	DOWN	DOWN	DOWN	DOWN
NOCHANGE	NOCHANGE	NOCHANGE	UP	NOCHANGE
NOCHANGE	NOCHANGE	NOCHANGE	NOCHANGE	NOCHANGE
DOWN	DOWN	NOCHANGE	NOCHANGE	DOWN
NOCHANGE	DOWN	NOCHANGE	UP	NOCHANGE
DOWN	NOCHANGE	UP	NOCHANGE	NOCHANGE
NOCHANGE	NOCHANGE	NOCHANGE	NOCHANGE	NOCHANGE
NOCHANGE	DOWN	NOCHANGE	DOWN	NOCHANGE
NOCHANGE	UP	UP	UP	NOCHANGE
NOCHANGE	NOCHANGE	NOCHANGE	NOCHANGE	NOCHANGE
NOCHANGE	NOCHANGE	NOCHANGE	NOCHANGE	NOCHANGE
NOCHANGE	NOCHANGE	NOCHANGE	DOWN	NOCHANGE
NOCHANGE	UP	NOCHANGE	NOCHANGE	NOCHANGE
NOCHANGE	UP	NOCHANGE	NOCHANGE	NOCHANGE
NOCHANGE	DOWN	NOCHANGE	NOCHANGE	DOWN
NOCHANGE	NOCHANGE	UP	UP	NOCHANGE
DOWN	NOCHANGE	NOCHANGE	DOWN	DOWN
NOCHANGE	NOCHANGE	NOCHANGE	NOCHANGE	NOCHANGE
UP	NOCHANGE	NOCHANGE	NOCHANGE	NOCHANGE
NOCHANGE	NOCHANGE	NOCHANGE	DOWN	DOWN
NOCHANGE	DOWN	DOWN	DOWN	DOWN
NOCHANGE	DOWN	DOWN	NOCHANGE	DOWN
NOCHANGE	NOCHANGE	NOCHANGE	NOCHANGE	DOWN
NOCHANGE	NOCHANGE	NOCHANGE	NOCHANGE	UP
NOCHANGE	NOCHANGE	NOCHANGE	NOCHANGE	NOCHANGE
NOCHANGE	NOCHANGE	NOCHANGE	NOCHANGE	NOCHANGE
NOCHANGE	NOCHANGE	NOCHANGE	NOCHANGE	NOCHANGE
DOWN	DOWN	DOWN	DOWN	DOWN

TCGA-B0-4710-01.	TCGA-A3-3331-01	TCGA-BP-5174-01.	TCGA-B0-5694-01.	TCGA-B0-5116-01.
NOCHANGE	NOCHANGE	UP	NOCHANGE	NOCHANGE
UP	UP	UP	UP	UP
NOCHANGE	NOCHANGE	NOCHANGE	NOCHANGE	NOCHANGE
DOWN	NOCHANGE	DOWN	DOWN	DOWN
NOCHANGE	NOCHANGE	UP	NOCHANGE	NOCHANGE
NOCHANGE	UP	UP	NOCHANGE	NOCHANGE
DOWN	NOCHANGE	DOWN	DOWN	DOWN
DOWN	DOWN	DOWN	NOCHANGE	NOCHANGE
DOWN	DOWN	NOCHANGE	DOWN	DOWN
NOCHANGE	NOCHANGE	NOCHANGE	NOCHANGE	DOWN
DOWN	DOWN	NOCHANGE	NOCHANGE	NOCHANGE
DOWN	NOCHANGE	NOCHANGE	NOCHANGE	DOWN
NOCHANGE	NOCHANGE	NOCHANGE	NOCHANGE	NOCHANGE
NOCHANGE	NOCHANGE	NOCHANGE	NOCHANGE	NOCHANGE
DOWN	DOWN	DOWN	DOWN	DOWN
NOCHANGE	NOCHANGE	DOWN	UP	NOCHANGE
NOCHANGE	NOCHANGE	NOCHANGE	NOCHANGE	NOCHANGE
DOWN	DOWN	NOCHANGE	NOCHANGE	NOCHANGE
UP	UP	NOCHANGE	NOCHANGE	UP
DOWN	NOCHANGE	DOWN	NOCHANGE	NOCHANGE
NOCHANGE	UP	NOCHANGE	NOCHANGE	NOCHANGE
NOCHANGE	UP	NOCHANGE	NOCHANGE	UP
NOCHANGE	NOCHANGE	DOWN	NOCHANGE	NOCHANGE
DOWN	DOWN	NOCHANGE	NOCHANGE	DOWN
NOCHANGE	NOCHANGE	NOCHANGE	DOWN	DOWN
DOWN	NOCHANGE	NOCHANGE	NOCHANGE	NOCHANGE
NOCHANGE	NOCHANGE	NOCHANGE	NOCHANGE	NOCHANGE
UP	NOCHANGE	NOCHANGE	NOCHANGE	NOCHANGE
NOCHANGE	NOCHANGE	DOWN	DOWN	NOCHANGE
NOCHANGE	UP	NOCHANGE	NOCHANGE	NOCHANGE
DOWN	NOCHANGE	NOCHANGE	DOWN	NOCHANGE

TCGA-DV-5567-01	TCGA-BP-5169-01.	TCGA-B0-4700-01.	TCGA-AK-3433-01	TCGA-CJ-4875-01/
DOWN	NOCHANGE	NOCHANGE	DOWN	NOCHANGE
NOCHANGE	UP	UP	NOCHANGE	UP
NOCHANGE	NOCHANGE	NOCHANGE	NOCHANGE	NOCHANGE
DOWN	DOWN	DOWN	DOWN	DOWN
DOWN	NOCHANGE	NOCHANGE	DOWN	NOCHANGE
DOWN	NOCHANGE	NOCHANGE	DOWN	NOCHANGE
DOWN	DOWN	DOWN	NOCHANGE	DOWN
DOWN	UP	NOCHANGE	DOWN	DOWN
NOCHANGE	NOCHANGE	NOCHANGE	NOCHANGE	DOWN
NOCHANGE	NOCHANGE	NOCHANGE	UP	NOCHANGE
NOCHANGE	NOCHANGE	NOCHANGE	NOCHANGE	UP
NOCHANGE	NOCHANGE	NOCHANGE	DOWN	DOWN
NOCHANGE	NOCHANGE	NOCHANGE	DOWN	NOCHANGE
NOCHANGE	DOWN	NOCHANGE	DOWN	DOWN
NOCHANGE	DOWN	NOCHANGE	DOWN	DOWN
DOWN	NOCHANGE	NOCHANGE	NOCHANGE	NOCHANGE
NOCHANGE	DOWN	NOCHANGE	NOCHANGE	NOCHANGE
DOWN	DOWN	NOCHANGE	DOWN	DOWN
UP	NOCHANGE	NOCHANGE	NOCHANGE	NOCHANGE
DOWN	NOCHANGE	DOWN	UP	DOWN
NOCHANGE	NOCHANGE	NOCHANGE	NOCHANGE	NOCHANGE
NOCHANGE	NOCHANGE	NOCHANGE	NOCHANGE	NOCHANGE
DOWN	DOWN	NOCHANGE	DOWN	DOWN
NOCHANGE	NOCHANGE	NOCHANGE	NOCHANGE	DOWN
DOWN	DOWN	DOWN	NOCHANGE	DOWN
NOCHANGE	NOCHANGE	NOCHANGE	NOCHANGE	NOCHANGE
NOCHANGE	NOCHANGE	NOCHANGE	UP	NOCHANGE
NOCHANGE	NOCHANGE	NOCHANGE	DOWN	NOCHANGE
NOCHANGE	DOWN	DOWN	DOWN	DOWN
NOCHANGE	NOCHANGE	NOCHANGE	DOWN	DOWN
DOWN	DOWN	NOCHANGE	UP	DOWN

TCGA-A3-3335-01	TCGA-B0-5115-01.	TCGA-BP-5195-01.	TCGA-BP-4354-01.	TCGA-BP-4974-01.
NOCHANGE	NOCHANGE	NOCHANGE	UP	NOCHANGE
UP	UP	UP	NOCHANGE	UP
NOCHANGE	NOCHANGE	NOCHANGE	NOCHANGE	NOCHANGE
DOWN	DOWN	DOWN	DOWN	DOWN
UP	NOCHANGE	UP	UP	NOCHANGE
UP	NOCHANGE	NOCHANGE	DOWN	NOCHANGE
DOWN	DOWN	DOWN	NOCHANGE	NOCHANGE
NOCHANGE	DOWN	NOCHANGE	NOCHANGE	NOCHANGE
NOCHANGE	DOWN	NOCHANGE	NOCHANGE	NOCHANGE
NOCHANGE	NOCHANGE	DOWN	NOCHANGE	NOCHANGE
DOWN	NOCHANGE	NOCHANGE	NOCHANGE	NOCHANGE
NOCHANGE	NOCHANGE	NOCHANGE	NOCHANGE	UP
NOCHANGE	NOCHANGE	NOCHANGE	DOWN	NOCHANGE
DOWN	NOCHANGE	NOCHANGE	UP	NOCHANGE
DOWN	DOWN	DOWN	DOWN	NOCHANGE
NOCHANGE	NOCHANGE	NOCHANGE	NOCHANGE	NOCHANGE
NOCHANGE	NOCHANGE	NOCHANGE	UP	NOCHANGE
NOCHANGE	DOWN	DOWN	DOWN	DOWN
NOCHANGE	UP	NOCHANGE	UP	NOCHANGE
NOCHANGE	NOCHANGE	NOCHANGE	DOWN	DOWN
NOCHANGE	NOCHANGE	UP	UP	NOCHANGE
UP	NOCHANGE	UP	NOCHANGE	NOCHANGE
NOCHANGE	NOCHANGE	NOCHANGE	UP	DOWN
NOCHANGE	DOWN	DOWN	DOWN	DOWN
NOCHANGE	DOWN	DOWN	DOWN	DOWN
NOCHANGE	DOWN	NOCHANGE	NOCHANGE	NOCHANGE
NOCHANGE	NOCHANGE	NOCHANGE	NOCHANGE	NOCHANGE
NOCHANGE	NOCHANGE	NOCHANGE	NOCHANGE	NOCHANGE
NOCHANGE	NOCHANGE	NOCHANGE	NOCHANGE	NOCHANGE
NOCHANGE	NOCHANGE	NOCHANGE	NOCHANGE	NOCHANGE
NOCHANGE	DOWN	DOWN	DOWN	DOWN

TCGA-CJ-4641-01A	TCGA-B4-5834-01.	TCGA-BP-4169-01.	TCGA-B2-5633-01.	TCGA-BP-4777-01.
NOCHANGE	NOCHANGE	NOCHANGE	NOCHANGE	NOCHANGE
UP	NOCHANGE	UP	UP	UP
NOCHANGE	NOCHANGE	NOCHANGE	NOCHANGE	NOCHANGE
DOWN	DOWN	DOWN	UP	DOWN
UP	NOCHANGE	UP	UP	NOCHANGE
NOCHANGE	NOCHANGE	NOCHANGE	NOCHANGE	NOCHANGE
NOCHANGE	NOCHANGE	DOWN	DOWN	DOWN
NOCHANGE	NOCHANGE	NOCHANGE	NOCHANGE	NOCHANGE
UP	DOWN	NOCHANGE	NOCHANGE	NOCHANGE
NOCHANGE	DOWN	DOWN	NOCHANGE	NOCHANGE
NOCHANGE	NOCHANGE	NOCHANGE	NOCHANGE	NOCHANGE
NOCHANGE	DOWN	NOCHANGE	NOCHANGE	DOWN
NOCHANGE	NOCHANGE	NOCHANGE	NOCHANGE	NOCHANGE
NOCHANGE	NOCHANGE	NOCHANGE	UP	NOCHANGE
DOWN	DOWN	NOCHANGE	DOWN	DOWN
NOCHANGE	NOCHANGE	NOCHANGE	UP	UP
UP	NOCHANGE	NOCHANGE	NOCHANGE	NOCHANGE
NOCHANGE	DOWN	DOWN	DOWN	DOWN
NOCHANGE	NOCHANGE	NOCHANGE	UP	NOCHANGE
DOWN	DOWN	DOWN	DOWN	NOCHANGE
UP	NOCHANGE	UP	UP	UP
NOCHANGE	UP	UP	NOCHANGE	NOCHANGE
NOCHANGE	DOWN	NOCHANGE	NOCHANGE	NOCHANGE
NOCHANGE	DOWN	DOWN	DOWN	NOCHANGE
DOWN	NOCHANGE	DOWN	DOWN	DOWN
NOCHANGE	NOCHANGE	NOCHANGE	DOWN	NOCHANGE
NOCHANGE	NOCHANGE	NOCHANGE	NOCHANGE	NOCHANGE
NOCHANGE	NOCHANGE	UP	DOWN	NOCHANGE
NOCHANGE	DOWN	NOCHANGE	NOCHANGE	NOCHANGE
NOCHANGE	NOCHANGE	NOCHANGE	NOCHANGE	NOCHANGE
NOCHANGE	DOWN	DOWN	DOWN	DOWN

TCGA-B0-5399-01.	TCGA-B0-5099-01.	TCGA-BP-4166-01.	TCGA-CJ-4890-01/	TCGA-B0-5084-01.
NOCHANGE	NOCHANGE	NOCHANGE	NOCHANGE	NOCHANGE
UP	UP	UP	UP	NOCHANGE
NOCHANGE	NOCHANGE	NOCHANGE	NOCHANGE	DOWN
DOWN	DOWN	NOCHANGE	DOWN	DOWN
NOCHANGE	NOCHANGE	NOCHANGE	UP	NOCHANGE
NOCHANGE	NOCHANGE	NOCHANGE	NOCHANGE	DOWN
NOCHANGE	NOCHANGE	DOWN	NOCHANGE	NOCHANGE
DOWN	NOCHANGE	NOCHANGE	NOCHANGE	NOCHANGE
NOCHANGE	NOCHANGE	NOCHANGE	UP	NOCHANGE
NOCHANGE	NOCHANGE	DOWN	NOCHANGE	NOCHANGE
NOCHANGE	NOCHANGE	DOWN	DOWN	NOCHANGE
NOCHANGE	NOCHANGE	DOWN	NOCHANGE	NOCHANGE
NOCHANGE	NOCHANGE	NOCHANGE	NOCHANGE	NOCHANGE
NOCHANGE	NOCHANGE	NOCHANGE	NOCHANGE	DOWN
DOWN	DOWN	DOWN	NOCHANGE	DOWN
NOCHANGE	NOCHANGE	NOCHANGE	NOCHANGE	DOWN
UP	NOCHANGE	NOCHANGE	NOCHANGE	DOWN
NOCHANGE	NOCHANGE	NOCHANGE	DOWN	DOWN
UP	NOCHANGE	NOCHANGE	NOCHANGE	NOCHANGE
NOCHANGE	DOWN	DOWN	NOCHANGE	DOWN
NOCHANGE	NOCHANGE	NOCHANGE	UP	NOCHANGE
NOCHANGE	NOCHANGE	UP	NOCHANGE	UP
NOCHANGE	DOWN	DOWN	UP	UP
NOCHANGE	NOCHANGE	NOCHANGE	DOWN	NOCHANGE
NOCHANGE	NOCHANGE	DOWN	DOWN	DOWN
NOCHANGE	NOCHANGE	NOCHANGE	NOCHANGE	UP
NOCHANGE	NOCHANGE	NOCHANGE	NOCHANGE	NOCHANGE
NOCHANGE	NOCHANGE	NOCHANGE	NOCHANGE	NOCHANGE
NOCHANGE	NOCHANGE	NOCHANGE	NOCHANGE	NOCHANGE
NOCHANGE	NOCHANGE	NOCHANGE	NOCHANGE	DOWN
NOCHANGE	DOWN	DOWN	DOWN	NOCHANGE

TCGA-BP-5168-01.	TCGA-BP-4770-01.	TCGA-BP-4332-01.	TCGA-B4-5832-01.	TCGA-B0-4849-01.
NOCHANGE	NOCHANGE	NOCHANGE	UP	NOCHANGE
UP	UP	UP	NOCHANGE	UP
NOCHANGE	NOCHANGE	NOCHANGE	NOCHANGE	NOCHANGE
DOWN	DOWN	NOCHANGE	DOWN	DOWN
NOCHANGE	DOWN	NOCHANGE	UP	NOCHANGE
NOCHANGE	DOWN	NOCHANGE	NOCHANGE	NOCHANGE
NOCHANGE	NOCHANGE	DOWN	DOWN	NOCHANGE
DOWN	NOCHANGE	NOCHANGE	NOCHANGE	NOCHANGE
NOCHANGE	NOCHANGE	NOCHANGE	NOCHANGE	NOCHANGE
NOCHANGE	NOCHANGE	NOCHANGE	DOWN	NOCHANGE
NOCHANGE	NOCHANGE	NOCHANGE	UP	NOCHANGE
NOCHANGE	NOCHANGE	NOCHANGE	NOCHANGE	NOCHANGE
NOCHANGE	DOWN	NOCHANGE	NOCHANGE	NOCHANGE
NOCHANGE	NOCHANGE	NOCHANGE	NOCHANGE	NOCHANGE
DOWN	DOWN	DOWN	DOWN	DOWN
UP	DOWN	NOCHANGE	UP	NOCHANGE
NOCHANGE	DOWN	NOCHANGE	DOWN	NOCHANGE
NOCHANGE	DOWN	DOWN	DOWN	NOCHANGE
NOCHANGE	DOWN	NOCHANGE	UP	NOCHANGE
DOWN	DOWN	DOWN	UP	NOCHANGE
NOCHANGE	NOCHANGE	NOCHANGE	UP	NOCHANGE
NOCHANGE	NOCHANGE	NOCHANGE	NOCHANGE	NOCHANGE
NOCHANGE	NOCHANGE	NOCHANGE	NOCHANGE	DOWN
NOCHANGE	NOCHANGE	NOCHANGE	DOWN	DOWN
NOCHANGE	DOWN	DOWN	NOCHANGE	DOWN
NOCHANGE	DOWN	NOCHANGE	NOCHANGE	DOWN
NOCHANGE	NOCHANGE	NOCHANGE	NOCHANGE	UP
NOCHANGE	NOCHANGE	NOCHANGE	DOWN	NOCHANGE
NOCHANGE	DOWN	NOCHANGE	NOCHANGE	NOCHANGE
NOCHANGE	NOCHANGE	NOCHANGE	NOCHANGE	NOCHANGE
DOWN	DOWN	DOWN	NOCHANGE	DOWN

TCGA-B8-5164-01	TCGA-CW-6093-01	TCGA-MM-A84U-(	TCGA-CJ-4637-01/	TCGA-CJ-4642-01I
NOCHANGE	NOCHANGE	NOCHANGE	NOCHANGE	NOCHANGE
UP	UP	UP	UP	NOCHANGE
NOCHANGE	NOCHANGE	NOCHANGE	NOCHANGE	NOCHANGE
DOWN	DOWN	DOWN	DOWN	DOWN
UP	NOCHANGE	NOCHANGE	NOCHANGE	DOWN
NOCHANGE	NOCHANGE	NOCHANGE	NOCHANGE	NOCHANGE
DOWN	DOWN	DOWN	DOWN	NOCHANGE
DOWN	NOCHANGE	NOCHANGE	DOWN	NOCHANGE
NOCHANGE	UP	NOCHANGE	NOCHANGE	UP
NOCHANGE	NOCHANGE	NOCHANGE	NOCHANGE	UP
NOCHANGE	NOCHANGE	NOCHANGE	NOCHANGE	UP
NOCHANGE	NOCHANGE	NOCHANGE	UP	UP
NOCHANGE	NOCHANGE	NOCHANGE	NOCHANGE	NOCHANGE
NOCHANGE	NOCHANGE	NOCHANGE	NOCHANGE	DOWN
DOWN	DOWN	NOCHANGE	DOWN	NOCHANGE
NOCHANGE	NOCHANGE	DOWN	NOCHANGE	NOCHANGE
UP	UP	NOCHANGE	UP	DOWN
DOWN	DOWN	NOCHANGE	DOWN	NOCHANGE
NOCHANGE	NOCHANGE	NOCHANGE	NOCHANGE	NOCHANGE
NOCHANGE	DOWN	DOWN	DOWN	DOWN
UP	NOCHANGE	NOCHANGE	UP	NOCHANGE
UP	NOCHANGE	NOCHANGE	UP	NOCHANGE
NOCHANGE	NOCHANGE	DOWN	NOCHANGE	DOWN
DOWN	DOWN	NOCHANGE	DOWN	NOCHANGE
NOCHANGE	DOWN	DOWN	DOWN	DOWN
DOWN	NOCHANGE	NOCHANGE	NOCHANGE	NOCHANGE
NOCHANGE	NOCHANGE	NOCHANGE	NOCHANGE	UP
NOCHANGE	NOCHANGE	NOCHANGE	NOCHANGE	NOCHANGE
NOCHANGE	NOCHANGE	DOWN	NOCHANGE	DOWN
NOCHANGE	NOCHANGE	NOCHANGE	NOCHANGE	NOCHANGE
DOWN	DOWN	NOCHANGE	DOWN	DOWN

TCGA-BP-5178-01.	TCGA-BP-4790-01.	TCGA-B0-5692-01.	TCGA-CJ-4885-01/	TCGA-BP-4768-01.
UP	NOCHANGE	NOCHANGE	NOCHANGE	NOCHANGE
UP	UP	UP	UP	UP
NOCHANGE	NOCHANGE	NOCHANGE	NOCHANGE	NOCHANGE
DOWN	NOCHANGE	DOWN	DOWN	DOWN
UP	NOCHANGE	NOCHANGE	UP	NOCHANGE
UP	NOCHANGE	NOCHANGE	NOCHANGE	NOCHANGE
NOCHANGE	NOCHANGE	DOWN	DOWN	DOWN
UP	DOWN	DOWN	NOCHANGE	NOCHANGE
NOCHANGE	NOCHANGE	DOWN	DOWN	NOCHANGE
NOCHANGE	DOWN	NOCHANGE	NOCHANGE	NOCHANGE
NOCHANGE	NOCHANGE	NOCHANGE	DOWN	DOWN
NOCHANGE	NOCHANGE	DOWN	DOWN	UP
NOCHANGE	NOCHANGE	NOCHANGE	NOCHANGE	NOCHANGE
DOWN	NOCHANGE	NOCHANGE	NOCHANGE	NOCHANGE
DOWN	NOCHANGE	DOWN	DOWN	DOWN
NOCHANGE	NOCHANGE	NOCHANGE	UP	NOCHANGE
NOCHANGE	NOCHANGE	NOCHANGE	NOCHANGE	NOCHANGE
NOCHANGE	DOWN	NOCHANGE	NOCHANGE	NOCHANGE
NOCHANGE	UP	NOCHANGE	NOCHANGE	UP
UP	DOWN	DOWN	NOCHANGE	UP
NOCHANGE	UP	NOCHANGE	NOCHANGE	NOCHANGE
NOCHANGE	UP	UP	UP	UP
UP	NOCHANGE	NOCHANGE	NOCHANGE	NOCHANGE
NOCHANGE	DOWN	NOCHANGE	DOWN	DOWN
DOWN	DOWN	NOCHANGE	NOCHANGE	DOWN
UP	DOWN	NOCHANGE	NOCHANGE	DOWN
NOCHANGE	NOCHANGE	NOCHANGE	NOCHANGE	NOCHANGE
NOCHANGE	NOCHANGE	NOCHANGE	NOCHANGE	NOCHANGE
DOWN	NOCHANGE	NOCHANGE	NOCHANGE	NOCHANGE
NOCHANGE	NOCHANGE	NOCHANGE	NOCHANGE	NOCHANGE
UP	DOWN	NOCHANGE	DOWN	DOWN

TCGA-B0-5713-01.	TCGA-CW-5587-01	TCGA-BP-5201-01.	TCGA-CW-5590-01	TCGA-A3-3370-01
NOCHANGE	NOCHANGE	NOCHANGE	NOCHANGE	NOCHANGE
UP	UP	UP	UP	UP
NOCHANGE	NOCHANGE	NOCHANGE	NOCHANGE	NOCHANGE
DOWN	NOCHANGE	DOWN	DOWN	NOCHANGE
NOCHANGE	NOCHANGE	NOCHANGE	NOCHANGE	NOCHANGE
NOCHANGE	NOCHANGE	NOCHANGE	NOCHANGE	NOCHANGE
DOWN	DOWN	NOCHANGE	NOCHANGE	DOWN
DOWN	DOWN	NOCHANGE	NOCHANGE	NOCHANGE
DOWN	NOCHANGE	NOCHANGE	NOCHANGE	NOCHANGE
NOCHANGE	NOCHANGE	NOCHANGE	NOCHANGE	NOCHANGE
NOCHANGE	DOWN	NOCHANGE	NOCHANGE	NOCHANGE
NOCHANGE	NOCHANGE	UP	NOCHANGE	NOCHANGE
NOCHANGE	NOCHANGE	NOCHANGE	NOCHANGE	NOCHANGE
NOCHANGE	NOCHANGE	DOWN	NOCHANGE	NOCHANGE
DOWN	DOWN	NOCHANGE	DOWN	DOWN
NOCHANGE	NOCHANGE	NOCHANGE	NOCHANGE	UP
UP	NOCHANGE	NOCHANGE	NOCHANGE	NOCHANGE
NOCHANGE	NOCHANGE	NOCHANGE	DOWN	DOWN
UP	NOCHANGE	NOCHANGE	NOCHANGE	NOCHANGE
DOWN	NOCHANGE	NOCHANGE	NOCHANGE	DOWN
NOCHANGE	UP	NOCHANGE	NOCHANGE	UP
UP	NOCHANGE	UP	UP	NOCHANGE
NOCHANGE	NOCHANGE	DOWN	NOCHANGE	DOWN
NOCHANGE	NOCHANGE	DOWN	NOCHANGE	DOWN
NOCHANGE	NOCHANGE	DOWN	DOWN	NOCHANGE
NOCHANGE	DOWN	NOCHANGE	NOCHANGE	DOWN
NOCHANGE	NOCHANGE	NOCHANGE	NOCHANGE	UP
NOCHANGE	NOCHANGE	NOCHANGE	NOCHANGE	NOCHANGE
NOCHANGE	NOCHANGE	NOCHANGE	NOCHANGE	DOWN
NOCHANGE	NOCHANGE	NOCHANGE	NOCHANGE	NOCHANGE
DOWN	NOCHANGE	DOWN	DOWN	DOWN

TCGA-B0-4848-01.	TCGA-B0-4698-01.	TCGA-BP-4982-01.	TCGA-CJ-4636-01/	TCGA-AK-3455-01
NOCHANGE	NOCHANGE	NOCHANGE	NOCHANGE	NOCHANGE
UP	NOCHANGE	UP	UP	UP
NOCHANGE	NOCHANGE	NOCHANGE	NOCHANGE	NOCHANGE
DOWN	DOWN	NOCHANGE	DOWN	DOWN
UP	DOWN	NOCHANGE	NOCHANGE	UP
UP	DOWN	NOCHANGE	NOCHANGE	NOCHANGE
NOCHANGE	NOCHANGE	DOWN	NOCHANGE	NOCHANGE
NOCHANGE	NOCHANGE	NOCHANGE	NOCHANGE	NOCHANGE
NOCHANGE	UP	NOCHANGE	UP	UP
DOWN	NOCHANGE	NOCHANGE	NOCHANGE	NOCHANGE
NOCHANGE	NOCHANGE	DOWN	NOCHANGE	UP
DOWN	NOCHANGE	DOWN	NOCHANGE	DOWN
NOCHANGE	DOWN	NOCHANGE	NOCHANGE	NOCHANGE
NOCHANGE	NOCHANGE	NOCHANGE	NOCHANGE	NOCHANGE
NOCHANGE	DOWN	DOWN	NOCHANGE	DOWN
NOCHANGE	DOWN	NOCHANGE	NOCHANGE	NOCHANGE
NOCHANGE	NOCHANGE	NOCHANGE	NOCHANGE	NOCHANGE
DOWN	DOWN	DOWN	DOWN	NOCHANGE
NOCHANGE	NOCHANGE	UP	NOCHANGE	NOCHANGE
NOCHANGE	DOWN	DOWN	DOWN	NOCHANGE
UP	UP	UP	UP	NOCHANGE
UP	UP	UP	NOCHANGE	NOCHANGE
NOCHANGE	UP	NOCHANGE	NOCHANGE	DOWN
NOCHANGE	DOWN	DOWN	DOWN	NOCHANGE
DOWN	DOWN	DOWN	DOWN	NOCHANGE
NOCHANGE	UP	NOCHANGE	NOCHANGE	DOWN
NOCHANGE	NOCHANGE	NOCHANGE	NOCHANGE	NOCHANGE
NOCHANGE	NOCHANGE	NOCHANGE	NOCHANGE	NOCHANGE
NOCHANGE	DOWN	NOCHANGE	NOCHANGE	DOWN
DOWN	NOCHANGE	NOCHANGE	NOCHANGE	NOCHANGE
NOCHANGE	DOWN	DOWN	DOWN	NOCHANGE

TCGA-A3-A6NN-0	TCGA-G6-A8L6-01	TCGA-B0-5703-01.	TCGA-BP-4176-01.	TCGA-B0-4707-01.
NOCHANGE	NOCHANGE	NOCHANGE	NOCHANGE	UP
UP	UP	UP	UP	UP
NOCHANGE	NOCHANGE	NOCHANGE	NOCHANGE	NOCHANGE
DOWN	DOWN	DOWN	DOWN	DOWN
NOCHANGE	NOCHANGE	NOCHANGE	UP	NOCHANGE
NOCHANGE	NOCHANGE	NOCHANGE	NOCHANGE	NOCHANGE
NOCHANGE	DOWN	DOWN	DOWN	DOWN
NOCHANGE	NOCHANGE	NOCHANGE	NOCHANGE	NOCHANGE
NOCHANGE	DOWN	DOWN	UP	UP
NOCHANGE	NOCHANGE	NOCHANGE	NOCHANGE	NOCHANGE
NOCHANGE	NOCHANGE	NOCHANGE	NOCHANGE	NOCHANGE
NOCHANGE	DOWN	DOWN	DOWN	UP
NOCHANGE	NOCHANGE	NOCHANGE	NOCHANGE	NOCHANGE
NOCHANGE	DOWN	NOCHANGE	NOCHANGE	NOCHANGE
DOWN	DOWN	NOCHANGE	DOWN	DOWN
NOCHANGE	NOCHANGE	NOCHANGE	NOCHANGE	NOCHANGE
NOCHANGE	NOCHANGE	NOCHANGE	NOCHANGE	DOWN
DOWN	NOCHANGE	NOCHANGE	NOCHANGE	NOCHANGE
NOCHANGE	NOCHANGE	NOCHANGE	NOCHANGE	NOCHANGE
NOCHANGE	DOWN	NOCHANGE	NOCHANGE	UP
NOCHANGE	NOCHANGE	NOCHANGE	UP	NOCHANGE
UP	NOCHANGE	UP	NOCHANGE	NOCHANGE
NOCHANGE	DOWN	DOWN	UP	UP
DOWN	NOCHANGE	NOCHANGE	NOCHANGE	DOWN
NOCHANGE	DOWN	NOCHANGE	DOWN	DOWN
NOCHANGE	NOCHANGE	DOWN	NOCHANGE	UP
NOCHANGE	NOCHANGE	NOCHANGE	NOCHANGE	NOCHANGE
NOCHANGE	NOCHANGE	NOCHANGE	NOCHANGE	NOCHANGE
NOCHANGE	DOWN	NOCHANGE	NOCHANGE	DOWN
NOCHANGE	NOCHANGE	NOCHANGE	NOCHANGE	DOWN
DOWN	DOWN	NOCHANGE	DOWN	DOWN

TCGA-CW-5591-01	TCGA-B4-5838-01.	TCGA-BP-4775-01.	TCGA-BP-4158-01.	TCGA-CJ-5676-01/
NOCHANGE	NOCHANGE	NOCHANGE	NOCHANGE	NOCHANGE
NOCHANGE	UP	UP	UP	UP
NOCHANGE	NOCHANGE	NOCHANGE	NOCHANGE	NOCHANGE
NOCHANGE	DOWN	NOCHANGE	DOWN	DOWN
UP	UP	NOCHANGE	NOCHANGE	UP
UP	UP	NOCHANGE	NOCHANGE	NOCHANGE
DOWN	NOCHANGE	DOWN	DOWN	DOWN
NOCHANGE	NOCHANGE	NOCHANGE	NOCHANGE	NOCHANGE
NOCHANGE	NOCHANGE	NOCHANGE	NOCHANGE	DOWN
NOCHANGE	DOWN	NOCHANGE	NOCHANGE	NOCHANGE
DOWN	NOCHANGE	NOCHANGE	NOCHANGE	NOCHANGE
NOCHANGE	DOWN	NOCHANGE	UP	NOCHANGE
NOCHANGE	NOCHANGE	NOCHANGE	NOCHANGE	NOCHANGE
UP	UP	NOCHANGE	NOCHANGE	DOWN
NOCHANGE	DOWN	DOWN	DOWN	DOWN
NOCHANGE	NOCHANGE	NOCHANGE	NOCHANGE	NOCHANGE
UP	NOCHANGE	NOCHANGE	NOCHANGE	NOCHANGE
NOCHANGE	DOWN	DOWN	NOCHANGE	NOCHANGE
NOCHANGE	NOCHANGE	NOCHANGE	NOCHANGE	NOCHANGE
NOCHANGE	NOCHANGE	NOCHANGE	DOWN	UP
NOCHANGE	NOCHANGE	UP	NOCHANGE	NOCHANGE
UP	NOCHANGE	NOCHANGE	NOCHANGE	NOCHANGE
UP	NOCHANGE	DOWN	NOCHANGE	DOWN
DOWN	NOCHANGE	DOWN	NOCHANGE	NOCHANGE
NOCHANGE	NOCHANGE	DOWN	DOWN	DOWN
NOCHANGE	NOCHANGE	DOWN	NOCHANGE	NOCHANGE
NOCHANGE	NOCHANGE	NOCHANGE	NOCHANGE	NOCHANGE
NOCHANGE	DOWN	NOCHANGE	NOCHANGE	NOCHANGE
NOCHANGE	NOCHANGE	NOCHANGE	DOWN	NOCHANGE
NOCHANGE	DOWN	NOCHANGE	NOCHANGE	NOCHANGE
NOCHANGE	UP	DOWN	DOWN	NOCHANGE

TCGA-DV-5565-01	TCGA-BP-4163-01.	TCGA-BP-4327-01.	TCGA-BP-5182-01.	TCGA-CJ-4884-01/
NOCHANGE	UP	NOCHANGE	NOCHANGE	NOCHANGE
UP	UP	UP	UP	UP
NOCHANGE	NOCHANGE	NOCHANGE	NOCHANGE	NOCHANGE
DOWN	NOCHANGE	DOWN	DOWN	DOWN
NOCHANGE	UP	NOCHANGE	NOCHANGE	UP
NOCHANGE	UP	NOCHANGE	NOCHANGE	NOCHANGE
NOCHANGE	DOWN	DOWN	NOCHANGE	DOWN
NOCHANGE	NOCHANGE	NOCHANGE	NOCHANGE	NOCHANGE
DOWN	NOCHANGE	NOCHANGE	NOCHANGE	NOCHANGE
NOCHANGE	NOCHANGE	NOCHANGE	NOCHANGE	NOCHANGE
NOCHANGE	DOWN	NOCHANGE	NOCHANGE	NOCHANGE
DOWN	NOCHANGE	NOCHANGE	NOCHANGE	DOWN
NOCHANGE	NOCHANGE	NOCHANGE	NOCHANGE	NOCHANGE
DOWN	NOCHANGE	DOWN	NOCHANGE	NOCHANGE
NOCHANGE	DOWN	DOWN	NOCHANGE	DOWN
NOCHANGE	NOCHANGE	NOCHANGE	NOCHANGE	NOCHANGE
NOCHANGE	UP	NOCHANGE	NOCHANGE	NOCHANGE
NOCHANGE	DOWN	DOWN	DOWN	DOWN
NOCHANGE	UP	NOCHANGE	NOCHANGE	NOCHANGE
DOWN	NOCHANGE	DOWN	DOWN	DOWN
NOCHANGE	UP	NOCHANGE	UP	UP
NOCHANGE	NOCHANGE	UP	NOCHANGE	NOCHANGE
NOCHANGE	NOCHANGE	DOWN	NOCHANGE	NOCHANGE
DOWN	NOCHANGE	NOCHANGE	DOWN	DOWN
DOWN	NOCHANGE	DOWN	DOWN	DOWN
NOCHANGE	NOCHANGE	NOCHANGE	NOCHANGE	NOCHANGE
NOCHANGE	NOCHANGE	NOCHANGE	NOCHANGE	NOCHANGE
NOCHANGE	NOCHANGE	NOCHANGE	NOCHANGE	NOCHANGE
NOCHANGE	NOCHANGE	DOWN	NOCHANGE	DOWN
DOWN	DOWN	NOCHANGE	NOCHANGE	NOCHANGE
DOWN	NOCHANGE	DOWN	DOWN	DOWN

TCGA-CZ-5459-01	TCGA-B0-5096-01.	TCGA-B8-A54J-01.	TCGA-BP-4987-01.	TCGA-B2-5641-01.
NOCHANGE	NOCHANGE	NOCHANGE	NOCHANGE	NOCHANGE
UP	UP	UP	UP	UP
NOCHANGE	NOCHANGE	NOCHANGE	NOCHANGE	NOCHANGE
DOWN	DOWN	DOWN	DOWN	DOWN
UP	UP	NOCHANGE	NOCHANGE	UP
NOCHANGE	NOCHANGE	NOCHANGE	NOCHANGE	NOCHANGE
DOWN	NOCHANGE	NOCHANGE	DOWN	DOWN
NOCHANGE	NOCHANGE	NOCHANGE	NOCHANGE	NOCHANGE
DOWN	NOCHANGE	DOWN	DOWN	NOCHANGE
NOCHANGE	NOCHANGE	NOCHANGE	NOCHANGE	NOCHANGE
NOCHANGE	NOCHANGE	NOCHANGE	DOWN	DOWN
NOCHANGE	NOCHANGE	NOCHANGE	DOWN	UP
NOCHANGE	DOWN	NOCHANGE	NOCHANGE	NOCHANGE
NOCHANGE	NOCHANGE	NOCHANGE	NOCHANGE	NOCHANGE
NOCHANGE	DOWN	NOCHANGE	DOWN	DOWN
NOCHANGE	NOCHANGE	NOCHANGE	UP	NOCHANGE
NOCHANGE	NOCHANGE	UP	NOCHANGE	NOCHANGE
DOWN	NOCHANGE	NOCHANGE	DOWN	DOWN
NOCHANGE	NOCHANGE	NOCHANGE	UP	NOCHANGE
DOWN	DOWN	DOWN	DOWN	NOCHANGE
NOCHANGE	UP	NOCHANGE	NOCHANGE	UP
NOCHANGE	NOCHANGE	NOCHANGE	UP	UP
DOWN	UP	NOCHANGE	NOCHANGE	NOCHANGE
NOCHANGE	NOCHANGE	DOWN	DOWN	NOCHANGE
DOWN	DOWN	DOWN	NOCHANGE	DOWN
NOCHANGE	NOCHANGE	NOCHANGE	NOCHANGE	DOWN
NOCHANGE	NOCHANGE	NOCHANGE	NOCHANGE	NOCHANGE
NOCHANGE	NOCHANGE	NOCHANGE	NOCHANGE	NOCHANGE
DOWN	NOCHANGE	NOCHANGE	DOWN	DOWN
NOCHANGE	DOWN	NOCHANGE	NOCHANGE	DOWN
DOWN	DOWN	DOWN	NOCHANGE	NOCHANGE

TCGA-AK-3426-01	TCGA-B4-5377-01.	TCGA-BP-4342-01.	TCGA-BP-4983-01.	TCGA-B0-4833-01.
NOCHANGE	NOCHANGE	NOCHANGE	UP	NOCHANGE
UP	UP	UP	UP	UP
NOCHANGE	NOCHANGE	NOCHANGE	NOCHANGE	NOCHANGE
DOWN	DOWN	DOWN	DOWN	DOWN
NOCHANGE	NOCHANGE	NOCHANGE	NOCHANGE	NOCHANGE
DOWN	NOCHANGE	NOCHANGE	DOWN	NOCHANGE
DOWN	DOWN	NOCHANGE	NOCHANGE	NOCHANGE
NOCHANGE	NOCHANGE	NOCHANGE	NOCHANGE	NOCHANGE
NOCHANGE	UP	NOCHANGE	NOCHANGE	NOCHANGE
NOCHANGE	NOCHANGE	NOCHANGE	NOCHANGE	NOCHANGE
UP	NOCHANGE	NOCHANGE	NOCHANGE	NOCHANGE
UP	DOWN	NOCHANGE	NOCHANGE	NOCHANGE
DOWN	NOCHANGE	NOCHANGE	DOWN	NOCHANGE
DOWN	NOCHANGE	NOCHANGE	NOCHANGE	NOCHANGE
NOCHANGE	DOWN	DOWN	DOWN	DOWN
NOCHANGE	NOCHANGE	NOCHANGE	NOCHANGE	NOCHANGE
DOWN	UP	NOCHANGE	UP	NOCHANGE
DOWN	NOCHANGE	DOWN	DOWN	NOCHANGE
NOCHANGE	NOCHANGE	NOCHANGE	NOCHANGE	NOCHANGE
DOWN	NOCHANGE	UP	DOWN	DOWN
NOCHANGE	UP	NOCHANGE	UP	NOCHANGE
NOCHANGE	UP	NOCHANGE	NOCHANGE	NOCHANGE
DOWN	NOCHANGE	NOCHANGE	UP	DOWN
NOCHANGE	NOCHANGE	NOCHANGE	DOWN	NOCHANGE
DOWN	NOCHANGE	DOWN	DOWN	DOWN
NOCHANGE	NOCHANGE	DOWN	DOWN	NOCHANGE
NOCHANGE	NOCHANGE	NOCHANGE	NOCHANGE	NOCHANGE
NOCHANGE	NOCHANGE	NOCHANGE	NOCHANGE	NOCHANGE
DOWN	NOCHANGE	NOCHANGE	NOCHANGE	DOWN
NOCHANGE	NOCHANGE	NOCHANGE	NOCHANGE	NOCHANGE
DOWN	DOWN	NOCHANGE	NOCHANGE	DOWN

TCGA-AS-3777-01	TCGA-CJ-4874-01/	TCGA-CJ-4887-01/	TCGA-AK-3428-01	TCGA-B8-A54F-01
UP	NOCHANGE	NOCHANGE	NOCHANGE	NOCHANGE
NOCHANGE	UP	UP	UP	UP
NOCHANGE	NOCHANGE	NOCHANGE	NOCHANGE	NOCHANGE
DOWN	DOWN	DOWN	DOWN	DOWN
NOCHANGE	UP	NOCHANGE	UP	UP
NOCHANGE	UP	NOCHANGE	UP	NOCHANGE
NOCHANGE	NOCHANGE	DOWN	DOWN	NOCHANGE
NOCHANGE	NOCHANGE	NOCHANGE	NOCHANGE	NOCHANGE
UP	NOCHANGE	UP	NOCHANGE	NOCHANGE
NOCHANGE	NOCHANGE	NOCHANGE	NOCHANGE	NOCHANGE
DOWN	NOCHANGE	NOCHANGE	NOCHANGE	NOCHANGE
DOWN	NOCHANGE	DOWN	DOWN	NOCHANGE
NOCHANGE	NOCHANGE	NOCHANGE	NOCHANGE	NOCHANGE
DOWN	NOCHANGE	DOWN	NOCHANGE	NOCHANGE
DOWN	DOWN	NOCHANGE	DOWN	DOWN
UP	NOCHANGE	NOCHANGE	NOCHANGE	UP
DOWN	NOCHANGE	NOCHANGE	UP	NOCHANGE
UP	NOCHANGE	NOCHANGE	NOCHANGE	NOCHANGE
DOWN	NOCHANGE	NOCHANGE	NOCHANGE	UP
NOCHANGE	DOWN	NOCHANGE	DOWN	NOCHANGE
NOCHANGE	NOCHANGE	NOCHANGE	NOCHANGE	UP
NOCHANGE	NOCHANGE	NOCHANGE	UP	UP
NOCHANGE	NOCHANGE	DOWN	NOCHANGE	NOCHANGE
UP	NOCHANGE	DOWN	NOCHANGE	NOCHANGE
NOCHANGE	NOCHANGE	DOWN	NOCHANGE	DOWN
NOCHANGE	NOCHANGE	NOCHANGE	NOCHANGE	DOWN
NOCHANGE	NOCHANGE	NOCHANGE	NOCHANGE	NOCHANGE
DOWN	NOCHANGE	NOCHANGE	NOCHANGE	NOCHANGE
DOWN	DOWN	DOWN	NOCHANGE	NOCHANGE
DOWN	NOCHANGE	NOCHANGE	NOCHANGE	NOCHANGE
DOWN	NOCHANGE	DOWN	NOCHANGE	DOWN

TCGA-BP-5187-01.	TCGA-CJ-5681-01/	TCGA-B0-4696-01.	TCGA-B0-5710-01.	TCGA-BP-5175-01.
NOCHANGE	DOWN	DOWN	NOCHANGE	UP
UP	NOCHANGE	NOCHANGE	NOCHANGE	UP
NOCHANGE	DOWN	NOCHANGE	NOCHANGE	NOCHANGE
NOCHANGE	NOCHANGE	NOCHANGE	DOWN	DOWN
UP	UP	DOWN	NOCHANGE	UP
NOCHANGE	DOWN	DOWN	NOCHANGE	NOCHANGE
NOCHANGE	NOCHANGE	NOCHANGE	DOWN	DOWN
NOCHANGE	DOWN	NOCHANGE	NOCHANGE	NOCHANGE
UP	NOCHANGE	NOCHANGE	UP	NOCHANGE
DOWN	NOCHANGE	NOCHANGE	DOWN	NOCHANGE
NOCHANGE	NOCHANGE	UP	NOCHANGE	NOCHANGE
UP	UP	DOWN	NOCHANGE	NOCHANGE
NOCHANGE	NOCHANGE	DOWN	NOCHANGE	NOCHANGE
NOCHANGE	NOCHANGE	UP	NOCHANGE	DOWN
NOCHANGE	DOWN	DOWN	DOWN	DOWN
UP	UP	NOCHANGE	NOCHANGE	DOWN
NOCHANGE	NOCHANGE	NOCHANGE	NOCHANGE	DOWN
DOWN	NOCHANGE	UP	DOWN	NOCHANGE
NOCHANGE	NOCHANGE	DOWN	UP	NOCHANGE
NOCHANGE	DOWN	DOWN	NOCHANGE	NOCHANGE
NOCHANGE	NOCHANGE	NOCHANGE	NOCHANGE	NOCHANGE
UP	DOWN	UP	NOCHANGE	NOCHANGE
NOCHANGE	UP	NOCHANGE	NOCHANGE	DOWN
DOWN	NOCHANGE	NOCHANGE	DOWN	DOWN
DOWN	NOCHANGE	DOWN	DOWN	DOWN
NOCHANGE	UP	UP	NOCHANGE	NOCHANGE
UP	NOCHANGE	NOCHANGE	NOCHANGE	NOCHANGE
NOCHANGE	DOWN	NOCHANGE	NOCHANGE	NOCHANGE
NOCHANGE	NOCHANGE	DOWN	NOCHANGE	DOWN
NOCHANGE	DOWN	NOCHANGE	NOCHANGE	DOWN
NOCHANGE	UP	DOWN	DOWN	DOWN

TCGA-DV-5569-01	TCGA-BP-5189-01.	TCGA-AK-3443-01	TCGA-CW-5589-01	TCGA-B0-5109-01.
NOCHANGE	NOCHANGE	DOWN	NOCHANGE	NOCHANGE
UP	UP	NOCHANGE	UP	NOCHANGE
NOCHANGE	NOCHANGE	NOCHANGE	NOCHANGE	NOCHANGE
DOWN	DOWN	DOWN	NOCHANGE	DOWN
NOCHANGE	UP	NOCHANGE	NOCHANGE	NOCHANGE
NOCHANGE	NOCHANGE	DOWN	NOCHANGE	NOCHANGE
DOWN	DOWN	NOCHANGE	NOCHANGE	NOCHANGE
NOCHANGE	NOCHANGE	NOCHANGE	NOCHANGE	NOCHANGE
DOWN	NOCHANGE	DOWN	UP	UP
NOCHANGE	NOCHANGE	UP	NOCHANGE	NOCHANGE
DOWN	DOWN	DOWN	NOCHANGE	NOCHANGE
DOWN	NOCHANGE	NOCHANGE	DOWN	NOCHANGE
NOCHANGE	NOCHANGE	NOCHANGE	NOCHANGE	DOWN
NOCHANGE	NOCHANGE	DOWN	NOCHANGE	NOCHANGE
DOWN	DOWN	DOWN	NOCHANGE	DOWN
NOCHANGE	NOCHANGE	NOCHANGE	NOCHANGE	NOCHANGE
UP	NOCHANGE	NOCHANGE	NOCHANGE	NOCHANGE
NOCHANGE	DOWN	DOWN	NOCHANGE	DOWN
NOCHANGE	NOCHANGE	NOCHANGE	NOCHANGE	NOCHANGE
DOWN	NOCHANGE	DOWN	DOWN	DOWN
NOCHANGE	UP	DOWN	NOCHANGE	NOCHANGE
NOCHANGE	NOCHANGE	DOWN	NOCHANGE	NOCHANGE
NOCHANGE	NOCHANGE	DOWN	NOCHANGE	NOCHANGE
NOCHANGE	DOWN	NOCHANGE	NOCHANGE	DOWN
DOWN	NOCHANGE	NOCHANGE	NOCHANGE	DOWN
NOCHANGE	NOCHANGE	NOCHANGE	NOCHANGE	NOCHANGE
NOCHANGE	NOCHANGE	NOCHANGE	NOCHANGE	UP
NOCHANGE	NOCHANGE	DOWN	NOCHANGE	NOCHANGE
NOCHANGE	NOCHANGE	NOCHANGE	NOCHANGE	NOCHANGE
NOCHANGE	DOWN	DOWN	NOCHANGE	NOCHANGE
DOWN	DOWN	UP	DOWN	DOWN

TCGA-B8-A8YJ-01	TCGA-B0-4810-01.	TCGA-BP-5001-01.	TCGA-EU-5906-01	TCGA-BP-4993-01.
NOCHANGE	NOCHANGE	NOCHANGE	NOCHANGE	NOCHANGE
UP	UP	UP	UP	UP
NOCHANGE	NOCHANGE	NOCHANGE	NOCHANGE	NOCHANGE
DOWN	DOWN	NOCHANGE	DOWN	DOWN
NOCHANGE	NOCHANGE	DOWN	NOCHANGE	NOCHANGE
NOCHANGE	NOCHANGE	NOCHANGE	NOCHANGE	NOCHANGE
NOCHANGE	NOCHANGE	NOCHANGE	DOWN	DOWN
NOCHANGE	NOCHANGE	DOWN	DOWN	NOCHANGE
DOWN	NOCHANGE	UP	NOCHANGE	DOWN
NOCHANGE	NOCHANGE	NOCHANGE	NOCHANGE	NOCHANGE
DOWN	NOCHANGE	UP	NOCHANGE	NOCHANGE
DOWN	NOCHANGE	NOCHANGE	NOCHANGE	NOCHANGE
NOCHANGE	NOCHANGE	NOCHANGE	NOCHANGE	NOCHANGE
NOCHANGE	NOCHANGE	DOWN	NOCHANGE	NOCHANGE
NOCHANGE	NOCHANGE	DOWN	DOWN	NOCHANGE
NOCHANGE	NOCHANGE	NOCHANGE	NOCHANGE	NOCHANGE
UP	NOCHANGE	DOWN	UP	UP
DOWN	NOCHANGE	NOCHANGE	DOWN	DOWN
NOCHANGE	NOCHANGE	NOCHANGE	NOCHANGE	NOCHANGE
DOWN	DOWN	DOWN	DOWN	DOWN
NOCHANGE	NOCHANGE	NOCHANGE	UP	NOCHANGE
UP	NOCHANGE	NOCHANGE	NOCHANGE	NOCHANGE
NOCHANGE	NOCHANGE	DOWN	NOCHANGE	NOCHANGE
DOWN	NOCHANGE	NOCHANGE	DOWN	DOWN
DOWN	DOWN	DOWN	NOCHANGE	DOWN
DOWN	NOCHANGE	DOWN	NOCHANGE	NOCHANGE
NOCHANGE	UP	UP	NOCHANGE	NOCHANGE
UP	NOCHANGE	NOCHANGE	NOCHANGE	NOCHANGE
NOCHANGE	NOCHANGE	DOWN	NOCHANGE	NOCHANGE
NOCHANGE	NOCHANGE	NOCHANGE	NOCHANGE	NOCHANGE
DOWN	DOWN	DOWN	NOCHANGE	DOWN

TCGA-B0-4823-01.	TCGA-DV-5576-01	TCGA-MM-A563-0	TCGA-BP-5192-01.	TCGA-CJ-4868-01/
NOCHANGE	NOCHANGE	NOCHANGE	NOCHANGE	NOCHANGE
UP	NOCHANGE	UP	UP	UP
NOCHANGE	NOCHANGE	NOCHANGE	NOCHANGE	NOCHANGE
DOWN	NOCHANGE	DOWN	DOWN	DOWN
NOCHANGE	DOWN	NOCHANGE	DOWN	NOCHANGE
NOCHANGE	NOCHANGE	NOCHANGE	NOCHANGE	NOCHANGE
DOWN	DOWN	NOCHANGE	DOWN	NOCHANGE
NOCHANGE	NOCHANGE	NOCHANGE	DOWN	NOCHANGE
DOWN	NOCHANGE	NOCHANGE	DOWN	NOCHANGE
NOCHANGE	NOCHANGE	NOCHANGE	NOCHANGE	NOCHANGE
NOCHANGE	DOWN	UP	NOCHANGE	DOWN
NOCHANGE	NOCHANGE	UP	DOWN	NOCHANGE
NOCHANGE	NOCHANGE	NOCHANGE	NOCHANGE	NOCHANGE
NOCHANGE	NOCHANGE	DOWN	NOCHANGE	NOCHANGE
NOCHANGE	NOCHANGE	NOCHANGE	DOWN	NOCHANGE
NOCHANGE	NOCHANGE	NOCHANGE	NOCHANGE	NOCHANGE
NOCHANGE	NOCHANGE	NOCHANGE	NOCHANGE	NOCHANGE
DOWN	DOWN	NOCHANGE	NOCHANGE	DOWN
NOCHANGE	NOCHANGE	NOCHANGE	UP	NOCHANGE
DOWN	DOWN	DOWN	NOCHANGE	DOWN
NOCHANGE	NOCHANGE	UP	NOCHANGE	UP
NOCHANGE	UP	UP	NOCHANGE	UP
NOCHANGE	DOWN	NOCHANGE	NOCHANGE	NOCHANGE
NOCHANGE	NOCHANGE	NOCHANGE	NOCHANGE	DOWN
DOWN	DOWN	DOWN	NOCHANGE	DOWN
NOCHANGE	NOCHANGE	NOCHANGE	NOCHANGE	NOCHANGE
NOCHANGE	NOCHANGE	NOCHANGE	NOCHANGE	NOCHANGE
NOCHANGE	NOCHANGE	NOCHANGE	NOCHANGE	NOCHANGE
NOCHANGE	NOCHANGE	NOCHANGE	NOCHANGE	DOWN
NOCHANGE	DOWN	NOCHANGE	NOCHANGE	NOCHANGE
DOWN	DOWN	DOWN	DOWN	DOWN

TCGA-A3-A8OU-0	TCGA-B0-4718-01.	TCGA-DV-5568-01	TCGA-AK-3456-01	TCGA-B0-4837-01.
NOCHANGE	NOCHANGE	NOCHANGE	NOCHANGE	UP
UP	UP	UP	UP	UP
NOCHANGE	NOCHANGE	NOCHANGE	NOCHANGE	NOCHANGE
DOWN	DOWN	DOWN	DOWN	DOWN
NOCHANGE	NOCHANGE	UP	NOCHANGE	NOCHANGE
NOCHANGE	NOCHANGE	NOCHANGE	UP	UP
NOCHANGE	DOWN	DOWN	DOWN	DOWN
NOCHANGE	NOCHANGE	NOCHANGE	DOWN	NOCHANGE
DOWN	NOCHANGE	NOCHANGE	DOWN	NOCHANGE
NOCHANGE	NOCHANGE	NOCHANGE	NOCHANGE	NOCHANGE
NOCHANGE	UP	NOCHANGE	DOWN	NOCHANGE
NOCHANGE	NOCHANGE	NOCHANGE	NOCHANGE	NOCHANGE
NOCHANGE	NOCHANGE	NOCHANGE	NOCHANGE	NOCHANGE
NOCHANGE	DOWN	DOWN	NOCHANGE	NOCHANGE
DOWN	DOWN	NOCHANGE	NOCHANGE	DOWN
NOCHANGE	NOCHANGE	NOCHANGE	UP	NOCHANGE
NOCHANGE	NOCHANGE	NOCHANGE	NOCHANGE	NOCHANGE
DOWN	DOWN	NOCHANGE	DOWN	NOCHANGE
NOCHANGE	NOCHANGE	DOWN	NOCHANGE	NOCHANGE
NOCHANGE	NOCHANGE	DOWN	NOCHANGE	NOCHANGE
NOCHANGE	NOCHANGE	NOCHANGE	NOCHANGE	NOCHANGE
NOCHANGE	NOCHANGE	UP	NOCHANGE	UP
DOWN	DOWN	DOWN	UP	UP
NOCHANGE	NOCHANGE	NOCHANGE	DOWN	NOCHANGE
DOWN	DOWN	DOWN	DOWN	DOWN
DOWN	NOCHANGE	NOCHANGE	DOWN	NOCHANGE
NOCHANGE	NOCHANGE	NOCHANGE	NOCHANGE	NOCHANGE
NOCHANGE	NOCHANGE	NOCHANGE	NOCHANGE	NOCHANGE
NOCHANGE	NOCHANGE	DOWN	NOCHANGE	DOWN
NOCHANGE	NOCHANGE	NOCHANGE	DOWN	NOCHANGE
DOWN	DOWN	DOWN	NOCHANGE	NOCHANGE

TCGA-B0-4694-01.	TCGA-BP-4334-01.	TCGA-B8-4146-01.	TCGA-CZ-4854-01	TCGA-DV-5575-01
NOCHANGE	DOWN	NOCHANGE	UP	NOCHANGE
UP	NOCHANGE	UP	UP	UP
NOCHANGE	NOCHANGE	NOCHANGE	NOCHANGE	NOCHANGE
DOWN	DOWN	NOCHANGE	DOWN	DOWN
UP	DOWN	UP	NOCHANGE	NOCHANGE
NOCHANGE	DOWN	NOCHANGE	NOCHANGE	NOCHANGE
DOWN	NOCHANGE	DOWN	DOWN	NOCHANGE
NOCHANGE	DOWN	NOCHANGE	NOCHANGE	NOCHANGE
NOCHANGE	NOCHANGE	NOCHANGE	NOCHANGE	NOCHANGE
NOCHANGE	UP	DOWN	DOWN	NOCHANGE
NOCHANGE	NOCHANGE	UP	NOCHANGE	NOCHANGE
NOCHANGE	DOWN	DOWN	NOCHANGE	NOCHANGE
NOCHANGE	NOCHANGE	NOCHANGE	NOCHANGE	NOCHANGE
NOCHANGE	DOWN	UP	NOCHANGE	NOCHANGE
NOCHANGE	DOWN	DOWN	DOWN	NOCHANGE
NOCHANGE	NOCHANGE	UP	NOCHANGE	NOCHANGE
NOCHANGE	DOWN	NOCHANGE	NOCHANGE	UP
NOCHANGE	DOWN	DOWN	NOCHANGE	DOWN
NOCHANGE	DOWN	UP	UP	NOCHANGE
NOCHANGE	DOWN	DOWN	UP	DOWN
UP	NOCHANGE	UP	NOCHANGE	NOCHANGE
UP	DOWN	NOCHANGE	UP	UP
NOCHANGE	DOWN	NOCHANGE	UP	NOCHANGE
NOCHANGE	UP	DOWN	DOWN	NOCHANGE
DOWN	DOWN	NOCHANGE	DOWN	DOWN
NOCHANGE	DOWN	NOCHANGE	DOWN	NOCHANGE
NOCHANGE	UP	NOCHANGE	NOCHANGE	NOCHANGE
NOCHANGE	NOCHANGE	NOCHANGE	NOCHANGE	NOCHANGE
DOWN	DOWN	NOCHANGE	NOCHANGE	NOCHANGE
NOCHANGE	DOWN	NOCHANGE	NOCHANGE	UP
DOWN	NOCHANGE	DOWN	DOWN	DOWN

A-01R-1541-07

Table S7: Clusters and OS-score information

Cluster	Oxidativestress_score P = 7.73e-13	
TCGA-A3-3306-01A-01R-0864-07	C1	-0.053583137
TCGA-A3-3329-01A-01R-0864-07	C2	0.357430504
TCGA-B8-5550-01A-01R-1541-07	C2	0.360308637
TCGA-A3-3378-01A-02R-1325-07	C2	0.105401557
TCGA-A3-3343-01A-01R-0864-07	C3	0.135505277
TCGA-B0-5100-01A-01R-1420-07	C3	-0.381551951
TCGA-CJ-4869-01A-02R-1426-07	C2	0.400899578
TCGA-CJ-4895-01A-01R-1305-07	C2	0.369759761
TCGA-B0-5691-01A-11R-1541-07	C2	0.027536506
TCGA-B0-5121-01A-02R-1420-07	C3	-0.186310164
TCGA-B0-5098-01A-01R-1420-07	C2	-0.25634248
TCGA-A3-3308-01A-02R-1325-07	C2	-0.191070546
TCGA-BP-4807-01A-01R-1305-07	C2	0.4421327
TCGA-B0-5707-01A-11R-1541-07	C2	0.174962375
TCGA-BP-4971-01A-01R-1334-07	C3	-0.47718997
TCGA-B8-4620-01A-02R-1325-07	C2	-0.190079084
TCGA-BP-4967-01A-01R-1334-07	C2	-0.21335267
TCGA-BP-5198-01A-01R-1426-07	C2	0.181643621
TCGA-BP-4326-01A-01R-1289-07	C2	-0.120723971
TCGA-CJ-4904-01A-02R-1426-07	C2	-0.031984153
TCGA-CZ-4853-01A-01R-1426-07	C1	0.176933005
TCGA-A3-3387-01A-01R-1541-07	C2	0.031713714
TCGA-A3-3326-01A-01R-0864-07	C2	-0.020968589
TCGA-BP-4784-01A-01R-1305-07	C3	-0.337361259
TCGA-BP-4766-01A-01R-1289-07	C2	0.060148064
TCGA-CZ-5456-01A-01R-1503-07	C2	0.308035542
TCGA-CZ-4857-01A-01R-1305-07	C2	-0.222342676
TCGA-B0-5400-01A-01R-1503-07	C3	0.350268026
TCGA-BP-5010-01A-02R-1420-07	C3	-0.101350232
TCGA-CJ-4644-01A-02R-1325-07	C1	0.199884596
TCGA-CJ-6033-01A-11R-1672-07	C3	-0.043147025
TCGA-EU-5907-01A-11R-1672-07	C1	-0.02644218
TCGA-B4-5836-01A-11R-1672-07	C2	0.329264829
TCGA-A3-3323-01A-02R-1325-07	C2	-0.204407097
TCGA-BP-4353-01A-02R-1289-07	C2	0.003179241
TCGA-B0-5077-01A-01R-1334-07	C2	-0.140464412
TCGA-CZ-5984-01A-11R-1672-07	C2	0.20878858
TCGA-B8-A54G-01A-11R-A266-07	C2	-0.067868402
TCGA-B0-4713-01A-01R-1277-07	C2	0.270017927
TCGA-CZ-5463-01A-01R-1503-07	C1	0.033667589
TCGA-6D-AA2E-01A-11R-A37O-07	C2	0.297482238
TCGA-CJ-6032-01A-11R-1672-07	C2	0.289623469
TCGA-BP-4801-01A-02R-1420-07	C2	0.414099494
TCGA-EU-5904-01A-11R-1672-07	C2	0.380937906
TCGA-B8-5158-01A-01R-1420-07	C2	0.344677223
TCGA-BP-4969-01A-01R-1334-07	C3	-0.261466871
TCGA-B8-5546-01A-01R-1541-07	C1	0.027413278

TCGA-B4-5844-01A-11R-1672-07	C2	0.032963348
TCGA-CZ-5452-01A-01R-1503-07	C2	0.056737663
TCGA-BP-4991-01A-01R-1334-07	C2	-0.340220247
TCGA-A3-3316-01A-01R-0864-07	C2	0.024114721
TCGA-B8-A54E-01A-11R-A266-07	C1	0.254368034
TCGA-BP-5170-01A-01R-1426-07	C3	0.063207673
TCGA-A3-A6NI-01A-11R-A33J-07	C2	0.126177382
TCGA-B0-5711-01A-11R-1672-07	C1	-0.075323747
TCGA-CZ-5451-01A-01R-1503-07	C1	0.346485243
TCGA-BP-4340-01A-01R-1289-07	C2	0.111797006
TCGA-BP-5180-01A-01R-1426-07	C1	0.247436108
TCGA-CJ-4639-01A-02R-1325-07	C1	-0.296431928
TCGA-B0-4821-01A-01R-1503-07	C3	-0.204767572
TCGA-CZ-4860-01A-01R-1305-07	C2	0.189519371
TCGA-B0-4813-01A-01R-1277-07	C3	-0.412689717
TCGA-B8-4619-01A-02R-1325-07	C1	-0.195128012
TCGA-CJ-4892-01A-01R-1305-07	C1	-0.249979416
TCGA-CJ-4916-01A-01R-1426-07	C3	-0.306193082
TCGA-BP-4964-01A-01R-1334-07	C2	0.058423565
TCGA-B0-5712-01A-11R-1672-07	C1	0.418625874
TCGA-BP-4965-01A-01R-1334-07	C2	0.006767381
TCGA-BP-5199-01A-01R-1426-07	C3	-0.414380222
TCGA-BP-4973-01A-01R-1334-07	C2	-0.236090558
TCGA-B2-5635-01A-01R-A277-07	C2	-0.053585004
TCGA-B0-4844-01A-01R-1277-07	C3	0.071570814
TCGA-G6-A8L7-01A-11R-A37O-07	C3	-0.106705437
TCGA-B0-5088-01A-01R-1334-07	C3	0.05006076
TCGA-A3-3324-01A-02R-1325-07	C2	-0.129870048
TCGA-BP-4968-01A-01R-1334-07	C3	-0.148878559
TCGA-BP-4352-01A-01R-1289-07	C3	-0.137560768
TCGA-CJ-4872-01A-01R-1305-07	C2	0.067686789
TCGA-DV-A4VZ-01A-11R-A266-07	C3	-0.40697873
TCGA-BP-5006-01A-01R-1334-07	C2	0.04016287
TCGA-BP-5009-01A-01R-1334-07	C2	-0.229386049
TCGA-B0-4699-01A-01R-1277-07	C3	-0.258068495
TCGA-B0-4834-01A-01R-1305-07	C1	-0.141718204
TCGA-BP-4998-01A-01R-1334-07	C2	-0.335985939
TCGA-B0-5104-01A-01R-1420-07	C2	0.085757563
TCGA-B0-4945-01A-01R-1420-07	C3	-0.253181797
TCGA-B0-4842-01A-02R-1420-07	C3	0.304409087
TCGA-AK-3447-01A-01R-1766-07	C1	0.146691116
TCGA-B8-5551-01A-01R-1541-07	C2	-0.085560902
TCGA-A3-3349-01A-01R-1188-07	C2	-0.332710599
TCGA-B0-4693-01A-01R-1277-07	C2	0.119147472
TCGA-A3-3346-01A-01R-1766-07	C2	0.215113534
TCGA-B0-5120-01A-01R-1420-07	C1	-0.012499243
TCGA-BP-5177-01A-01R-1426-07	C2	-0.038536794
TCGA-B8-4154-01A-01R-1188-07	C1	0.297893605
TCGA-B8-4153-01B-11R-1672-07	C3	0.129255657

TCGA-CJ-6030-01A-11R-1672-07	C1	0.213652595
TCGA-CW-5584-01A-01R-1541-07	C2	0.150766614
TCGA-B0-5696-01A-11R-1541-07	C2	0.419168312
TCGA-BP-4765-01A-01R-1289-07	C2	0.12882106
TCGA-BP-4782-01A-02R-1420-07	C2	0.352847425
TCGA-B8-5165-01A-01R-1420-07	C2	0.04033482
TCGA-B2-3923-01A-02R-A277-07	C1	-0.110822225
TCGA-AK-3425-01A-02R-1277-07	C2	0.42425801
TCGA-AS-3778-01A-01R-A32Z-07	C3	-0.171561831
TCGA-CW-6087-01A-11R-1672-07	C2	-0.063822133
TCGA-B0-5108-01A-01R-1420-07	C2	0.005020772
TCGA-B0-5699-01A-11R-1541-07	C1	0.154156521
TCGA-B0-4814-01A-01R-1277-07	C1	0.160550064
TCGA-AK-3465-01A-02R-1325-07	C1	-0.186380228
TCGA-CJ-4918-01A-01R-1426-07	C2	-0.064310434
TCGA-BP-4344-01A-01R-1289-07	C2	-0.255232082
TCGA-B4-5835-01A-11R-1672-07	C2	0.456567146
TCGA-A3-3372-01A-02R-1325-07	C2	-0.390968369
TCGA-BP-4981-01A-01R-1334-07	C3	-0.137117221
TCGA-BP-4170-01A-02R-1289-07	C1	0.286618
TCGA-CJ-4643-01A-02R-1325-07	C2	-0.152976371
TCGA-BP-4977-01A-01R-1334-07	C2	0.20126653
TCGA-B2-5633-01A-01R-1541-07	C2	-0.374374275
TCGA-A3-3311-01A-02R-1325-07	C1	0.370421306
TCGA-BP-5173-01A-01R-1426-07	C3	-0.012130397
TCGA-BP-5007-01A-01R-1334-07	C3	-0.360608944
TCGA-A3-3362-01A-02R-1325-07	C2	0.125993864
TCGA-BP-4781-01A-01R-1305-07	C2	0.045410425
TCGA-A3-3365-01A-01R-0864-07	C2	-0.282760171
TCGA-DV-A4W0-01A-11R-A266-07	C2	0.258397544
TCGA-B0-5092-01A-01R-1420-07	C3	-0.388207464
TCGA-CJ-5683-01A-11R-1541-07	C3	0.190700793
TCGA-BP-4331-01A-01R-1289-07	C2	-0.440466859
TCGA-B2-A4SR-01A-11R-A266-07	C2	-0.432662847
TCGA-BP-4976-01A-01R-1334-07	C1	-0.020734368
TCGA-B2-4099-01A-02R-1188-07	C1	0.174351045
TCGA-B2-3923-01B-10R-A277-07	C1	-0.086104742
TCGA-G6-A5PC-01A-11R-A33J-07	C3	0.156510171
TCGA-B0-5812-01A-11R-1672-07	C2	0.100355976
TCGA-BP-4341-01A-01R-1289-07	C3	-0.298706635
TCGA-BP-4795-01A-02R-1420-07	C3	-0.285747102
TCGA-AK-3451-01A-02R-1188-07	C3	0.370580741
TCGA-CZ-5987-01A-11R-1672-07	C3	0.135345494
TCGA-MM-A564-01A-11R-A266-07	C3	-0.291446854
TCGA-BP-4787-01A-01R-1305-07	C2	0.338096326
TCGA-DV-5566-01A-01R-1541-07	C1	0.370849952
TCGA-BP-5190-01A-01R-1426-07	C2	0.365389765
TCGA-BP-4162-01A-02R-1325-07	C2	-0.053833065
TCGA-BP-4343-01A-02R-1289-07	C1	-0.034952361

TCGA-AK-3458-01A-01R-1503-07	C3	-0.099850284
TCGA-CZ-5457-01A-01R-1503-07	C2	0.166460302
TCGA-A3-A8OX-01A-11R-A37O-07	C2	0.290468621
TCGA-B0-4817-01A-01R-1277-07	C2	0.489508841
TCGA-CJ-4888-01A-01R-1305-07	C2	0.061381205
TCGA-BP-5191-01A-01R-1426-07	C3	0.081467645
TCGA-B2-5635-01A-01R-1541-07	C2	-0.072642173
TCGA-T7-A92I-01A-11R-A37O-07	C2	0.246075327
TCGA-CJ-4901-01A-01R-1426-07	C2	-0.30427806
TCGA-G6-A8L8-01A-21R-A37O-07	C3	-0.121838675
TCGA-BP-4173-01A-02R-1289-07	C2	-0.202173391
TCGA-AK-3434-01A-02R-1277-07	C3	-0.12551069
TCGA-BP-4329-01A-02R-1289-07	C1	0.453409564
TCGA-CZ-5462-01A-01R-1503-07	C2	0.508519451
TCGA-B0-4706-01A-01R-1503-07	C2	-0.196259939
TCGA-CJ-4920-01A-01R-1426-07	C2	-0.008793477
TCGA-B8-A54I-01A-21R-A33J-07	C3	0.133957863
TCGA-CJ-4893-01A-01R-1305-07	C2	0.217184992
TCGA-BP-4960-01A-01R-1334-07	C3	-0.1648053
TCGA-B2-3924-01A-02R-1325-07	C1	0.038301133
TCGA-B2-3923-01A-02R-1325-07	C1	-0.058644371
TCGA-BP-5185-01A-01R-1426-07	C3	0.126196274
TCGA-A3-3357-01A-02R-1420-07	C2	0.34873651
TCGA-A3-A8CQ-01A-11R-A37O-07	C2	-0.325623463
TCGA-BP-5176-01A-01R-1426-07	C1	0.391844401
TCGA-BP-4160-01A-02R-1289-07	C2	0.210293905
TCGA-B2-3924-01A-02R-A277-07	C2	-0.226007021
TCGA-B0-4822-01A-01R-1277-07	C3	-0.217107608
TCGA-A3-3328-01A-01R-0864-07	C1	-0.041675613
TCGA-CW-5583-01A-02R-1541-07	C2	-0.108610549
TCGA-B8-4148-01A-02R-1325-07	C2	-0.50172036
TCGA-BP-4959-01A-01R-1334-07	C2	0.224750112
TCGA-B0-5097-01A-01R-1420-07	C2	0.181643121
TCGA-CW-6090-01A-11R-1672-07	C1	0.185847663
TCGA-B2-4102-01A-02R-1325-07	C2	-0.2261456
TCGA-BP-4799-01A-01R-1305-07	C3	0.285918198
TCGA-BP-4177-01A-02R-1420-07	C3	-0.27056082
TCGA-CZ-4858-01A-01R-1305-07	C3	-0.536921305
TCGA-AK-3450-01A-02R-1277-07	C1	0.334782712
TCGA-B0-5102-01A-01R-1420-07	C3	-0.050965864
TCGA-BP-4167-01A-02R-1325-07	C3	0.119526875
TCGA-BP-4989-01A-01R-1334-07	C2	-0.411674463
TCGA-BP-5200-01A-01R-1426-07	C2	-0.076626407
TCGA-B0-4818-01A-01R-1503-07	C2	-0.124837299
TCGA-B0-4714-01A-01R-1277-07	C2	-0.590863978
TCGA-3Z-A93Z-01A-11R-A37O-07	C2	-0.169449068
TCGA-DV-5574-01A-01R-1541-07	C2	0.126959378
TCGA-BP-5194-01A-02R-1426-07	C1	0.13179743
TCGA-BP-4961-01A-01R-1334-07	C2	-0.284215525

TCGA-BP-4963-01A-01R-1334-07	C1	-0.144626432
TCGA-AK-3460-01A-02R-1277-07	C3	-0.164561227
TCGA-B8-5545-01A-01R-1672-07	C2	-0.024701993
TCGA-BP-4165-01A-02R-1289-07	C1	-0.192249819
TCGA-DV-A4VX-01A-11R-A266-07	C2	0.54036279
TCGA-B0-4846-01A-01R-1277-07	C2	-0.007149073
TCGA-CZ-5453-01A-01R-1503-07	C1	0.178305571
TCGA-B0-5095-01A-01R-1420-07	C2	-0.353443542
TCGA-BP-4972-01A-01R-1334-07	C2	0.147722687
TCGA-B0-4712-01A-01R-1503-07	C2	0.178644427
TCGA-BP-4164-01A-02R-1325-07	C1	0.039582128
TCGA-CZ-5454-01A-01R-1503-07	C1	0.453980309
TCGA-BP-5202-01A-02R-1426-07	C1	0.358875149
TCGA-CZ-5470-01A-01R-1503-07	C1	0.33030495
TCGA-CJ-4902-01A-01R-1426-07	C3	-0.368324336
TCGA-CJ-4905-01A-02R-1426-07	C2	-0.215402619
TCGA-B0-5075-01A-01R-1334-07	C2	0.455613602
TCGA-BP-4798-01A-01R-1305-07	C2	-0.331215106
TCGA-CJ-4882-01A-02R-1426-07	C3	-0.352423993
TCGA-B0-5706-01A-11R-1541-07	C2	-0.117871823
TCGA-CJ-4891-01A-01R-1305-07	C2	0.306958141
TCGA-CZ-5988-01A-11R-1672-07	C2	0.183850199
TCGA-AK-3440-01A-02R-1277-07	C1	-0.004465539
TCGA-AK-3453-01A-02R-1277-07	C3	-0.299126475
TCGA-B0-5697-01A-11R-1541-07	C2	0.186425254
TCGA-B0-4841-01A-01R-1277-07	C3	0.108067415
TCGA-CJ-5679-01A-11R-1541-07	C3	0.334415654
TCGA-B0-4845-01A-01R-1277-07	C3	-0.389833215
TCGA-CZ-5465-01A-01R-1503-07	C1	0.220794646
TCGA-CJ-5686-01A-11R-1672-07	C2	0.447744689
TCGA-BP-4345-01A-01R-1289-07	C2	-0.374615952
TCGA-B0-5693-01A-11R-1541-07	C2	0.00188592
TCGA-B0-4838-01A-01R-1305-07	C2	-0.163307879
TCGA-B8-5549-01A-01R-1541-07	C1	0.413704014
TCGA-CJ-4876-01A-01R-1305-07	C3	-0.136380459
TCGA-BP-4337-01A-01R-1289-07	C3	-0.225940264
TCGA-BP-4985-01A-01R-1334-07	C2	-0.025812346
TCGA-A3-3374-01A-02R-1325-07	C1	0.200516193
TCGA-A3-3373-01A-02R-1420-07	C2	0.22620149
TCGA-B0-5700-01A-11R-1541-07	C3	0.336399695
TCGA-B4-5378-01A-01R-1503-07	C3	-0.318449913
TCGA-B0-5709-01A-11R-1541-07	C2	0.222327401
TCGA-BP-4351-01A-01R-1289-07	C3	-0.339437377
TCGA-BP-4774-01A-01R-1289-07	C3	-0.125164721
TCGA-BP-4986-01A-01R-1334-07	C2	-0.432115996
TCGA-B0-5113-01A-01R-1420-07	C2	-0.145181354
TCGA-A3-A6NL-01A-11R-A33J-07	C2	-0.444086961
TCGA-CJ-5680-01A-11R-1541-07	C1	0.233749044
TCGA-B0-5402-01A-01R-1503-07	C1	0.198958358

TCGA-CZ-4862-01A-01R-1305-07	C2	-0.192434033
TCGA-CW-6097-01A-11R-1672-07	C2	-0.053625918
TCGA-B4-5843-01A-11R-1672-07	C2	0.028459621
TCGA-A3-3307-01A-01R-0864-07	C1	-0.128045746
TCGA-B0-4688-01A-01R-1277-07	C3	-0.274235562
TCGA-CJ-5689-01A-11R-1541-07	C2	0.240085544
TCGA-BP-4335-01A-01R-1289-07	C3	-0.288797792
TCGA-CJ-4908-01A-01R-1426-07	C2	-0.386487133
TCGA-CZ-5458-01A-01R-1503-07	C2	0.158500812
TCGA-B0-4690-01A-01R-1277-07	C2	-0.038772439
TCGA-CJ-4900-01A-01R-1334-07	C2	-0.137063621
TCGA-A3-3359-01A-01R-0864-07	C2	-0.216911408
TCGA-CJ-4907-01A-01R-1426-07	C2	-0.181332446
TCGA-CZ-5460-01A-01R-1503-07	C1	0.350223106
TCGA-B8-4622-01A-02R-1277-07	C2	-0.101390902
TCGA-BP-4994-01A-01R-1334-07	C1	0.08986531
TCGA-B0-4703-01A-01R-1277-07	C2	-0.496747178
TCGA-BP-4346-01A-01R-1289-07	C2	-0.402287667
TCGA-B0-4839-01A-01R-1305-07	C3	0.074445084
TCGA-CZ-5985-01A-11R-1672-07	C2	0.379293882
TCGA-CZ-4865-01A-02R-1503-07	C3	0.268575258
TCGA-CJ-5675-01A-11R-1541-07	C1	0.214439522
TCGA-AK-3429-01A-02R-1325-07	C1	0.00068707
TCGA-B0-4843-01A-01R-1277-07	C3	-0.20926357
TCGA-CJ-4886-01A-01R-1305-07	C2	-0.243541714
TCGA-B0-5083-01A-02R-1420-07	C3	0.171707797
TCGA-B0-5117-01A-01R-1420-07	C1	-0.111167615
TCGA-BP-4325-01A-02R-1289-07	C2	0.04248184
TCGA-CW-5580-01A-01R-1672-07	C2	0.044245878
TCGA-BP-5000-01A-01R-1334-07	C3	-0.080713774
TCGA-B0-5106-01A-01R-1420-07	C3	-0.268121457
TCGA-CJ-6031-01A-11R-1672-07	C2	0.535281919
TCGA-B0-4852-01A-01R-1503-07	C2	-0.223832775
TCGA-B0-5690-01A-11R-1541-07	C2	-0.378396842
TCGA-B0-4697-01A-01R-1277-07	C3	-0.492322534
TCGA-CZ-4859-01A-02R-1426-07	C1	0.311139902
TCGA-BP-4789-01A-01R-1305-07	C2	-0.128111475
TCGA-CZ-5989-01A-11R-1672-07	C2	0.099446469
TCGA-A3-3347-01A-02R-1325-07	C2	-0.153427009
TCGA-B0-4827-01A-02R-1420-07	C2	0.34693188
TCGA-BP-4760-01A-02R-1420-07	C3	-0.254182213
TCGA-CW-5581-01A-02R-1541-07	C1	0.334949308
TCGA-AK-3427-01A-01R-0864-07	C1	-0.078087091
TCGA-AK-3445-01A-02R-1277-07	C2	-0.154002429
TCGA-BP-4763-01A-01R-1289-07	C1	0.196271602
TCGA-BP-4776-01A-01R-1289-07	C3	-0.048146801
TCGA-BP-5008-01A-01R-1334-07	C2	-0.036291886
TCGA-B0-5119-01A-02R-1420-07	C1	0.333684582
TCGA-CJ-5684-01A-11R-1541-07	C1	-0.065976828

TCGA-BP-4338-01A-01R-1289-07	C2	0.528251193
TCGA-B0-5085-01A-01R-1334-07	C3	-0.020837411
TCGA-A3-3380-01A-01R-0864-07	C3	-0.455979045
TCGA-B0-4691-01A-01R-1277-07	C3	0.137606499
TCGA-B0-5695-01A-11R-1541-07	C2	0.207336621
TCGA-A3-3317-01A-02R-1325-07	C2	-0.347414042
TCGA-CJ-5672-01A-11R-1541-07	C3	0.224487278
TCGA-CJ-5677-01A-11R-1541-07	C3	0.442901022
TCGA-A3-3367-01A-02R-1420-07	C1	0.246406249
TCGA-B8-A54K-01A-11R-A33J-07	C3	-0.504300751
TCGA-BP-4992-01A-01R-1334-07	C3	-0.402708879
TCGA-B0-5705-01A-11R-1541-07	C2	0.421088068
TCGA-CW-6088-01A-11R-1672-07	C2	0.103102448
TCGA-BP-4758-01A-01R-1289-07	C3	-0.183828201
TCGA-BP-4355-01A-01R-1289-07	C3	-0.338614837
TCGA-CZ-4866-01A-01R-1503-07	C2	0.139548546
TCGA-B8-4621-01A-01R-1503-07	C2	0.363381447
TCGA-BP-4995-01A-01R-1334-07	C1	0.105499551
TCGA-BP-5004-01A-01R-1334-07	C2	0.111045924
TCGA-B8-5162-01A-01R-1420-07	C2	-0.030683198
TCGA-B0-4847-01A-01R-1277-07	C3	-0.276554767
TCGA-BP-4769-01A-01R-1289-07	C3	-0.110942025
TCGA-CZ-5982-01A-11R-1672-07	C2	0.10000047
TCGA-CJ-4870-01A-01R-1305-07	C1	0.320303372
TCGA-AK-3454-01A-02R-1277-07	C3	-0.349738384
TCGA-BP-4975-01A-01R-1334-07	C1	0.487749663
TCGA-CW-5585-01A-01R-1541-07	C1	0.100596565
TCGA-B2-4098-01A-02R-1325-07	C2	0.393984196
TCGA-CJ-5678-01A-11R-1541-07	C1	0.264697206
TCGA-B0-5702-01A-11R-1541-07	C3	-0.014108204
TCGA-CJ-4638-01A-02R-1325-07	C3	0.172319289
TCGA-DV-5573-01A-01R-1541-07	C3	-0.251045539
TCGA-BP-5196-01A-01R-1426-07	C2	-0.087444856
TCGA-A3-3351-01A-02R-1325-07	C2	-0.392537458
TCGA-B8-A54D-01A-21R-A266-07	C2	-0.170092421
TCGA-A3-3322-01A-02R-1325-07	C2	-0.107955909
TCGA-B8-A54H-01A-11R-A33J-07	C1	-0.185737286
TCGA-A3-A8OW-01A-11R-A37O-07	C1	-0.211639161
TCGA-B0-4701-01A-01R-1277-07	C2	0.041331792
TCGA-B2-4101-01A-02R-1277-07	C2	-0.088360952
TCGA-A3-3320-01A-02R-1325-07	C2	0.093302908
TCGA-BP-4999-01A-01R-1334-07	C2	-0.37118013
TCGA-BP-4161-01A-02R-1325-07	C2	0.075540153
TCGA-B8-4143-01A-01R-1188-07	C2	-0.456472748
TCGA-B0-5701-01A-11R-1541-07	C3	-0.00084792
TCGA-BP-4797-01A-01R-1305-07	C2	0.542391883
TCGA-DV-A4W0-05A-11R-A266-07	C3	-0.563231743
TCGA-BP-4174-01A-02R-1289-07	C2	0.424857652
TCGA-A3-3385-01A-02R-1420-07	C2	-0.048148638

TCGA-CZ-5469-01A-01R-1503-07	C3	-0.257380695
TCGA-BP-4970-01A-01R-1334-07	C2	-0.15082798
TCGA-BP-4330-01A-01R-1289-07	C2	-0.374446081
TCGA-BP-4349-01A-01R-1289-07	C2	0.124908753
TCGA-AK-3461-01A-02R-1277-07	C2	-0.19692783
TCGA-A3-3383-01A-02R-1325-07	C3	0.031445644
TCGA-BP-4159-01A-02R-1289-07	C3	0.046440839
TCGA-B2-5635-01B-04R-A277-07	C2	-0.175467561
TCGA-CJ-4889-01A-01R-1305-07	C2	-0.147074834
TCGA-BP-4962-01A-01R-1334-07	C2	0.407692891
TCGA-CJ-5682-01A-11R-1541-07	C2	0.103223158
TCGA-BP-4347-01A-01R-1289-07	C2	-0.35312798
TCGA-BP-5186-01A-01R-1426-07	C2	0.096889604
TCGA-CZ-4856-01A-02R-1426-07	C3	0.428142136
TCGA-B0-4824-01A-01R-1277-07	C3	-0.265523122
TCGA-A3-3376-01A-02R-1420-07	C2	-0.500501753
TCGA-CJ-4635-01A-02R-1305-07	C2	-0.286136786
TCGA-BP-5183-01A-01R-1426-07	C1	-0.41158061
TCGA-A3-A8OV-01A-11R-A37O-07	C1	0.196342855
TCGA-B8-5159-01A-01R-1420-07	C1	0.188631007
TCGA-B8-5163-01A-01R-1420-07	C2	-0.075430561
TCGA-CZ-5461-01A-01R-1503-07	C2	0.058365721
TCGA-GK-A6C7-01A-11R-A33J-07	C2	-0.243500309
TCGA-BP-4771-01A-01R-1289-07	C2	-0.370685944
TCGA-BP-4803-01A-01R-1305-07	C2	0.154686367
TCGA-B2-5639-01A-01R-1541-07	C1	0.391620693
TCGA-CZ-5455-01A-01R-1503-07	C2	-0.060146215
TCGA-CJ-4897-01A-03R-1426-07	C2	0.088105102
TCGA-B0-5107-01A-01R-1420-07	C3	0.011521955
TCGA-BP-4759-01A-01R-1289-07	C3	-0.14224324
TCGA-B0-4836-01A-01R-1305-07	C2	-0.141550014
TCGA-B8-A7U6-01A-12R-A37O-07	C1	0.048930234
TCGA-B2-5636-01A-02R-1541-07	C3	-0.305518602
TCGA-A3-3382-01A-02R-1325-07	C2	0.158114622
TCGA-CZ-5464-01A-01R-1503-07	C1	0.251537395
TCGA-CJ-4634-01A-02R-1325-07	C2	0.217376821
TCGA-CJ-6028-01A-11R-1672-07	C2	0.138735429
TCGA-BP-4804-01A-02R-1305-07	C2	-0.32581532
TCGA-CJ-4912-01A-01R-1426-07	C3	-0.159892416
TCGA-CJ-4873-01A-01R-1305-07	C3	-0.260438346
TCGA-CJ-4894-01A-01R-1305-07	C2	-0.310387913
TCGA-CW-5588-01A-01R-1541-07	C2	0.397711657
TCGA-A3-3325-01A-01R-0864-07	C2	-0.342351441
TCGA-CJ-4871-01A-01R-1305-07	C2	0.231065849
TCGA-CJ-4640-01A-02R-1325-07	C1	0.329047928
TCGA-BP-4762-01A-02R-1289-07	C2	0.174101905
TCGA-B0-5094-01A-01R-1420-07	C2	-0.050956555
TCGA-CZ-5467-01A-01R-1503-07	C2	0.271333374
TCGA-A3-3363-01A-01R-0864-07	C1	-0.262153881

TCGA-CZ-5468-01A-01R-1503-07	C3	-0.008849183
TCGA-B2-3924-01B-03R-A277-07	C2	-0.391729485
TCGA-BP-5181-01A-01R-1426-07	C1	0.262064279
TCGA-B8-5553-01A-01R-1541-07	C2	0.064135375
TCGA-BP-4761-01A-01R-1289-07	C3	0.221363239
TCGA-B2-5633-01A-01R-A277-07	C1	-0.248447588
TCGA-A3-3358-01A-01R-1541-07	C2	0.221038811
TCGA-CZ-4864-01A-01R-1503-07	C1	-0.290867314
TCGA-AK-3436-01A-02R-1325-07	C3	-0.013765975
TCGA-CZ-5466-01A-01R-1503-07	C2	0.197033223
TCGA-B0-5080-01A-01R-1503-07	C2	-0.107302421
TCGA-CZ-4861-01A-01R-1305-07	C2	0.337713123
TCGA-CJ-5671-01A-11R-1541-07	C2	0.205229526
TCGA-AK-3431-01A-02R-1277-07	C3	0.005786279
TCGA-B0-4811-01A-01R-1503-07	C3	-0.37009057
TCGA-A3-3313-01A-02R-1325-07	C1	0.089178535
TCGA-B8-5552-01B-11R-1672-07	C2	0.176050295
TCGA-CJ-6027-01A-11R-1672-07	C3	-0.17622167
TCGA-BP-4756-01A-01R-1289-07	C1	-0.132837421
TCGA-A3-3319-01A-02R-1325-07	C2	0.398985678
TCGA-MW-A4EC-01A-11R-A266-07	C2	0.01946516
TCGA-B0-4816-01A-01R-1503-07	C2	0.113958723
TCGA-CZ-4863-01A-01R-1503-07	C2	0.125276335
TCGA-B0-5110-01A-01R-1420-07	C2	-0.167443804
TCGA-CZ-5986-01A-11R-1672-07	C2	0.124165553
TCGA-B0-4828-01A-01R-1277-07	C1	-0.104107622
TCGA-B8-4151-01A-01R-1188-07	C1	0.293538586
TCGA-EU-5905-01A-11R-1672-07	C3	-0.306973808
TCGA-CJ-4903-01A-01R-1426-07	C2	0.150681239
TCGA-B0-5698-01A-11R-1672-07	C2	0.365255964
TCGA-B0-4815-01A-01R-1503-07	C2	-0.084120128
TCGA-B0-5081-01A-01R-1334-07	C3	-0.310989134
TCGA-A3-3352-01A-01R-0864-07	C1	-0.210934275
TCGA-A3-A6NJ-01A-12R-A33J-07	C2	-0.054208677
TCGA-BP-5184-01A-01R-1426-07	C2	0.308561367
TCGA-CJ-4899-01A-01R-1334-07	C2	-0.284928765
TCGA-CJ-4881-01A-01R-1305-07	C2	0.219606569
TCGA-CJ-4878-01A-01R-1305-07	C2	-0.102169712
TCGA-B0-4819-01A-01R-1277-07	C3	-0.383191732
TCGA-B0-4710-01A-01R-1503-07	C2	-0.346636597
TCGA-A3-3331-01A-02R-1325-07	C2	0.163809575
TCGA-BP-5174-01A-01R-1426-07	C3	0.333911464
TCGA-B0-5694-01A-11R-1541-07	C1	0.212888962
TCGA-B0-5116-01A-02R-1420-07	C1	0.300547868
TCGA-DV-5567-01A-01R-1541-07	C3	-0.375874679
TCGA-BP-5169-01A-01R-1426-07	C3	-0.058312315
TCGA-B0-4700-01A-02R-1541-07	C3	-0.100544359
TCGA-AK-3433-01A-02R-1277-07	C1	-0.08042821
TCGA-CJ-4875-01A-01R-1305-07	C3	-0.344435563

TCGA-A3-3335-01A-01R-0864-07	C1	0.242014232
TCGA-B0-5115-01A-01R-1420-07	C2	-0.242637875
TCGA-BP-5195-01A-02R-1426-07	C2	0.203625362
TCGA-BP-4354-01A-02R-1289-07	C2	-0.248686912
TCGA-BP-4974-01A-01R-1334-07	C3	-0.486998742
TCGA-CJ-4641-01A-02R-1325-07	C2	0.232866652
TCGA-B4-5834-01A-11R-1672-07	C2	-0.020938386
TCGA-BP-4169-01A-02R-1289-07	C2	-0.112038893
TCGA-B2-5633-01B-04R-A277-07	C2	-0.394831993
TCGA-BP-4777-01A-01R-1289-07	C2	0.114821819
TCGA-B0-5399-01A-01R-1503-07	C1	-0.019126681
TCGA-B0-5099-01A-01R-1420-07	C3	0.07862534
TCGA-BP-4166-01A-02R-1289-07	C3	0.08059884
TCGA-CJ-4890-01A-01R-1305-07	C2	0.032808498
TCGA-B0-5084-01A-01R-1334-07	C3	-0.308383224
TCGA-BP-5168-01A-01R-1420-07	C3	0.257181664
TCGA-BP-4770-01A-01R-1503-07	C3	-0.444553388
TCGA-BP-4332-01A-01R-1289-07	C2	-0.212297238
TCGA-B4-5832-01A-11R-1672-07	C2	0.33259166
TCGA-B0-4849-01A-01R-1277-07	C3	-0.4188743
TCGA-B8-5164-01A-01R-1420-07	C2	0.20615725
TCGA-CW-6093-01A-11R-1672-07	C2	-0.183158865
TCGA-MM-A84U-01A-11R-A37O-07	C3	0.083087818
TCGA-CJ-4637-01A-02R-1325-07	C2	-0.425839047
TCGA-CJ-4642-01B-01R-1305-07	C3	-0.357642522
TCGA-BP-5178-01A-01R-1426-07	C2	0.462251975
TCGA-BP-4790-01A-01R-1305-07	C2	-0.099606253
TCGA-B0-5692-01A-11R-1541-07	C1	0.135782161
TCGA-CJ-4885-01A-01R-1305-07	C2	0.342877178
TCGA-BP-4768-01A-01R-1289-07	C2	-0.097740489
TCGA-B0-5713-01A-11R-1672-07	C1	0.132240136
TCGA-CW-5587-01A-01R-1541-07	C2	0.168728951
TCGA-BP-5201-01A-01R-1426-07	C3	-0.213980001
TCGA-CW-5590-01A-01R-1541-07	C2	0.052207827
TCGA-A3-3370-01A-02R-1420-07	C2	-0.361445824
TCGA-B0-4848-01A-01R-1277-07	C2	0.235056204
TCGA-B0-4698-01A-01R-1503-07	C3	-0.394849534
TCGA-BP-4982-01A-01R-1334-07	C2	-0.084477545
TCGA-CJ-4636-01A-02R-1325-07	C2	0.17042887
TCGA-AK-3455-01A-01R-0864-07	C1	-0.035189552
TCGA-A3-A6NN-01A-12R-A33J-07	C2	-0.249836494
TCGA-G6-A8L6-01A-11R-A37O-07	C3	-0.243917635
TCGA-B0-5703-01A-11R-1541-07	C1	0.270609972
TCGA-BP-4176-01A-02R-1289-07	C2	0.188217657
TCGA-B0-4707-01A-01R-1277-07	C2	0.165073173
TCGA-CW-5591-01A-01R-1541-07	C2	0.402228414
TCGA-B4-5838-01A-11R-1672-07	C2	0.366903483
TCGA-BP-4775-01A-01R-1289-07	C2	-0.203858495
TCGA-BP-4158-01A-02R-1289-07	C3	-0.425941105

TCGA-CJ-5676-01A-11R-1541-07	C3	0.30902507
TCGA-DV-5565-01A-01R-1541-07	C2	-0.146438965
TCGA-BP-4163-01A-02R-1325-07	C2	0.293034616
TCGA-BP-4327-01A-01R-1289-07	C3	-0.338386843
TCGA-BP-5182-01A-01R-1426-07	C2	0.346651076
TCGA-CJ-4884-01A-01R-1305-07	C2	0.14978034
TCGA-CZ-5459-01A-01R-1503-07	C2	0.389677633
TCGA-B0-5096-01A-01R-1420-07	C2	-0.013857699
TCGA-B8-A54J-01A-11R-A33J-07	C2	-0.272838453
TCGA-BP-4987-01A-01R-1334-07	C2	-0.301442818
TCGA-B2-5641-01A-01R-1541-07	C2	0.158082123
TCGA-AK-3426-01A-02R-1325-07	C3	-0.36258411
TCGA-B4-5377-01A-01R-1503-07	C1	0.12975563
TCGA-BP-4342-01A-01R-1289-07	C1	-0.056983311
TCGA-BP-4983-01A-01R-1334-07	C2	-0.251538187
TCGA-B0-4833-01A-01R-1305-07	C3	-0.441430615
TCGA-AS-3777-01A-01R-0864-07	C3	0.297724492
TCGA-CJ-4874-01A-01R-1305-07	C1	0.296270866
TCGA-CJ-4887-01A-01R-1305-07	C3	-0.019451492
TCGA-AK-3428-01A-02R-1277-07	C1	0.466742645
TCGA-B8-A54F-01A-11R-A266-07	C2	0.11908097
TCGA-BP-5187-01A-01R-1426-07	C2	0.293563378
TCGA-CJ-5681-01A-11R-1541-07	C1	0.021892691
TCGA-B0-4696-01A-01R-1277-07	C3	-0.319621113
TCGA-B0-5710-01A-11R-1672-07	C2	-0.071643759
TCGA-BP-5175-01A-01R-1426-07	C3	0.170282803
TCGA-DV-5569-01A-01R-1541-07	C2	0.116205441
TCGA-BP-5189-01A-02R-1426-07	C2	0.23767272
TCGA-AK-3443-01A-02R-1325-07	C1	-0.173484092
TCGA-CW-5589-01A-01R-1541-07	C2	0.376295834
TCGA-B0-5109-01A-02R-1420-07	C2	-0.402683451
TCGA-B8-A8YJ-01A-13R-A39I-07	C2	-0.107577925
TCGA-B0-4810-01A-01R-1503-07	C3	-0.330966135
TCGA-BP-5001-01A-01R-1334-07	C3	-0.175920029
TCGA-EU-5906-01A-11R-1672-07	C2	0.255290666
TCGA-BP-4993-01A-02R-1420-07	C2	-0.137620549
TCGA-B0-4823-01A-02R-1420-07	C2	0.355173709
TCGA-DV-5576-01A-01R-1541-07	C3	-0.341083733
TCGA-MM-A563-01A-11R-A266-07	C3	-0.247655006
TCGA-BP-5192-01A-01R-1426-07	C1	0.056593605
TCGA-CJ-4868-01A-01R-1305-07	C2	-0.194187031
TCGA-A3-A8OU-01A-11R-A37O-07	C2	-0.171166195
TCGA-B0-4718-01A-01R-1277-07	C3	-0.304990632
TCGA-DV-5568-01A-01R-1541-07	C3	-0.09206857
TCGA-AK-3456-01A-02R-1325-07	C2	0.327887437
TCGA-B0-4837-01A-01R-1305-07	C2	0.464654609
TCGA-B0-4694-01A-01R-1277-07	C3	0.22207015
TCGA-BP-4334-01A-01R-1289-07	C1	-0.268958819
TCGA-B8-4146-01B-11R-1672-07	C2	0.298872144

TCGA-CZ-4854-01A-01R-1305-07	C2	-0.144865733
TCGA-DV-5575-01A-01R-1541-07	C2	0.160757483

Table S8: Univariate Cox regression for hazard ratio of 32 oxidative stress genes

id	HR	HR.95L	HR.95H	pvalue
CAT	0.981559106	0.974096941	0.989078436	1.75E-06
MT1X	1.003163628	1.001681642	1.004647808	2.82E-05
NFKB1	0.918321975	0.881017452	0.957206066	5.65E-05
TXNRD1	1.022932228	1.010786735	1.035223659	0.000198822
NQO1	1.01606743	1.007393638	1.024815904	0.000268403
NOX1	3.83524286	1.713516183	8.584154581	0.001075206
GCLC	0.863130022	0.788274594	0.945093805	0.001472692
SOD2	1.002887455	1.001041608	1.004736707	0.002158121
MAPK10	0.717608306	0.569266558	0.904605537	0.004977359
NFE2L2	0.971996264	0.951134787	0.993315301	0.010291858
HMOX1	0.997403844	0.995268355	0.999543914	0.017448224
MGST1	1.0115201	1.00143695	1.021704775	0.0250336
GSTT2	2.849093772	0.940629648	8.62968262	0.064066615
NFIX	0.97532318	0.948615798	1.002782483	0.077762613
GPX1	1.000837644	0.999860881	1.001815361	0.092822657
FOS	0.999030576	0.99786839	1.000194116	0.102440157
SP1	0.978090025	0.952109796	1.004779177	0.10677648
NOX4	0.973577043	0.939818315	1.008548399	0.136956943
GSR	0.99110046	0.977648541	1.00473747	0.199804636
CYBB	1.005187656	0.996788954	1.013657123	0.22678778
CYP1A1	0.702030494	0.378128015	1.303386143	0.262438604
GPX3	1.000117013	0.999907943	1.000326128	0.272681824
TXN2	0.99297428	0.980078983	1.006039246	0.290439046
SOD1	1.002007614	0.997287432	1.006750138	0.40513128
MAOA	0.99625026	0.986796914	1.005794167	0.439944166
SOD3	0.996223758	0.985484502	1.007080045	0.493871377
MAPK14	0.984190705	0.931192086	1.040205729	0.572588445
NOX3	0.533037206	0.037134818	7.651273916	0.643447746
TXNRD2	0.976768826	0.863571394	1.104804243	0.708388493
UGT1A6	1.001802717	0.990127272	1.013615838	0.763317417
XDH	1.012097146	0.87186029	1.174890798	0.874446746
JUNB	0.999896414	0.998261934	1.001533569	0.901231196
NOX5	0.979143371	0.688590061	1.392296802	0.906583595

Table S9: Lasso regression coefficients of 9 selected genes

Gene	Coef
CAT	-0.009160543
MT1X	0.001344262
NFKB1	-0.035375003
TXNRD1	0.013734239
NOX1	0.298518736
GCLC	-0.020526275
SOD2	0.002072399
MAPK10	-0.015777878
HMOX1	-0.0024321

Table S10: Information of GSEA in GO database

ONTOLOGY	ID	Description	setSize	enrichmentScor	NES
MF	GO:0002162	dystroglycan bi	10	0.858130588	1.615937779
CC	GO:0042571	immunoglobuli	61	0.743914921	1.708087774
CC	GO:0019814	immunoglobuli	142	0.736489005	1.757291654
MF	GO:0034987	immunoglobuli	65	0.734812909	1.690761695
BP	GO:0006910	phagocytosis, re	73	0.704009799	1.634868452
MF	GO:0003823	antigen binding	147	0.662754731	1.582016398
MF	GO:0005549	odorant binding	98	0.651442675	1.534151528
BP	GO:0002433	immune respons	138	0.639279021	1.524228984
BP	GO:0038096	Fc-gamma recep	138	0.639279021	1.524228984
BP	GO:0006958	complement act	126	0.638742155	1.517034609
BP	GO:0002431	Fc receptor med	144	0.631947744	1.509259321
BP	GO:0006911	phagocytosis, en	107	0.631590969	1.492661746
BP	GO:0038094	Fc-gamma recep	141	0.626940146	1.494359358
BP	GO:0099024	plasma membra	116	0.618680454	1.468597888
BP	GO:0050871	positive regulati	131	0.613531235	1.458670383
BP	GO:0002455	humoral immun	139	0.612676789	1.460587444
BP	GO:0050853	B cell receptor s	114	0.609233633	1.444653058
BP	GO:0038095	Fc-epsilon recep	167	0.608145404	1.457762731
BP	GO:0010324	membrane invag	124	0.606929229	1.443132791
MF	GO:0004984	olfactory recept	419	0.598295022	1.466417825
BP	GO:0050911	detection of che	419	0.598295022	1.466417825
BP	GO:0070268	cornification	112	0.594874111	1.409490279
BP	GO:0002377	immunoglobuli	192	0.584811758	1.409797444
BP	GO:0007608	sensory percepti	446	0.583518962	1.429814984
BP	GO:0050907	detection of che	468	0.573793857	1.407061157
BP	GO:0050864	regulation of B	173	0.562846881	1.350619302
BP	GO:0031424	keratinization	223	0.559649061	1.355501547
BP	GO:0016064	immunoglobuli	207	0.5568685	1.34587794
BP	GO:0019724	B cell mediated	210	0.552236706	1.334532352
BP	GO:0008037	cell recognition	204	0.549081473	1.325358789
BP	GO:0030216	keratinocyte dif	301	0.5287161	1.289231888
BP	GO:0038093	Fc receptor sign	239	0.528564369	1.283159053
BP	GO:0009913	epidermal cell d	354	0.506221159	1.237393265
BP	GO:0030073	insulin secretion	206	-0.299858046	-1.0705521
BP	GO:0009743	response to carb	227	-0.305559733	-1.181291825
BP	GO:0031669	cellular respons	235	-0.307879207	-1.234978142
BP	GO:0045017	glycerolipid bio	249	-0.310337081	-1.306060975
BP	GO:0051650	establishment of	183	-0.313979705	-1.237440343
BP	GO:0009746	response to hex	199	-0.314890811	-1.091219328
BP	GO:0034284	response to mon	204	-0.315432433	-1.114910548
BP	GO:0050796	regulation of ins	175	-0.324345447	-1.091930293
BP	GO:0009749	response to gluc	194	-0.325634096	-1.116383759
BP	GO:0006814	sodium ion tran	218	-0.326230709	-1.221406577
CC	GO:0031968	organelle outer	198	-0.32755002	-1.134017127

BP	GO:0009408	response to heat	172	-0.327629487	-1.10074027
CC	GO:0019867	outer membrane	200	-0.327948133	-1.138205876
BP	GO:0010565	regulation of ce	179	-0.328864243	-1.137680577
CC	GO:0005681	spliceosomal co	185	-0.329597574	-1.121139098
BP	GO:0006839	mitochondrial tr	248	-0.331416738	-1.393172602
BP	GO:0045732	positive regulati	213	-0.332926325	-1.231410708
MF	GO:1901681	sulfur compoun	242	-0.333354995	-1.358716809
MF	GO:0043177	organic acid bin	201	-0.335193286	-1.165809815
BP	GO:0140053	mitochondrial g	159	-0.3355921	-1.215441782
MF	GO:0016747	transferase activ	226	-0.338713718	-1.307039023
BP	GO:0009132	nucleoside diph	153	-0.338817391	-1.235720889
MF	GO:0035091	phosphatidylino	241	-0.338896754	-1.379415863
BP	GO:0042133	neurotransmitte	151	-0.340640494	-1.224660505
BP	GO:0097237	cellular respons	237	-0.341434049	-1.384545363
BP	GO:0002262	myeloid cell ho	147	-0.342493557	-1.116921993
BP	GO:0042445	hormone metab	232	-0.34315673	-1.351773837
CC	GO:0043296	apical junction c	143	-0.343337199	-1.082734835
MF	GO:0008201	heparin binding	162	-0.346114421	-1.258891663
BP	GO:0008033	tRNA processing	126	-0.346305088	-1.252527926
BP	GO:0006575	cellular modifie	186	-0.347899749	-1.331139684
MF	GO:0016829	lyase activity	186	-0.349706613	-1.338053136
BP	GO:0042157	lipoprotein meta	130	-0.353214024	-1.250524154
CC	GO:0005923	bicellular tight j	123	-0.354283667	-1.302933396
BP	GO:0019751	polyol metaboli	124	-0.354734689	-1.234513815
CC	GO:0070160	tight junction	128	-0.356097754	-1.248724898
BP	GO:0055067	monovalent ino	154	-0.358409059	-1.278262145
BP	GO:0016482	cytosolic transp	157	-0.359531324	-1.298261158
BP	GO:0042180	cellular ketone r	245	-0.360240521	-1.498794142
BP	GO:0006399	tRNA metabolic	165	-0.361088111	-1.334771109
BP	GO:0006661	phosphatidylino	116	-0.3612793	-1.188199685
BP	GO:0007584	response to nutr	218	-0.361279495	-1.3526291
BP	GO:0046031	ADP metabolic	121	-0.362418782	-1.245707184
BP	GO:0000041	transition metal	123	-0.363132227	-1.335475354
BP	GO:0006888	endoplasmic ret	212	-0.36326736	-1.342306736
MF	GO:0005506	iron ion binding	152	-0.363584887	-1.308195078
BP	GO:0009185	ribonucleoside c	135	-0.36455993	-1.319368615
BP	GO:0046890	regulation of lip	195	-0.36508066	-1.253579672
BP	GO:0098754	detoxification	134	-0.366832621	-1.321771255
MF	GO:0015081	sodium ion tran	150	-0.367544556	-1.320263165
BP	GO:0009135	purine nucleosic	133	-0.367748874	-1.329072474
BP	GO:0009179	purine ribonucl	133	-0.367748874	-1.329072474
BP	GO:0034754	cellular hormon	129	-0.37069332	-1.305860001
CC	GO:0005741	mitochondrial o	175	-0.371177427	-1.249593235
MF	GO:0019842	vitamin binding	136	-0.371756703	-1.172564422
BP	GO:0007586	digestion	138	-0.372717605	-1.158993762
MF	GO:0016651	oxidoreductase	100	-0.376179037	-1.35379787

BP	GO:1903034	regulation of res	171	-0.377107673	-1.26040315
BP	GO:0032091	negative regulat	101	-0.380777544	-1.376804187
MF	GO:0016874	ligase activity	124	-0.380939226	-1.325708343
BP	GO:0016241	regulation of ma	170	-0.382034371	-1.275820331
CC	GO:0030134	COPII-coated E	91	-0.382145576	-1.461381032
BP	GO:0015718	monocarboxylic	162	-0.38461928	-1.398942012
BP	GO:0042594	response to starv	188	-0.386255806	-1.490569074
BP	GO:0003014	renal system pro	120	-0.389837031	-1.263305253
BP	GO:0034101	erythrocyte hom	122	-0.392086455	-1.351645657
BP	GO:0048259	regulation of rec	99	-0.394037647	-1.511980484
BP	GO:0050810	regulation of ste	95	-0.394081168	-1.426370414
BP	GO:0033559	unsaturated fatty	109	-0.395354506	-1.250702588
MF	GO:0051087	chaperone bindi	101	-0.396030805	-1.431956477
BP	GO:0030218	erythrocyte diff	114	-0.398435532	-1.306629405
BP	GO:0045834	positive regulati	143	-0.400952943	-1.264429604
MF	GO:0140101	catalytic activity	102	-0.401072607	-1.407554981
BP	GO:1901655	cellular response	93	-0.40114107	-1.445716323
BP	GO:0019217	regulation of fat	96	-0.402145314	-1.495447987
MF	GO:0008135	translation facto	85	-0.402765435	-1.49873383
MF	GO:0016705	oxidoreductase a	159	-0.403273039	-1.46056746
BP	GO:0016052	carbohydrate ca	197	-0.404304061	-1.396530156
MF	GO:0031072	heat shock prote	119	-0.406584405	-1.316078938
BP	GO:0003073	regulation of sys	91	-0.406679869	-1.555203787
BP	GO:0015748	organophosphat	119	-0.407320583	-1.318461884
MF	GO:0016209	antioxidant activ	84	-0.407531973	-1.506727581
BP	GO:0097581	lamellipodium c	80	-0.407550031	-1.456702578
MF	GO:0043178	alcohol binding	85	-0.408407189	-1.519727408
BP	GO:0032368	regulation of lip	118	-0.40990222	-1.324523519
BP	GO:0006720	isoprenoid meta	138	-0.410549175	-1.276633908
BP	GO:0045333	cellular respirati	183	-0.410639661	-1.61839149
BP	GO:0098876	vesicle-mediated	96	-0.41094226	-1.528160975
BP	GO:0001523	retinoid metabo	104	-0.411441428	-1.430862232
BP	GO:1990542	mitochondrial tr	90	-0.411937743	-1.560738239
CC	GO:0098862	cluster of actin-l	150	-0.412107002	-1.480336698
MF	GO:0016410	N-acyltransferas	107	-0.414285951	-1.44167655
BP	GO:0006826	iron ion transpo	76	-0.416757229	-1.452495318
BP	GO:0097327	response to antin	96	-0.417152371	-1.551254367
CC	GO:0072562	blood micropart	144	-0.419503219	-1.346878244
BP	GO:1903035	negative regulat	89	-0.419769946	-1.582977382
BP	GO:0061041	regulation of wo	141	-0.422765635	-1.32592752
BP	GO:0006721	terpenoid metab	120	-0.423529963	-1.372490514
BP	GO:0098869	cellular oxidant	99	-0.423730949	-1.625918055
BP	GO:0016101	diterpenoid meta	110	-0.423963652	-1.353266531
MF	GO:0030165	PDZ domain bin	86	-0.425295475	-1.595851196
CC	GO:0016234	inclusion body	82	-0.427201197	-1.489832664
MF	GO:0015399	primary active tr	111	-0.428260709	-1.370896215

MF	GO:0042626	ATPase-coupled	102	-0.430277488	-1.510048829
BP	GO:1901568	fatty acid deriva	167	-0.430353752	-1.423699122
BP	GO:0055088	lipid homeostasi	146	-0.43062089	-1.398516322
BP	GO:0006090	pyruvate metabo	149	-0.430635629	-1.51937183
BP	GO:0022600	digestive system	99	-0.430973424	-1.653708501
BP	GO:1903008	organelle disass	100	-0.43150836	-1.552917736
CC	GO:0031091	platelet alpha gr	91	-0.433814136	-1.658969222
BP	GO:1900034	regulation of ce	78	-0.435053547	-1.541106813
MF	GO:0001098	basal transcriptio	71	-0.435702756	-1.528064736
MF	GO:0001099	basal RNA poly	71	-0.435702756	-1.528064736
BP	GO:0061008	hepaticobiliary s	138	-0.437148878	-1.35934771
BP	GO:0051893	regulation of fo	60	-0.437341427	-1.475176713
BP	GO:0090109	regulation of ce	60	-0.437341427	-1.475176713
BP	GO:0150116	regulation of ce	60	-0.437341427	-1.475176713
MF	GO:0004402	histone acetyltra	64	-0.438724452	-1.518898477
BP	GO:0001889	liver developme	135	-0.439407842	-1.590248594
BP	GO:0046165	alcohol biosynth	175	-0.439650093	-1.480110974
BP	GO:0009267	cellular respons	147	-0.441214677	-1.438866121
BP	GO:0006690	icosanoid metab	114	-0.441226752	-1.446958923
BP	GO:0046889	positive regulati	81	-0.44214066	-1.561839785
BP	GO:0032922	circadian regula	62	-0.44275827	-1.536142856
BP	GO:0006694	steroid biosynth	196	-0.44492316	-1.529839288
BP	GO:1901607	alpha-amino aci	64	-0.445236521	-1.541443768
BP	GO:0060964	regulation of ge	83	-0.445774846	-1.607634939
BP	GO:0006633	fatty acid biosyn	162	-0.446131033	-1.622673323
BP	GO:0046364	monosaccharide	91	-0.446315359	-1.706775745
MF	GO:0015297	antiporter activi	85	-0.446699322	-1.662216584
BP	GO:0070830	bicellular tight j	55	-0.447570653	-1.471555085
BP	GO:0044242	cellular lipid cat	216	-0.449132316	-1.643711805
BP	GO:0051188	cofactor biosynt	230	-0.449543609	-1.763609782
BP	GO:1901616	organic hydrox	74	-0.449848702	-1.596522331
MF	GO:0008514	organic anion tr	209	-0.450055698	-1.621893028
MF	GO:0015291	secondary active	237	-0.451140831	-1.829416098
MF	GO:0022853	active ion trans	236	-0.45174857	-1.829368772
BP	GO:0072330	monocarboxylic	235	-0.454118604	-1.821579819
MF	GO:0043175	RNA polymeras	60	-0.454888414	-1.534363666
BP	GO:1990748	cellular detoxifi	109	-0.455283743	-1.440288517
BP	GO:1905953	negative regulat	55	-0.455297705	-1.496960644
BP	GO:0000422	autophagy of m	76	-0.456098333	-1.589608166
BP	GO:0061726	mitochondrion	76	-0.456098333	-1.589608166
CC	GO:0008023	transcription elo	58	-0.456906965	-1.552434587
MF	GO:0005342	organic acid tra	152	-0.45786228	-1.647409453
MF	GO:0046943	carboxylic acid	152	-0.45786228	-1.647409453
MF	GO:0015294	solute:cation sy	103	-0.458164551	-1.564202524
BP	GO:0008652	cellular amino a	81	-0.458398887	-1.61927116
MF	GO:0004497	monooxygenase	99	-0.458975518	-1.76115666

BP	GO:0019218	regulation of ste	124	-0.460100969	-1.601199491
BP	GO:0042304	regulation of fat	55	-0.460994354	-1.515690495
BP	GO:0019319	hexose biosynth	85	-0.461689744	-1.717997568
BP	GO:0002576	platelet degranu	128	-0.462128475	-1.620541906
BP	GO:0019674	NAD metabolic	51	-0.462223989	-1.465834224
BP	GO:0090181	regulation of ch	63	-0.462645306	-1.591304876
BP	GO:0006094	gluconeogenesis	82	-0.464514093	-1.619958639
MF	GO:0015293	symporter activi	144	-0.465950407	-1.496003934
BP	GO:0046854	phosphatidylino	50	-0.467107757	-1.506582215
MF	GO:0016597	amino acid bind	56	-0.468195278	-1.597376508
BP	GO:0061045	negative regulat	75	-0.47129938	-1.676570136
BP	GO:0030004	cellular monova	108	-0.471443098	-1.486421548
BP	GO:0009060	aerobic respirati	83	-0.471556578	-1.700613745
BP	GO:0001676	long-chain fatty	108	-0.472059606	-1.488365347
CC	GO:0016323	basolateral plasn	217	-0.475160378	-1.746468116
BP	GO:0007040	lysosome organ	61	-0.475965968	-1.63797178
BP	GO:0080171	lytic vacuole org	61	-0.475965968	-1.63797178
BP	GO:0009108	coenzyme biosy	146	-0.479042043	-1.555772449
BP	GO:0006639	acylglycerol me	126	-0.479185248	-1.733133373
BP	GO:0006513	protein monoub	66	-0.479538923	-1.651337256
BP	GO:0006638	neutral lipid me	127	-0.479724713	-1.739941637
BP	GO:0071466	cellular response	180	-0.480088301	-1.871971984
MF	GO:0005319	lipid transporter	130	-0.480704664	-1.701893902
MF	GO:0016616	oxidoreductase a	119	-0.481443202	-1.558390461
BP	GO:0050994	regulation of lip	54	-0.487177822	-1.596846353
MF	GO:0015370	solute:sodium sy	73	-0.488347807	-1.698863549
BP	GO:0017001	antibiotic catabo	57	-0.489098461	-1.669585282
CC	GO:0010494	cytoplasmic stre	66	-0.48964569	-1.686140856
BP	GO:0042398	cellular modified	50	-0.490784963	-1.582949299
BP	GO:0006081	cellular aldehyd	61	-0.491076354	-1.689972108
MF	GO:0019829	ATPase-coupled	53	-0.491435466	-1.563302978
CC	GO:0031093	platelet alpha gr	67	-0.491737088	-1.709618016
BP	GO:0097006	regulation of pla	95	-0.494409952	-1.789508824
BP	GO:0033013	tetrapyrrole met	58	-0.494547212	-1.680325002
CC	GO:0032592	integral compon	70	-0.494619773	-1.73731617
MF	GO:0042625	ATPase-coupled	57	-0.495604931	-1.691795752
CC	GO:0012507	ER to Golgi tran	60	-0.495699377	-1.672021291
MF	GO:0046906	tetrapyrrole bind	140	-0.496074618	-1.554119935
BP	GO:0045851	pH reduction	53	-0.496600465	-1.579733331
BP	GO:0030641	regulation of ce	90	-0.497264021	-1.884020062
BP	GO:0030258	lipid modificatio	238	-0.497410786	-2.022388412
BP	GO:0006733	oxidoreduction	51	-0.497420336	-1.577451128
BP	GO:0016125	sterol metabolic	166	-0.497776305	-1.645489732
MF	GO:0050661	NADP binding	52	-0.500139674	-1.620216346
BP	GO:0006805	xenobiotic meta	125	-0.50041733	-1.821331521
CC	GO:0098573	intrinsic compon	71	-0.501077832	-1.757343405

MF	GO:0001221	transcription co	43	-0.501539957	-1.561310978
BP	GO:0034308	primary alcohol	85	-0.501908135	-1.867654558
BP	GO:0045540	regulation of ch	50	-0.502531209	-1.620834959
BP	GO:0106118	regulation of ste	50	-0.502531209	-1.620834959
BP	GO:1900046	regulation of he	79	-0.503188891	-1.764031089
MF	GO:0016614	oxidoreductase a	128	-0.50354547	-1.765778522
MF	GO:0005496	steroid binding	95	-0.503760032	-1.82335129
BP	GO:0033865	nucleoside bispl	139	-0.507213284	-1.588138788
BP	GO:0033875	ribonucleoside b	139	-0.507213284	-1.588138788
BP	GO:0034032	purine nucleosic	139	-0.507213284	-1.588138788
MF	GO:0016903	oxidoreductase a	43	-0.508528729	-1.583067263
BP	GO:0043648	dicarboxylic aci	97	-0.509036851	-1.897512893
BP	GO:0006892	post-Golgi vesic	104	-0.509825185	-1.773009604
MF	GO:0020037	heme binding	132	-0.509937949	-1.833792806
BP	GO:0006893	Golgi to plasma	63	-0.51005986	-1.754390958
BP	GO:0050818	regulation of co	81	-0.51041156	-1.803003328
BP	GO:1903036	positive regulati	70	-0.511987318	-1.798318416
BP	GO:0042737	drug catabolic p	139	-0.51258325	-1.604952722
BP	GO:0051187	cofactor catabol	64	-0.513965083	-1.779387445
BP	GO:0006885	regulation of pH	98	-0.514553997	-1.933168293
BP	GO:0009066	aspartate family	49	-0.515598863	-1.686295519
BP	GO:0042572	retinol metaboli	41	-0.516198448	-1.636019028
BP	GO:0006641	triglyceride meta	102	-0.516271561	-1.811843028
BP	GO:0044275	cellular carbohy	45	-0.516771399	-1.65759855
BP	GO:0030193	regulation of blo	78	-0.517377286	-1.832725339
BP	GO:1900047	negative regulat	54	-0.517538659	-1.696361538
BP	GO:0051453	regulation of int	84	-0.517758562	-1.914257426
CC	GO:0016469	proton-transpor	48	-0.518399941	-1.660237562
BP	GO:0015918	sterol transport	107	-0.520544107	-1.811445041
BP	GO:0016999	antibiotic metab	120	-0.520643504	-1.687196496
BP	GO:0006635	fatty acid beta-o	72	-0.522286438	-1.829971303
BP	GO:0019076	viral release fro	35	-0.523540874	-1.658579985
BP	GO:0035890	exit from host	35	-0.523540874	-1.658579985
BP	GO:0035891	exit from host c	35	-0.523540874	-1.658579985
BP	GO:0046503	glycerolipid cata	64	-0.524269726	-1.815062927
MF	GO:1901682	sulfur compoun	35	-0.525287124	-1.664112112
BP	GO:0030301	cholesterol trans	94	-0.525545765	-1.884932155
MF	GO:0032934	sterol binding	56	-0.525574006	-1.793139765
BP	GO:0034381	plasma lipoprote	65	-0.525738455	-1.795364076
MF	GO:0030170	pyridoxal phosp	54	-0.527111141	-1.727737728
MF	GO:0070279	vitamin B6 bind	54	-0.527111141	-1.727737728
BP	GO:0034440	lipid oxidation	101	-0.52899397	-1.912720758
BP	GO:0050819	negative regulat	55	-0.530323962	-1.743637385
BP	GO:1990928	response to ami	45	-0.532610529	-1.708404225
MF	GO:0017112	Rab guanyl-nuc	37	-0.533620134	-1.669114112
BP	GO:0019395	fatty acid oxidat	99	-0.533773478	-2.048167447

BP	GO:0030195	negative regulat	53	-0.53631534	-1.706070129
BP	GO:0035329	hippo signaling	38	-0.536718744	-1.674282529
BP	GO:1901605	alpha-amino aci	199	-0.536848261	-1.860388356
BP	GO:0031100	animal organ re;	72	-0.536916562	-1.881231887
BP	GO:0035384	thioester biosyn	54	-0.537040064	-1.76028224
BP	GO:0071616	acyl-CoA biosyı	54	-0.537040064	-1.76028224
BP	GO:0033866	nucleoside bispl	68	-0.537600974	-1.921674258
BP	GO:0034030	ribonucleoside l	68	-0.537600974	-1.921674258
BP	GO:0034033	purine nucleosic	68	-0.537600974	-1.921674258
BP	GO:0032365	intracellular lipi	43	-0.538509325	-1.67639788
CC	GO:0016592	mediator compl	37	-0.539484894	-1.687458534
BP	GO:0042558	pteridine-contai	34	-0.539993622	-1.726050157
BP	GO:0014741	negative regulat	34	-0.540166381	-1.726602367
BP	GO:0051452	intracellular pH	51	-0.542365069	-1.719982736
MF	GO:0016877	ligase activity, f	40	-0.542502772	-1.734616014
MF	GO:0016830	carbon-carbon l	51	-0.542549567	-1.720567826
BP	GO:0046164	alcohol cataboli	54	-0.543316228	-1.780853928
BP	GO:0086005	ventricular card	35	-0.543958016	-1.723261587
BP	GO:0008203	cholesterol meta	150	-0.544151381	-1.954655597
BP	GO:0019433	triglyceride cata	35	-0.54907871	-1.739483971
CC	GO:0099023	vesicle tethering	64	-0.54940144	-1.902070892
BP	GO:1902652	secondary alcoh	161	-0.55025087	-1.99790692
BP	GO:0032374	regulation of ch	57	-0.550670872	-1.879768709
BP	GO:1903514	release of seque	34	-0.551164165	-1.761755983
MF	GO:0015485	cholesterol bind	49	-0.551481829	-1.80365281
BP	GO:0032371	regulation of ste	58	-0.554269226	-1.883242721
BP	GO:0046460	neutral lipid bio	41	-0.554389619	-1.757060619
BP	GO:0046463	acylglycerol bio	41	-0.554389619	-1.757060619
MF	GO:0050660	flavin adenine d	79	-0.55564815	-1.947937704
BP	GO:0016126	sterol biosynthe	81	-0.558017834	-1.971170114
BP	GO:0046621	negative regulat	39	-0.558294722	-1.774813162
BP	GO:0009064	glutamine famil	72	-0.559438952	-1.96014515
BP	GO:0090303	positive regulati	58	-0.560298451	-1.903728242
CC	GO:0005903	brush border	99	-0.561350748	-2.153985494
BP	GO:0019432	triglyceride bios	39	-0.561972036	-1.786503304
BP	GO:0010614	negative regulat	32	-0.563511754	-1.819992574
MF	GO:0015301	anion:anion anti	31	-0.564283242	-1.800243324
MF	GO:0140323	solute:anion ant	31	-0.564283242	-1.800243324
BP	GO:0055092	sterol homeosta	93	-0.565131185	-2.036738295
BP	GO:0042632	cholesterol hom	92	-0.566355947	-2.131556989
BP	GO:0009069	serine family an	40	-0.569092598	-1.819635189
MF	GO:0032451	demethylase act	37	-0.574620483	-1.797359389
MF	GO:1901618	organic hydrox	44	-0.574905602	-1.810784725
MF	GO:0120013	lipid transfer act	40	-0.576847138	-1.844429806
BP	GO:1902653	secondary alcoh	76	-0.577384968	-2.012320134
BP	GO:0072329	monocarboxylic	131	-0.5775906	-2.075973102

BP	GO:0061912	selective autoph	48	-0.577632511	-1.849936921
MF	GO:0019825	oxygen binding	35	-0.578240811	-1.831869647
MF	GO:0009678	pyrophosphate l	33	-0.578258448	-1.861396724
BP	GO:0006536	glutamate metab	34	-0.578261104	-1.848369366
BP	GO:0060612	adipose tissue de	42	-0.578461191	-1.852463411
MF	GO:0016627	oxidoreductase a	58	-0.578578271	-1.965837658
MF	GO:0015144	carbohydrate tra	37	-0.578925026	-1.810823597
BP	GO:0006695	cholesterol biosy	75	-0.579135132	-2.060178115
CC	GO:0098576	lumenal side of	32	-0.579457351	-1.871492597
BP	GO:0055022	negative regulat	31	-0.580209037	-1.851051685
BP	GO:0061117	negative regulat	31	-0.580209037	-1.851051685
BP	GO:0071827	plasma lipoprote	44	-0.581056544	-1.830158398
BP	GO:0090382	phagosome matu	44	-0.581247655	-1.830760342
CC	GO:0005778	peroxisomal me	59	-0.582654717	-1.996844322
CC	GO:0031903	microbody mem	59	-0.582654717	-1.996844322
BP	GO:0019835	cytolysis	40	-0.585055217	-1.870674584
MF	GO:0016709	oxidoreductase a	39	-0.58521908	-1.860405419
MF	GO:0016878	acid-thiol ligase	30	-0.585828196	-1.870252309
BP	GO:0046461	neutral lipid cata	43	-0.586864058	-1.826927812
BP	GO:0046464	acylglycerol cata	43	-0.586864058	-1.826927812
BP	GO:0071825	protein-lipid con	48	-0.587041999	-1.880071929
BP	GO:0010880	regulation of rel	29	-0.588761789	-1.929589157
BP	GO:0008211	glucocorticoid m	25	-0.589026574	-1.776337373
BP	GO:0051181	cofactor transpo	41	-0.589173821	-1.867304302
BP	GO:0006637	acyl-CoA metab	104	-0.589513426	-2.050139924
BP	GO:0035383	thioester metabo	104	-0.589513426	-2.050139924
BP	GO:0032369	negative regulat	40	-0.589710711	-1.885560214
BP	GO:0034198	cellular response	42	-0.589877868	-1.889024161
BP	GO:0098927	vesicle-mediated	41	-0.591971099	-1.876169882
BP	GO:0009062	fatty acid catabo	107	-0.59244115	-2.061640059
MF	GO:0008395	steroid hydroxy	38	-0.592580227	-1.848541221
BP	GO:0099625	ventricular cardi	28	-0.593268895	-1.907278525
BP	GO:0033344	cholesterol efflu	53	-0.595237234	-1.893506278
MF	GO:0008028	monocarboxylic	51	-0.595263184	-1.887736613
CC	GO:0032994	protein-lipid con	39	-0.595743032	-1.893860952
BP	GO:0015701	bicarbonate tran	42	-0.596259599	-1.909461009
BP	GO:0048821	erythrocyte deve	33	-0.600629055	-1.933407043
CC	GO:0034358	plasma lipoprote	37	-0.602137902	-1.883431313
CC	GO:1990777	lipoprotein parti	37	-0.602137902	-1.883431313
BP	GO:0045022	early endosome	38	-0.608785371	-1.899092816
BP	GO:0034453	microtubule anc	26	-0.614353866	-1.85758226
BP	GO:0060307	regulation of ve	24	-0.616203929	-1.82427294
CC	GO:0034364	high-density lipo	26	-0.618739081	-1.870841554
CC	GO:0005777	peroxisome	133	-0.619012671	-2.237158996
CC	GO:0042579	microbody	133	-0.619012671	-2.237158996
MF	GO:1901567	fatty acid deriva	27	-0.61922573	-1.911390498

BP	GO:0051004	regulation of lip	23	-0.621826667	-1.830087471
BP	GO:0009065	glutamine family	27	-0.621909994	-1.919676129
BP	GO:0006730	one-carbon meta	27	-0.625157444	-1.929700173
BP	GO:0032528	microvillus orga	24	-0.626517192	-1.854805376
BP	GO:0097066	response to thyr	26	-0.627544146	-1.897464861
MF	GO:0030371	translation repre	26	-0.62950014	-1.903379076
BP	GO:0006063	uronic acid meta	24	-0.62969352	-1.864208901
BP	GO:0019585	glucuronate met	24	-0.62969352	-1.864208901
MF	GO:0051287	NAD binding	56	-0.631848926	-2.15572578
BP	GO:0009074	aromatic amino	28	-0.633188022	-2.03561307
BP	GO:0002374	cytokine secretio	19	-0.63597525	-1.816196981
BP	GO:0072376	protein activatio	27	-0.636185899	-1.963742175
BP	GO:0072378	blood coagulatio	27	-0.636185899	-1.963742175
BP	GO:0009063	cellular amino a	124	-0.637688776	-2.219223629
BP	GO:0046653	tetrahydrofolate	19	-0.639189586	-1.825376375
MF	GO:0015248	sterol transporte	31	-0.639358123	-2.039756113
MF	GO:0034593	phosphatidylino	23	-0.643485429	-1.893831005
BP	GO:0034377	plasma lipoprote	27	-0.645578955	-1.99273612
MF	GO:0001223	transcription coa	23	-0.645897972	-1.900931321
BP	GO:0000423	mitophagy	22	-0.647179806	-1.831341877
BP	GO:0065005	protein-lipid con	31	-0.649706119	-2.072769519
MF	GO:0016790	thiolester hydro	36	-0.650856973	-1.99728812
BP	GO:1901606	alpha-amino aci	105	-0.65141277	-2.060024258
BP	GO:0003081	regulation of sy	25	-0.652045849	-1.966385663
BP	GO:0042759	long-chain fatty	29	-0.652880054	-2.139728318
BP	GO:0008053	mitochondrial fu	22	-0.653276353	-1.848593437
CC	GO:0031306	intrinsic compon	21	-0.655882135	-1.863134945
BP	GO:0050892	intestinal absorp	38	-0.65660189	-2.048255416
MF	GO:0070001	aspartic-type pe	25	-0.656735031	-1.980526908
BP	GO:0045940	positive regulati	30	-0.659384018	-2.105078742
BP	GO:0034367	protein-containi	29	-0.66028873	-2.16400928
BP	GO:0034368	protein-lipid con	28	-0.661984507	-2.128189839
BP	GO:0034369	plasma lipoprote	28	-0.661984507	-2.128189839
BP	GO:0090140	regulation of mi	22	-0.667381919	-1.888508332
MF	GO:0034185	apolipoprotein b	17	-0.667623485	-1.816291709
BP	GO:0098856	intestinal lipid a	17	-0.66815497	-1.817737632
BP	GO:0006699	bile acid biosyn	36	-0.670787223	-2.058448179
BP	GO:0050820	positive regulati	27	-0.672239412	-2.075030093
MF	GO:0004190	aspartic-type en	24	-0.672263248	-1.990236665
BP	GO:0008206	bile acid metabo	47	-0.674994132	-2.208772929
BP	GO:0090383	phagosome acid	28	-0.678355049	-2.180818899
BP	GO:0007031	peroxisome orga	80	-0.678853725	-2.426421042
BP	GO:0030194	positive regulati	26	-0.680640246	-2.058008124
BP	GO:1900048	positive regulati	26	-0.680640246	-2.058008124
MF	GO:0044769	ATPase activity,	22	-0.680911841	-1.926794313
MF	GO:0046961	proton-transport	22	-0.680911841	-1.926794313

BP	GO:0006677	glycosylceramid	20	-0.680913237	-1.897352389
BP	GO:0009068	aspartate family	23	-0.681099856	-2.004533387
BP	GO:0043574	peroxisomal tra	70	-0.683027607	-2.399085061
BP	GO:0006625	protein targeting	68	-0.684094632	-2.445321171
BP	GO:0072662	protein localizat	68	-0.684094632	-2.445321171
BP	GO:0072663	establishment of	68	-0.684094632	-2.445321171
CC	GO:0042611	MHC protein co	23	-0.686916618	-2.021652598
CC	GO:0033176	proton-transport	26	-0.687880009	-2.07989853
BP	GO:0070989	oxidative demet	19	-0.689505392	-1.969066579
MF	GO:0008308	voltage-gated ar	17	-0.690846507	-1.879470708
BP	GO:0032372	negative regulat	24	-0.691328135	-2.0466783
BP	GO:0032375	negative regulat	24	-0.691328135	-2.0466783
MF	GO:0050811	GABA receptor	17	-0.695363047	-1.891758104
BP	GO:1901998	toxin transport	37	-0.69589882	-2.176706739
BP	GO:0042168	heme metabolic	31	-0.697170047	-2.224194571
BP	GO:0006084	acetyl-CoA meta	38	-0.698694981	-2.179563903
BP	GO:0006067	ethanol metabol	21	-0.702215328	-1.994751569
BP	GO:1904469	positive regulati	20	-0.702852733	-1.958486397
CC	GO:0031526	brush border me	53	-0.702911608	-2.236028708
MF	GO:0000062	fatty-acyl-CoA b	21	-0.707779465	-2.010557361
MF	GO:0120015	sterol transfer ac	19	-0.709388204	-2.025847255
BP	GO:0060044	negative regulat	20	-0.712210493	-1.984561627
MF	GO:0015106	bicarbonate tran	19	-0.712251211	-2.034023335
CC	GO:0036019	endolysosome	20	-0.714373427	-1.990588602
CC	GO:0016471	vacuolar proton	17	-0.717631844	-1.9523411
BP	GO:0002418	immune respons	17	-0.71774631	-1.952652508
BP	GO:0034375	high-density lip	18	-0.718117866	-2.003150228
MF	GO:0120020	cholesterol trans	18	-0.724838371	-2.021896707
BP	GO:0002347	response to tum	23	-0.725332872	-2.134714822
BP	GO:0034380	high-density lip	14	-0.72670369	-1.932540615
BP	GO:0043691	reverse choleste	17	-0.730948257	-1.988568841
BP	GO:0006099	tricarboxylic aci	34	-0.73134211	-2.337681617
MF	GO:0010181	FMN binding	16	-0.733735118	-1.997579392
BP	GO:0006778	porphyrin-conta	38	-0.733806768	-2.289094367
MF	GO:0005402	carbohydrate:ca	19	-0.738049379	-2.107696883
CC	GO:0034361	very-low-density	20	-0.738238438	-2.057088022
CC	GO:0034385	triglyceride-rich	20	-0.738238438	-2.057088022
MF	GO:0055102	lipase inhibitor a	17	-0.738571746	-2.009308794
BP	GO:0034433	steroid esterifica	17	-0.740455327	-2.014433137
BP	GO:0034434	sterol esterificati	17	-0.740455327	-2.014433137
BP	GO:0034435	cholesterol ester	17	-0.740455327	-2.014433137
BP	GO:0034370	triglyceride-rich	14	-0.741754936	-1.972566755
BP	GO:0002834	regulation of res	14	-0.743781662	-1.977956477
BP	GO:0002837	regulation of im	14	-0.743781662	-1.977956477
CC	GO:0045239	tricarboxylic aci	14	-0.74420483	-1.979081819
BP	GO:0036092	phosphatidylino	14	-0.7446039	-1.980143073

BP	GO:0097067	cellular respons	16	-0.746562137	-2.032500701
CC	GO:0034362	low-density lipo	14	-0.747384257	-1.987536945
MF	GO:0000900	translation repre	15	-0.750670867	-2.000303532
BP	GO:0015669	gas transport	18	-0.751689697	-2.096797001
BP	GO:0006085	acetyl-CoA bios	22	-0.755536458	-2.137961572
BP	GO:0006086	acetyl-CoA bios	16	-0.757099349	-2.061188051
MF	GO:0008179	adenylate cyclas	13	-0.757346671	-1.952129979
BP	GO:0006000	fructose metabo	15	-0.758128205	-2.020175009
BP	GO:0046415	urate metabolic	13	-0.758934463	-1.956222657
MF	GO:0047617	acyl-CoA hydro	19	-0.759555828	-2.169114285
BP	GO:0072350	tricarboxylic aci	14	-0.761226968	-2.0243492
BP	GO:0001991	regulation of sy	18	-0.761302455	-2.12361126
CC	GO:0046930	pore complex	23	-0.763322706	-2.246522054
BP	GO:0006553	lysine metabolic	13	-0.765873491	-1.974108633
MF	GO:0005542	folic acid bindin	12	-0.767055453	-1.947212556
BP	GO:0009081	branched-chain	21	-0.771543211	-2.191688174
BP	GO:0009083	branched-chain	21	-0.771543211	-2.191688174
MF	GO:0016289	CoA hydrolase a	21	-0.773272442	-2.196600321
BP	GO:0006544	glycine metabol	17	-0.774256011	-2.106389013
BP	GO:0006554	lysine catabolic	12	-0.774719464	-1.966668071
BP	GO:0006103	2-oxoglutarate r	16	-0.775978245	-2.112585473
BP	GO:0018206	peptidyl-methio	13	-0.778423346	-2.006457026
CC	GO:0005782	peroxisomal ma	51	-0.778975809	-2.470337821
CC	GO:0031907	microbody lume	51	-0.778975809	-2.470337821
BP	GO:0043116	negative regulat	13	-0.781276943	-2.013812434
BP	GO:0034372	very-low-densit	12	-0.786217216	-1.99585575
MF	GO:0060229	lipase activator a	14	-0.790405141	-2.101943417
BP	GO:0006525	arginine metabo	19	-0.794344081	-2.268461423
BP	GO:0051917	regulation of fib	14	-0.794882721	-2.113850752
BP	GO:0009437	carnitine metabo	13	-0.802025338	-2.067293309
BP	GO:0006069	ethanol oxidatio	12	-0.802880965	-2.038157593
MF	GO:0019203	carbohydrate ph	10	-0.815921269	-2.021438333
MF	GO:0050308	sugar-phosphata	10	-0.815921269	-2.021438333
BP	GO:0002002	regulation of an	11	-0.818163407	-2.06531305
BP	GO:0002003	angiotensin matu	11	-0.818163407	-2.06531305
BP	GO:0006577	amino-acid beta	17	-0.823831102	-2.241259685
BP	GO:0046185	aldehyde catabo	11	-0.826685226	-2.08682492
BP	GO:0007597	blood coagulatio	18	-0.828847496	-2.31202443
BP	GO:0051918	negative regulat	10	-0.831182737	-2.059248495
MF	GO:0018455	alcohol dehydro	10	-0.832552577	-2.062642265
BP	GO:0033700	phospholipid ef	12	-0.833919457	-2.116950518
BP	GO:0034384	high-density lip	16	-0.837359484	-2.279694688
MF	GO:0016290	palmitoyl-CoA h	13	-0.83997544	-2.165113151
BP	GO:1902260	negative regulat	12	-0.840047056	-2.132505764
MF	GO:0102991	myristoyl-CoA h	10	-0.848199007	-2.101406167
CC	GO:0042627	chylomicron	13	-0.848533074	-2.187171231

BP	GO:0035376	sterol import	10	-0.888756359	-2.201886679
BP	GO:0070508	cholesterol impo	10	-0.888756359	-2.201886679

pvalue	p.adjust	qvalues	leading_edge	core_enrichmen	rank
0.002795213	0.046310038	0.04152423	tags=20%, list=5	10551/155465	1683
7.36E-08	1.04E-05	9.36E-06	tags=77%, list=1	28409/3497/35[illegible]	7047
1.00E-10	3.85E-08	3.45E-08	tags=75%, list=2	28773/28883/2[illegible]	7427
7.57E-08	1.06E-05	9.47E-06	tags=72%, list=1	28409/3497/35[illegible]	7047
5.55E-07	6.10E-05	5.47E-05	tags=64%, list=1	28409/3497/35[illegible]	6994
3.47E-09	7.59E-07	6.80E-07	tags=50%, list=1	28883/28896/2[illegible]	7047
1.06E-05	0.000732095	0.000656438	tags=57%, list=2	219437/390148	7958
7.52E-07	7.60E-05	6.82E-05	tags=38%, list=2	28883/28896/2[illegible]	7427
7.52E-07	7.60E-05	6.82E-05	tags=38%, list=2	28883/28896/2[illegible]	7427
3.21E-06	0.000265083	0.000237689	tags=62%, list=1	28883/28896/2[illegible]	6994
4.30E-07	4.90E-05	4.39E-05	tags=37%, list=2	28883/28896/2[illegible]	7427
4.26E-05	0.002253552	0.002020663	tags=43%, list=1	343702/28409/[illegible]	6149
1.89E-06	0.0001682	0.000150818	tags=38%, list=2	28883/28896/2[illegible]	7427
2.98E-05	0.001659356	0.001487873	tags=42%, list=1	343702/28409/[illegible]	6994
1.88E-05	0.001131601	0.001014658	tags=42%, list=2	28409/3497/35[illegible]	8382
1.59E-05	0.000983456	0.000881823	tags=56%, list=1	28883/28896/2[illegible]	6994
8.00E-05	0.00369419	0.003312422	tags=39%, list=1	28409/3497/35[illegible]	6149
1.86E-06	0.000166948	0.000149695	tags=27%, list=1	28883/28896/2[illegible]	5751
9.29E-05	0.004057883	0.00363853	tags=40%, list=1	343702/28409/[illegible]	6994
1.00E-10	3.85E-08	3.45E-08	tags=53%, list=2	343406/219437	8376
1.00E-10	3.85E-08	3.45E-08	tags=53%, list=2	343406/219437	8376
0.000462437	0.012892415	0.011560077	tags=49%, list=2	23581/126638/[illegible]	7976
1.48E-05	0.000928001	0.000832099	tags=38%, list=2	28773/28883/2[illegible]	8382
1.00E-10	3.85E-08	3.45E-08	tags=54%, list=2	343406/219437	9040
2.37E-10	7.36E-08	6.60E-08	tags=56%, list=2	343406/219437	10065
0.00030145	0.009748888	0.008741411	tags=34%, list=2	28409/3623/34[illegible]	8382
0.000147694	0.005591095	0.005013296	tags=51%, list=2	23581/337975/[illegible]	8703
0.000453323	0.012726096	0.011410946	tags=43%, list=2	28883/28896/2[illegible]	8380
0.000596627	0.015168962	0.013601359	tags=43%, list=2	28883/28896/2[illegible]	8380
0.000883373	0.020347786	0.018244989	tags=32%, list=1	55079/5047/28[illegible]	6671
0.000430312	0.012337131	0.011062177	tags=42%, list=2	23581/337975/[illegible]	8703
0.001701106	0.032059313	0.028746214	tags=22%, list=1	28883/28896/2[illegible]	6857
0.002634331	0.044651078	0.040036712	tags=38%, list=2	23581/337975/[illegible]	8703
0.002830647	0.04670568	0.041878985	tags=22%, list=8	54097/3033/11[illegible]	2793
0.003024676	0.048811381	0.043767078	tags=18%, list=7	51085/7057/11[illegible]	2697
0.000959278	0.021603795	0.019371199	tags=31%, list=2	867/8493/2303[illegible]	7268
0.001835361	0.033909607	0.030405294	tags=31%, list=1	10554/2822/88[illegible]	5891
0.002945607	0.048014579	0.043052619	tags=55%, list=2	4641/6845/228[illegible]	9475
0.002368126	0.040998499	0.0367616	tags=19%, list=7	51085/7057/11[illegible]	2697
0.001653644	0.031384291	0.028140951	tags=19%, list=7	51085/7057/11[illegible]	2697
0.001518529	0.029512757	0.026462827	tags=22%, list=8	3033/11069/77[illegible]	2762
0.001465664	0.029043853	0.02604238	tags=19%, list=7	51085/7057/11[illegible]	2697
0.000888143	0.020399531	0.018291387	tags=19%, list=6	23327/154/656[illegible]	2194
0.000544867	0.014480936	0.012984436	tags=33%, list=1	57678/54332/9[illegible]	5804

0.001483345	0.029131024	0.026120543	tags=41%, list=2	3326/1026/107	8948
0.001283189	0.026660192	0.023905054	tags=33%, list=1	57678/54332/9	5804
0.002954322	0.048059748	0.043093121	tags=24%, list=1	4953/407032/3	4946
0.001428826	0.028453351	0.025512902	tags=99%, list=6	6636/55692/51	22560
5.88E-05	0.002879269	0.002581717	tags=44%, list=2	10105/22885/1	9437
0.000565598	0.014656592	0.013141939	tags=38%, list=2	7917/10297/29	8178
0.000122164	0.004938492	0.004428134	tags=21%, list=9	7049/1991/717	3365
0.000309864	0.009829573	0.008813758	tags=33%, list=1	9731/1719/484	4529
0.001340315	0.02736456	0.024536631	tags=52%, list=6	23107/51069/1	24280
4.81E-05	0.002511297	0.002251773	tags=37%, list=1	26122/3054/28	6648
0.00250934	0.043044198	0.038595891	tags=39%, list=2	23165/2821/29	7391
8.86E-05	0.003980163	0.003568842	tags=37%, list=1	55204/27236/8	6824
0.001187371	0.025130612	0.022533544	tags=30%, list=1	6095/10993/36	4236
3.74E-05	0.002029899	0.001820124	tags=25%, list=1	1719/10935/48	4508
0.002512908	0.043044198	0.038595891	tags=31%, list=1	9258/4779/425	6080
7.33E-05	0.003447564	0.003091283	tags=25%, list=8	4790/1645/155	3053
0.001181563	0.025130612	0.022533544	tags=41%, list=1	84612/10015/3	6523
0.000851689	0.020017174	0.017948544	tags=13%, list=5	2254/6422/273	1716
0.001740209	0.032493282	0.029135337	tags=98%, list=6	24140/80324/1	21880
9.73E-05	0.004164143	0.003733809	tags=31%, list=1	883/4524/2008	3845
8.53E-05	0.003873089	0.003472833	tags=29%, list=1	51074/4953/88	5011
0.002059348	0.037164797	0.033324084	tags=40%, list=1	9488/55062/22	4375
0.001319353	0.02708651	0.024287315	tags=38%, list=1	90952/54532/6	6096
0.001903163	0.034733798	0.031144311	tags=35%, list=1	653308/89869/	5909
0.002892846	0.047441497	0.042538761	tags=38%, list=1	90952/54532/6	6096
0.000309515	0.009829573	0.008813758	tags=30%, list=1	183/5166/478/1	4202
0.00027671	0.00916886	0.008221325	tags=55%, list=2	8724/23258/56	9404
3.25E-06	0.000265093	0.000237697	tags=32%, list=1	51422/2820/84	6227
0.000278378	0.009186487	0.00823713	tags=99%, list=6	24140/54938/8	21880
0.001038873	0.023011752	0.020633654	tags=50%, list=2	54872/8776/52	9306
2.92E-05	0.001649606	0.001479131	tags=21%, list=1	594/4524/5660	3754
0.00109445	0.023980031	0.021501868	tags=38%, list=1	9818/5211/230/	7064
0.000728924	0.017487679	0.015680454	tags=33%, list=1	79689/538/567/	5277
1.10E-05	0.000737923	0.000661664	tags=52%, list=2	9382/51272/63	10138
0.000299955	0.00973949	0.008732984	tags=26%, list=1	1571/1036/552	3526
0.000473763	0.013117705	0.011762085	tags=39%, list=1	131870/9818/5	7073
6.01E-05	0.002927712	0.002625155	tags=31%, list=1	2822/57678/40	4300
0.00060838	0.015275637	0.013697011	tags=21%, list=7	51109/573971/	2645
0.000564769	0.014656592	0.013141939	tags=29%, list=8	6330/254428/6	2882
0.000585397	0.014977647	0.013429816	tags=39%, list=1	131870/9818/5	7073
0.000585397	0.014977647	0.013429816	tags=39%, list=1	131870/9818/5	7073
0.000312355	0.009829573	0.008813758	tags=26%, list=8	1645/1558/328	3018
2.36E-05	0.001371993	0.001230207	tags=43%, list=1	345778/51309/	5804
0.000404548	0.011723205	0.010511696	tags=38%, list=1	55258/23042/1	4730
0.000499024	0.013676632	0.012263251	tags=33%, list=9	51474/6446/11	3118
0.0029738	0.048182705	0.043203371	tags=31%, list=2	4688/114112/4	7149

5.82E-05	0.002867764	0.002571402	tags=25%, list=1	4846/8428/384	4498
0.002141825	0.038396136	0.034428173	tags=16%, list=8	407019/6653/9	2932
0.00045502	0.012729533	0.011414028	tags=35%, list=1	51493/10352/7	6028
2.76E-05	0.001574186	0.001411505	tags=55%, list=2	8517/823/2724	9354
0.001326417	0.027149566	0.024343854	tags=60%, list=2	22937/81562/9	9925
3.19E-05	0.001762592	0.001580441	tags=32%, list=9	51660/6754/32	3330
8.65E-06	0.000617938	0.000554079	tags=51%, list=2	355/27244/748	7268
0.000603067	0.015236855	0.013662236	tags=35%, list=1	361/22841/113	4457
0.000147374	0.005591095	0.005013296	tags=27%, list=1	6777/94081/92/	4617
0.000704676	0.017025448	0.015265991	tags=30%, list=1	84896/3190/42	5291
0.001302609	0.026935028	0.024151488	tags=39%, list=1	57678/407031/	5804
0.000560175	0.014652754	0.013138498	tags=29%, list=1	6342/406987/5	4165
0.001108562	0.024124353	0.021631276	tags=34%, list=1	2896/80273/96	6691
0.000224493	0.007752269	0.006951128	tags=37%, list=1	4090/9991/143	4617
1.76E-05	0.001067482	0.000957166	tags=28%, list=1	2321/9619/138	4366
0.001147863	0.024551506	0.022014285	tags=100%, list=	54938/80324/7	21880
0.000882398	0.020347786	0.018244989	tags=22%, list=9	10365/687/546	3262
0.000821466	0.019363134	0.017362094	tags=34%, list=1	407032/3638/5	4878
0.000942318	0.021400669	0.019189064	tags=46%, list=2	60678/1981/19	9525
6.01E-06	0.000454362	0.000407407	tags=22%, list=6	9420/1645/155	2027
1.32E-07	1.69E-05	1.52E-05	tags=35%, list=1	5207/405/3479/	6422
9.39E-05	0.004060983	0.00364131	tags=28%, list=1	406/3337/1027/	6323
0.000337168	0.010248131	0.009189061	tags=36%, list=1	5547/4846/135	4568
8.74E-05	0.003947358	0.003539427	tags=32%, list=1	2542/56910/91	4300
0.001166369	0.024881521	0.022310196	tags=15%, list=7	3050/2938/842	2441
0.001834097	0.033909607	0.030405294	tags=36%, list=2	998/221178/88	7217
0.000698536	0.017019778	0.015260907	tags=27%, list=6	9619/114882/6	2203
0.000108624	0.004598034	0.00412286	tags=30%, list=8	407032/5727/6	3053
2.21E-05	0.001294526	0.001160746	tags=32%, list=8	2239/1645/558	3066
2.07E-07	2.54E-05	2.28E-05	tags=38%, list=1	4724/54205/56	7000
0.000480313	0.013208617	0.011843602	tags=56%, list=2	6845/64083/23	9441
0.000593648	0.01514084	0.013576144	tags=36%, list=8	2239/1645/222	3066
0.001387989	0.027984759	0.025092736	tags=40%, list=1	91689/10440/5	7103
1.92E-05	0.001144114	0.001025878	tags=29%, list=9	9368/113235/1	3147
0.000135239	0.005333704	0.004782504	tags=42%, list=2	219970/9397/6	7505
0.001479165	0.029131024	0.026120543	tags=37%, list=1	79689/538/567/	5277
0.000325184	0.010091993	0.009049059	tags=40%, list=1	1977/546/3877	6949
2.37E-06	0.000201616	0.00018078	tags=17%, list=2	2220/922/3078/	618
0.000698895	0.017019778	0.015260907	tags=15%, list=5	55966/634/204	1763
1.99E-06	0.000175035	0.000156947	tags=28%, list=1	4846/3848/231	4498
6.36E-05	0.003061333	0.002744967	tags=35%, list=8	2239/1645/558	3066
9.35E-05	0.004060983	0.00364131	tags=30%, list=1	119391/538/12	5206
7.31E-05	0.003447564	0.003091283	tags=36%, list=8	2239/1645/558	3066
0.000520078	0.013969533	0.012525883	tags=44%, list=1	1837/55970/83	6338
0.001408151	0.028180445	0.025268199	tags=48%, list=2	3326/2060/507	8948
2.51E-05	0.0014478	0.00129818	tags=40%, list=1	529/55862/683	5558

0.000226302	0.007752269	0.006951128	tags=40%, list=1	529/6833/1092	5558
2.64E-07	3.19E-05	2.86E-05	tags=33%, list=1	23205/3155/84	4394
1.45E-06	0.000136399	0.000122303	tags=31%, list=1	4023/9619/609	4397
3.18E-06	0.000264746	0.000237386	tags=48%, list=2	5162/4928/419	7762
5.24E-05	0.00264815	0.002374483	tags=35%, list=9	51474/6446/11	3344
9.07E-05	0.003995701	0.003582775	tags=47%, list=2	6811/11337/66	8980
7.47E-05	0.003492782	0.003131828	tags=11%, list=3	6812/2153/195	1048
0.000651014	0.016195227	0.014521568	tags=49%, list=2	57805/23511/3	9379
0.002923423	0.047749248	0.042814709	tags=37%, list=1	3192/8202/692	7040
0.002923423	0.047749248	0.042814709	tags=37%, list=1	3192/8202/692	7040
2.24E-06	0.000194952	0.000174805	tags=24%, list=1	3482/3249/591	3677
0.002701742	0.04513138	0.040467378	tags=42%, list=1	4088/5747/387	4761
0.002701742	0.04513138	0.040467378	tags=42%, list=1	4088/5747/387	4761
0.002701742	0.04513138	0.040467378	tags=42%, list=1	4088/5747/387	4761
0.002999241	0.048497726	0.043485836	tags=58%, list=2	1386/284058/5	9351
2.70E-06	0.000227185	0.000203707	tags=24%, list=1	3482/3249/591	3677
2.19E-08	3.69E-06	3.31E-06	tags=38%, list=1	653308/89869/	5909
1.51E-06	0.000140649	0.000126114	tags=51%, list=2	6720/355/2724	9327
1.10E-05	0.000737923	0.000661664	tags=32%, list=1	406987/50640/	4060
0.000312243	0.009829573	0.008813758	tags=23%, list=6	9619/1389/394	2060
0.002421908	0.041840017	0.037516154	tags=45%, list=1	5927/8864/319	7040
3.99E-09	8.50E-07	7.62E-07	tags=28%, list=1	9965/2222/315	3767
0.002185385	0.038662659	0.034667152	tags=39%, list=1	2806/1491/274	6088
0.000206633	0.007283466	0.006530773	tags=52%, list=2	23369/1981/56	9520
1.28E-07	1.66E-05	1.49E-05	tags=34%, list=1	55258/3638/23	4730
3.50E-05	0.001912741	0.001715073	tags=47%, list=1	8402/230/1077	6982
0.000114907	0.004788778	0.004293892	tags=28%, list=8	254428/1183/2	2858
0.002602117	0.044197717	0.039630202	tags=45%, list=1	4301/7124/846	6523
1.92E-10	6.20E-08	5.56E-08	tags=36%, list=1	2581/3949/634	4228
1.00E-10	3.85E-08	3.45E-08	tags=51%, list=2	4338/10327/27	8544
0.0002695	0.008966694	0.008040051	tags=28%, list=8	4129/6531/570	2988
5.90E-10	1.44E-07	1.30E-07	tags=32%, list=8	6506/345274/6	2741
1.00E-10	3.85E-08	3.45E-08	tags=32%, list=8	254428/26503/	2858
1.00E-10	3.85E-08	3.45E-08	tags=33%, list=8	254428/26503/	2858
1.00E-10	3.85E-08	3.45E-08	tags=35%, list=1	55258/3638/23	4730
0.001418226	0.028311987	0.025386148	tags=42%, list=1	3192/8202/123	7040
1.56E-05	0.000970517	0.000870221	tags=21%, list=7	51109/573971/	2645
0.001989351	0.036062559	0.032335754	tags=27%, list=8	4790/407019/5	3053
0.000187074	0.006772593	0.006072695	tags=47%, list=2	11337/665/576	8927
0.000187074	0.006772593	0.006072695	tags=47%, list=2	11337/665/576	8927
0.002850885	0.046943792	0.04209249	tags=48%, list=2	2074/6924/582	8463
6.56E-08	9.82E-06	8.81E-06	tags=30%, list=6	6568/23428/10	2072
6.56E-08	9.82E-06	8.81E-06	tags=30%, list=6	6568/23428/10	2072
8.71E-06	0.000617938	0.000554079	tags=38%, list=8	57210/26503/6	2979
0.000117046	0.004803649	0.004307227	tags=42%, list=1	2806/1491/274	6088
6.56E-06	0.000490935	0.000440201	tags=31%, list=9	9420/1645/155	3158

8.05E-07	7.94E-05	7.12E-05	tags=41%, list=1 57678/407031/[illegible]	5804
0.001673584	0.031614316	0.028347205	tags=20%, list=6 406959/9104/5[illegible]	2060
5.54E-05	0.002767237	0.002481263	tags=49%, list=1 8402/230/1077[illegible]	6982
1.39E-06	0.000133411	0.000119624	tags=20%, list=4 7450/7057/271[illegible]	1613
0.002723457	0.045213863	0.040541337	tags=49%, list=2 2821/3945/521[illegible]	7341
0.001051757	0.023233495	0.020832481	tags=40%, list=1 57678/407031/[illegible]	5804
0.000192887	0.00687001	0.006160044	tags=50%, list=1 8402/230/1077[illegible]	6982
3.40E-08	5.39E-06	4.83E-06	tags=36%, list=8 57210/26503/6[illegible]	2979
0.002258616	0.039697634	0.035595171	tags=40%, list=1 285172/200576	5758
0.001446943	0.028743331	0.025772916	tags=41%, list=7 55258/9731/48[illegible]	2469
0.000348137	0.010386287	0.009312939	tags=31%, list=5 4846/3848/231[illegible]	1763
5.08E-06	0.000391195	0.000350768	tags=37%, list=1 1203/55647/91[illegible]	5140
5.80E-05	0.002867764	0.002571402	tags=54%, list=2 7384/4697/158/	8528
5.08E-06	0.000391195	0.000350768	tags=30%, list=1 10998/1571/32[illegible]	3630
1.00E-10	3.85E-08	3.45E-08	tags=30%, list=8 286/254428/36[illegible]	2903
0.000705445	0.017025448	0.015265991	tags=61%, list=2 3257/950/8431[illegible]	9300
0.000705445	0.017025448	0.015265991	tags=61%, list=2 3257/950/8431[illegible]	9300
1.51E-08	2.78E-06	2.50E-06	tags=54%, list=2 4338/10327/27[illegible]	8544
9.73E-08	1.31E-05	1.18E-05	tags=26%, list=8 6653/392636/2[illegible]	2837
0.001129808	0.024358671	0.021841378	tags=65%, list=3 84678/6045/55[illegible]	11161
1.47E-07	1.85E-05	1.66E-05	tags=28%, list=8 139189/6622/6[illegible]	3077
3.11E-10	8.65E-08	7.76E-08	tags=32%, list=1 1571/1036/882[illegible]	3526
7.18E-08	1.04E-05	9.29E-06	tags=32%, list=7 9619/114882/2[illegible]	2731
3.71E-07	4.35E-05	3.90E-05	tags=37%, list=1 650/84869/284[illegible]	4218
0.002000292	0.036179774	0.032440856	tags=24%, list=8 6653/7067/234[illegible]	2837
6.55E-05	0.003131466	0.002807852	tags=42%, list=8 6531/6506/345[illegible]	2763
0.000864276	0.020079516	0.018004443	tags=32%, list=7 3050/128/2728[illegible]	2441
0.000809302	0.01913218	0.017155008	tags=48%, list=2 113251/1656/5[illegible]	8564
0.000986957	0.02204295	0.019764971	tags=40%, list=1 200895/501/55[illegible]	3694
0.000332353	0.010195423	0.0091418	tags=49%, list=1 216/2806/2600[illegible]	6155
0.001350148	0.02736456	0.024536631	tags=43%, list=1 529/538/9114/5	5184
0.00034715	0.010386287	0.009312939	tags=13%, list=2 2153/1950/534[illegible]	745
3.30E-06	0.000266428	0.000238895	tags=24%, list=8 407019/406977	2932
0.000685425	0.016792922	0.015057495	tags=36%, list=1 55788/1355/17[illegible]	4024
0.000116288	0.004796861	0.00430114	tags=44%, list=1 91689/10440/1[illegible]	7103
0.000618937	0.015492592	0.013891545	tags=42%, list=1 538/9114/5160[illegible]	5184
0.000293152	0.009557001	0.008569354	tags=73%, list=2 2890/3134/234[illegible]	9925
2.25E-08	3.72E-06	3.33E-06	tags=26%, list=8 1558/6392/156[illegible]	2962
0.001022231	0.022767882	0.020414986	tags=66%, list=2 9550/79641/23[illegible]	8610
9.85E-06	0.000692216	0.000620681	tags=38%, list=1 1203/55647/91[illegible]	5140
1.00E-10	3.85E-08	3.45E-08	tags=43%, list=1 163404/30/228[illegible]	5888
0.0008722	0.020205554	0.018117456	tags=43%, list=1 23475/57017/8[illegible]	6632
1.89E-10	6.20E-08	5.56E-08	tags=36%, list=1 3953/8578/32/2	5166
0.001117986	0.024233023	0.021728715	tags=44%, list=1 1717/7881/232[illegible]	5687
2.72E-08	4.40E-06	3.94E-06	tags=36%, list=1 1571/1036/882[illegible]	3526
0.000115587	0.004792406	0.004297146	tags=45%, list=1 91689/10440/1[illegible]	7103

0.001715328	0.032252147	0.02891912	tags=47%, list=2	1385/8864/988/	7657
4.85E-06	0.000380523	0.000341199	tags=35%, list=8	1645/55825/222	3018
0.000578566	0.014897141	0.013357629	tags=48%, list=1	8720/7108/5562	5804
0.000578566	0.014897141	0.013357629	tags=48%, list=1	8720/7108/5562	5804
1.09E-05	0.000737923	0.000661664	tags=24%, list=4	2039/7099/7057	1574
4.91E-08	7.63E-06	6.85E-06	tags=38%, list=1	650/84869/2842	4218
1.77E-06	0.000160625	0.000144026	tags=37%, list=1	9619/6095/1148	4300
5.29E-09	1.04E-06	9.35E-07	tags=38%, list=1	55066/10157/54	4652
5.29E-09	1.04E-06	9.35E-07	tags=38%, list=1	55066/10157/54	4652
5.29E-09	1.04E-06	9.35E-07	tags=38%, list=1	55066/10157/54	4652
0.001407026	0.028180445	0.025268199	tags=49%, list=1	594/501/55711/	3754
1.55E-06	0.000142454	0.000127732	tags=46%, list=1	6389/3418/8801	5250
5.83E-07	6.29E-05	5.64E-05	tags=61%, list=2	55763/23041/58	8997
2.68E-09	6.20E-07	5.56E-07	tags=28%, list=8	3046/9420/1558	2962
0.000131257	0.005227644	0.004687405	tags=62%, list=2	6845/64083/557	9441
6.99E-06	0.000509298	0.000456666	tags=23%, list=4	2039/7099/7057	1574
4.93E-05	0.002535332	0.002273324	tags=24%, list=8	2039/7099/4069	2955
2.96E-09	6.65E-07	5.96E-07	tags=30%, list=8	4129/1558/5860	2988
0.000126533	0.005064449	0.004541075	tags=39%, list=9	3046/728441/28	3258
1.01E-06	9.82E-05	8.81E-05	tags=41%, list=1	1203/55647/911	4130
0.00107527	0.023688163	0.021240162	tags=61%, list=2	2639/84245/511	7818
0.001776182	0.033088558	0.029669095	tags=41%, list=8	1565/51109/125	2836
7.48E-07	7.60E-05	6.82E-05	tags=25%, list=6	3991/128486/91	2105
0.001375325	0.027798765	0.024925963	tags=38%, list=1	2820/26007/801	6215
7.19E-06	0.000518863	0.000465242	tags=24%, list=4	2039/7099/7057	1574
0.00052764	0.014125735	0.012665942	tags=20%, list=4	634/84830/18/5	1553
2.32E-06	0.000199692	0.000179055	tags=42%, list=1	51463/529/1203	5561
0.001140846	0.024466148	0.021937749	tags=38%, list=1	518/529/9114/5	5696
9.04E-08	1.24E-05	1.11E-05	tags=36%, list=1	407015/23411/9	4658
2.03E-08	3.49E-06	3.13E-06	tags=35%, list=8	3658/48/1645/4	3079
1.03E-05	0.000721237	0.000646702	tags=62%, list=1	10478/207/2639	4165
0.002363829	0.040998499	0.0367616	tags=60%, list=2	91392/9218/259	9362
0.002363829	0.040998499	0.0367616	tags=60%, list=2	91392/9218/259	9362
0.002363829	0.040998499	0.0367616	tags=60%, list=2	91392/9218/259	9362
6.94E-05	0.003300054	0.002959017	tags=27%, list=8	6653/8605/2160	2837
0.002275465	0.039907008	0.035782908	tags=31%, list=7	253512/80736/1	2411
7.46E-07	7.60E-05	6.82E-05	tags=36%, list=1	407015/23411/9	4658
0.000119447	0.004852898	0.004351386	tags=29%, list=1	9619/6095/1148	4300
4.85E-05	0.002512353	0.002252719	tags=28%, list=9	29116/407019/4	3223
0.000397761	0.011567975	0.010372509	tags=43%, list=1	11212/55258/23	4851
0.000397761	0.011567975	0.010372509	tags=43%, list=1	11212/55258/23	4851
1.12E-07	1.49E-05	1.33E-05	tags=55%, list=1	2475/1432/2340	6767
9.61E-05	0.004133157	0.003706025	tags=20%, list=4	634/84830/18/5	1553
0.000676463	0.0167254	0.014996951	tags=76%, list=2	64121/9451/561	10466
0.002690364	0.04513138	0.040467378	tags=65%, list=2	23258/163486/2	9346
1.73E-08	3.04E-06	2.72E-06	tags=56%, list=1	2475/1432/2340	6767

0.000235701	0.008006885	0.007179431	tags=21%, list=4 634/84830/18/5	1553
0.002156544	0.038404542	0.03443571	tags=55%, list=2 7529/8994/678	8402
1.00E-10	3.85E-08	3.45E-08	tags=42%, list=1 51074/26227/4	5011
5.86E-06	0.000447035	0.000400837	tags=31%, list=8 3482/8013/704	2739
0.000257139	0.008590771	0.007702977	tags=61%, list=2 2639/5162/109	7818
0.000257139	0.008590771	0.007702977	tags=61%, list=2 2639/5162/109	7818
1.26E-05	0.000807336	0.000723904	tags=54%, list=2 55229/2639/51	7832
1.26E-05	0.000807336	0.000723904	tags=54%, list=2 55229/2639/51	7832
1.26E-05	0.000807336	0.000723904	tags=54%, list=2 55229/2639/51	7832
0.000554691	0.014560635	0.013055898	tags=40%, list=1 9619/3949/634	4300
0.002205436	0.038932199	0.034908838	tags=70%, list=2 1024/400569/9	10750
0.001845092	0.033909607	0.030405294	tags=41%, list=1 84105/1719/88	4508
0.001845092	0.033909607	0.030405294	tags=15%, list=7 406977/5465/4	2695
0.000141219	0.005436942	0.004875074	tags=67%, list=2 9550/79641/23	8610
0.00139809	0.028118308	0.025212484	tags=40%, list=1 8801/23205/84	5074
0.000141219	0.005436942	0.004875074	tags=35%, list=1 4953/23042/31	4946
0.000211366	0.007397803	0.006633293	tags=30%, list=6 27284/1593/51	2161
0.00128602	0.026660192	0.023905054	tags=31%, list=1 1824/1832/287	3606
1.00E-10	3.85E-08	3.45E-08	tags=37%, list=1 3953/8578/32/2	5166
0.001033597	0.022957778	0.020585257	tags=31%, list=8 6653/8228/399	2837
1.90E-05	0.001137723	0.001020147	tags=67%, list=2 9382/6399/233	9580
1.00E-10	3.85E-08	3.45E-08	tags=37%, list=1 3953/8578/32/3	5166
4.95E-05	0.002535332	0.002273324	tags=39%, list=8 5727/407015/2	3053
0.001308346	0.026984636	0.024195969	tags=35%, list=5 5144/845/801/6	1759
0.000317103	0.009929495	0.008903354	tags=27%, list=0 9619/114882/6	144
4.51E-05	0.002368514	0.002123745	tags=40%, list=8 5727/407015/2	3053
0.0005123	0.013899151	0.012462774	tags=44%, list=1 2822/57678/96	5838
0.0005123	0.013899151	0.012462774	tags=44%, list=1 2822/57678/96	5838
6.09E-07	6.48E-05	5.81E-05	tags=48%, list=1 80724/25821/7	5464
1.94E-07	2.41E-05	2.16E-05	tags=44%, list=1 7108/403313/5	6688
0.001203135	0.025397781	0.022773104	tags=23%, list=7 80014/406977/	2695
1.45E-06	0.000136399	0.000122303	tags=31%, list=7 2729/58510/10	2450
3.20E-05	0.001762592	0.001580441	tags=29%, list=8 2039/7099/406	2955
1.34E-09	3.19E-07	2.86E-07	tags=38%, list=9 51474/9368/11	3344
0.00112833	0.024358671	0.021841378	tags=44%, list=1 2822/57678/96	5838
0.001186868	0.025130612	0.022533544	tags=16%, list=7 406977/5465/4	2695
0.003113709	0.049850168	0.044698514	tags=39%, list=7 292/57835/108	2440
0.003113709	0.049850168	0.044698514	tags=39%, list=7 292/57835/108	2440
5.77E-09	1.11E-06	9.96E-07	tags=42%, list=1 9969/64240/40	5161
1.69E-08	3.03E-06	2.72E-06	tags=42%, list=1 9969/64240/40	5161
0.000412017	0.011896719	0.010667279	tags=57%, list=1 51540/63826/2	6751
0.000628004	0.015671015	0.014051529	tags=62%, list=2 80853/22992/2	7956
0.00015324	0.005683235	0.005095914	tags=43%, list=5 200931/634/59	1677
0.000332912	0.010195423	0.0091418	tags=28%, list=1 26207/1071/50	3770
6.73E-08	9.89E-06	8.87E-06	tags=39%, list=1 57678/407031/	5804
1.00E-10	3.85E-08	3.45E-08	tags=50%, list=1 6342/23600/51	4165

0.000118032	0.004819645	0.004321569	tags=73%, list=3 9776/7249/947	11236
0.000334679	0.010210855	0.009155637	tags=31%, list=4 3043/58157/15	1603
0.001658503	0.031402797	0.028157545	tags=48%, list=1 529/9114/5160	5558
0.000467824	0.012997777	0.011654551	tags=56%, list=1 2806/2744/137	4678
0.000183546	0.006745331	0.00604825	tags=48%, list=1 79068/30812/8	5499
1.22E-05	0.000804973	0.000721785	tags=57%, list=2 6716/28976/26	7851
0.000533331	0.014230949	0.012760283	tags=49%, list=1 6527/66035/57	3820
5.58E-07	6.10E-05	5.47E-05	tags=40%, list=1 57678/407031/	5804
0.000863059	0.020079516	0.018004443	tags=69%, list=2 4924/3123/311	10041
0.002082189	0.037410004	0.03354395	tags=23%, list=7 406977/5465/7	2695
0.002082189	0.037410004	0.03354395	tags=23%, list=7 406977/5465/7	2695
0.000109458	0.0046092	0.004132873	tags=23%, list=4 3990/55937/18	1333
0.000105651	0.004495715	0.004031115	tags=59%, list=2 10981/23682/2	7707
6.91E-06	0.000507727	0.000455257	tags=53%, list=1 8799/5830/848	6200
6.91E-06	0.000507727	0.000455257	tags=53%, list=1 8799/5830/848	6200
0.000280434	0.009216688	0.00826421	tags=18%, list=2 729/3078/731/7	886
0.00051797	0.013959304	0.012516711	tags=51%, list=1 51004/2329/48	4786
0.001883952	0.034539111	0.030969744	tags=50%, list=1 8801/23205/84	5074
9.09E-05	0.003995701	0.003582775	tags=35%, list=8 6653/2166/822	2837
9.09E-05	0.003995701	0.003582775	tags=35%, list=8 6653/2166/822	2837
8.45E-05	0.003857641	0.003458982	tags=23%, list=4 3990/55937/18	1333
0.002888719	0.047441497	0.042538761	tags=17%, list=5 9472/55151/79	1759
0.003072912	0.049392636	0.044288264	tags=24%, list=3 866/653/3291/1	958
0.000140799	0.005436942	0.004875074	tags=39%, list=1 22/55640/5578	4561
4.55E-10	1.15E-07	1.03E-07	tags=46%, list=1 284486/32/880	5192
4.55E-10	1.15E-07	1.03E-07	tags=46%, list=1 284486/32/880	5192
0.000241075	0.008121212	0.007281943	tags=30%, list=8 4790/407019/7	3053
0.000111769	0.004682157	0.00419829	tags=76%, list=2 64121/9451/56	10466
0.000134808	0.005333704	0.004782504	tags=63%, list=2 8724/5604/259	9404
1.11E-10	4.07E-08	3.65E-08	tags=46%, list=1 6342/23600/51	4165
0.000326518	0.010091993	0.009049059	tags=32%, list=5 1593/51302/15	1786
0.001584121	0.030349797	0.027213365	tags=29%, list=1 10142/287/972	3844
1.44E-05	0.000912333	0.00081805	tags=26%, list=3 407019/100126	1185
1.18E-05	0.000782048	0.000701229	tags=45%, list=5 9122/347411/6	1851
0.000362892	0.010707955	0.009601366	tags=26%, list=4 3990/55937/40	1333
8.93E-05	0.003988074	0.003575935	tags=38%, list=6 57835/10861/8	2370
0.000855928	0.02002315	0.017953902	tags=42%, list=1 669/6886/1036	4006
0.000224532	0.007752269	0.006951128	tags=27%, list=4 3990/55937/40	1333
0.000224532	0.007752269	0.006951128	tags=27%, list=4 3990/55937/40	1333
0.000187792	0.006772593	0.006072695	tags=66%, list=2 8724/5604/259	9404
0.002550662	0.04350654	0.039010454	tags=62%, list=2 11127/23299/9	9324
0.001983837	0.036043418	0.032318592	tags=29%, list=1 10142/287/972	3844
0.002297183	0.040200707	0.036046255	tags=35%, list=4 3990/55937/54	1333
1.00E-10	3.85E-08	3.45E-08	tags=53%, list=1 8799/83752/58	6200
1.00E-10	3.85E-08	3.45E-08	tags=53%, list=1 8799/83752/58	6200
0.001349014	0.02736456	0.024536631	tags=44%, list=1 3155/6342/797	4359

0.00223094	0.039296625	0.035235603	tags=30%, list=7 338328/5125/2	2592
0.001231069	0.025852442	0.023180779	tags=63%, list=1 2806/2744/1378	6088
0.001083637	0.023807626	0.02134728	tags=59%, list=1 94081/27430/1	4570
0.001620788	0.030833099	0.027646721	tags=58%, list=9 5337/5911/9368	3147
0.001726859	0.032393625	0.029045978	tags=58%, list=1 5469/4005/2629	5792
0.00160012	0.030583862	0.027423241	tags=58%, list=1 1153/132864/2	5967
0.001542991	0.029734317	0.02666149	tags=54%, list=5 5581/51084/66	1948
0.001542991	0.029734317	0.02666149	tags=54%, list=5 5581/51084/66	1948
2.83E-07	3.37E-05	3.02E-05	tags=61%, list=1 4723/23409/21	6656
0.000561825	0.014652754	0.013138498	tags=46%, list=1 125061/23498/8	4747
0.002970939	0.048182705	0.043203371	tags=26%, list=0 2150/117854/28	144
0.000759289	0.018055452	0.016189551	tags=22%, list=2 2155/2160/350/	601
0.000759289	0.018055452	0.016189551	tags=22%, list=2 2155/2160/350/	601
1.00E-10	3.85E-08	3.45E-08	tags=53%, list=1 125061/7263/5	4747
0.002582587	0.043958343	0.039415566	tags=53%, list=1 1719/4524/2008	4508
0.000327038	0.010091993	0.009049059	tags=35%, list=1 9619/114882/1	4300
0.001109989	0.024124353	0.021631276	tags=87%, list=3 114971/5728/3	11079
0.000546492	0.014480936	0.012984436	tags=19%, list=3 55937/336/335/	1185
0.00094727	0.021452869	0.01923587	tags=48%, list=1 2308/9686/229	6527
0.002154971	0.038404542	0.03443571	tags=82%, list=3 57154/9776/72	11645
0.000207198	0.007283466	0.006530773	tags=19%, list=3 55937/6456/33	1185
0.000153196	0.005683235	0.005095914	tags=58%, list=2 2639/84945/55	7818
1.00E-10	3.85E-08	3.45E-08	tags=54%, list=1 125061/55258/	4747
0.000600701	0.015224655	0.013651297	tags=56%, list=1 1359/2150/183/	4386
0.000449618	0.012666061	0.011357115	tags=38%, list=1 1571/1558/156	3526
0.001845421	0.033909607	0.030405294	tags=68%, list=2 55669/115209/	8800
0.002471902	0.042612637	0.038208929	tags=52%, list=1 54332/9804/51	5783
2.94E-05	0.001649606	0.001479131	tags=50%, list=9 51474/113235/	3344
0.000510423	0.013899151	0.012462774	tags=24%, list=3 162540/643847	1008
0.000147025	0.005591095	0.005013296	tags=33%, list=6 406959/2246/1	2060
0.000377098	0.011086671	0.009940944	tags=34%, list=4 3990/55937/18	1333
0.000227246	0.007752269	0.006951128	tags=36%, list=4 3990/55937/18	1333
0.000227246	0.007752269	0.006951128	tags=36%, list=4 3990/55937/18	1333
0.001107733	0.024124353	0.021631276	tags=55%, list=1 64423/55737/2	5933
0.002716008	0.04518297	0.040513636	tags=41%, list=4 4023/4040/399	1333
0.002716008	0.04518297	0.040513636	tags=35%, list=1 948/1056/6424	285
5.07E-05	0.002576672	0.002310391	tags=53%, list=1 23411/6342/23	4165
0.00020256	0.00718288	0.006440581	tags=37%, list=8 2039/7099/705	2955
0.000516775	0.013959304	0.012516711	tags=25%, list=3 162540/643847	1008
7.82E-07	7.81E-05	7.00E-05	tags=47%, list=1 6342/23600/99	4165
0.00012307	0.004950369	0.004438784	tags=54%, list=1 529/55647/911	5558
1.00E-10	3.85E-08	3.45E-08	tags=61%, list=2 196743/5192/2	7275
0.000189314	0.006772593	0.006072695	tags=38%, list=8 2039/7099/705	2955
0.000189314	0.006772593	0.006072695	tags=38%, list=8 2039/7099/705	2955
0.000681231	0.0167409	0.015010849	tags=59%, list=1 529/9114/5160	5558
0.000681231	0.0167409	0.015010849	tags=59%, list=1 529/9114/5160	5558

0.002813025	0.046509833	0.041703377	tags=55%, list=1 5562/5660/262[illegible]	6331
0.000413479	0.011896719	0.010667279	tags=57%, list=1 10157/10993/5[illegible]	4600
1.90E-10	6.20E-08	5.56E-08	tags=64%, list=2 196743/5192/2[illegible]	7275
3.21E-10	8.65E-08	7.76E-08	tags=65%, list=2 196743/5192/2[illegible]	7275
3.21E-10	8.65E-08	7.76E-08	tags=65%, list=2 196743/5192/2[illegible]	7275
3.21E-10	8.65E-08	7.76E-08	tags=65%, list=2 196743/5192/2[illegible]	7275
0.000361434	0.010703996	0.009597815	tags=83%, list=2 3123/3115/310[illegible]	9634
0.000152423	0.005683235	0.005095914	tags=50%, list=1 9114/51606/53[illegible]	5048
0.000668158	0.016570724	0.014858259	tags=37%, list=8 79068/8846/15[illegible]	2962
0.001619529	0.030833099	0.027646721	tags=41%, list=1 1182/1185/118[illegible]	3686
0.000308221	0.009829573	0.008813758	tags=46%, list=8 4790/407019/1[illegible]	3053
0.000308221	0.009829573	0.008813758	tags=46%, list=8 4790/407019/1[illegible]	3053
0.001537007	0.029734317	0.02666149	tags=65%, list=1 11345/22906/2[illegible]	6784
4.64E-06	0.000368161	0.000330115	tags=35%, list=1 9619/664/9325/	4300
2.61E-05	0.001496388	0.001341747	tags=52%, list=1 1352/644/4779/	3976
4.44E-06	0.000355418	0.000318688	tags=68%, list=1 5162/51166/29[illegible]	5117
0.000732557	0.017522843	0.015711984	tags=48%, list=7 125/128/27284/	2626
0.00127998	0.026660192	0.023905054	tags=55%, list=1 4023/23586/64[illegible]	4397
1.39E-08	2.62E-06	2.35E-06	tags=51%, list=6 51474/9368/11[illegible]	2018
0.00060523	0.015243865	0.013668522	tags=71%, list=1 11332/80339/2[illegible]	4359
0.000361136	0.010703996	0.009597815	tags=42%, list=1 9619/1071/500[illegible]	281
0.000970139	0.021787714	0.019536111	tags=30%, list=7 406977/7048/4[illegible]	2695
0.000318088	0.009929495	0.008903354	tags=53%, list=6 57835/10861/8[illegible]	2370
0.000910622	0.020739105	0.018595868	tags=45%, list=1 161/1213/1514/	5527
0.000908432	0.020739105	0.018595868	tags=53%, list=1 9114/51606/53[illegible]	5048
0.000908432	0.020739105	0.018595868	tags=18%, list=4 84868/634/327[illegible]	1553
0.001503392	0.029288982	0.026262177	tags=33%, list=4 3990/55937/33[illegible]	1333
0.001140163	0.024466148	0.021937749	tags=28%, list=1 54762/64241/3[illegible]	281
7.66E-05	0.003558739	0.003190969	tags=30%, list=1 10628/3176/84[illegible]	4488
0.002323976	0.040581743	0.036387913	tags=29%, list=3 55937/336/335/	1185
0.00054917	0.014480936	0.012984436	tags=35%, list=4 3990/55937/40[illegible]	1333
4.45E-07	5.00E-05	4.48E-05	tags=74%, list=1 5160/4190/143[illegible]	6468
0.002490293	0.042838344	0.038411311	tags=38%, list=8 4846/55163/48[illegible]	2975
4.14E-07	4.78E-05	4.29E-05	tags=39%, list=1 1355/211/3658/	3976
0.000172541	0.00636985	0.005711572	tags=53%, list=1 6527/57210/26[illegible]	3820
0.00034647	0.010386287	0.009312939	tags=30%, list=3 55937/336/350/	1185
0.00034647	0.010386287	0.009312939	tags=30%, list=3 55937/336/350/	1185
0.00047752	0.013176607	0.0118149	tags=29%, list=4 27329/6620/33[illegible]	1476
0.000436663	0.012356588	0.011079624	tags=47%, list=3 9619/183/4070[illegible]	1163
0.000436663	0.012356588	0.011079624	tags=47%, list=3 9619/183/4070[illegible]	1163
0.000436663	0.012356588	0.011079624	tags=47%, list=3 9619/183/4070[illegible]	1163
0.00154464	0.029734317	0.02666149	tags=36%, list=4 3990/336/335/3	1333
0.001491681	0.029131024	0.026120543	tags=21%, list=4 84868/634/327[illegible]	1553
0.001491681	0.029131024	0.026120543	tags=21%, list=4 84868/634/327[illegible]	1553
0.001491681	0.029131024	0.026120543	tags=79%, list=1 8801/2271/594/	5074
0.001491681	0.029131024	0.026120543	tags=93%, list=2 9896/3631/529[illegible]	8294

0.001892148	0.034610906	0.031034119	tags=88%, list=2	1508/2730/151	8838
0.001350459	0.02736456	0.024536631	tags=36%, list=3	3949/6653/559	1185
0.000711836	0.017128556	0.015358443	tags=87%, list=1	10658/5914/16	5661
0.000437104	0.012356588	0.011079624	tags=61%, list=7	3046/3050/304	2441
6.28E-05	0.003040368	0.002726168	tags=82%, list=2	5162/2954/516	7762
0.001258238	0.026354535	0.023630984	tags=88%, list=2	5162/2954/516	7762
0.003123441	0.049907148	0.044749605	tags=69%, list=1	117/801/808/10	5821
0.000549864	0.014480936	0.012984436	tags=67%, list=4	230/5207/216/2	1525
0.003032618	0.048842066	0.043794592	tags=69%, list=8	4860/6568/566	2867
8.23E-05	0.003780406	0.003389728	tags=42%, list=9	641372/10965/	3399
0.000979751	0.021942621	0.01967501	tags=71%, list=1	23409/1431/34	6602
0.000282246	0.00923871	0.008283956	tags=67%, list=1	1359/2150/431	4386
1.33E-05	0.000847469	0.000759889	tags=35%, list=1	947/340348/59	3873
0.002519643	0.043068309	0.038617511	tags=77%, list=2	2639/51166/60	7818
0.002643989	0.044683273	0.04006558	tags=67%, list=1	1719/348751/1	4508
4.24E-05	0.002253552	0.002020663	tags=76%, list=1	3155/64087/11	4359
4.24E-05	0.002253552	0.002020663	tags=76%, list=1	3155/64087/11	4359
3.79E-05	0.002043265	0.001832108	tags=48%, list=9	641372/283927	3399
0.000138488	0.005409043	0.004850058	tags=59%, list=1	275/2653/2622	6333
0.00214906	0.038404542	0.03443571	tags=75%, list=1	2639/51166/10	4600
0.000778947	0.018468591	0.016559996	tags=81%, list=2	56267/51166/9	7649
0.001825619	0.033909607	0.030405294	tags=46%, list=0	80155/23173/6	144
1.00E-10	3.85E-08	3.45E-08	tags=65%, list=1	3155/6342/236	4359
1.00E-10	3.85E-08	3.45E-08	tags=65%, list=1	3155/6342/236	4359
0.001735095	0.03247278	0.029116952	tags=69%, list=1	135/5795/9353/	4761
0.001575985	0.030265657	0.02713792	tags=42%, list=4	3990/336/335/3	1333
0.000386744	0.011329081	0.010158303	tags=29%, list=0	395/5170/350/1	132
2.10E-05	0.001236838	0.00110902	tags=37%, list=6	384/23576/484	2042
0.000312455	0.009829573	0.008813758	tags=50%, list=1	7057/3818/216	273
0.000953364	0.021530581	0.019305551	tags=69%, list=9	55217/223/647	3343
0.001211815	0.02551438	0.022877653	tags=58%, list=7	125/128/219/13	2626
0.002174813	0.038560005	0.034575107	tags=50%, list=5	57818/5208/87	1787
0.002174813	0.038560005	0.034575107	tags=50%, list=5	57818/5208/87	1787
0.00265281	0.044683273	0.04006558	tags=73%, list=8	1359/4311/202	2808
0.00265281	0.044683273	0.04006558	tags=73%, list=8	1359/4311/202	2808
1.74E-05	0.001063911	0.000953963	tags=76%, list=1	501/55217/223/	3678
0.001964521	0.035772873	0.032076005	tags=55%, list=9	112817/57026/	3273
1.09E-05	0.000737923	0.000661664	tags=39%, list=1	7450/3818/216	273
0.001319986	0.02708651	0.024287315	tags=50%, list=1	7057/5340/136	219
0.001269702	0.026525948	0.023784683	tags=80%, list=7	125/128/10170/	2626
0.000338547	0.01025151	0.00919209	tags=42%, list=0	9619/336/335/3	144
5.30E-05	0.0026618	0.002386722	tags=50%, list=6	693120/81693/	2011
0.00018769	0.006772593	0.006072695	tags=54%, list=7	10965/26027/6	2455
0.000239025	0.008085847	0.007250233	tags=33%, list=3	288/4544/2363	919
0.000856897	0.02002315	0.017953902	tags=60%, list=7	10965/26027/6	2455
0.000137763	0.005406857	0.004848098	tags=46%, list=0	4023/336/350/3	144

0.000148681	0.005591095	0.005013296	tags=40%, list=4948/336/335/34	1574
0.000148681	0.005591095	0.005013296	tags=40%, list=4948/336/335/34	1574

Table S11: Information of GSEA in KEGG database

ID	Description	setSize	enrichmentScor	NES	pvalue
hsa04144	Endocytosis	252	-0.282012545	-1.242419135	0.012332441
hsa00190	Oxidative phosp	120	-0.338767279	-1.272601734	0.013246499
hsa04142	Lysosome	128	-0.353624688	-1.388219525	0.000857348
hsa01522	Endocrine resist	96	-0.362042734	-1.380261202	0.008531749
hsa04068	FoxO signaling	129	-0.371516669	-1.459658751	0.000894663
hsa03018	RNA degradatio	79	-0.374260108	-1.411492441	0.009750284
hsa00562	Inositol phospha	73	-0.424288396	-1.484360238	0.000917981
hsa01230	Biosynthesis of a	75	-0.440004744	-1.571830073	0.000533509
hsa01240	Biosynthesis of	152	-0.44554393	-1.884765139	2.11E-07
hsa00330	Arginine and pr	50	-0.511757662	-1.754011548	0.000794074
hsa00380	Tryptophan met	42	-0.525041257	-1.702926109	0.00085919
hsa00410	beta-Alanine me	30	-0.542237103	-1.671974167	0.005406014
hsa01040	Biosynthesis of	27	-0.545234171	-1.647255331	0.013714367
hsa01212	Fatty acid metab	57	-0.552885076	-1.948216529	4.60E-05
hsa00970	Aminoacyl-tRN.	25	-0.569460424	-1.72697163	0.003020524
hsa00983	Drug metabolisr	79	-0.573694289	-2.163642704	2.15E-07
hsa01200	Carbon metabol	117	-0.576791367	-2.048271662	2.25E-10
hsa00860	Porphyrin and c	41	-0.588467597	-1.859146344	0.000405591
hsa00350	Tyrosine metabo	36	-0.598817665	-1.926966335	0.000251588
hsa00040	Pentose and glu	33	-0.603943628	-1.885506187	0.000347508
hsa00010	Glycolysis / Glu	67	-0.60796331	-2.08399488	8.60E-08
hsa00650	Butanoate metab	28	-0.613626549	-1.890199764	0.001107311
hsa00140	Steroid hormon	60	-0.616100057	-2.219987536	6.61E-07
hsa00260	Glycine, serine a	40	-0.625826051	-1.96699196	3.33E-05
hsa04146	Peroxisome	83	-0.641056618	-2.33802235	2.46E-10
hsa00830	Retinol metabol	67	-0.642932771	-2.20386425	6.66E-09
hsa00980	Metabolism of x	75	-0.644529388	-2.302453984	1.45E-09
hsa00900	Terpenoid backl	22	-0.647000263	-1.873208815	0.0040463
hsa00053	Ascorbate and a	30	-0.650505682	-2.005817544	0.000171744
hsa03320	PPAR signaling	74	-0.661397557	-2.344075312	4.64E-10
hsa00220	Arginine biosyn	22	-0.668965493	-1.936803014	0.002291762
hsa00620	Pyruvate metabo	39	-0.681707373	-2.176231908	1.76E-06
hsa00982	Drug metabolisr	69	-0.685718662	-2.384265958	6.92E-10
hsa00640	Propanoate meta	33	-0.730814978	-2.281597321	1.34E-06
hsa00072	Synthesis and de	10	-0.744893969	-1.861303815	0.012093599
hsa00071	Fatty acid degra	43	-0.759754711	-2.485561789	4.19E-09
hsa00630	Glyoxylate and	29	-0.760796042	-2.340278625	1.91E-06
hsa00280	Valine, leucine a	47	-0.774775139	-2.646659515	1.00E-10
hsa00120	Primary bile aci	17	-0.775633048	-2.139060366	0.000243527
hsa00020	Citrate cycle (TC	30	-0.782898403	-2.414047097	1.81E-07

p.adjust	qvalues	leading_edge	core_enrichmen	rank
0.043488081	0.027671128	tags=44%, list=2	157/26052/229(	9280
0.045513612	0.028959957	tags=31%, list=1	4724/1353/534/	5767
0.004241706	0.002698964	tags=41%, list=2	6609/2760/175/	8142
0.032664411	0.020784111	tags=48%, list=2	5595/5601/207/	8075
0.004241706	0.002698964	tags=45%, list=1	11345/1432/40[illegible]	6784
0.036292725	0.023092778	tags=49%, list=2	29883/54464/5[illegible]	8746
0.004241706	0.002698964	tags=52%, list=2	8776/5298/517(	9276
0.002978757	0.00189536	tags=39%, list=1	2806/1491/143[illegible]	6088
2.40E-06	1.53E-06	tags=34%, list=1	51004/125061/[illegible]	4786
0.004241706	0.002698964	tags=50%, list=1	4953/26/8659/4	4946
0.004241706	0.002698964	tags=55%, list=1	26/125061/234[illegible]	4868
0.021306057	0.013556878	tags=53%, list=1	35/501/3030/51	4002
0.045943129	0.029233256	tags=37%, list=1	6342/3295/514[illegible]	4165
0.000342779	0.000218108	tags=40%, list=1	23205/84869/6[illegible]	4394
0.012648443	0.008048106	tags=72%, list=2	57038/2193/28[illegible]	9500
2.40E-06	1.53E-06	tags=32%, list=1	1571/7172/882[illegible]	3526
1.10E-08	6.99E-09	tags=57%, list=1	5211/230/2098/	6944
0.002363009	0.001503564	tags=44%, list=1	124454/211/73(	3608
0.00160537	0.001021484	tags=39%, list=8	4129/125/3081/	2988
0.00211664	0.001346802	tags=45%, list=5	51084/6652/79[illegible]	1725
1.28E-06	8.15E-07	tags=40%, list=1	130589/669/52[illegible]	4027
0.00494599	0.003147095	tags=61%, list=1	3155/35/3030/5	4359
6.82E-06	4.34E-06	tags=33%, list=4	79799/54658/6[illegible]	1350
0.000262177	0.000166821	tags=48%, list=1	10993/669/522[illegible]	4226
1.10E-08	6.99E-09	tags=49%, list=1	8504/5189/341[illegible]	5271
1.12E-07	7.10E-08	tags=49%, list=7	51109/125/822[illegible]	2645
3.23E-08	2.05E-08	tags=44%, list=9	2939/7364/545[illegible]	3239
0.016430432	0.010454556	tags=41%, list=1	79947/2224/57[illegible]	5551
0.001211244	0.000770705	tags=60%, list=4	7364/54578/22[illegible]	1404
1.55E-08	9.88E-09	tags=49%, list=1	4023/23205/63[illegible]	4397
0.009906328	0.006303319	tags=64%, list=1	2806/2744/95/2	6088
1.57E-05	1.00E-05	tags=67%, list=2	2739/5162/419[illegible]	8086
1.85E-08	1.18E-08	tags=48%, list=9	2939/7364/545[illegible]	3239
1.29E-05	8.18E-06	tags=67%, list=1	55862/32/8801/	5505
0.043488081	0.027671128	tags=70%, list=1	56898/3155/54[illegible]	5020
8.03E-08	5.11E-08	tags=60%, list=1	35/501/3030/51	4002
1.60E-05	1.02E-05	tags=66%, list=1	275/2653/4190/	6333
1.10E-08	6.99E-09	tags=74%, list=1	3155/64087/35/	4359
0.00160537	0.001021484	tags=71%, list=1	6342/23600/10[illegible]	4165
2.40E-06	1.53E-06	tags=83%, list=1	5160/4190/143[illegible]	6468

Table S12: Information of Univariate Cox regression analysis for the influencing factors of the prediction model

id	HR	HR.95L	HR.95H	pvalue
age	1.029703838	1.015880474	1.0437153	2.19E-05
grade	2.282634127	1.840530804	2.830932548	5.73E-14
stage	1.925763251	1.676994202	2.211435254	1.60E-20
T	1.972554424	1.660951738	2.3426153	9.65E-15
M	4.499325396	3.25414987	6.220957803	9.22E-20
riskScore	4.599414555	3.284982951	6.439794228	6.33E-19

Table S13: Information of Multivariate Cox regression analysis for the influencing factors of the prediction model

id	HR	HR.95L	HR.95H	pvalue
age	1.030638635	1.01544576	1.046058823	6.81E-05
grade	1.333392181	1.04228896	1.705798273	0.022047219
stage	1.69827253	1.062221658	2.7151862	0.02696064
T	0.858816975	0.556418057	1.325561936	0.491904517
M	1.279259965	0.640501426	2.555038898	0.485324592
riskScore	3.272507928	2.14905146	4.98327208	3.29E-08

Table S14: Tumor mutation burden (TMB) value of each samples

id	TMB
TCGA-BP-5168	2.289473684
TCGA-CJ-4913	0.973684211
TCGA-BP-5010	1.263157895
TCGA-B0-5692	1.394736842
TCGA-G6-A8L6	1.315789474
TCGA-B0-5077	1.026315789
TCGA-B4-5843	0.868421053
TCGA-B8-5158	0.842105263
TCGA-A3-3380	0.631578947
TCGA-6D-AA2E	0.368421053
TCGA-A3-3346	1.789473684
TCGA-A3-3385	1.263157895
TCGA-DV-5565	1.236842105
TCGA-CJ-4901	0.921052632
TCGA-CJ-5679	1.921052632
TCGA-BP-5006	0.710526316
TCGA-CJ-6027	1.526315789
TCGA-B8-4153	1.157894737
TCGA-CZ-5457	1.552631579
TCGA-BP-4177	0.184210526
TCGA-B8-4143	1.210526316
TCGA-B8-5551	0.710526316
TCGA-A3-A6NJ	1.052631579
TCGA-B0-4827	1.684210526
TCGA-BP-4965	1.105263158
TCGA-CZ-4853	2.263157895
TCGA-B2-5641	1.289473684
TCGA-CJ-4869	1.578947368
TCGA-CJ-5689	1.394736842
TCGA-BP-5196	0.947368421
TCGA-CZ-5461	1.447368421
TCGA-B0-5119	1.921052632
TCGA-B0-5121	0.894736842
TCGA-B0-5698	1.526315789
TCGA-A3-A6NL	0.789473684
TCGA-BP-5201	0.868421053
TCGA-CJ-6030	2.236842105
TCGA-B8-5163	1.289473684
TCGA-A3-3316	0.868421053
TCGA-A3-3374	0.210526316
TCGA-BP-4964	1.394736842
TCGA-MW-A4EC	0.789473684
TCGA-A3-3322	1.026315789
TCGA-BP-5181	1.184210526
TCGA-A3-3370	0.789473684
TCGA-BP-4960	1.236842105
TCGA-CJ-4903	0.973684211

TCGA-BP-5000	0.763157895
TCGA-CW-6097	0.236842105
TCGA-CZ-5989	0.763157895
TCGA-CJ-6028	0.815789474
TCGA-BP-5004	0.947368421
TCGA-CZ-5458	0.552631579
TCGA-B8-4151	1.184210526
TCGA-BP-4998	0.631578947
TCGA-B8-5553	1
TCGA-DV-5566	0.842105263
TCGA-B0-5099	1.315789474
TCGA-B0-5709	1.342105263
TCGA-B2-5633	0.921052632
TCGA-3Z-A93Z	1.789473684
TCGA-BP-5173	1.552631579
TCGA-B8-A54E	0.947368421
TCGA-CW-5589	1
TCGA-BP-4992	0.815789474
TCGA-B0-4700	0.263157895
TCGA-BP-4782	1.684210526
TCGA-DV-A4VZ	0.210526316
TCGA-CZ-5452	0.605263158
TCGA-BP-5183	0.973684211
TCGA-B0-5109	0.973684211
TCGA-BP-5195	1.368421053
TCGA-A3-3376	0.815789474
TCGA-B0-5088	1.026315789
TCGA-B2-4102	1.578947368
TCGA-BP-5190	0.815789474
TCGA-AK-3465	0.578947368
TCGA-CZ-5463	1
TCGA-BP-5194	0.394736842
TCGA-BP-4989	1.236842105
TCGA-B0-5697	1.052631579
TCGA-B0-5085	1.342105263
TCGA-BP-5198	1.605263158
TCGA-B2-5639	1.052631579
TCGA-B0-5703	1.526315789
TCGA-A3-A8CQ	0.657894737
TCGA-B0-5696	0.921052632
TCGA-CJ-4923	0.868421053
TCGA-BP-4770	1.078947368
TCGA-BP-4973	0.631578947
TCGA-B8-5549	1.105263158
TCGA-B0-5711	0.763157895
TCGA-B0-5084	1
TCGA-A3-3363	0.973684211
TCGA-T7-A92I	0.684210526
TCGA-BP-4967	1.342105263

TCGA-CJ-5671	1.131578947
TCGA-AK-3453	0.184210526
TCGA-DV-5573	0.578947368
TCGA-G6-A8L8	1.5
TCGA-AK-3443	0.105263158
TCGA-BP-4995	0.868421053
TCGA-BP-4983	1.131578947
TCGA-CZ-5455	0.684210526
TCGA-B8-4621	1.763157895
TCGA-CJ-4899	0.605263158
TCGA-CJ-5681	0.342105263
TCGA-A3-3358	1.289473684
TCGA-CZ-5469	0.894736842
TCGA-BP-5192	1.052631579
TCGA-DV-5567	0.315789474
TCGA-A3-3387	1.368421053
TCGA-BP-5008	0.710526316
TCGA-CZ-5454	0.526315789
TCGA-BP-5189	1
TCGA-B0-5075	1.921052632
TCGA-CZ-4866	1.605263158
TCGA-MM-A564	1.921052632
TCGA-CJ-4882	1.368421053
TCGA-CW-5583	0.526315789
TCGA-A3-3326	0.973684211
TCGA-B0-5713	2.026315789
TCGA-BP-4971	0.763157895
TCGA-B0-5695	1.131578947
TCGA-A3-3317	1.394736842
TCGA-A3-3378	1.184210526
TCGA-BP-4968	0.868421053
TCGA-B0-4842	1.105263158
TCGA-B0-5701	2.184210526
TCGA-A3-3382	2.026315789
TCGA-B0-5694	1.342105263
TCGA-A3-3308	1.605263158
TCGA-B8-A54J	1.315789474
TCGA-CZ-5470	0.815789474
TCGA-A3-A8OW	0.447368421
TCGA-B0-5690	1.052631579
TCGA-DV-A4VX	1.763157895
TCGA-BP-4962	0.736842105
TCGA-CW-6090	1.947368421
TCGA-BP-5007	0.578947368
TCGA-CZ-4864	1.473684211
TCGA-GK-A6C7	2.026315789
TCGA-A3-3372	1.184210526
TCGA-G6-A5PC	1.184210526
TCGA-A3-3320	1.605263158

TCGA-CW-5581	1.263157895
TCGA-CZ-5456	1.315789474
TCGA-CZ-4865	1.789473684
TCGA-G6-A8L7	1.394736842
TCGA-CJ-6032	0.973684211
TCGA-BP-4981	0.868421053
TCGA-B0-5113	0.684210526
TCGA-A3-3357	1.868421053
TCGA-CJ-5683	1.236842105
TCGA-DV-5568	0.289473684
TCGA-CZ-4859	1.894736842
TCGA-B0-5107	1.210526316
TCGA-CJ-5672	1.947368421
TCGA-BP-5001	0.631578947
TCGA-B0-5712	1.894736842
TCGA-CW-5587	1.315789474
TCGA-B8-5546	0.184210526
TCGA-B0-4945	0.842105263
TCGA-BP-4987	0.447368421
TCGA-B0-5699	0.789473684
TCGA-AK-3447	0.394736842
TCGA-B4-5844	1.210526316
TCGA-A3-3383	1.131578947
TCGA-B2-5636	0.342105263
TCGA-B0-5812	0.868421053
TCGA-BP-5176	2.447368421
TCGA-B0-5402	1.236842105
TCGA-A3-3367	1.263157895
TCGA-A3-3311	1.157894737
TCGA-BP-4977	0.842105263
TCGA-B8-A54F	0.5
TCGA-BP-4963	1.631578947
TCGA-BP-5186	0.657894737
TCGA-B4-5835	1.473684211
TCGA-CJ-5678	1
TCGA-B8-5164	1.605263158
TCGA-CZ-5987	1.026315789
TCGA-A3-3373	1.184210526
TCGA-EU-5906	1.263157895
TCGA-CJ-5682	1.552631579
TCGA-B0-5097	1.157894737
TCGA-B8-5159	1.078947368
TCGA-CZ-5466	1.368421053
TCGA-B0-5707	0.763157895
TCGA-BP-5191	1.157894737
TCGA-BP-4760	0.131578947
TCGA-A3-3331	1.315789474
TCGA-B8-5165	0.289473684
TCGA-BP-5169	0.973684211

TCGA-CJ-6033	1.447368421
TCGA-B8-4622	1.078947368
TCGA-B4-5834	0.578947368
TCGA-BP-4988	0.552631579
TCGA-B0-5117	0.210526316
TCGA-CJ-4916	0.921052632
TCGA-BP-5174	0.868421053
TCGA-B8-5545	0.394736842
TCGA-CJ-4905	1.052631579
TCGA-B0-5702	0.973684211
TCGA-BP-5170	0.842105263
TCGA-B0-5092	0.973684211
TCGA-CW-6087	0.921052632
TCGA-CZ-5982	0.868421053
TCGA-B0-5108	0.815789474
TCGA-CJ-4900	1.105263158
TCGA-B2-5635	1.052631579
TCGA-BP-5175	0.736842105
TCGA-BP-4972	0.736842105
TCGA-A3-A8OV	3.026315789
TCGA-CW-5588	0.921052632
TCGA-B8-5550	1.894736842
TCGA-B8-4146	0.710526316
TCGA-CJ-4904	0.763157895
TCGA-CZ-5460	1.289473684
TCGA-BP-5185	1.710526316
TCGA-CZ-4856	1.078947368
TCGA-B4-5836	1.236842105
TCGA-B0-5098	13.47368421
TCGA-EU-5905	1.289473684
TCGA-BP-5200	0.842105263
TCGA-CJ-6031	1.394736842
TCGA-B0-5120	1
TCGA-BP-4982	0.736842105
TCGA-CZ-5459	1.921052632
TCGA-A3-3313	1.868421053
TCGA-EU-5904	0.710526316
TCGA-BP-5184	0.526315789
TCGA-BP-4999	0.973684211
TCGA-B4-5377	0.789473684
TCGA-B8-A54K	0.289473684
TCGA-A3-A6NI	1.605263158
TCGA-BP-4961	0.657894737
TCGA-CJ-5677	1.473684211
TCGA-CZ-5465	2.263157895
TCGA-B0-5102	0.921052632
TCGA-BP-5180	0.894736842
TCGA-CZ-5988	0.789473684
TCGA-BP-4970	0.605263158

TCGA-CZ-5984	0.842105263
TCGA-B8-5552	0.578947368
TCGA-B0-5083	0.131578947
TCGA-CJ-4902	1.236842105
TCGA-B8-A54H	1.5
TCGA-BP-5009	0.868421053
TCGA-B0-5705	1.605263158
TCGA-CJ-5676	1.157894737
TCGA-BP-4974	0.605263158
TCGA-B0-5095	1.5
TCGA-CZ-5985	1.052631579
TCGA-CZ-5468	2.394736842
TCGA-AS-3777	0.289473684
TCGA-B0-5700	1.473684211
TCGA-BP-4975	0.315789474
TCGA-CJ-4920	3.078947368
TCGA-B0-5094	1.736842105
TCGA-MM-A84U	1.684210526
TCGA-A3-3365	0.789473684
TCGA-B0-5691	1.157894737
TCGA-B0-5100	0.657894737
TCGA-MM-A563	1.026315789
TCGA-BP-5182	1.315789474
TCGA-AK-3427	0.210526316
TCGA-CJ-4908	0.5
TCGA-B0-4823	2.210526316
TCGA-AK-3455	1.263157895
TCGA-B0-5104	1.078947368
TCGA-BP-4795	0.315789474
TCGA-BP-4993	1.026315789
TCGA-CW-5580	1.710526316
TCGA-DV-5575	0.447368421
TCGA-B2-A4SR	0.868421053
TCGA-BP-4985	1.447368421
TCGA-CZ-5453	1.447368421
TCGA-A3-3319	1.5
TCGA-B8-A54I	1.210526316
TCGA-B8-A54D	1.184210526
TCGA-B8-A7U6	1
TCGA-BP-5202	0.763157895
TCGA-CW-5585	0.921052632
TCGA-AK-3440	0.394736842
TCGA-B0-5116	1.263157895
TCGA-BP-5178	1.157894737
TCGA-BP-5199	1.578947368
TCGA-CJ-5686	1.263157895
TCGA-CZ-5462	1.315789474
TCGA-A3-A8OU	1.473684211
TCGA-AK-3444	1.815789474

TCGA-DV-5574	0.184210526
TCGA-BP-4801	1.289473684
TCGA-B0-5706	1.131578947
TCGA-CZ-5467	1.052631579
TCGA-CJ-5675	1.210526316
TCGA-B4-5838	1.342105263
TCGA-B0-5096	1.605263158
TCGA-AS-3778	1.184210526
TCGA-DV-5569	0.157894737
TCGA-B0-5399	0.710526316
TCGA-CJ-4912	1.631578947
TCGA-B0-5693	0.815789474
TCGA-B8-A8YJ	0.026315789
TCGA-BP-4976	1.868421053
TCGA-A3-A8OX	0.157894737
TCGA-B0-5710	0.789473684
TCGA-EU-5907	0.973684211
TCGA-CZ-5986	1.157894737
TCGA-B0-5081	0.447368421
TCGA-BP-5187	1
TCGA-CW-5591	0.631578947
TCGA-B0-5110	1.236842105
TCGA-DV-5576	0.026315789
TCGA-CJ-4918	1.5
TCGA-CJ-5684	0.789473684
TCGA-BP-4986	0.789473684
TCGA-BP-5177	0.894736842
TCGA-B4-5832	1.157894737
TCGA-B8-A54G	0.789473684
TCGA-DV-A4W0	1.342105263
TCGA-CJ-5680	0.973684211
TCGA-A3-A6NN	2.131578947
TCGA-B0-5106	1.736842105
TCGA-CW-6093	2.184210526
TCGA-B2-4101	0.763157895
TCGA-CJ-4907	1.131578947
TCGA-B8-5162	1.210526316
TCGA-CZ-4863	0.815789474
TCGA-BP-4991	0.842105263
TCGA-B8-4148	0.657894737
TCGA-CZ-5451	1.657894737
TCGA-B0-5400	0.605263158
TCGA-B0-5115	1.236842105
TCGA-A3-3323	0.947368421
